# A SILAC-Based Approach Elicits the Proteomic Responses to Vancomycin-Associated Nephrotoxicity in Human Proximal Tubule Epithelial HK-2 Cells

**DOI:** 10.3390/molecules21020148

**Published:** 2016-01-29

**Authors:** Zhi-Ling Li, Shu-Feng Zhou

**Affiliations:** 1 Department of Pharmacy, Shanghai Children’s Hospital, Shanghai Jiao Tong University, Shanghai 200040, China; 2 Department of Pharmaceutical Sciences, College of Pharmacy, University of South Florida, Tampa, FL 33612, USA

**Keywords:** vancomycin, nephrotoxicity, SILAC, proteomics

## Abstract

Vancomycin, a widely used antibiotic, often induces nephrotoxicity, however, the molecular targets and underlying mechanisms of this side effect remain unclear. The present study aimed to examine molecular interactome and analyze the signaling pathways related to the vancomycin-induced nephrotoxicity in human proximal tubule epithelial HK-2 cells using the stable isotope labeling by amino acids in cell culture (SILAC) approach. The quantitative proteomic study revealed that there were at least 492 proteins interacting with vancomycin and there were 290 signaling pathways and cellular functions potentially regulated by vancomycin in HK-2 cells. These proteins and pathways played a critical role in the regulation of cell cycle, apoptosis, autophagy, EMT, and ROS generation. These findings suggest that vancomycin-induced proteomic responses in HK-2 cells involvefunctional proteins and pathways that regulate cell cycle, apoptosis, autophagy, and redox homeostasis. This is the first systemic study revealed the networks of signaling pathways and proteomic responses to vancomycin treatment in HK-2 cells, and the data may be used to discriminate the molecular and clinical subtypes and to identify new targets and biomarkers for vancomycin-induced nephrotoxic effect. Further studies are warranted to explore the potential of quantitative proteomic analysis in the identification of new targets and biomarkers for drug-induced renal toxicity.

## 1. Introduction

Neonatal sepsis is common and is a major cause of morbidity and mortality [1]. Vancomycin is the preferred treatment for several neonatal staphylococcal infections. It remains the primary antibiotic treatment for multi-resistant Gram-positive infections, such as methicillin-resistant *Staphylococcus aureus* (MRSA) and *Enterococcus faecium* [2]. Vancomycin pharmacokinetic estimates, which are different in neonates compared with adults, also exhibit extensive inter-neonatal variability [3]. In neonates, several vancomycin dosing schedules have been proposed, mainly based on age (*i.e*., postmenstrual and postnatal), body weight, or serum creatinine level. Although vancomycin has historically been linked to various toxicities, in particular nephrotoxicity, it was largely attributed to drug impurities in early formulations [4,5]. The incidence of such toxicities was drastically reduced after refinement of purification methods and the risk of nephrotoxicity was considered relatively low at less than 5% [5,6,7].

However, the molecular targets for vancomycin-associated nephrotoxicity are unclear. There is a lack of study which reveals the global targets of vancomycin with regard to its renal toxicity, although the characterization and identification of individual targets and related signaling pathways have provided important evidence for the mechanism of actions of vancomycin *in vitro* and *in vivo*. Stable isotope labeling by amino acids in cell culture (SILAC) is a practical and powerful approach to uncover the global proteomic responses to drug treatment and other interventions [8,9]. In particular, it can be used to systemically and quantitatively assess the target network of drugs, evaluate drug toxicity, and identify new biomarkers for the diagnosis and treatment of importance diseases such as cancer and Alzheimer’s disease [8,10,11].

In this regard, in order to uncover the comprehensive and global understanding on the effect of vancomycin, we investigated the molecular targets of vancomycin in human proximal tubule epithelial HK-2 cells with a focus on cell cycle, apoptosis, autophagy, and epithelial to mesenchymal transition (EMT) pathways.

## 2. Results

### 2.1. Overview of Proteomic Response to Vancomycin Treatment in HK-2 Cells

**Figure 1:**
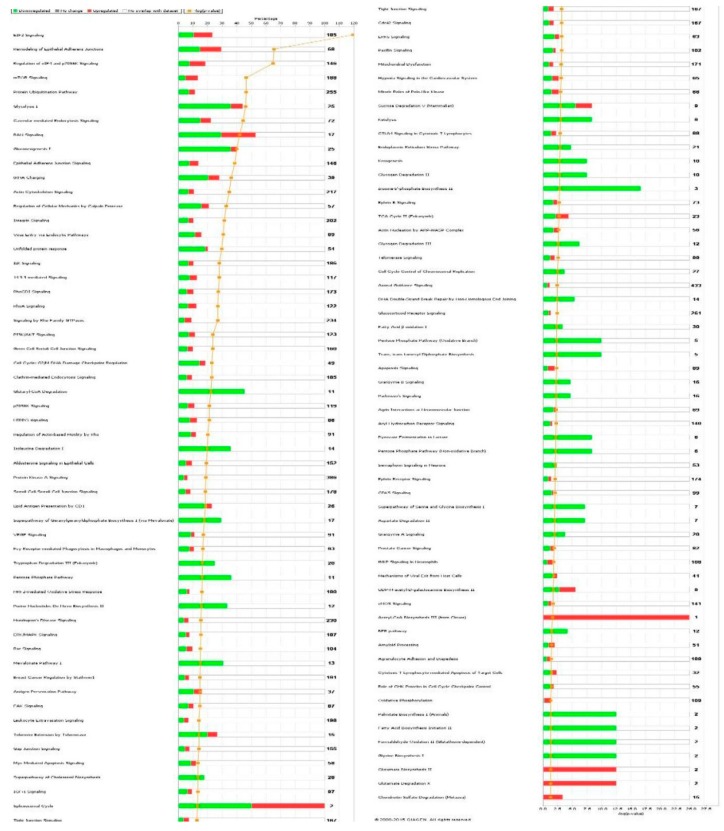
Proteomic analysis revealed molecular interactome regulated by vancomycin in HK-2 cells. HK-2 cells were treated with 50 μg/mL vancomycin for 24 h and the protein samples were subject to quantitative proteomic analysis. There were 290 related pathways regulated by vancomycin in HK-2 cells. Red indicates an up-regulation; green indicates a down-regulation.

**Figure 2:**
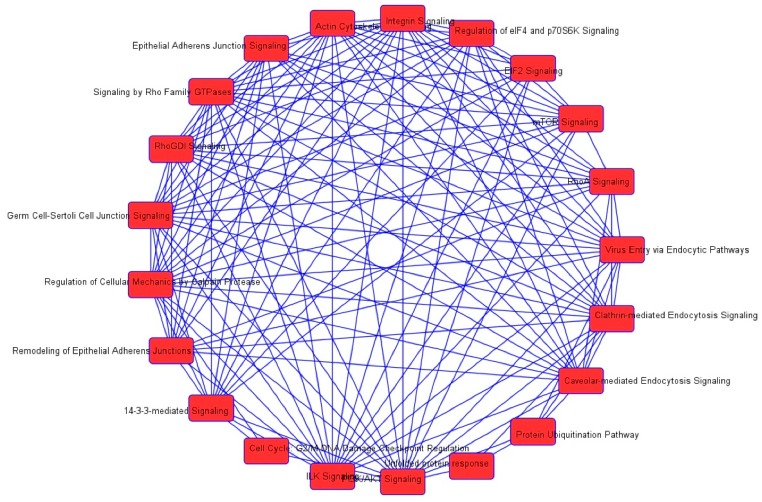
Intergrated signaling pathways regulated by vancomycin in HK-2 cells.

**Figure 3:**
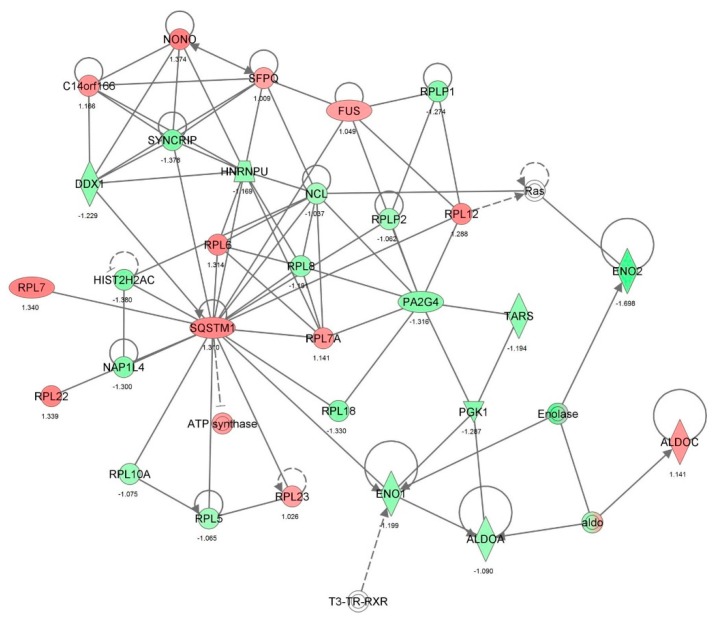
Vancomycin regulated RNA post-transcriptional modification, carbohydrate metabolism, and small molecule biochemistry. Red indicates an up-regulation; green indicates a down-regulation. The intensity of green and red colors indicates the degree of down- or up-regulation. Solid arrow indicates direct interaction and dashed arrow indicates indirect interaction.

**Figure 4:**
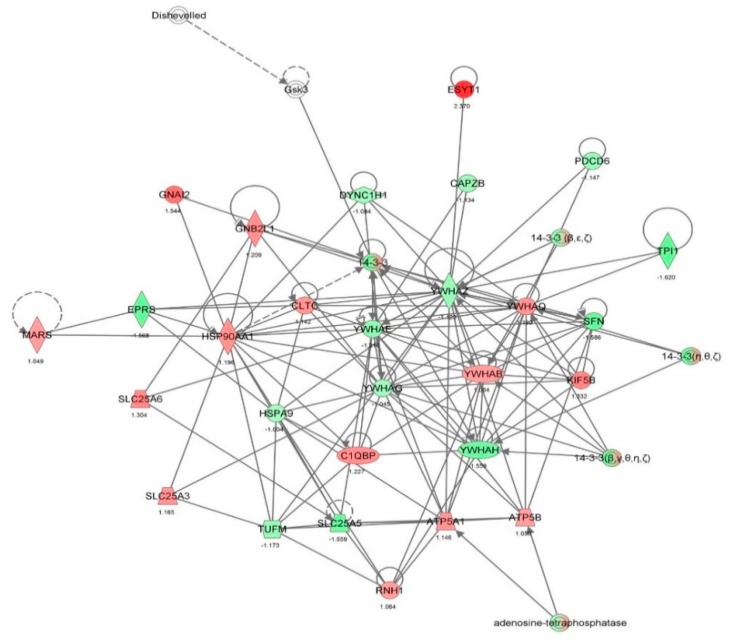
Vancomycin regulated protein trafficking, cell death and survival, and nucleic acid metabolism. Red indicates an up-regulation; green indicates a down-regulation. The intensity of green and red colors indicates the degree of down- or up-regulation. Solid arrow indicates direct interaction and dashed arrow indicates indirect interaction.

**Figure 5:**
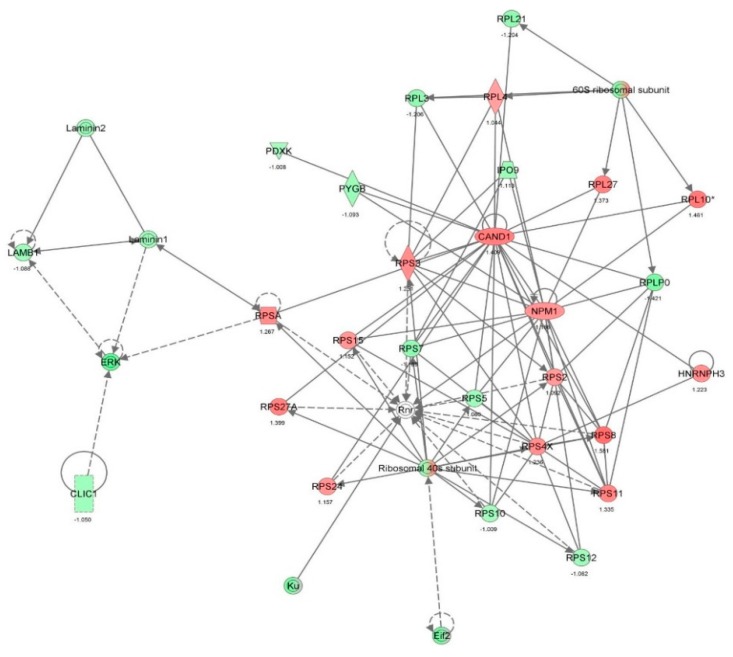
Vancomycin regulated hematological disease, organismal injury and abnormalities, and RNA post-transcriptional modification. Red indicates an up-regulation; green indicates a down-regulation. The intensity of green and red colors indicates the degree of down- or up-regulation. Solid arrow indicates direct interaction and dashed arrow indicates indirect interaction.

**Figure 6:**
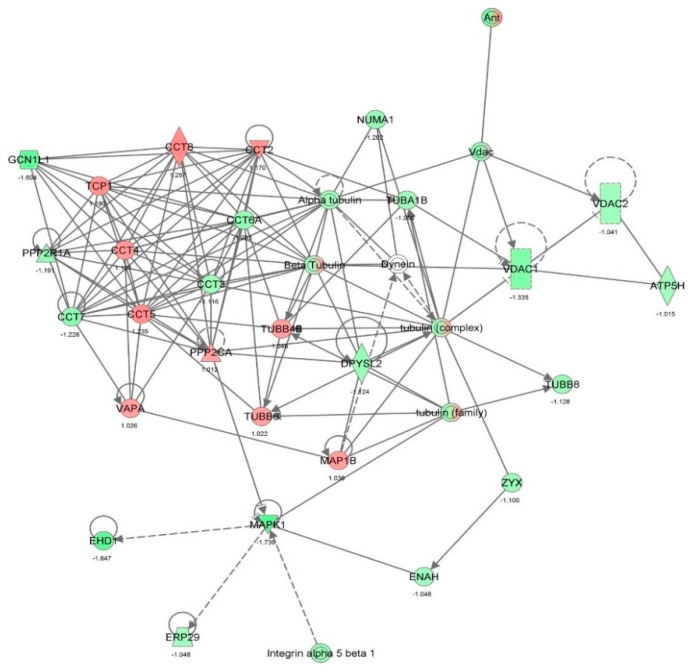
Vancomycin regulated cellular assembly and organization, cell-to-cell signaling and interaction, and reproductive system development and function. Red indicates an up-regulation; green indicates a down-regulation. The intensity of green and red colors indicates the degree of down- or up-regulation. Solid arrow indicates direct interaction and dashed arrow indicates indirect interaction.

**Figure 7:**
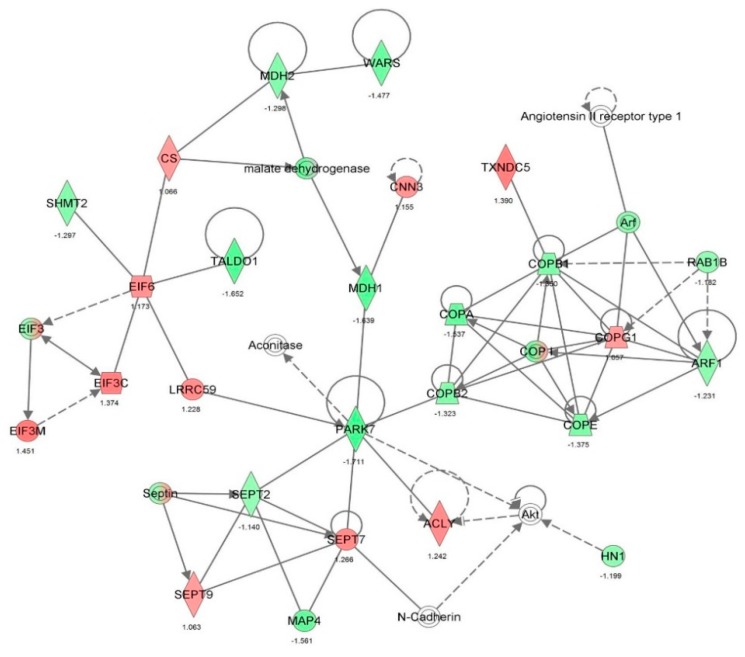
Vancomycin regulated infectious disease, small molecule biochemistry, and reproductive system disease. Red indicates an up-regulation; green indicates a down-regulation. The intensity of green and red colors indicates the degree of down- or up-regulation. Solid arrow indicates direct interaction and dashed arrow indicates indirect interaction.

**Figure 8:**
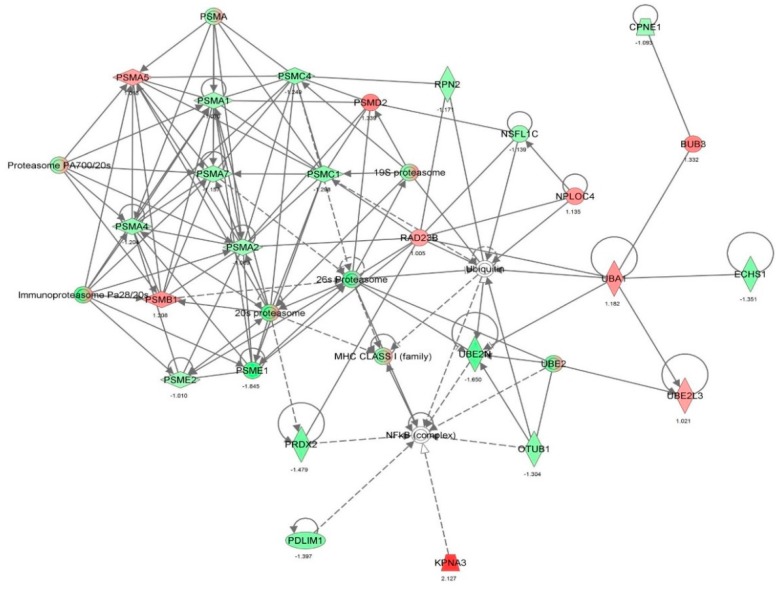
Vancomycin regulated infectious disease, organismal injury and abnormalities, and immunological disease. Red indicates an up-regulation; green indicates a down-regulation. The intensity of green and red colors indicates the degree of down- or up-regulation. Solid arrow indicates direct interaction and dashed arrow indicates indirect interaction.

**Figure 9:**
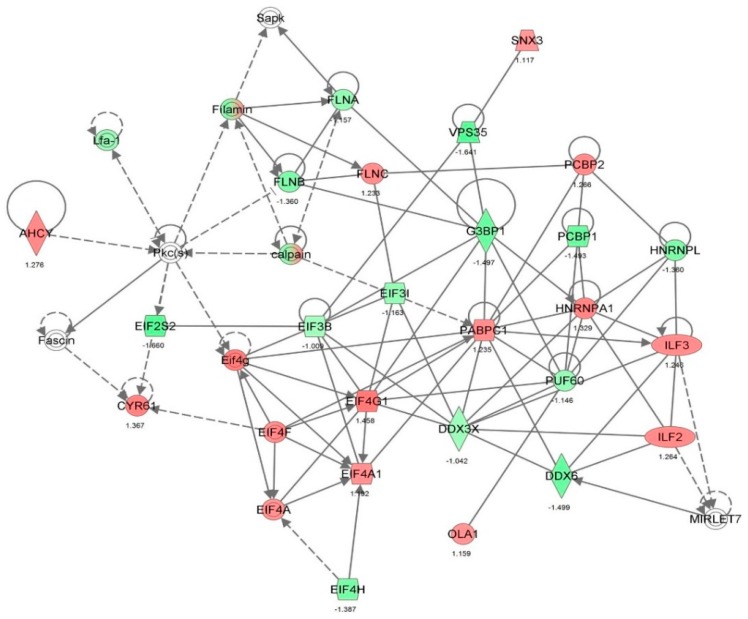
Vancomycin regulated protein synthesis, cellular movement, and gene expression. Red indicates an up-regulation; green indicates a down-regulation. The intensity of green and red colors indicates the degree of down- or up-regulation. Solid arrow indicates direct interaction and dashed arrow indicates indirect interaction.

**Figure 10:**
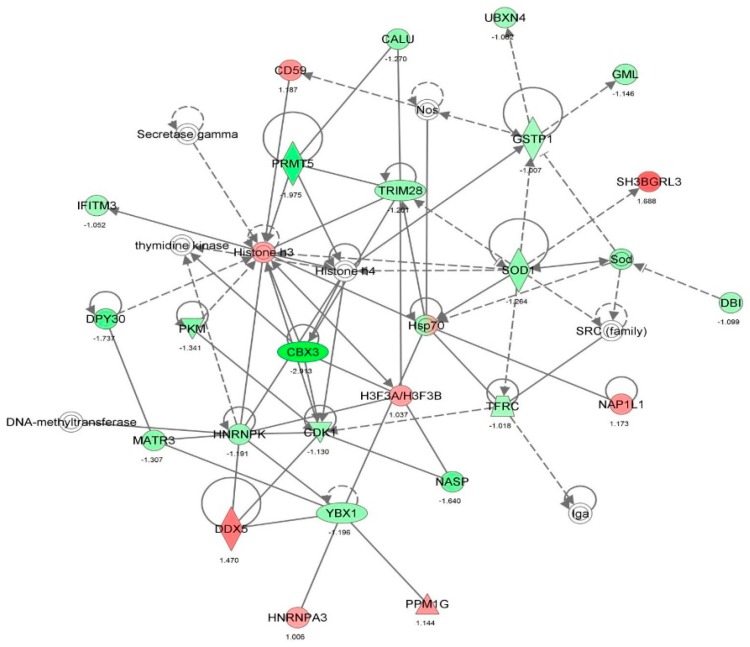
Vancomycin regulated cellular growth and proliferation, infectious disease, and cellular development. Red indicates an up-regulation; green indicates a down-regulation. The intensity of green and red colors indicates the degree of down- or up-regulation. Solid arrow indicates direct interaction and dashed arrow indicates indirect interaction.

**Figure 11:**
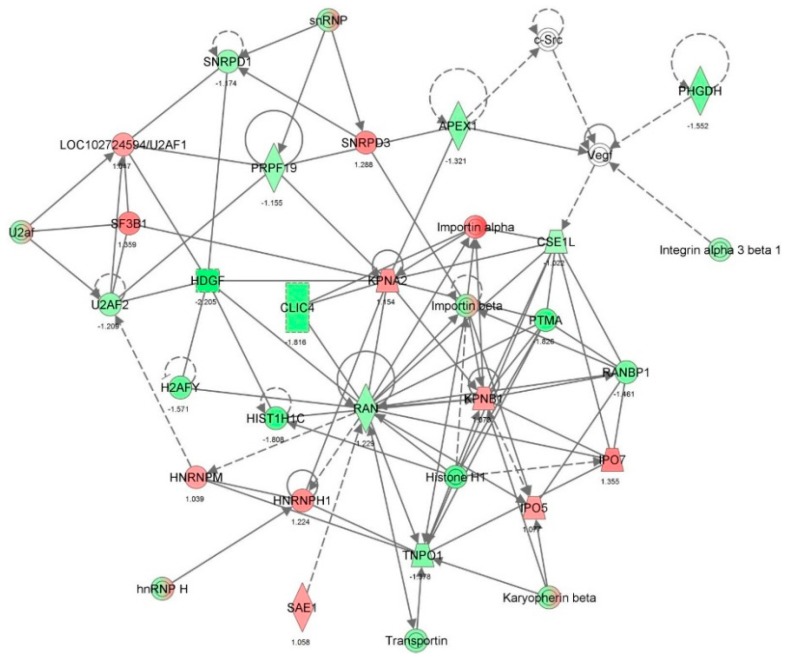
Vancomycin regulated molecular transport, protein trafficking, and RNA post-transcriptional modification. Red indicates an up-regulation; green indicates a down-regulation. The intensity of green and red colors indicates the degree of down- or up-regulation. Solid arrow indicates direct interaction and dashed arrow indicates indirect interaction.

**Figure 12:**
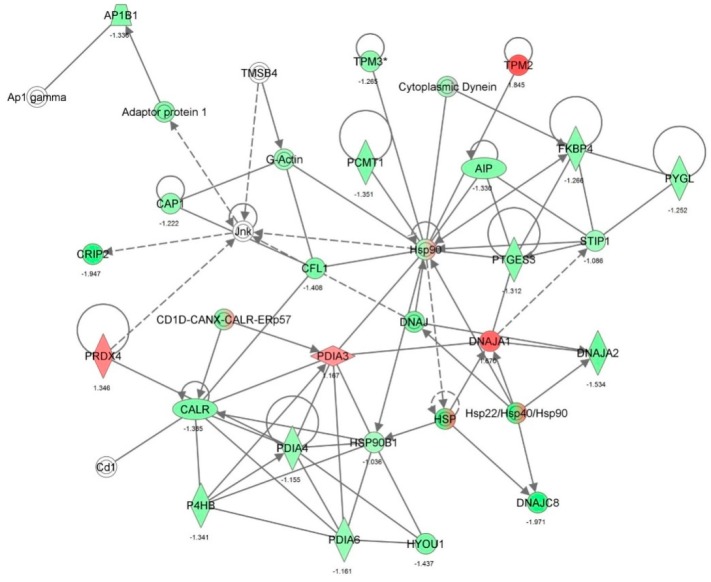
Vancomycin regulated post-translational modification, protein folding, and drug metabolism. Red indicates an up-regulation; green indicates a down-regulation. The intensity of green and red colors indicates the degree of down- or up-regulation. Solid arrow indicates direct interaction and dashed arrow indicates indirect interaction.

**Figure 13:**
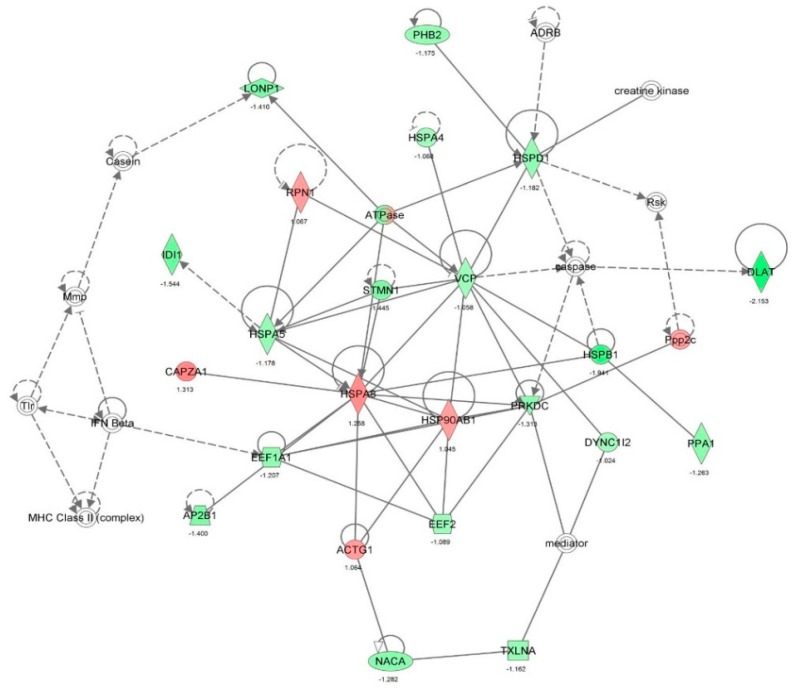
Vancomycin regulated post-translational modification, protein folding, and cell morphology. Red indicates an up-regulation; green indicates a down-regulation. The intensity of green and red colors indicates the degree of down- or up-regulation. Solid arrow indicates direct interaction and dashed arrow indicates indirect interaction.

**Figure 14:**
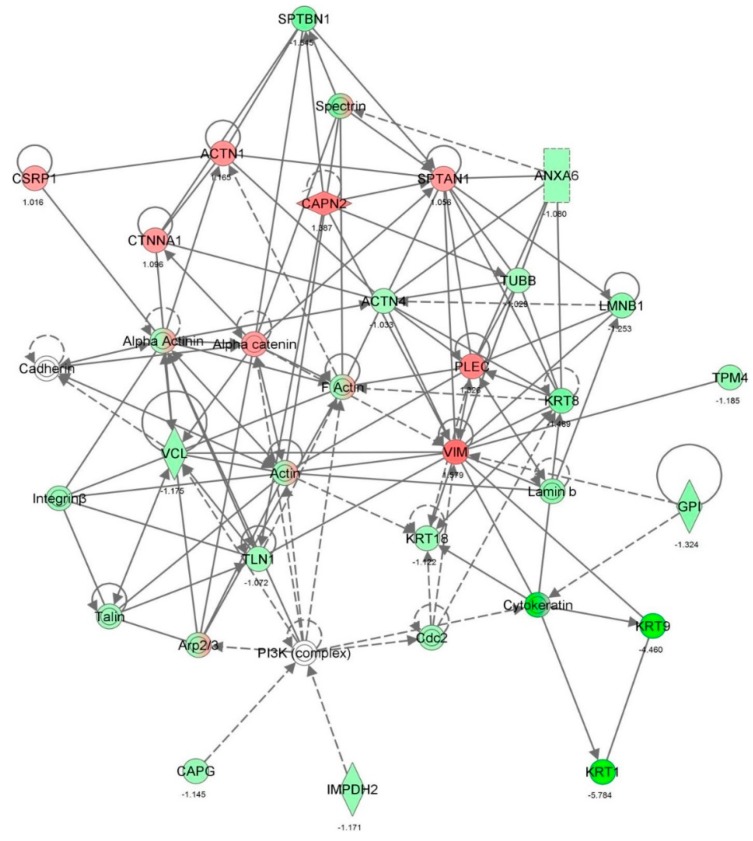
Vancomycin regulated cellular compromise, cellular assembly and organization, and cellular function and maintenance. Red indicates an up-regulation; green indicates a down-regulation. The intensity of green and red colors indicates the degree of down- or up-regulation. Solid arrow indicates direct interaction and dashed arrow indicates indirect interaction.

**Figure 15:**
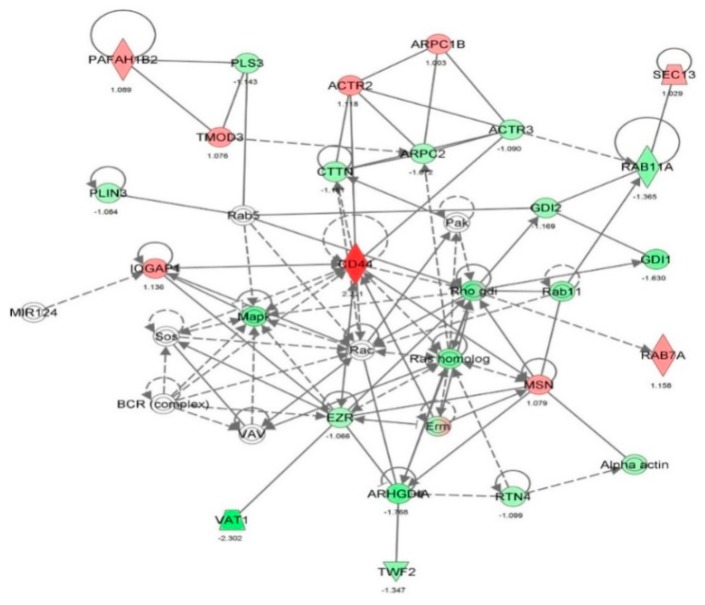
Vancomycin regulated cellular assembly and organization, cellular function and maintenance, and cell morphology. Red indicates an up-regulation; green indicates a down-regulation. The intensity of green and red colors indicates the degree of down- or up-regulation. Solid arrow indicates direct interaction and dashed arrow indicates indirect interaction.

**Figure 16:**
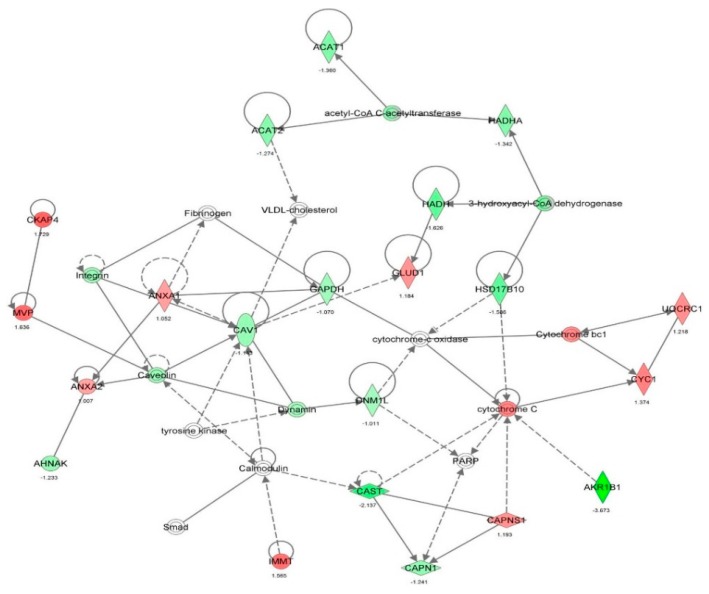
Vancomycin regulated endocrine system development and function, lipid metabolism, and small molecule biochemistry. Red indicates an up-regulation; green indicates a down-regulation. The intensity of green and red colors indicates the degree of down- or up-regulation. Solid arrow indicates direct interaction and dashed arrow indicates indirect interaction.

**Figure 17:**
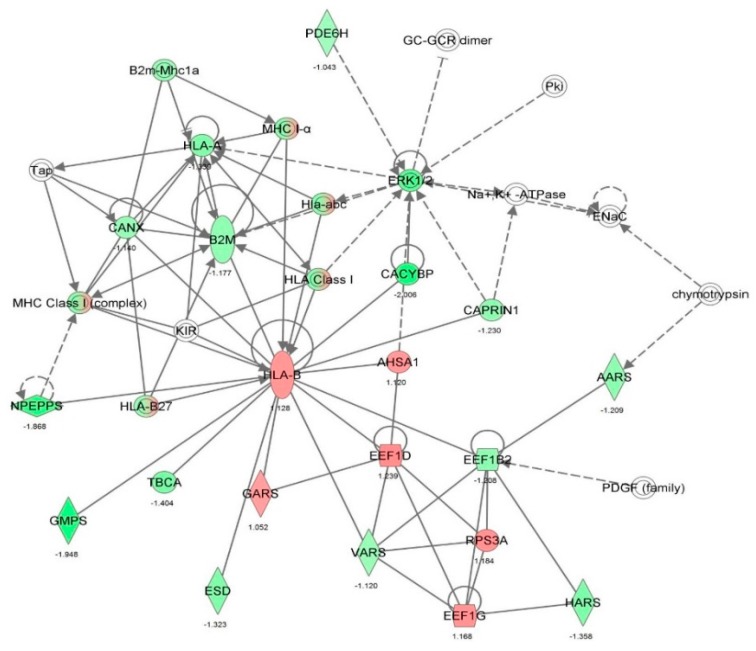
Vancomycin regulated post-translational modification, protein folding, and dermatological diseases and conditions. Red indicates an up-regulation; green indicates a down-regulation. The intensity of green and red colors indicates the degree of down- or up-regulation. Solid arrow indicates direct interaction and dashed arrow indicates indirect interaction.

**Figure 18:**
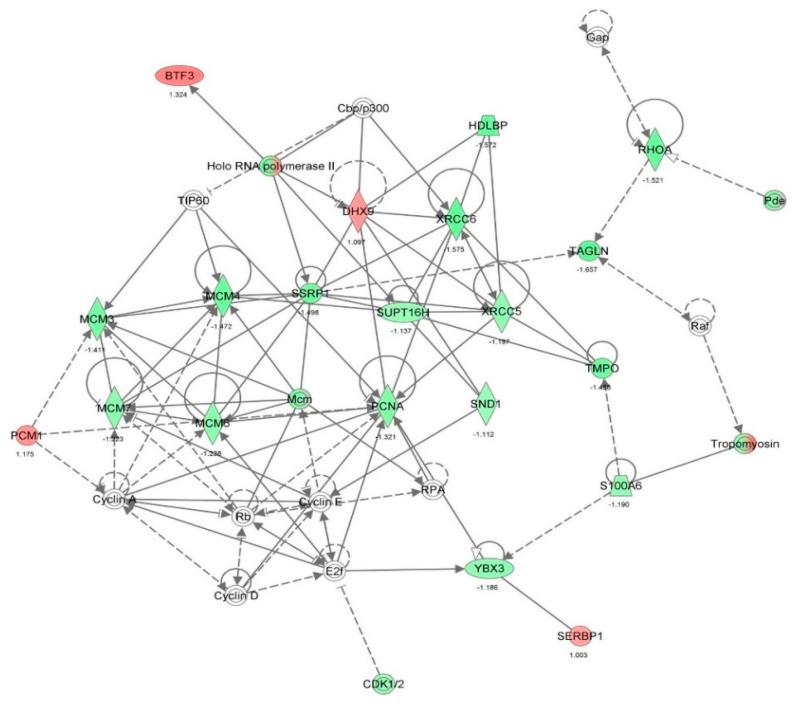
Vancomycin regulated DNA replication, recombination, and repair, cell morphology, and cellular function and maintenance. Red indicates an up-regulation; green indicates a down-regulation. The intensity of green and red colors indicates the degree of down- or up-regulation. Solid arrow indicates direct interaction and dashed arrow indicates indirect interaction.

**Figure 19:**
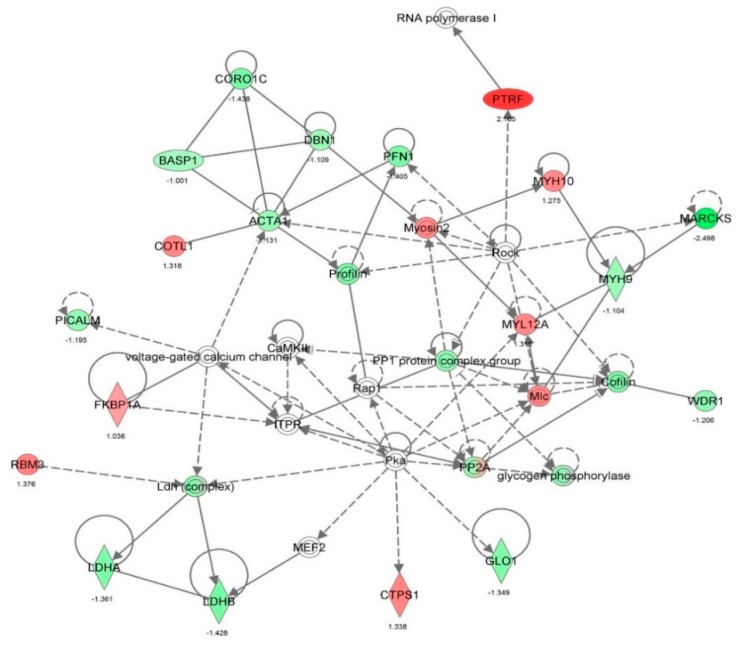
Vancomycin regulated cellular movement, connective tissue disorders, and hematological disease. Red indicates an up-regulation; green indicates a down-regulation. The intensity of green and redcolors indicates the degree of down- or up-regulation. Solid arrow indicates direct interaction and dashed arrow indicates indirect interaction.

**Figure 20:**
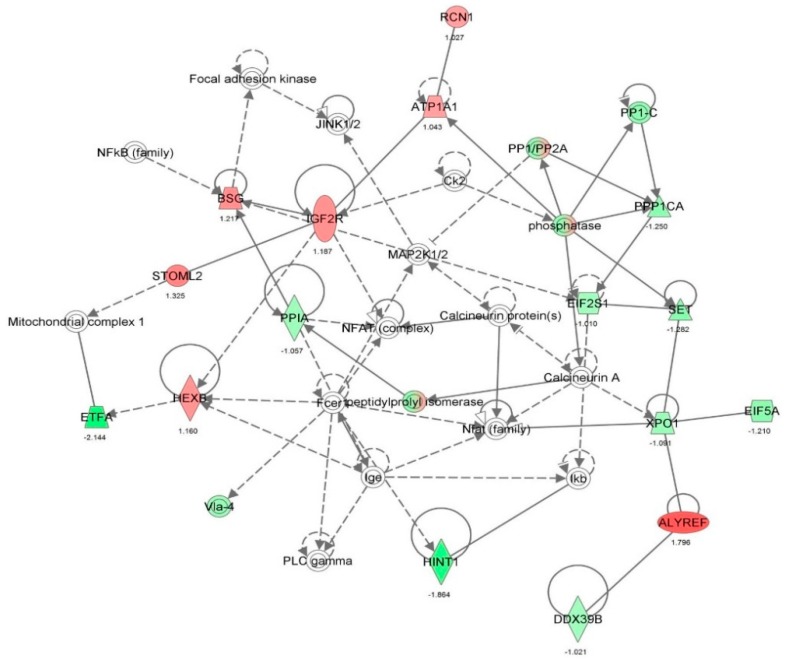
Vancomycin regulated molecular transport, RNA trafficking, and cell death and survival. Red indicates an up-regulation; green indicates a down-regulation. The intensity of green and red colors indicates the degree of down- or up-regulation. Solid arrow indicates direct interaction and dashed arrow indicates indirect interaction.

**Figure 21:**
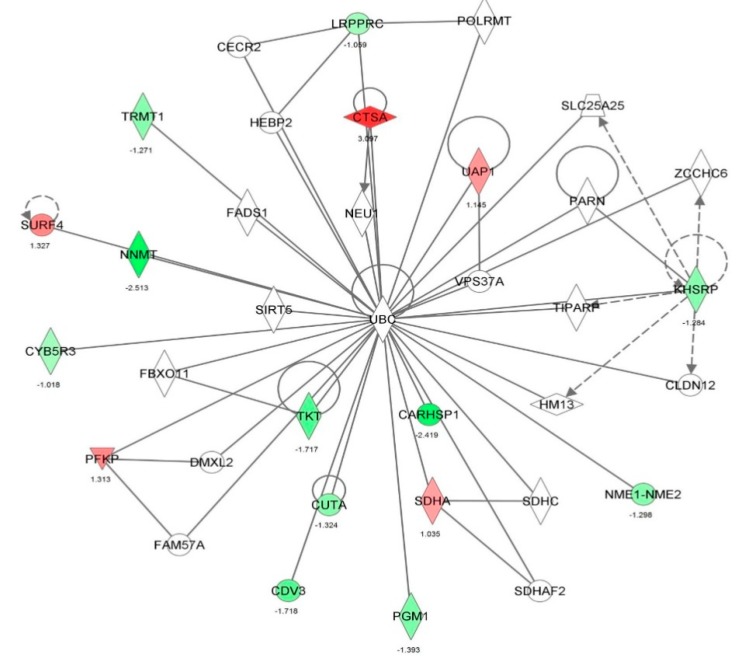
Vancomycin regulated developmental disorder, hereditary disorder, and metabolic disease. Red indicates an up-regulation; green indicates a down-regulation. The intensity of green and red colors indicates the degree of down- or up-regulation. Solid arrow indicates direct interaction and dashed arrow indicates indirect interaction.

**Figure 22:**
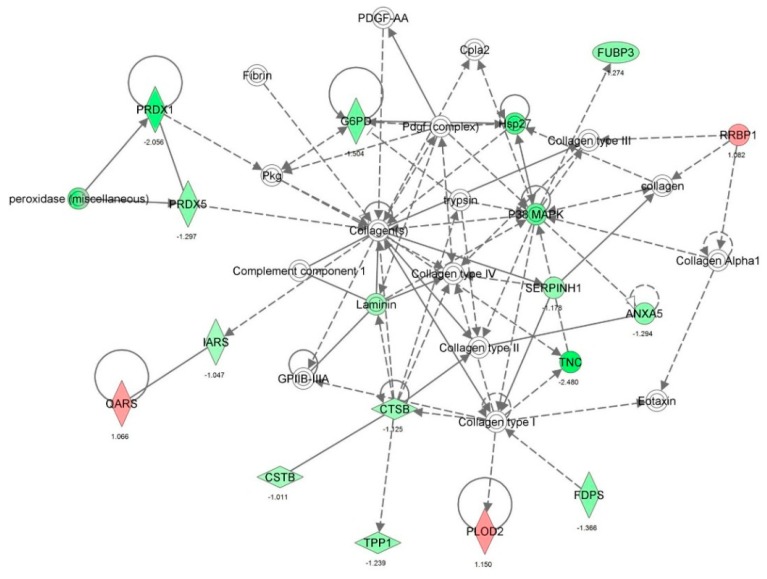
Vancomycin regulated cancer, endocrine system disorders, and organismal injury and abnormalities. Red indicates an up-regulation; green indicates a down-regulation. The intensity of green and redcolors indicates the degree of down- or up-regulation. Solid arrow indicates direct interaction and dashed arrow indicates indirect interaction.

**Figure 23:**
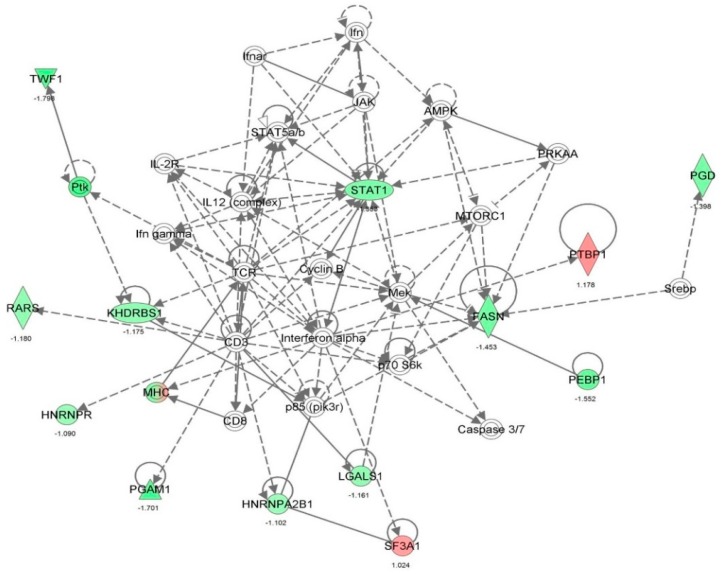
Vancomycin regulated RNA post-transcriptional modification, dermatological diseases and conditions, cell death and survival. Red indicates an up-regulation; green indicates a down-regulation. The intensity of green and redcolors indicates the degree of down- or up-regulation. Solid arrow indicates direct interaction and dashed arrow indicates indirect interaction.

**Figure 24:**
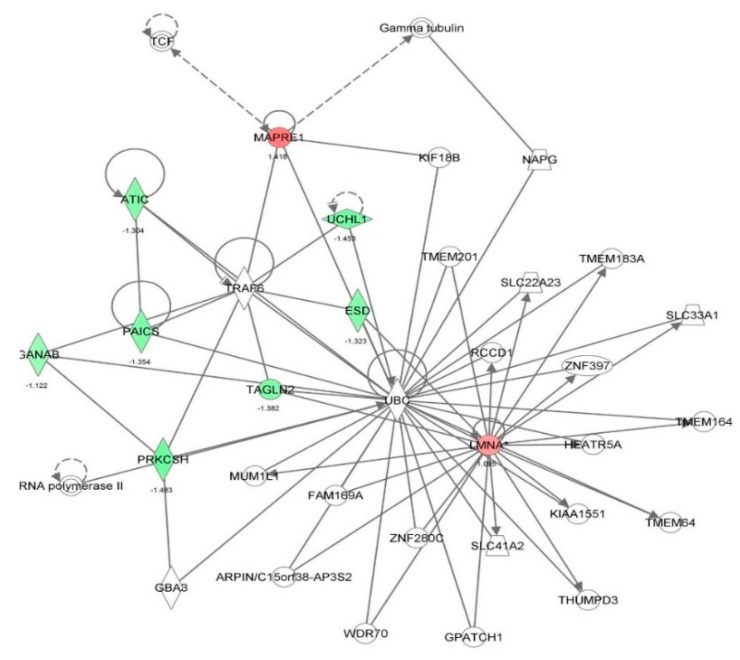
Vancomycin regulated carbohydrate metabolism, small molecule biochemistry, and cell morphology. Red indicates an up-regulation; green indicates a down-regulation. The intensity of green and red colors indicates the degree of down- or up-regulation. Solid arrow indicates direct interaction and dashed arrow indicates indirect interaction.

**Figure 25:**
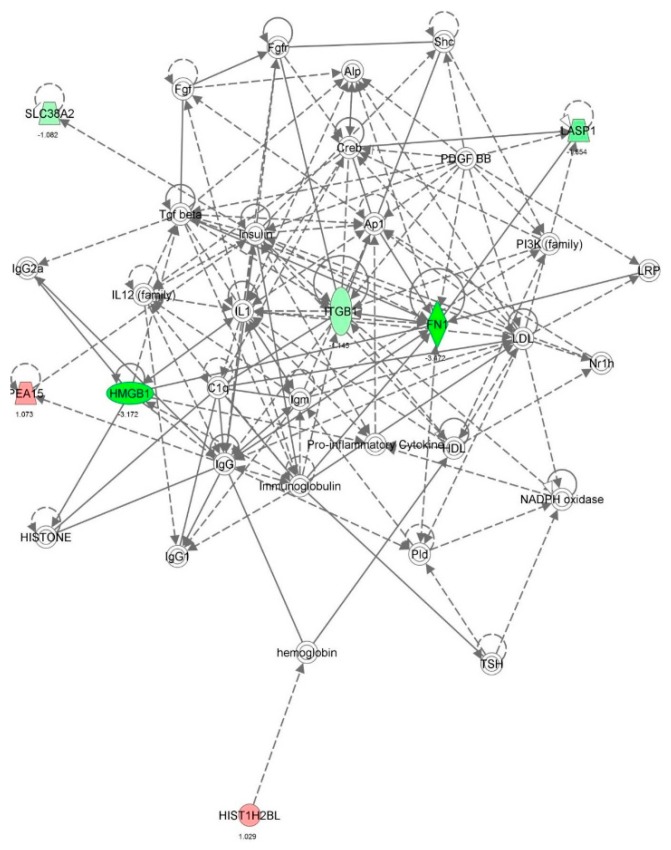
Vancomycin regulated cell-to-cell signaling and interaction, tissue development, and cardiovascular system development and function. Red indicates an up-regulation; green indicates a down-regulation. The intensity of green and red colors indicates the degree of down- or up-regulation. Solid arrow indicates direct interaction and dashed arrow indicates indirect interaction.

**Figure 26:**
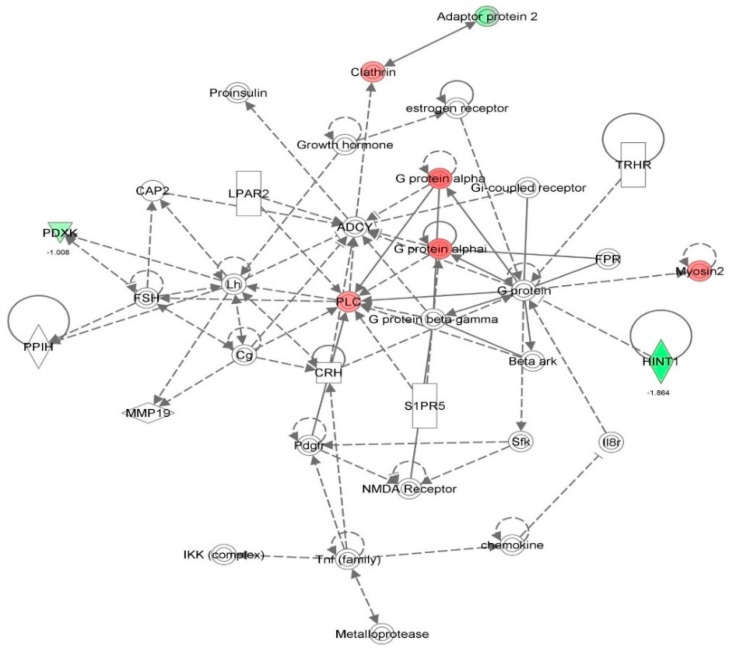
Vancomycin regulated connective tissue development and function, tissue morphology and behavior. Red indicates an up-regulation; green indicates a down-regulation. The intensity of green and red colors indicates the degree of down- or up-regulation. Solid arrow indicates direct interaction and dashed arrow indicates indirect interaction.

First, we performed a SILAC-based proteomic study to quantitatively determine the interactome of vancomycin in HK-2 cells treated with vancomycin at 50 μg/mL. There were 492 protein molecules identified as the potential targets of vancomycin in HK-2 cells (Table 1). These included a number of molecules involved in cell proliferation, cell metabolism, cell migration, cell invasion, cell survival, and cell death. Vancomycin increased the expression level of 178 protein molecules, but decreased the expression level of 314 protein molecules in HK-2 cells. Subsequently, these proteins were subject to IPA pathway analysis. A total of 486 molecular proteins were mapped using IPA (Table 2). Furthermore, as shown in and Table 3 and Figure 1 and Figure 2, there were 290 signaling pathways and cellular functions that were potentially regulated by vancomycin in HK-2 cells. Additionally, there were 24 networks of molecular signaling pathways that were regulated in HK-2 cells when treated with vancomycin (Table 4 and Figure 3, Figure 4, Figure 5, Figure 6, Figure 7, Figure 8, Figure 9, Figure 10, Figure 11, Figure 12, Figure 13, Figure 14, Figure 15, Figure 16, Figure 17, Figure 18, Figure 19, Figure 20, Figure 21, Figure 22, Figure 23, Figure 24, Figure 25 and Figure 26).

### 2.2. Vancomycin Alters a Number of Cellular Functions in HK-2 cells

Since we have identified numerous molecular targets which were regulated by vancomycin in HK-2 cells, we predicted the modulating effects of vancomycin on cellular functions in HK-2 cells by functional analysis using IPA software. As shown in Table 5, there were 500 categories of cellular functions which were altered by vancomycin in HK-2 cells. Treatment of vancomycin induced marked alterations in cell growth, cell proliferation, cell metabolism, cell morphology, gene expression, RNA post-translational modification, DNA replication and repair, cell-to-cell signaling and interaction, and cell death and survival. These cellular functional alterations are closely related to disease development. Notably, the functional analysis showed that vancomycin induced remarkable abnormalities in renal tissues and caused injury, probabaly resulting in renal tissue damage and cancer (Table 6), with the involvement of molecular proteins of AHNAK, ANLN, BASP1, EDC4, NUMA1, PDXK, PNP, SDHAF2, SMARCA4, SNRNP200, and TUBA1C. Moreover, vancomycin induced the inflammation, degradation, and fibrosis of renal tubules involving PSAP and APRT. Taken together, the functional analysis of vancomycin suggests that vancomycin has a damaging effect on cellular functions and thus cause renal injuries.

**Table 1:** 492 molecular proteins regulated by vancomycin in HK-2 cells.

Protein ID	Protein Name	Gene Names	Average(H/L)
P04264	Keratin, type II cytoskeletal 1	KRT1	0.172905
P35527	Keratin, type I cytoskeletal 9	KRT9	0.224193
E9PCX2	Aldose reductase	AKR1B1	0.272265
P02751	Fibronectin	FN1	0.288053
Q5T7C4	High mobility group protein B1	HMGB1; HMGB1P1	0.315275
Q13185	Chromobox protein homolog 3	CBX3	0.34326
P40261	Nicotinamide *N*-methyltransferase	NNMT	0.397895
P29966	Myristoylated alanine-rich C-kinase substrate	MARCKS	0.400377
F5H7V9	Tenascin	TNC	0.40316
Q9Y2V2	Calcium-regulated heat stable protein 1	CARHSP1	0.41335
K7ERT7	Synaptic vesicle membrane protein VAT-1 homolog	VAT1	0.434395
P51858	Hepatoma-derived growth factor	HDGF	0.453443
E9PEJ4	Dihydrolipoyllysine-residue acetyltransferase component of pyruvate dehydrogenase complex, mitochondrial	DLAT	0.4645
H0YK49	Electron transfer flavoprotein subunit α, mitochondrial	ETFA	0.46645
E7ES10	Calpastatin	CAST	0.46797
Q06830	Peroxiredoxin-1	PRDX1	0.486445
Q9HB71	Calcyclin-binding protein	CACYBP	0.49843
H0YJX6	Protein arginine *N*-methyltransferase 5	PRMT5	0.50622
O75937	DNAJ homolog subfamily C member 8	DNAJC8	0.50726
P49915	GMP synthase [glutamine-hydrolyzing]	GMPS	0.513415
H0YFA4	Cysteine-rich protein 2	CRIP2	0.51363
P04792	Heat shock protein β-1	HSPB1	0.51522
E9PLK3	Puromycin-sensitive aminopeptidase	NPEPPS	0.535463
P49773	Histidine triad nucleotide-binding protein 1	HINT1	0.5365
Q06323	Proteasome activator complex subunit 1	PSME1	0.541965
B8ZZQ6	Prothymosin α	PTMA	0.547615
Q9Y696	Chloride intracellular channel protein 4	CLIC4	0.55066
P16403	Histone H1.2	HIST1H1C; HIST1H1E; HIST1H1D; HIST1H1T; HIST1H1A	0.55312
H7C469	Cathepsin D	CTSD	0.554823
Q12792	Twinfilin-1	TWF1	0.55623
J3KTF8	Rho GDP-dissociation inhibitor 1	ARHGDIA	0.5657
P28482	Mitogen-activated protein kinase 1	MAPK1	0.57503
Q9C005	Protein DPY-30 homolog	DPY30	0.57574
Q9UKY7	Protein CDV3 homolog	CDV3	0.582217
B4E022	Transketolase	TKT	0.58253
Q99497	Protein DJ-1	PARK7	0.584365
P18669	Phosphoglycerate mutase 1	PGAM1	0.587947
P09104	γ-Enolase	ENO2	0.58879
P20042	Eukaryotic translation initiation factor 2 subunit 2	EIF2S2	0.60232
Q01995	Transgelin	TAGLN	0.60334
C9J9W2	LIM and SH3 domain protein 1	LASP1	0.60464
P37837	Transaldolase	TALDO1	0.60516
F8VZ29	Ubiquitin-conjugating enzyme E_2_ N	UBE2N	0.60613
Q9H4M9	EH domain-containing protein 1	EHD1	0.60702
Q96QK1	Vacuolar protein sorting-associated protein 35	VPS35	0.609495
P49321	Nuclear autoantigenic sperm protein	NASP	0.6098
P40925	Malate dehydrogenase, cytoplasmic	MDH1	0.61013
P31150	Rab GDP dissociation inhibitor α	GDI1	0.61355
Q16836	Hydroxyacyl-coenzyme A dehydrogenase, mitochondrial	HADH	0.61513
P60174	Triosephosphate isomerase	TPI1	0.617187
Q92616	Translational activator GCN1	GCN1L1	0.623255
Q99714	3-Hydroxyacyl-CoA dehydrogenase type-2	HSD17B10	0.630585
P31947	14-3-3 protein σ	SFN	0.630687
P12956	X-ray repair cross-complementing protein 6	XRCC6	0.63482
Q00341	Vigilin	HDLBP	0.63632
O75367	Core histone macro-H2A.1	H2AFY	0.63646
P07814	Bifunctional glutamate/proline-tRNA ligase	EPRS	0.6377
P27816	Microtubule-associated protein 4	MAP4	0.64062
P05141	ADP/ATP translocase 2	SLC25A5	0.641425
Q04917	14-3-3 protein η	YWHAH	0.641525
O43175	d-3-phosphoglycerate dehydrogenase	PHGDH	0.644225
P30086	Phosphatidylethanolamine-binding protein 1	PEBP1	0.644533
Q01082	Spectrin β chain, non-erythrocytic 1	SPTBN1	0.64709
Q13907	Isopentenyl-diphosphate δ-isomerase 1	IDI1	0.647705
P53621	Coatomer subunit α	COPA	0.65046
O60884	DNAJ homolog subfamily A member 2	DNAJA2	0.65208
C9JNR4	Transforming protein RhoA	RHOA	0.65726
P11413	Glucose-6-phosphate 1-dehydrogenase	G6PD	0.664673
P26196	Probable ATP-dependent RNA helicase DDX6	DDX6	0.66701
Q08945	FACT complex subunit SSRP1	SSRP1	0.66756
Q13283	Ras GTPase-activating protein-binding protein 1	G3BP1	0.66807
Q15365	Poly(rC)-binding protein 1	PCBP1	0.66997
P42166	Lamina-associated polypeptide 2, isoform α	TMPO	0.6721
K7ELL7	Glucosidase 2 subunit β	PRKCSH	0.67451
P32119	Peroxiredoxin-2	PRDX2	0.67591
P23381	Tryptophan-tRNA ligase, cytoplasmic	WARS	0.677045
P33991	DNA replication licensing factor MCM4	MCM4	0.6794
P05787	Keratin, type II cytoskeletal 8	KRT8	0.680803
C9JJ34	Ran-specific GTPase-activating protein	RANBP1	0.68427
P49327	Fatty acid synthase	FASN	0.687997
P09936	Ubiquitin carboxyl-terminal hydrolase isozyme L1	UCHL1	0.688333
A2A2D0	Stathmin	STMN1	0.69224
Q9ULV4	Coronin-1C	CORO1C	0.695523
Q9Y4L1	Hypoxia up-regulated protein 1	HYOU1	0.696023
P07195	l-lactate dehydrogenase B chain	LDHB	0.700453
F8VWS0	60S acidic ribosomal protein P0	RPLP0	0.703667
P25205	DNA replication licensing factor MCM3	MCM3	0.708945
K7EJE8	Lon protease homolog, mitochondrial	LONP1	0.70909
P23528	Cofilin-1	CFL1	0.71004
P07737	Profilin-1	PFN1	0.71163
E5RIW3	Tubulin-specific chaperone A	TBCA	0.71218
P63010	AP-2 complex subunit β	AP2B1	0.714287
P52209	6-Phosphogluconate dehydrogenase, decarboxylating	PGD	0.715343
O00151	PDZ and LIM domain protein 1	PDLIM1	0.71603
P36871	Phosphoglucomutase-1	PGM1	0.717643
Q15056	Eukaryotic translation initiation factor 4H	EIF4H	0.721007
P27797	Calreticulin	CALR	0.722223
P37802	Transgelin-2	TAGLN2	0.723653
Q16777	Histone H2A type 2-C	HIST2H2AC; HIST2H2AA3	0.72469
Q92973	Transportin-1	TNPO1	0.72551
O60506	Heterogeneous nuclear ribonucleoprotein Q	SYNCRIP	0.7259
O14579	Coatomer subunit ε	COPE	0.72719
P14324	Farnesyl pyrophosphate synthase	FDPS	0.7323
B4DQU5	Ras-related protein Rab-11A	RAB11A; RAB11B	0.73251
P00338	l-lactate dehydrogenase A chain	LDHA	0.735003
P24752	Acetyl-CoA acetyltransferase, mitochondrial	ACAT1	0.73511
O75369	Filamin-B	FLNB	0.73513
P14866	Heterogeneous nuclear ribonucleoprotein L	HNRNPL	0.735237
B4DDD8	Histidine—tRNA ligase, cytoplasmic	HARS	0.73643
E9PBS1	Multifunctional protein ADE2	PAICS	0.738453
P42224	Signal transducer and activator of transcription 1-α/β	STAT1	0.738843
P30084	Enoyl-CoA hydratase, mitochondrial	ECHS1	0.740183
B7Z972	Protein-l-isoaspartate *O*-methyltransferase	PCMT1	0.7404
P53618	Coatomer subunit β	COPB1	0.74088
Q04760	Lactoylglutathione lyase	GLO1	0.7413
D6RG15	Twinfilin-2	TWF2	0.74232
P40939	Trifunctional enzyme subunit α, mitochondrial	HADHA	0.744977
P14618	Pyruvate kinase PKM	PKM	0.745617
P07237	Protein disulfide-isomerase	P4HB	0.7458
P30447	HLA class I histocompatibility antigen, A-23 α chain	HLA-A; HLA-H; HLA-C	0.74708
Q10567	AP-1 complex subunit β-1	AP1B1	0.74868
P21796	Voltage-dependent anion-selective channel protein 1	VDAC1	0.748783
E9PMH2	Peptidyl-prolyl *cis*-*trans* isomerase	AIP	0.75192
F8VUA6	60S ribosomal protein L18	RPL18	0.75214
P06744	Glucose-6-phosphate isomerase	GPI	0.755337
O60888	Protein CutA	CUTA	0.7555
P35606	Coatomer subunit β	COPB2	0.755705
P10768	*S*-formylglutathione hydrolase	ESD	0.75572
P27695	DNA-(apurinic or apyrimidinic site) lyase	APEX1	0.75672
P12004	Proliferating cell nuclear antigen	PCNA	0.75721
Q6ZR64		MXRA7	0.75738
Q9UQ80	Proliferation-associated protein 2G4	PA2G4	0.75995
E7EUY0	DNA-dependent protein kinase catalytic subunit	PRKDC	0.761483
Q15185	Prostaglandin E synthase 3	PTGES3	0.762053
P43243	Matrin-3	MATR3	0.765323
Q96FW1	Ubiquitin thioesterase OTUB1	OTUB1	0.766887
P31939	Bifunctional purine biosynthesis protein PURH	ATIC	0.766895
Q99733	Nucleosome assembly protein 1-like 4	NAP1L4	0.769025
P62191	26S protease regulatory subunit 4	PSMC1	0.77016
P40926	Malate dehydrogenase, mitochondrial	MDH2	0.770383
Q32Q12	Nucleoside diphosphate kinase	NME1-NME2; NME2; NME2P1; NME1	0.770707
P30044	Peroxiredoxin-5, mitochondrial	PRDX5	0.77087
P34897	Serine hydroxymethyltransferase, mitochondrial	SHMT2	0.77093
P08758	Annexin A5	ANXA5	0.772893
P00558	Phosphoglycerate kinase 1	PGK1	0.776877
Q92945	Far upstream element-binding protein 2	KHSRP	0.778823
Q01105	Protein SET	SET	0.779857
F8W1N5	Nascent polypeptide-associated complex subunit α	NACA	0.78007
P05386	60S acidic ribosomal protein P1	RPLP1	0.784843
Q9BWD1	Acetyl-CoA acetyltransferase, cytosolic	ACAT2	0.7849
Q96I24	Far upstream element-binding protein 3	FUBP3	0.78507
F8VQE1	LIM domain and actin-binding protein 1	LIMA1	0.786983
O43852	Calumenin	CALU	0.787113
Q02790	Peptidyl-prolyl *cis*-*trans* isomerase FKBP4	FKBP4	0.790175
J3KN67	Tropomyosin α-3 chain	TPM3	0.790573
P00441	Superoxide dismutase [Cu-Zn]	SOD1	0.79129
Q15181	Inorganic pyrophosphatase	PPA1	0.79164
P20700	Lamin-B1	LMNB1	0.798267
E9PK47	Phosphorylase	PYGL	0.79884
P62136	Serine/threonine-protein phosphatase PP1-α catalytic subunit	PPP1CA; PPP1CC	0.79982
P43686	26S protease regulatory subunit 6B	PSMC4	0.800465
P07384	Calpain-1 catalytic subunit	CAPN1	0.806005
O14773	Tripeptidyl-peptidase 1	TPP1	0.807353
Q14566	DNA replication licensing factor MCM6	MCM6	0.80749
Q09666	Neuroblast differentiation-associated protein AHNAK	AHNAK	0.81098
P84077	ADP-ribosylation factor 1	ARF1; ARF3; ARF4; ARF5	0.81248
Q14444	Caprin-1	CAPRIN1	0.812877
F5H018	GTP-binding nuclear protein Ran	RAN	0.813607
Q92499	ATP-dependent RNA helicase DDX1	DDX1	0.813953
Q99832	T-complex protein 1 subunit η	CCT7	0.81562
P68431	Histone H3.1	HIST1H3A; H3F3B; H3F3A; HIST3H3; H3F3C	0.816783
P33993	DNA replication licensing factor MCM7	MCM7	0.81733
Q01518	Adenylyl cyclase-associated protein 1	CAP1	0.818003
P62258	14-3-3 protein ε	YWHAE	0.822137
P63241	Eukaryotic translation initiation factor 5A-1	EIF5A; EIF5AL1	0.82674
P26368	Splicing factor U2AF 65 kDa subunit	U2AF2	0.827353
P49588	Alanine-tRNA ligase, cytoplasmic	AARS	0.827427
P24534	Elongation factor 1-β	EEF1B2	0.82769
P68104	Elongation factor 1-α 1	EEF1A1; EEF1A1P5	0.828823
P39023	60S ribosomal protein L3	RPL3	0.829365
O75083	WD repeat-containing protein 1	WDR1	0.82946
H0YLC2	Proteasome subunit α type	PSMA4	0.83034
P46778	60S ribosomal protein L21	RPL21	0.83041
P40227	T-complex protein 1 subunit ζ	CCT6A	0.83204
Q14980	Nuclear mitotic apparatus protein 1	NUMA1	0.832107
Q13263	Transcription intermediary factor 1-β	TRIM28	0.832473
P06733	α-Enolase	ENO1	0.833683
Q9UK76	Hematological and neurological expressed 1 protein	HN1	0.833713
P13010	X-ray repair cross-complementing protein 5	XRCC5	0.835137
P67809	Nuclease-sensitive element-binding protein 1	YBX1	0.83629
Q13492	Phosphatidylinositol-binding clathrin assembly protein	PICALM	0.83656
P26639	Threonine-tRNA ligase, cytoplasmic	TARS	0.837765
P30153	Serine/threonine-protein phosphatase 2A 65 kDa regulatory subunit A α isoform	PPP2R1A	0.839417
P61978	Heterogeneous nuclear ribonucleoprotein K	HNRNPK	0.839853
G3V1A1	60S ribosomal protein L8	RPL8	0.83992
R4GN98	Protein S100-A6	S100A6	0.840685
P62081	40S ribosomal protein S7	RPS7	0.84122
P16989	Y-box-binding protein 3	YBX3	0.84325
P67936	Tropomyosin α-4 chain	TPM4	0.84394
P10809	60 kDa heat shock protein, mitochondrial	HSPD1	0.84574
E9PLD0	Ras-related protein Rab-1B	RAB1B; RAB1C	0.84636
P54136	Arginine-tRNA ligase, cytoplasmic	RARS	0.84759
P50454	Serpin H1	SERPINH1	0.848623
P11021	78 kDa glucose-regulated protein	HSPA5	0.84892
P61769	β2-microglobulin	B2M	0.849333
F5GY37	Prohibitin-2	PHB2	0.85107
Q07666	KH domain-containing, RNA-binding, signal transduction-associated protein 1	KHDRBS1	0.85112
P18206	Vinculin	VCL	0.851217
P62314	Small nuclear ribonucleoprotein Sm D1	SNRPD1	0.85213
P49411	Elongation factor Tu, mitochondrial	TUFM	0.85245
H0Y4R1	Inosine-5-monophosphate dehydrogenase 2	IMPDH2	0.853715
P04844	Dolichyl-diphosphooligosaccharide-protein glycosyltransferase subunit 2	RPN2	0.85403
Q71DI3	Histone H3.2	HIST2H3A	0.854437
P50395	Rab GDP dissociation inhibitor β	GDI2	0.855377
Q00839	Heterogeneous nuclear ribonucleoprotein U	HNRNPU	0.8554
Q13347	Eukaryotic translation initiation factor 3 subunit I	EIF3I	0.85975
P40222	α-Taxilin	TXLNA	0.860857
P09382	Galectin-1	LGALS1	0.861283
Q15084	Protein disulfide-isomerase A6	PDIA6	0.861437
Q14247	Src substrate cortactin	CTTN	0.86161
P21333	Filamin-A	FLNA	0.86397
O14818	Proteasome subunit α type-7	PSMA7; PSMA8	0.86409
P13667	Protein disulfide-isomerase A4	PDIA4	0.86544
Q9UMS4	Pre-mRNA-processing factor 19	PRPF19	0.866023
E7EPW6	Programmed cell death protein 6	PDCD6	0.87172
E9PIR7	Thioredoxin reductase 1, cytoplasmic	TXNRD1	0.872513
Q9UHX1	Poly(U)-binding-splicing factor PUF60	PUF60	0.872915
P05556	Integrin β-1	ITGB1	0.873165
P40121	Macrophage-capping protein	CAPG	0.87371
Q03135	Caveolin-1	CAV1	0.87472
P13797	Plastin-3	PLS3	0.87507
P27824	Calnexin	CANX	0.87692
Q15019	Septin-2	42249	0.87721
Q9UNZ2	NSFL1 cofactor p47	NSFL1C	0.878297
Q9Y5B9	FACT complex subunit SPT16	SUPT16H	0.879483
B1AK85	F-actin-capping protein subunit β	CAPZB	0.88193
P68133	Actin, α skeletal muscle	ACTA1; ACTC1; ACTG2; ACTA2	0.88416
P06493	Cyclin-dependent kinase 1	CDK1	0.88483
Q3ZCM7	Tubulin β-8 chain	TUBB8	0.88649
P07858	Cathepsin B	CTSB	0.889247
Q16555	Dihydropyrimidinase-related protein 2	DPYSL2	0.88961
P63104	14-3-3 protein ζ/δ	YWHAZ	0.890777
Q14697	Neutral α-glucosidase AB	GANAB	0.89087
P05783	Keratin, type I cytoskeletal 18	KRT18	0.891143
P26640	Valine-tRNA ligase	VARS	0.89307
B4DUR8	T-complex protein 1 subunit γ	CCT3	0.895913
Q96P70	Importin-9	IPO9	0.89849
Q7KZF4	Staphylococcal nuclease domain-containing protein 1	SND1	0.899653
Q16643	Drebrin	DBN1	0.90207
P35579	Myosin-9	MYH9	0.90543
P22626	Heterogeneous nuclear ribonucleoproteins A2/B1	HNRNPA2B1	0.907457
Q15942	Zyxin	ZYX	0.909143
Q9NQC3	Reticulon-4	RTN4	0.90961
P07108	Acyl-CoA-binding protein	DBI	0.91033
P11216	Glycogen phosphorylase, brain form	PYGB	0.914587
A6PVH9	Copine-1	CPNE1	0.914685
O14980	Exportin-1	XPO1	0.91646
P04075	Fructose-bisphosphate aldolase A	ALDOA	0.917223
O43390	Heterogeneous nuclear ribonucleoprotein R	HNRNPR	0.91729
P61158	Actin-related protein 3	ACTR3	0.91757
P13639	Elongation factor 2	EEF2	0.918273
P07942	Laminin subunit β-1	LAMB1	0.91949
P31948	Stress-induced-phosphoprotein 1	STIP1	0.920767
Q14204	Cytoplasmic dynein 1 heavy chain 1	DYNC1H1	0.92284
O60664	Perilipin-3	PLIN3	0.922903
P62158	Calmodulin	CALM1; CALM2; CALM3	0.923537
Q96QD8	Sodium-coupled neutral amino acid transporter 2	SLC38A2	0.92438
Q92575	UBX domain-containing protein 4	UBXN4	0.924495
M0R0F0	40S ribosomal protein S5	RPS5	0.925887
P08133	Annexin A6	ANXA6	0.92615
P62906	60S ribosomal protein L10a	RPL10A	0.930083
O15144	Actin-related protein 2/3 complex subunit 2	ARPC2	0.932825
Q9Y490	Talin-1	TLN1	0.933233
P04406	Glyceraldehyde-3-phosphate dehydrogenase	GAPDH	0.934323
P25786	Proteasome subunit α type-1	PSMA1	0.93491
P25787	Proteasome subunit α type-2	PSMA2	0.93574
P34932	Heat shock 70 kDa protein 4	HSPA4	0.935993
E7EQR4	Ezrin	EZR	0.93788
P46777	60S ribosomal protein L5	RPL5	0.939327
P05387	60S acidic ribosomal protein P2	RPLP2	0.94137
P25398	40S ribosomal protein S12	RPS12	0.941813
P42704	Leucine-rich PPR motif-containing protein, mitochondrial	LRPPRC	0.944687
P55072	Transitional endoplasmic reticulum ATPase	VCP	0.945267
P62937	Peptidyl-prolyl *cis*-*tran*s isomerase A	PPIA	0.94628
P68363	Tubulin α-1B chain	TUBA1B	0.94723
Q01628	Interferon-induced transmembrane protein 3	IFITM3	0.950665
O00299	Chloride intracellular channel protein 1	CLIC1	0.952683
P30040	Endoplasmic reticulum resident protein 29	ERP29	0.954027
Q8N8S7	Protein enabled homolog	ENAH	0.954545
P41252	Isoleucine-tRNA ligase, cytoplasmic	IARS	0.95526
P61981	14-3-3 protein γ	YWHAG	0.9565
F8VPF3	Myosin light polypeptide 6	MYL6	0.95902
O00571	ATP-dependent RNA helicase DDX3X	DDX3X; DDX3Y	0.959895
P45880	Voltage-dependent anion-selective channel protein 2	VDAC2	0.960875
P19338	Nucleolin	NCL	0.963977
P14625	Endoplasmin	HSP90B1	0.96555
O43707	α-Actinin-4	ACTN4	0.968487
Q5JP53	Tubulin β chain	TUBB	0.971943
Q13409	Cytoplasmic dynein 1 intermediate chain 2	DYNC1I2	0.976747
P55060	Exportin-2	CSE1L	0.978383
Q13838	Spliceosome RNA helicase DDX39B	DDX39B	0.979103
P02786	Transferrin receptor protein 1	TFRC	0.982483
P00387	NADH-cytochrome *b*_5_ reductase 3	CYB5R3	0.98267
O75947	ATP synthase subunit d, mitochondrial	ATP5H	0.985415
G8JLD5	Dynamin-1-like protein	DNM1L	0.98906
P04080	Cystatin-B	CSTB	0.98954
P05198	Eukaryotic translation initiation factor 2 subunit 1	EIF2S1	0.98961
Q9UL46	Proteasome activator complex subunit 2	PSME2	0.990545
P55884	Eukaryotic translation initiation factor 3 subunit B	EIF3B	0.991517
P46783	40S ribosomal protein S10	RPS10; RPS10P5	0.99156
F2Z2Y4	Pyridoxal kinase	PDXK	0.99206
P09211	Glutathione *S*-transferase P	GSTP1	0.992743
P38646	Stress-70 protein, mitochondrial	HSPA9	0.995527
P80723	Brain acid soluble protein 1	BASP1	0.998527
O15143	Actin-related protein 2/3 complex subunit 1B	ARPC1B	1.003
Q8NC51	Plasminogen activator inhibitor 1 RNA-binding protein	SERBP1	1.003403
P54727	UV excision repair protein RAD23 homolog B	RAD23B	1.005245
P51991	Heterogeneous nuclear ribonucleoprotein A3	HNRNPA3	1.006415
P07355	Annexin A2	ANXA2; ANXA2P2	1.007367
P23246	Splicing factor, proline- and glutamine-rich	SFPQ	1.009463
P08107	Heat shock 70 kDa protein 1A/1B	HSPA1A	1.01165
P67775	Serine/threonine-protein phosphatase 2A catalytic subunit α isoform	PPP2CA; PPP2CB	1.011925
P21291	Cysteine and glycine-rich protein 1	CSRP1	1.01607
P28066	Proteasome subunit α type-5	PSMA5	1.01847
P68036	Ubiquitin-conjugating enzyme E2 L3	UBE2L3	1.02085
Q9BUF5	Tubulin β-6 chain	TUBB6	1.021827
Q15459	Splicing factor 3A subunit 1	SF3A1	1.02408
Q9P0L0	Vesicle-associated membrane protein-associated protein A	VAPA	1.026153
C9JD32	60S ribosomal protein L23	RPL23	1.026225
Q15293	Reticulocalbin-1	RCN1	1.02665
P55735	Protein SEC13 homolog	SEC13	1.0291
Q99880	Histone H2B type 1-l	HIST1H2BL; HIST1H2BM; HIST1H2BN; HIST1H2BH; HIST2H2BF; HIST1H2BC; HIST1H2BD; H2BFS; HIST1H2BK	1.02927
D6RFM5	Succinate dehydrogenase [ubiquinone] flavoprotein subunit, mitochondrial	SDHA	1.034553
P46821	Microtubule-associated protein 1B	MAP1B	1.03558
Q5W0X3	Peptidyl-prolyl *cis*-*trans* isomerase	FKBP1A	1.0359
K7EK07	Histone H3	H3F3B; H3F3A	1.037015
P52272	Heterogeneous nuclear ribonucleoprotein M	HNRNPM	1.03914
P05023	Sodium/potassium-transporting ATPase subunit α-1	ATP1A1; ATP1A3	1.043157
P36578	60*S* ribosomal protein L4	RPL4	1.044293
P08238	Heat shock protein HSP 90-β	HSP90AB1	1.044997
P68371	Tubulin β-4B chain	TUBB4B	1.045547
Q01081	Splicing factor U2AF 35 kDa subunit	U2AF1; U2AF1L4	1.0472
P35637	RNA-binding protein FUS	FUS	1.049003
P56192	Methionine-tRNA ligase, cytoplasmic	MARS	1.0493
P06576	ATP synthase subunit β, mitochondrial	ATP5B	1.049537
P04083	Annexin A1	ANXA1	1.0517
P41250	Glycine-tRNA ligase	GARS	1.05216
Q13813	Spectrin αα chain, non-erythrocytic 1	SPTAN1	1.0563
Q9Y678	Coatomer subunit γ-1	COPG1	1.0569
M0QZS6	SUMO-activating enzyme subunit 1	SAE1	1.0578
Q9UHD8	Septin-9	42256	1.063053
P31946	14-3-3 protein β/α	YWHAB	1.063833
P63261	Actin, cytoplasmic 2	ACTG1	1.063967
P13489	Ribonuclease inhibitor	RNH1	1.064393
P47897	Glutamine-tRNA ligase	QARS	1.0658
B4DJV2	Citrate synthase	CS	1.0662
P04843	Dolichyl-diphosphooligosaccharide-protein glycosyltransferase subunit 1	RPN1	1.066757
Q14974	Importin subunit β-1	KPNB1	1.072667
Q15121	Astrocytic phosphoprotein PEA-15	PEA15	1.072933
Q9NYL9	Tropomodulin-3	TMOD3	1.0755
O00410	Importin-5	IPO5	1.0767
P26038	Moesin	MSN	1.078713
Q9P2E9	Ribosome-binding protein 1	RRBP1	1.081993
P68402	Platelet-activating factor acetylhydrolase IB subunit β	PAFAH1B2	1.0887
H0YEN5	40S ribosomal protein S2	RPS2	1.091785
Q5VU59		TPM3	1.0933
P02545	Prelamin-A/C	LMNA	1.095077
P35221	Catenin α-1	CTNNA1	1.09635
Q08211	ATP-dependent RNA helicase A	DHX9	1.096937
P50991	T-complex protein 1 subunit δ	CCT4	1.103
O60493	Sorting nexin-3	SNX3	1.1169
P61160	Actin-related protein 2	ACTR2	1.1175
O95433	Activator of 90 kDa heat shock protein ATPase homolog 1	AHSA1	1.1199
P62805	Histone H4	HIST1H4A	1.1241
P18465	HLA class I histocompatibility antigen, B-57 α chain	HLA-B	1.12765
Q8TAT6	Nuclear protein localization protein 4 homolog	NPLOC4	1.135
P46940	Ras GTPase-activating-like protein IQGAP1	IQGAP1	1.135853
P09972	Fructose-bisphosphate aldolase C	ALDOC	1.1406
P62424	60S ribosomal protein L7a	RPL7A	1.14125
Q00610	Clathrin heavy chain 1	CLTC	1.142133
O15355	Protein phosphatase 1G	PPM1G	1.1437
Q16222	UDP-N-acetylhexosamine pyrophosphorylase	UAP1	1.145295
P25705	ATP synthase subunit α, mitochondrial	ATP5A1	1.14579
E7ETU9	Procollagen-lysine, 2-oxoglutarate 5-dioxygenase 2	PLOD2	1.150217
K7EJ78	40S ribosomal protein S15	RPS15	1.1525
P52292	Importin subunit α-1	KPNA2	1.153933
Q15417	Calponin-3	CNN3	1.154837
E7ETK0	40S ribosomal protein S24	RPS24	1.1574
P51149	Ras-related protein Rab-7a	RAB7A	1.158243
Q9NTK5	OBG-like ATPase 1	OLA1	1.1594
Q5URX0	β-Hexosaminidase subunit β	HEXB	1.159533
P12814	α-Actinin-1	ACTN1	1.165233
F8VVM2	Phosphate carrier protein, mitochondrial	SLC25A3	1.165257
Q9Y224	UPF0568 protein C14orf166	C14orf166	1.1655
P06748	Nucleophosmin	NPM1	1.1659
P30101	Protein disulfide-isomerase A3	PDIA3	1.167067
P26641	Elongation factor 1-γ	EEF1G	1.1681
P78371	T-complex protein 1 subunit β	CCT2	1.169767
F8VY35	Nucleosome assembly protein 1-like 1	NAP1L1	1.17258
P56537	Eukaryotic translation initiation factor 6	EIF6	1.172633
E7EV56	Pericentriolar material 1 protein	PCM1	1.1749
P26599	Polypyrimidine tract-binding protein 1	PTBP1	1.1785
P27348	14-3-3 protein θ	YWHAQ	1.180407
P22314	Ubiquitin-like modifier-activating enzyme 1	UBA1	1.182133
D6RG13	40S ribosomal protein S3a	RPS3A	1.184367
P00367	Glutamate dehydrogenase 1, mitochondrial	GLUD1; GLUD2	1.1844
E9PNW4	CD59 glycoprotein	CD59	1.1867
P11717	Cation-independent mannose-6-phosphate receptor	IGF2R	1.1869
P17987	T-complex protein 1 subunit α	TCP1	1.1904
P60842	Eukaryotic initiation factor 4A–I	EIF4A1	1.191733
P04632	Calpain small subunit 1	CAPNS1	1.19265
P07900	Heat shock protein HSP 90-α	HSP90AA1	1.195633
P20618	Proteasome subunit β type-1	PSMB1	1.2078
J3KPE3	Guanine nucleotide-binding protein subunit β-2-like 1	GNB2L1	1.209467
P35613	Basigin	BSG	1.21668
P31930	Cytochrome b-c1 complex subunit 1, mitochondrial	UQCRC1	1.217533
P31942	Heterogeneous nuclear ribonucleoprotein H3	HNRNPH3	1.223433
E9PCY7	Heterogeneous nuclear ribonucleoprotein H	HNRNPH1	1.2236
F8W7C6		RPL10	1.225633
Q07021	Complement component 1 Q subcomponent-binding protein, mitochondrial	C1QBP	1.2273
Q96AG4	Leucine-rich repeat-containing protein 59	LRRC59	1.227793
Q14315	Filamin-C	FLNC	1.2332
P48643	T-complex protein 1 subunit ε	CCT5	1.234767
E7EQV3	Polyadenylate-binding protein 1	PABPC1; PABPC4	1.235133
P62701	40S ribosomal protein S4, X isoform	RPS4X	1.2362
P29692	Elongation factor 1-δ	EEF1D	1.239133
P53396	ATP-citrate synthase	ACLY	1.241963
Q12906	Interleukin enhancer-binding factor 3	ILF3	1.245833
P23396	40S ribosomal protein S3	RPS3	1.2509
P50990	T-complex protein 1 subunit θ	CCT8	1.257
P11142	Heat shock cognate 71 kDa protein	HSPA8	1.258433
Q12905	Interleukin enhancer-binding factor 2	ILF2	1.2642
H0Y3Y4	Septin-7	42254	1.2656
F8VZX2	Poly(rC)-binding protein 2	PCBP2	1.265963
C9J9K3	40S ribosomal protein SA	RPSA; RPSAP58	1.2668
P35580	Myosin-10	MYH10	1.275013
P23526	Adenosylhomocysteinase	AHCY	1.276
P30050	60S ribosomal protein L12	RPL12	1.287833
H3BT13	Small nuclear ribonucleoprotein Sm D3	SNRPD3	1.288
I7HJJ0	ADP/ATP translocase 3	SLC25A6; SLC25A4	1.30365
E7EMC7	Sequestosome-1	SQSTM1	1.3099
P52907	F-actin-capping protein subunit α-1	CAPZA1	1.3127
Q01813	6-Phosphofructokinase type C	PFKP	1.312933
Q02878	60S ribosomal protein L6	RPL6	1.3141
P19105	Myosin regulatory light chain 12A	MYL12A; MYL12B; MYL9	1.315967
Q14019	Coactosin-like protein	COTL1	1.318367
Q5TCI8		LMNA	1.318467
P20290	Transcription factor BTF3	BTF3	1.324333
Q9UJZ1	Stomatin-like protein 2, mitochondrial	STOML2	1.3251
Q15149	Plectin	PLEC	1.325767
Q5T8U5	Surfeit locus protein 4	SURF4	1.3268
F8W6I7	Heterogeneous nuclear ribonucleoprotein A1	HNRNPA1; HNRNPA1L2	1.329333
J3QSX4	Mitotic checkpoint protein BUB3	BUB3	1.3315
P33176	Kinesin-1 heavy chain	KIF5B	1.3317
P62280	40S ribosomal protein S11	RPS11	1.33505
P17812	CTP synthase 1	CTPS1	1.3383
P35268	60S ribosomal protein L22	RPL22	1.338767
Q13200	26S proteasome non-ATPase regulatory subunit 2	PSMD2	1.33905
P18124	60S ribosomal protein L7	RPL7	1.340267
Q13162	Peroxiredoxin-4	PRDX4	1.3457
O95373	Importin-7	IPO7	1.355167
O75533	Splicing factor 3B subunit 1	SF3B1	1.35925
O00622	Protein CYR61	CYR61	1.36705
P61353	60S ribosomal protein L27	RPL27	1.373033
Q15233	Non-POU domain-containing octamer-binding protein	NONO	1.3738
Q99613	Eukaryotic translation initiation factor 3 subunit C	EIF3C; EIF3CL	1.37415
P08574	Cytochrome *c*_1_, heme protein, mitochondrial	CYC1	1.37425
P98179	Putative RNA-binding protein 3	RBM3	1.37635
P17655	Calpain-2 catalytic subunit	CAPN2	1.386833
Q86UY0	Thioredoxin domain-containing protein 5	TXNDC5	1.390233
P62979	Ubiquitin-40S ribosomal protein S27a	RPS27A; UBB; UBC; UBA52; UBBP4	1.399267
Q86VP6	Cullin-associated NEDD8-dissociated protein 1	CAND1	1.4086
Q15691	Microtubule-associated protein RP/EB family member 1	MAPRE1	1.4179
Q7L2H7	Eukaryotic translation initiation factor 3 subunit M	EIF3M	1.45085
E7EX73	Eukaryotic translation initiation factor 4 γ 1	EIF4G1	1.4581
X1WI28	60S ribosomal protein L10	RPL10	1.4608
P17844	Probable ATP-dependent RNA helicase DDX5	DDX5	1.470067
P04899	Guanine nucleotide-binding protein G_i_ subunit α-2	GNAI2	1.5439
Q16891	Mitochondrial inner membrane protein	IMMT	1.5646
P08670	Vimentin	VIM	1.578767
P62241	40S ribosomal protein S8	RPS8	1.5809
Q14764	Major vault protein	MVP	1.63639
P31689	DNAJ homolog subfamily A member 1	DNAJA1	1.6699
Q9H299	SH3 domain-binding glutamic acid-rich-like protein 3	SH3BGRL3	1.68785
Q07065	Cytoskeleton-associated protein 4	CKAP4	1.728867
Q86V81	THO complex subunit 4	ALYREF	1.7965
Q5TCU3	Tropomyosin β chain	TPM2	1.8451
O00505	Importin subunit α-4	KPNA3	2.1267
Q6NZI2	Polymerase I and transcript release factor	PTRF	2.1653
H0YD13	CD44 antigen	CD44	2.2206
Q9BSJ8	Extended synaptotagmin-1	ESYT1	2.36957
P10619	Lysosomal protective protein	CTSA	3.0967

**Table 2:** 486 mapped molecular proteins by IPA.

ID	Symbol	Entrez Gene Name	Location	Type(s)	Fold Change
P49588	AARS	Alanyl-tRNA synthetase	Cytoplasm	Enzyme	−1.209
P24752	ACAT1	Acetyl-CoA acetyltransferase 1	Cytoplasm	Enzyme	−1.360
Q9BWD1	ACAT2	Acetyl-CoA acetyltransferase 2	Cytoplasm	Enzyme	−1.274
P53396	ACLY	ATP citrate lyase	Cytoplasm	Enzyme	1.242
P68133	ACTA1	Actin, α 1, skeletal muscle	Cytoplasm	Other	−1.131
P63261	ACTG1	Actin, γ 1	Cytoplasm	Other	1.064
P12814	ACTN1	Actinin, α 1	Cytoplasm	Other	1.165
O43707	ACTN4	Actinin, α 4	Cytoplasm	Other	−1.033
P61160	ACTR2	ARP2 actin-related protein 2 homolog (yeast)	Plasma Membrane	Other	1.118
P61158	ACTR3	ARP3 actin-related protein 3 homolog (yeast)	Plasma Membrane	Other	−1.090
P23526	AHCY	Adenosylhomocysteinase	Cytoplasm	Enzyme	1.276
Q09666	AHNAK	AHNAK nucleoprotein	Nucleus	Other	−1.233
O95433	AHSA1	AHA1, activator of heat shock 90 kDa protein ATPase homolog 1 (yeast)	Cytoplasm	Other	1.120
E9PMH2	AIP	Aryl hydrocarbon receptor interacting protein	Nucleus	Transcription regulator	−1.330
E9PCX2	AKR1B1	Aldo-keto reductase family 1, member B1 (aldose reductase)	Cytoplasm	Enzyme	−3.673
P04075	ALDOA	Aldolase A, fructose-bisphosphate	Cytoplasm	Enzyme	−1.090
P09972	ALDOC	Aldolase C, fructose-bisphosphate	Cytoplasm	Enzyme	1.141
Q86V81	ALYREF	Aly/REF export factor	Nucleus	Transcription regulator	1.796
P04083	ANXA1	Annexin A1	Plasma Membrane	Enzyme	1.052
P07355	ANXA2	Annexin A2	Plasma Membrane	Other	1.007
P08758	ANXA5	Annexin A5	Plasma Membrane	Other	−1.294
P08133	ANXA6	Annexin A6	Plasma Membrane	Ion channel	−1.080
Q10567	AP1B1	Adaptor-related protein complex 1, β 1 subunit	Cytoplasm	Transporter	−1.336
P63010	AP2B1	Adaptor-related protein complex 2, β 1 subunit	Plasma Membrane	Transporter	−1.400
P27695	APEX1	APEX nuclease (multifunctional DNA repair enzyme) 1	Nucleus	Enzyme	−1.321
P84077	ARF1	ADP-ribosylation factor 1	Cytoplasm	Enzyme	−1.231
J3KTF8	ARHGDIA	Rho GDP dissociation inhibitor (GDI) α	Cytoplasm	Other	−1.768
O15143	ARPC1B	Actin related protein 2/3 complex, subunit 1B, 41 kDa	Cytoplasm	Other	1.003
O15144	ARPC2	Actin related protein 2/3 complex, subunit 2, 34 kDa	Cytoplasm	Other	−1.072
P31939	ATIC	5-Aminoimidazole-4-carboxamide ribonucleotide formyltransferase/IMP cyclohydrolase	Cytoplasm	Enzyme	−1.304
P05023	ATP1A1	ATPase, Na^+^/K^+^ transporting, α1 polypeptide	Plasma Membrane	Transporter	1.043
P25705	ATP5A1	ATP synthase, H^+^ transporting, mitochondrial F1 complex, α subunit 1, cardiac muscle	Cytoplasm	Transporter	1.146
P06576	ATP5B	ATP synthase, H^+^ transporting, mitochondrial F1 complex, β polypeptide	Cytoplasm	Transporter	1.050
O75947	ATP5H	ATP synthase, H^+^ transporting, mitochondrial Fo complex, subunit d	Cytoplasm	Enzyme	−1.015
P61769	B2M	β2-Microglobulin	Plasma Membrane	Transmembrane receptor	−1.177
P80723	BASP1	Brain abundant, membrane attached signal protein 1	Nucleus	Transcription regulator	−1.001
P35613	BSG	Basigin (Ok blood group)	Plasma Membrane	Transporter	1.217
P20290	BTF3	Basic transcription factor 3	Nucleus	Transcription regulator	1.324
J3QSX4	BUB3	BUB3 mitotic checkpoint protein	Nucleus	Other	1.332
Q9Y224	C14orf166	Chromosome 14 open reading frame 166	Nucleus	Other	1.166
Q07021	C1QBP	Complement component 1, q subcomponent binding protein	Cytoplasm	Transcription regulator	1.227
Q9HB71	CACYBP	Calcyclin binding protein	Nucleus	Other	−2.006
P27797	CALR	Calreticulin	Cytoplasm	Transcription regulator	−1.385
O43852	CALU	Calumenin	Cytoplasm	Other	−1.270
Q86VP6	CAND1	Cullin-associated and neddylation-dissociated 1	Cytoplasm	Transcription regulator	1.409
P27824	CANX	calnexin	Cytoplasm	Other	−1.140
Q01518	CAP1	CAP, adenylate cyclase-associated protein 1 (yeast)	Plasma Membrane	Other	−1.222
P40121	CAPG	Capping protein (actin filament), gelsolin-like	Nucleus	Other	−1.145
P07384	CAPN1	Calpain 1, (mu/I) large subunit	Cytoplasm	Peptidase	−1.241
P17655	CAPN2	Calpain 2, (m/II) large subunit	Cytoplasm	Peptidase	1.387
P04632	CAPNS1	Calpain, small subunit 1	Cytoplasm	Peptidase	1.193
Q14444	CAPRIN1	Cell cycle associated protein 1	Plasma Membrane	Other	−1.230
P52907	CAPZA1	Capping protein (actin filament) muscle Z-line, α 1	Cytoplasm	Other	1.313
B1AK85	CAPZB	Capping protein (actin filament) muscle Z-line, β	Cytoplasm	Other	−1.134
Q9Y2V2	CARHSP1	Calcium regulated heat stable protein 1, 24 kDa	Cytoplasm	Other	−2.419
E7ES10	CAST	Calpastatin	Cytoplasm	Peptidase	−2.137
Q03135	CAV1	Caveolin 1, caveolae protein, 22 kDa	Plasma Membrane	Transmembrane receptor	−1.143
Q13185	CBX3	Chromobox homolog 3	Nucleus	Transcription regulator	−2.913
P78371	CCT2	Chaperonin containing TCP1, subunit 2 (β)	Cytoplasm	Kinase	1.170
B4DUR8	CCT3	Chaperonin containing TCP1, subunit 3 (γ)	Cytoplasm	Other	−1.116
P50991	CCT4	Chaperonin containing TCP1, subunit 4 (δ)	Cytoplasm	Other	1.103
P48643	CCT5	Chaperonin containing TCP1, subunit 5 (ε)	Cytoplasm	Other	1.235
P40227	CCT6A	Chaperonin containing TCP1, subunit 6A (ζ 1)	Cytoplasm	Other	−1.202
Q99832	CCT7	Chaperonin containing TCP1, subunit 7 (η)	Cytoplasm	Other	−1.226
P50990	CCT8	Chaperonin containing TCP1, subunit 8 (θ)	Cytoplasm	Enzyme	1.257
H0YD13	CD44	CD44 molecule (Indian blood group)	Plasma Membrane	Enzyme	2.221
E9PNW4	CD59	CD59 molecule, complement regulatory protein	Plasma Membrane	Other	1.187
P06493	CDK1	Cyclin-dependent kinase 1	Nucleus	Kinase	−1.130
Q9UKY7	CDV3	CDV3 homolog (mouse)	Cytoplasm	Other	−1.718
P23528	CFL1	Cofilin 1 (non-muscle)	Nucleus	Other	−1.408
Q07065	CKAP4	Cytoskeleton-associated protein 4	Cytoplasm	Other	1.729
O00299	CLIC1	Chloride intracellular channel 1	Nucleus	Ion channel	-1.050
Q9Y696	CLIC4	Chloride intracellular channel 4	Plasma Membrane	Ion channel	−1.816
Q00610	CLTC	Clathrin, heavy chain (Hc)	Plasma Membrane	Other	1.142
Q15417	CNN3	Calponin 3, acidic	Cytoplasm	Other	1.155
P53621	COPA	Coatomer protein complex, subunit α	Cytoplasm	Transporter	−1.537
P53618	COPB1	Coatomer protein complex, subunit β 1	Cytoplasm	Transporter	−1.350
P35606	COPB2	Coatomer protein complex, subunit β 2 (β prime)	Cytoplasm	Transporter	−1.323
O14579	COPE	Coatomer protein complex, subunit ε	Cytoplasm	Transporter	−1.375
Q9Y678	COPG1	Coatomer protein complex, subunit γ 1	Cytoplasm	Transporter	1.057
Q9ULV4	CORO1C	Coronin, actin binding protein, 1C	Cytoplasm	Other	−1.438
Q14019	COTL1	Coactosin-like F-actin binding protein 1	Cytoplasm	Other	1.318
A6PVH9	CPNE1	Copine I	Nucleus	Transporter	−1.093
H0YFA4	CRIP2	Cysteine-rich protein 2	Other	Other	−1.947
B4DJV2	CS	Citrate synthase	Cytoplasm	Enzyme	1.066
P55060	CSE1L	CSE1 chromosome segregation 1-like (yeast)	Nucleus	Transporter	−1.022
P21291	CSRP1	Cysteine and glycine-rich protein 1	Nucleus	Other	1.016
P04080	CSTB	Cystatin B (stefin B)	Cytoplasm	Peptidase	−1.011
P35221	CTNNA1	Catenin (cadherin-associated protein), α 1, 102 kDa	Plasma Membrane	Other	1.096
P17812	CTPS1	CTP synthase 1	Nucleus	Enzyme	1.338
P10619	CTSA	Cathepsin A	Cytoplasm	Peptidase	3.097
P07858	CTSB	Cathepsin B	Cytoplasm	Peptidase	−1.125
Q14247	CTTN	Cortactin	Plasma Membrane	Other	−1.161
O60888	CUTA	CutA divalent cation tolerance homolog (*E. coli*)	Cytoplasm	Other	−1.324
P00387	CYB5R3	Cytochrome *b*_5_ reductase 3	Cytoplasm	Enzyme	−1.018
P08574	CYC1	Cytochrome c-1	Cytoplasm	Enzyme	1.374
O00622	CYR61	Cysteine-rich, angiogenic inducer, 61	Extracellular Space	Other	1.367
P07108	DBI	Diazepam binding inhibitor (GABA receptor modulator, acyl-CoA binding protein)	Cytoplasm	Other	−1.099
Q16643	DBN1	Drebrin 1	Cytoplasm	Other	−1.109
Q92499	DDX1	DEAD (Asp-Glu-Ala-Asp) box helicase 1	Nucleus	Enzyme	−1.229
Q13838	DDX39B	DEAD (Asp-Glu-Ala-Asp) box polypeptide 39B	Nucleus	Enzyme	−1.021
O00571	DDX3X	DEAD (Asp-Glu-Ala-Asp) box helicase 3, X-linked	Cytoplasm	Enzyme	−1.042
P17844	DDX5	DEAD (Asp-Glu-Ala-Asp) box helicase 5	Nucleus	Enzyme	1.470
P26196	DDX6	DEAD (Asp-Glu-Ala-Asp) box helicase 6	Nucleus	Enzyme	−1.499
Q08211	DHX9	DEAH (Asp-Glu-Ala-His) box helicase 9	Nucleus	Enzyme	1.097
E9PEJ4	DLAT	Dihydrolipoamide *S*-acetyltransferase	Cytoplasm	Enzyme	−2.153
P31689	DNAJA1	DnaJ (Hsp40) homolog, subfamily A, member 1	Nucleus	Other	1.670
O60884	DNAJA2	DnaJ (Hsp40) homolog, subfamily A, member 2	Nucleus	Enzyme	−1.534
O75937	DNAJC8	DnaJ (Hsp40) homolog, subfamily C, member 8	Nucleus	Other	−1.971
G8JLD5	DNM1L	Dynamin 1-like	Cytoplasm	Enzyme	−1.011
Q9C005	DPY30	Dpy-30 homolog (*C. elegans*)	Nucleus	Other	−1.737
Q16555	DPYSL2	Dihydropyrimidinase-like 2	Cytoplasm	Enzyme	−1.124
Q14204	DYNC1H1	Dynein, cytoplasmic 1, heavy chain 1	Cytoplasm	Peptidase	−1.084
Q13409	DYNC1I2	Dynein, cytoplasmic 1, intermediate chain 2	Cytoplasm	Other	−1.024
P30084	ECHS1	Enoyl CoA hydratase, short chain, 1, mitochondrial	Cytoplasm	Enzyme	−1.351
P68104	EEF1A1	Eukaryotic translation elongation factor 1 α 1	Cytoplasm	Transcription regulator	−1.207
P24534	EEF1B2	Eukaryotic translation elongation factor 1 β 2	Cytoplasm	Transcription regulator	−1.208
P29692	EEF1D	Eukaryotic translation elongation factor 1 delta (guanine nucleotide exchange protein)	Cytoplasm	Transcription regulator	1.239
P26641	EEF1G	Eukaryotic translation elongation factor 1 γ	Cytoplasm	Transcription regulator	1.168
P13639	EEF2	Eukaryotic translation elongation factor 2	Cytoplasm	Transcription regulator	−1.089
Q9H4M9	EHD1	EH-domain containing 1	Cytoplasm	Other	−1.647
P05198	EIF2S1	Eukaryotic translation initiation factor 2, subunit 1 α, 35 kDa	Cytoplasm	Transcription regulator	−1.010
P20042	EIF2S2	Eukaryotic translation initiation factor 2, subunit 2 β, 38 kDa	Cytoplasm	Transcription regulator	−1.660
P55884	EIF3B	Eukaryotic translation initiation factor 3, subunit B	Cytoplasm	Transcription regulator	−1.009
Q99613	EIF3C	Eukaryotic translation initiation factor 3, subunit C	Other	Transcription regulator	1.374
Q13347	EIF3I	Eukaryotic translation initiation factor 3, subunit I	Cytoplasm	Transcription regulator	−1.163
Q7L2H7	EIF3M	Eukaryotic translation initiation factor 3, subunit M	Other	Other	1.451
P60842	EIF4A1	Eukaryotic translation initiation factor 4A1	Cytoplasm	Transcription regulator	1.192
E7EX73	EIF4G1	Eukaryotic translation initiation factor 4 γ, 1	Cytoplasm	Transcription regulator	1.458
Q15056	EIF4H	Eukaryotic translation initiation factor 4H	Cytoplasm	Transcription regulator	−1.387
P63241	EIF5A	Eukaryotic translation initiation factor 5A	Cytoplasm	Transcription regulator	−1.210
P56537	EIF6	Eukaryotic translation initiation factor 6	Cytoplasm	Transcription regulator	1.173
Q8N8S7	ENAH	Enabled homolog (Drosophila)	Plasma Membrane	Other	−1.048
P06733	ENO1	Enolase 1, (α)	Cytoplasm	Enzyme	−1.199
P09104	ENO2	Enolase 2 (γ, neuronal)	Cytoplasm	Enzyme	−1.698
P07814	EPRS	Glutamyl-prolyl-tRNA synthetase	Cytoplasm	Enzyme	−1.568
P30040	ERP29	Endoplasmic reticulum protein 29	Cytoplasm	Transporter	−1.048
P10768	ESD	Esterase D	Cytoplasm	Enzyme	−1.323
Q9BSJ8	ESYT1	Extended synaptotagmin-like protein 1	Cytoplasm	Other	2.370
H0YK49	ETFA	Electron-transfer-flavoprotein, α polypeptide	Cytoplasm	Transporter	−2.144
E7EQR4	EZR	Ezrin	Plasma Membrane	Other	−1.066
P49327	FASN	Fatty acid synthase	Cytoplasm	Enzyme	−1.453
P14324	FDPS	Farnesyl diphosphate synthase	Cytoplasm	Enzyme	−1.366
Q5W0X3	FKBP1A	FK506 binding protein 1A, 12 kDa	Cytoplasm	Enzyme	1.036
Q02790	FKBP4	FK506 binding protein 4, 59 kDa	Nucleus	Enzyme	−1.266
P21333	FLNA	Filamin A, α	Cytoplasm	Other	−1.157
O75369	FLNB	Filamin B, β	Cytoplasm	Other	−1.360
Q14315	FLNC	Filamin C, γ	Cytoplasm	Other	1.233
P02751	FN1	Fibronectin 1	Extracellular Space	Enzyme	−3.472
Q96I24	FUBP3	Far upstream element (FUSE) binding protein 3	Nucleus	Transcription regulator	−1.274
P35637	FUS	FUS RNA binding protein	Nucleus	Transcription regulator	1.049
Q13283	G3BP1	GTPase activating protein (SH3 domain) binding protein 1	Nucleus	Enzyme	−1.497
P11413	G6PD	Flucose-6-phosphate dehydrogenase	Cytoplasm	Enzyme	−1.504
Q14697	GANAB	Flucosidase, α; neutral AB	Cytoplasm	Enzyme	−1.122
P04406	GAPDH	Flyceraldehyde-3-phosphate dehydrogenase	Cytoplasm	Enzyme	−1.070
P41250	GARS	Flycyl-tRNA synthetase	Cytoplasm	Enzyme	1.052
Q92616	GCN1L1	GCN1 general control of amino-acid synthesis 1-like 1 (yeast)	Cytoplasm	Transcription regulator	−1.604
P31150	GDI1	GDP dissociation inhibitor 1	Cytoplasm	Other	−1.630
P50395	GDI2	GDP dissociation inhibitor 2	Cytoplasm	Other	−1.169
Q04760	GLO1	Flyoxalase I	Cytoplasm	Enzyme	−1.349
P00367	GLUD1	Flutamate dehydrogenase 1	Cytoplasm	Enzyme	1.184
E9PIR7	GML	Flycosylphosphatidylinositol anchored molecule like	Plasma Membrane	Other	−1.146
P49915	GMPS	Fuanine monphosphate synthase	Nucleus	Enzyme	−1.948
P04899	GNAI2	Fuanine nucleotide binding protein (G protein), α inhibiting activity polypeptide 2	Plasma Membrane	Other	1.544
J3KPE3	GNB2L1	Fuanine nucleotide binding protein (G protein), β polypeptide 2-like 1	Cytoplasm	Enzyme	1.209
P06744	GPI	Flucose-6-phosphate isomerase	Extracellular Space	Enzyme	−1.324
P09211	GSTP1	Flutathione *S*-transferase pi 1	Cytoplasm	Enzyme	−1.007
O75367	H2AFY	H2A histone family, member Y	Nucleus	Other	−1.571
K7EK07	H3F3A/H3F3B	H3 histone, family 3A	Nucleus	Other	1.037
Q16836	HADH	Hydroxyacyl-CoA dehydrogenase	Cytoplasm	Enzyme	−1.626
P40939	HADHA	Hydroxyacyl-CoA dehydrogenase/3-ketoacyl-CoA thiolase/enoyl-CoA hydratase (trifunctional protein), α subunit	Cytoplasm	Enzyme	−1.342
B4DDD8	HARS	Histidyl-tRNA synthetase	Cytoplasm	Enzyme	−1.358
P51858	HDGF	Hepatoma-derived growth factor	Extracellular Space	Growth factor	−2.205
Q00341	HDLBP	High density lipoprotein binding protein	Nucleus	Transporter	−1.572
Q5URX0	HEXB	Hexosaminidase B (β polypeptide)	Cytoplasm	Enzyme	1.160
P49773	HINT1	Histidine triad nucleotide binding protein 1	Nucleus	Enzyme	−1.864
P16403	HIST1H1C	Histone cluster 1, H1c	Nucleus	Other	−1.808
Q99880	HIST1H2BL	Histone cluster 1, H2bl	Nucleus	Other	1.029
Q16777	HIST2H2AC	Histone cluster 2, H2ac	Nucleus	Other	−1.380
P30447	HLA-A	Major histocompatibility complex, class I, A	Plasma Membrane	Other	−1.339
P18465	HLA-B	Major histocompatibility complex, class I, B	Plasma Membrane	Transmembrane receptor	1.128
Q5T7C4	HMGB1	High mobility group box 1	Nucleus	Transcription regulator	−3.172
Q9UK76	HN1	Hematological and neurological expressed 1	Nucleus	Other	−1.199
F8W6I7	HNRNPA1	Heterogeneous nuclear ribonucleoprotein A1	Nucleus	Other	1.329
P22626	HNRNPA2B1	Heterogeneous nuclear ribonucleoprotein A2/B1	Nucleus	Other	−1.102
P51991	HNRNPA3	Heterogeneous nuclear ribonucleoprotein A3	Nucleus	Other	1.006
E9PCY7	HNRNPH1	Heterogeneous nuclear ribonucleoprotein H1 (H)	Nucleus	Other	1.224
P31942	HNRNPH3	Heterogeneous nuclear ribonucleoprotein H3 (2H9)	Nucleus	Other	1.223
P61978	HNRNPK	Heterogeneous nuclear ribonucleoprotein K	Nucleus	Other	−1.191
P14866	HNRNPL	Heterogeneous nuclear ribonucleoprotein L	Nucleus	Other	−1.360
P52272	HNRNPM	Heterogeneous nuclear ribonucleoprotein M	Nucleus	Other	1.039
O43390	HNRNPR	Heterogeneous nuclear ribonucleoprotein R	Nucleus	Other	−1.090
Q00839	HNRNPU	Heterogeneous nuclear ribonucleoprotein U (scaffold attachment factor A)	Nucleus	Transporter	−1.169
Q99714	HSD17B10	Hydroxysteroid (17-β) dehydrogenase 10	Cytoplasm	Enzyme	−1.586
P07900	HSP90AA1	Heat shock protein 90 kDa α (cytosolic), class A member 1	Cytoplasm	Enzyme	1.196
P08238	HSP90AB1	Heat shock protein 90 kDa α (cytosolic), class B member 1	Cytoplasm	Enzyme	1.045
P14625	HSP90B1	Heat shock protein 90 kDa β (GRP94), member 1	Cytoplasm	Other	−1.036
P34932	HSPA4	Heat shock 70 kDa protein 4	Cytoplasm	Other	−1.068
P11021	HSPA5	Heat shock 70 kDa protein 5 (glucose-regulated protein, 78 kDa)	Cytoplasm	Enzyme	−1.178
P11142	HSPA8	Heat shock 70 kDa protein 8	Cytoplasm	Enzyme	1.258
P38646	HSPA9	Heat shock 70 kDa protein 9 (mortalin)	Cytoplasm	Other	−1.004
P04792	HSPB1	Heat shock 27 kDa protein 1	Cytoplasm	Other	−1.941
P10809	HSPD1	Heat shock 60 kDa protein 1 (chaperonin)	Cytoplasm	Enzyme	−1.182
Q9Y4L1	HYOU1	Hypoxia up-regulated 1	Cytoplasm	Other	−1.437
P41252	IARS	Isoleucyl-tRNA synthetase	Cytoplasm	Enzyme	−1.047
Q13907	IDI1	Isopentenyl-diphosphate delta isomerase 1	Cytoplasm	Enzyme	−1.544
Q01628	IFITM3	Interferon induced transmembrane protein 3	Plasma Membrane	Other	−1.052
P11717	IGF2R	Insulin-like growth factor 2 receptor	Plasma Membrane	Transmembrane receptor	1.187
Q12905	ILF2	Interleukin enhancer binding factor 2	Nucleus	Transcription regulator	1.264
Q12906	ILF3	Interleukin enhancer binding factor 3, 90 kDa	Nucleus	Transcription regulator	1.246
Q16891	IMMT	Inner membrane protein, mitochondrial	Cytoplasm	Other	1.565
H0Y4R1	IMPDH2	IMP (inosine 5′-monophosphate) dehydrogenase 2	Cytoplasm	Enzyme	−1.171
O00410	IPO5	Importin 5	Nucleus	Transporter	1.077
O95373	IPO7	Importin 7	Nucleus	Transporter	1.355
Q96P70	IPO9	Importin 9	Nucleus	Transporter	−1.113
P46940	IQGAP1	IQ motif containing GTPase activating protein 1	Cytoplasm	Other	1.136
P05556	ITGB1	Integrin, β 1 (fibronectin receptor, β polypeptide, antigen CD29 includes MDF2, MSK12)	Plasma Membrane	Transmembrane receptor	−1.145
Q07666	KHDRBS1	KH domain containing, RNA binding, signal transduction associated 1	Nucleus	Transcription regulator	−1.175
Q92945	KHSRP	KH-type splicing regulatory protein	Nucleus	Enzyme	−1.284
P33176	KIF5B	Kinesin family member 5B	Cytoplasm	Other	1.332
P52292	KPNA2	Karyopherin α 2 (RAG cohort 1, importin α 1)	Nucleus	Transporter	1.154
O00505	KPNA3	Karyopherin α 3 (importin α 4)	Nucleus	Transporter	2.127
Q14974	KPNB1	Karyopherin (importin) β 1	Nucleus	Transporter	1.073
P04264	KRT1	Keratin 1	Cytoplasm	Other	−5.784
P05783	KRT18	Keratin 18	Cytoplasm	Other	−1.122
P05787	KRT8	Keratin 8	Cytoplasm	Other	−1.469
P35527	KRT9	Keratin 9	Other	Other	−4.460
P07942	LAMB1	Laminin, β 1	Extracellular Space	Other	−1.088
C9J9W2	LASP1	LIM and SH3 protein 1	Cytoplasm	Transporter	−1.654
P00338	LDHA	Lactate dehydrogenase A	Cytoplasm	Enzyme	−1.361
P07195	LDHB	Lactate dehydrogenase B	Cytoplasm	Enzyme	−1.428
P09382	LGALS1	Lectin, galactoside-binding, soluble, 1	Extracellular Space	Other	−1.161
P02545	LMNA	Lamin A/C	Nucleus	Other	1.095
P20700	LMNB1	Lamin B1	Nucleus	Other	−1.253
Q01081	LOC102724594/U2AF1	U2 small nuclear RNA auxiliary factor 1	Nucleus	Other	1.047
K7EJE8	LONP1	Lon peptidase 1, mitochondrial	Cytoplasm	Peptidase	−1.410
P42704	LRPPRC	Leucine-rich pentatricopeptide repeat containing	Cytoplasm	Other	−1.059
Q96AG4	LRRC59	Leucine rich repeat containing 59	Cytoplasm	Other	1.228
P46821	MAP1B	Microtubule-associated protein 1B	Cytoplasm	Other	1.036
P27816	MAP4	Microtubule-associated protein 4	Cytoplasm	Other	−1.561
P28482	MAPK1	Mitogen-activated protein kinase 1	Cytoplasm	Kinase	−1.739
Q15691	MAPRE1	Microtubule-associated protein, RP/EB family, member 1	Cytoplasm	Other	1.418
P29966	MARCKS	Myristoylated alanine-rich protein kinase C substrate	Plasma Membrane	Other	−2.498
P56192	MARS	Methionyl-tRNA synthetase	Cytoplasm	Enzyme	1.049
P43243	MATR3	Matrin 3	Nucleus	Other	−1.307
P25205	MCM3	Minichromosome maintenance complex component 3	Nucleus	Enzyme	−1.411
P33991	MCM4	Minichromosome maintenance complex component 4	Nucleus	Enzyme	−1.472
Q14566	MCM6	Minichromosome maintenance complex component 6	Nucleus	Enzyme	−1.238
P33993	MCM7	Minichromosome maintenance complex component 7	Nucleus	Enzyme	−1.223
P40925	MDH1	Malate dehydrogenase 1, NAD (soluble)	Cytoplasm	Enzyme	−1.639
P40926	MDH2	Malate dehydrogenase 2, NAD (mitochondrial)	Cytoplasm	Enzyme	−1.298
P26038	MSN	Moesin	Plasma Membrane	Other	1.079
Q14764	MVP	Major vault protein	Nucleus	Other	1.636
P35580	MYH10	Myosin, heavy chain 10, non-muscle	Cytoplasm	Other	1.275
P35579	MYH9	Myosin, heavy chain 9, non-muscle	Cytoplasm	Enzyme	−1.104
P19105	MYL12A	Myosin, light chain 12A, regulatory, non-sarcomeric	Cytoplasm	Other	1.316
F8W1N5	NACA	Nascent polypeptide-associated complex α subunit	Cytoplasm	Transcription regulator	−1.282
F8VY35	NAP1L1	Nucleosome assembly protein 1-like 1	Nucleus	Other	1.173
Q99733	NAP1L4	Nucleosome assembly protein 1-like 4	Cytoplasm	Other	−1.300
P49321	NASP	Nuclear autoantigenic sperm protein (histone-binding)	Nucleus	Other	−1.640
P19338	NCL	Nucleolin	Nucleus	Other	−1.037
Q32Q12	NME1-NME2	NME1-NME2 readthrough	Cytoplasm	Other	−1.298
P40261	NNMT	Nicotinamide *N*-methyltransferase	Cytoplasm	Enzyme	−2.513
Q15233	NONO	Non-POU domain containing, octamer-binding	Nucleus	Other	1.374
E9PLK3	NPEPPS	Aminopeptidase puromycin sensitive	Cytoplasm	Peptidase	−1.868
Q8TAT6	NPLOC4	Nuclear protein localization 4 homolog (*S. cerevisiae*)	Nucleus	Other	1.135
P06748	NPM1	Nucleophosmin (nucleolar phosphoprotein B23, numatrin)	Nucleus	Transcription regulator	1.166
Q9UNZ2	NSFL1C	NSFL1 (p97) cofactor (p47)	Cytoplasm	Other	−1.139
Q14980	NUMA1	Nuclear mitotic apparatus protein 1	Nucleus	Other	−1.202
Q9NTK5	OLA1	Obg-like ATPase 1	Cytoplasm	Other	1.159
Q96FW1	OTUB1	OTU deubiquitinase, ubiquitin aldehyde binding 1	Cytoplasm	Enzyme	−1.304
P07237	P4HB	Prolyl 4-hydroxylase, β polypeptide	Cytoplasm	Enzyme	−1.341
Q9UQ80	PA2G4	Proliferation-associated 2G4, 38kDa	Nucleus	Transcription regulator	−1.316
E7EQV3	PABPC1	Poly(A) binding protein, cytoplasmic 1	Cytoplasm	Transcription regulator	1.235
P68402	PAFAH1B2	Platelet-activating factor acetylhydrolase 1b, catalytic subunit 2 (30 kDa)	Cytoplasm	Enzyme	1.089
E9PBS1	PAICS	Phosphoribosylaminoimidazole carboxylase, phosphoribosylaminoimidazole succinocarboxamide synthetase	Cytoplasm	Enzyme	−1.354
Q99497	PARK7	Parkinson protein 7	Nucleus	Enzyme	−1.711
Q15365	PCBP1	Poly(rC) binding protein 1	Nucleus	Transcription regulator	−1.493
F8VZX2	PCBP2	Poly(rC) binding protein 2	Nucleus	Other	1.266
E7EV56	PCM1	Pericentriolar material 1	Cytoplasm	Other	1.175
B7Z972	PCMT1	Protein-l-isoaspartate (d-aspartate) *O*-methyltransferase	Cytoplasm	Enzyme	−1.351
P12004	PCNA	Proliferating cell nuclear antigen	Nucleus	Enzyme	−1.321
E7EPW6	PDCD6	Programmed cell death 6	Cytoplasm	Other	−1.147
F8VPF3	PDE6H	Phosphodiesterase 6H, cGMP-specific, cone, γ	Cytoplasm	Enzyme	−1.043
P30101	PDIA3	Protein disulfide isomerase family A, member 3	Cytoplasm	Peptidase	1.167
P13667	PDIA4	Protein disulfide isomerase family A, member 4	Cytoplasm	Enzyme	−1.155
Q15084	PDIA6	Protein disulfide isomerase family A, member 6	Cytoplasm	Enzyme	−1.161
O00151	PDLIM1	PDZ and LIM domain 1	Cytoplasm	Transcription regulator	−1.397
F2Z2Y4	PDXK	Pyridoxal (pyridoxine, vitamin B6) kinase	Cytoplasm	Kinase	−1.008
Q15121	PEA15	Phosphoprotein enriched in astrocytes 15	Cytoplasm	Transporter	1.073
P30086	PEBP1	Phosphatidylethanolamine binding protein 1	Cytoplasm	Other	−1.552
Q01813	PFKP	Phosphofructokinase, platelet	Cytoplasm	Kinase	1.313
P07737	PFN1	Profilin 1	Cytoplasm	Other	−1.405
P18669	PGAM1	Phosphoglycerate mutase 1 (brain)	Cytoplasm	Phosphatase	−1.701
P52209	PGD	Phosphogluconate dehydrogenase	Cytoplasm	Enzyme	−1.398
P00558	PGK1	Phosphoglycerate kinase 1	Cytoplasm	Kinase	−1.287
P36871	PGM1	Phosphoglucomutase 1	Cytoplasm	Enzyme	−1.393
F5GY37	PHB2	Prohibitin 2	Cytoplasm	Transcription regulator	−1.175
O43175	PHGDH	Phosphoglycerate dehydrogenase	Cytoplasm	Enzyme	−1.552
Q13492	PICALM	Phosphatidylinositol binding clathrin assembly protein	Cytoplasm	Other	−1.195
P14618	PKM	Pyruvate kinase, muscle	Cytoplasm	Kinase	−1.341
Q15149	PLEC	Plectin	Cytoplasm	Other	1.326
O60664	PLIN3	Perilipin 3	Cytoplasm	Other	−1.084
E7ETU9	PLOD2	Procollagen-lysine, 2-oxoglutarate 5-dioxygenase 2	Cytoplasm	Enzyme	1.150
P13797	PLS3	Plastin 3	Cytoplasm	Other	−1.143
Q15181	PPA1	Pyrophosphatase (inorganic) 1	Cytoplasm	Enzyme	−1.263
P62937	PPIA	Peptidylprolyl isomerase A (cyclophilin A)	Cytoplasm	Enzyme	−1.057
O15355	PPM1G	Protein phosphatase, Mg^2+^/Mn^2+^ dependent, 1G	Nucleus	Phosphatase	1.144
P62136	PPP1CA	Protein phosphatase 1, catalytic subunit, α isozyme	Cytoplasm	Phosphatase	−1.250
P67775	PPP2CA	Protein phosphatase 2, catalytic subunit, α isozyme	Cytoplasm	Phosphatase	1.012
P30153	PPP2R1A	Protein phosphatase 2, regulatory subunit A, α	Cytoplasm	Phosphatase	−1.191
Q06830	PRDX1	Peroxiredoxin 1	Cytoplasm	Enzyme	−2.056
P32119	PRDX2	Peroxiredoxin 2	Cytoplasm	Enzyme	−1.479
Q13162	PRDX4	Peroxiredoxin 4	Cytoplasm	Enzyme	1.346
P30044	PRDX5	Peroxiredoxin 5	Cytoplasm	Enzyme	−1.297
K7ELL7	PRKCSH	Protein kinase C substrate 80K–H	Cytoplasm	Enzyme	−1.483
E7EUY0	PRKDC	Protein kinase, DNA-activated, catalytic polypeptide	Nucleus	Kinase	−1.313
H0YJX6	PRMT5	Protein arginine methyltransferase 5	Cytoplasm	Enzyme	−1.975
Q9UMS4	PRPF19	Pre-mRNA processing factor 19	Nucleus	Enzyme	−1.155
P25786	PSMA1	Proteasome (prosome, macropain) subunit, α type, 1	Cytoplasm	Peptidase	−1.070
P25787	PSMA2	Proteasome (prosome, macropain) subunit, α type, 2	Cytoplasm	Peptidase	−1.069
H0YLC2	PSMA4	Proteasome (prosome, macropain) subunit, α type, 4	Cytoplasm	Peptidase	-1.204
P28066	PSMA5	Proteasome (prosome, macropain) subunit, α type, 5	Cytoplasm	Peptidase	1.018
O14818	PSMA7	Proteasome (prosome, macropain) subunit, α type, 7	Cytoplasm	Peptidase	−1.157
P20618	PSMB1	Proteasome (prosome, macropain) subunit, β type, 1	Cytoplasm	Peptidase	1.208
P62191	PSMC1	Proteasome (prosome, macropain) 26S subunit, ATPase, 1	Nucleus	Peptidase	−1.298
P43686	PSMC4	Proteasome (prosome, macropain) 26S subunit, ATPase, 4	Nucleus	Peptidase	−1.249
Q13200	PSMD2	Proteasome (prosome, macropain) 26S subunit, non−ATPase, 2	Cytoplasm	Other	1.339
Q06323	PSME1	Proteasome (prosome, macropain) activator subunit 1 (PA28 α)	Cytoplasm	Other	−1.845
Q9UL46	PSME2	Proteasome (prosome, macropain) activator subunit 2 (PA28 β)	Cytoplasm	Peptidase	−1.010
P26599	PTBP1	Polypyrimidine tract binding protein 1	Nucleus	Enzyme	1.178
Q15185	PTGES3	Prostaglandin E synthase 3 (cytosolic)	Cytoplasm	Enzyme	−1.312
B8ZZQ6	PTMA	Prothymosin, α	Nucleus	Other	−1.826
Q6NZI2	PTRF	Polymerase I and transcript release factor	Nucleus	Transcription regulator	2.165
Q9UHX1	PUF60	Poly-U binding splicing factor 60 kDa	Nucleus	Other	−1.146
P11216	PYGB	Phosphorylase, glycogen; brain	Cytoplasm	Enzyme	−1.093
E9PK47	PYGL	Phosphorylase, glycogen, liver	Cytoplasm	Enzyme	−1.252
P47897	QARS	Glutaminyl-tRNA synthetase	Cytoplasm	Enzyme	1.066
B4DQU5	RAB11A	RAB11A, member RAS oncogene family	Cytoplasm	Enzyme	−1.365
E9PLD0	RAB1B	RAB1B, member RAS oncogene family	Cytoplasm	Other	−1.182
P51149	RAB7A	RAB7A, member RAS oncogene family	Cytoplasm	Enzyme	1.158
P54727	RAD23B	RAD23 homolog B (*S. cerevisiae*)	Nucleus	Other	1.005
F5H018	RAN	RAN, member RAS oncogene family	Nucleus	Enzyme	−1.229
C9JJ34	RANBP1	RAN binding protein 1	Nucleus	Other	−1.461
P54136	RARS	Arginyl-tRNA synthetase	Cytoplasm	Enzyme	−1.180
P98179	RBM3	RNA binding motif (RNP1, RRM) protein 3	Cytoplasm	Other	1.376
Q15293	RCN1	Reticulocalbin 1, EF-hand calcium binding domain	Cytoplasm	Other	1.027
C9JNR4	RHOA	Ras homolog family member A	Cytoplasm	Enzyme	−1.521
P13489	RNH1	Ribonuclease/angiogenin inhibitor 1	Cytoplasm	Other	1.064
X1WI28	RPL10	Ribosomal protein L10	Cytoplasm	Other	1.461
P62906	RPL10A	Ribosomal protein L10a	Nucleus	Other	−1.075
P30050	RPL12	Ribosomal protein L12	Nucleus	Other	1.288
F8VUA6	RPL18	Ribosomal protein L18	Cytoplasm	Other	−1.330
P46778	RPL21	Ribosomal protein L21	Cytoplasm	Other	−1.204
P35268	RPL22	Ribosomal protein L22	Nucleus	Other	1.339
C9JD32	RPL23	Ribosomal protein L23	Cytoplasm	Other	1.026
P61353	RPL27	Ribosomal protein L27	Cytoplasm	Other	1.373
P39023	RPL3	Ribosomal protein L3	Cytoplasm	Other	−1.206
P36578	RPL4	Ribosomal protein L4	Cytoplasm	Enzyme	1.044
P46777	RPL5	Ribosomal protein L5	Cytoplasm	Other	−1.065
Q02878	RPL6	Ribosomal protein L6	Cytoplasm	Other	1.314
P18124	RPL7	Ribosomal protein L7	Nucleus	Transcription regulator	1.340
P62424	RPL7A	Ribosomal protein L7a	Cytoplasm	Other	1.141
G3V1A1	RPL8	Ribosomal protein L8	Other	Other	−1.191
F8VWS0	RPLP0	Ribosomal protein, large, P0	Cytoplasm	Other	−1.421
P05386	RPLP1	Ribosomal protein, large, P1	Cytoplasm	Other	−1.274
P05387	RPLP2	Ribosomal protein, large, P2	Cytoplasm	Other	−1.062
P04843	RPN1	Ribophorin I	Cytoplasm	Enzyme	1.067
P04844	RPN2	Ribophorin II	Cytoplasm	Enzyme	−1.171
P46783	RPS10	Ribosomal protein S10	Cytoplasm	Other	−1.009
P62280	RPS11	Ribosomal protein S11	Cytoplasm	Other	1.335
P25398	RPS12	Ribosomal protein S12	Cytoplasm	Other	−1.062
K7EJ78	RPS15	Ribosomal protein S15	Cytoplasm	Other	1.152
H0YEN5	RPS2	Ribosomal protein S2	Cytoplasm	Other	1.092
E7ETK0	RPS24	Ribosomal protein S24	Cytoplasm	Other	1.157
P62979	RPS27A	Ribosomal protein S27a	Cytoplasm	Other	1.399
P23396	RPS3	Ribosomal protein S3	Cytoplasm	Enzyme	1.251
D6RG13	RPS3A	Ribosomal protein S3A	Nucleus	Other	1.184
P62701	RPS4X	Ribosomal protein S4, X-linked	Cytoplasm	Other	1.236
M0R0F0	RPS5	Ribosomal protein S5	Cytoplasm	Other	−1.080
P62081	RPS7	Ribosomal protein S7	Cytoplasm	Other	−1.189
P62241	RPS8	Ribosomal protein S8	Cytoplasm	Other	1.581
C9J9K3	RPSA	Ribosomal protein SA	Cytoplasm	Transcription regulator	1.267
Q9P2E9	RRBP1	Ribosome binding protein 1	Cytoplasm	Other	1.082
Q9NQC3	RTN4	Reticulon 4	Cytoplasm	Other	−1.099
R4GN98	S100A6	S100 calcium binding protein A6	Cytoplasm	Transporter	−1.190
M0QZS6	SAE1	SUMO1 activating enzyme subunit 1	Cytoplasm	Enzyme	1.058
D6RFM5	SDHA	uccinate dehydrogenase complex, subunit A, flavoprotein (Fp)	Cytoplasm	Enzyme	1.035
P55735	SEC13	SEC13 homolog (*S. cerevisiae*)	Cytoplasm	Transporter	1.029
Q15019	SEPT2	Septin 2	Cytoplasm	Enzyme	−1.140
H0Y3Y4	SEPT7	Septin 7	Cytoplasm	Other	1.266
Q9UHD8	SEPT9	Septin 9	Cytoplasm	Enzyme	1.063
Q8NC51	SERBP1	SERPINE1 mRNA binding protein 1	Cytoplasm	Other	1.003
P50454	SERPINH1	Serpin peptidase inhibitor, clade H (heat shock protein 47), member 1, (collagen binding protein 1)	Extracellular Space	Other	−1.178
Q01105	SET	SET nuclear proto-oncogene	Nucleus	Phosphatase	−1.282
Q15459	SF3A1	Splicing factor 3a, subunit 1, 120 kDa	Nucleus	Other	1.024
O75533	SF3B1	Splicing factor 3b, subunit 1, 155 kDa	Nucleus	Other	1.359
P31947	SFN	Stratifin	Cytoplasm	Other	−1.586
P23246	SFPQ	Splicing factor proline/glutamine-rich	Nucleus	Other	1.009
Q9H299	SH3BGRL3	SH3 domain binding glutamate-rich protein like 3	Nucleus	Other	1.688
P34897	SHMT2	Serine hydroxymethyltransferase 2 (mitochondrial)	Cytoplasm	Enzyme	−1.297
F8VVM2	SLC25A3	Solute carrier family 25 (mitochondrial carrier; phosphate carrier), member 3	Cytoplasm	Transporter	1.165
P05141	SLC25A5	Solute carrier family 25 (mitochondrial carrier; adenine nucleotide translocator), member 5	Cytoplasm	Transporter	−1.559
I7HJJ0	SLC25A6	Solute carrier family 25 (mitochondrial carrier; adenine nucleotide translocator), member 6	Cytoplasm	Transporter	1.304
Q96QD8	SLC38A2	Solute carrier family 38, member 2	Plasma Membrane	Transporter	−1.082
Q7KZF4	SND1	Staphylococcal nuclease and tudor domain containing 1	Nucleus	Enzyme	−1.112
P62314	SNRPD1	Small nuclear ribonucleoprotein D1 polypeptide 16 kDa	Nucleus	Other	−1.174
H3BT13	SNRPD3	Small nuclear ribonucleoprotein D3 polypeptide 18 kDa	Nucleus	Other	1.288
O60493	SNX3	Sorting nexin 3	Cytoplasm	Transporter	1.117
P00441	SOD1	Superoxide dismutase 1, soluble	Cytoplasm	Enzyme	−1.264
Q13813	SPTAN1	Spectrin, α, non-erythrocytic 1	Plasma Membrane	Other	1.056
Q01082	SPTBN1	Spectrin, β, non-erythrocytic 1	Plasma Membrane	Other	−1.545
E7EMC7	SQSTM1	Sequestosome 1	Cytoplasm	Transcription regulator	1.310
Q08945	SSRP1	Structure specific recognition protein 1	Nucleus	Other	−1.498
P42224	STAT1	Signal transducer and activator of transcription 1, 91 kDa	Nucleus	Transcription regulator	−1.353
P31948	STIP1	Stress-induced phosphoprotein 1	Cytoplasm	Other	−1.086
A2A2D0	STMN1	Stathmin 1	Cytoplasm	Other	−1.445
Q9UJZ1	STOML2	Stomatin (EPB72)-like 2	Plasma Membrane	Other	1.325
Q9Y5B9	SUPT16H	Suppressor of Ty 16 homolog (*S. cerevisiae*)	Nucleus	Transcription regulator	−1.137
Q5T8U5	SURF4	Surfeit 4	Cytoplasm	Other	1.327
O60506	SYNCRIP	Synaptotagmin binding, cytoplasmic RNA interacting protein	Nucleus	Other	−1.378
Q01995	TAGLN	Transgelin	Cytoplasm	Other	−1.657
P37802	TAGLN2	Transgelin 2	Cytoplasm	Other	−1.382
P37837	TALDO1	Transaldolase 1	Cytoplasm	Enzyme	−1.652
P26639	TARS	Threonyl-tRNA synthetase	Nucleus	Enzyme	−1.194
E5RIW3	TBCA	Tubulin folding cofactor A	Cytoplasm	Other	−1.404
P17987	TCP1	T-complex 1	Cytoplasm	Other	1.190
P02786	TFRC	Transferrin receptor	Plasma Membrane	Transporter	−1.018
B4E022	TKT	Transketolase	Cytoplasm	Enzyme	−1.717
Q9Y490	TLN1	Talin 1	Plasma Membrane	Other	−1.072
Q9NYL9	TMOD3	Tropomodulin 3 (ubiquitous)	Cytoplasm	Other	1.076
P42166	TMPO	Thymopoietin	Nucleus	Other	−1.488
F5H7V9	TNC	Tenascin C	Extracellular Space	Other	−2.480
Q92973	TNPO1	Transportin 1	Nucleus	Transporter	−1.378
P60174	TPI1	Triosephosphate isomerase 1	Cytoplasm	Enzyme	−1.620
Q5TCU3	TPM2	Tropomyosin 2 (β)	Other	Other	1.845
J3KN67	TPM3	Tropomyosin 3	Cytoplasm	Other	−1.265
P67936	TPM4	Tropomyosin 4	Cytoplasm	Other	−1.185
O14773	TPP1	Tripeptidyl peptidase I	Cytoplasm	Peptidase	−1.239
Q13263	TRIM28	Tripartite motif containing 28	Nucleus	Transcription regulator	−1.201
F8VQE1	TRMT1	tRNA methyltransferase 1 homolog (*S. cerevisiae*)	Extracellular Space	Enzyme	−1.271
P68363	TUBA1B	Tubulin, α 1b	Cytoplasm	Other	−1.056
Q5JP53	TUBB	Tubulin, β class I	Cytoplasm	Other	−1.029
P68371	TUBB4B	Tubulin, β 4B class IVb	Cytoplasm	Other	1.046
Q9BUF5	TUBB6	Tubulin, β 6 class V	Cytoplasm	Other	1.022
Q3ZCM7	TUBB8	Tubulin, β 8 class VIII	Cytoplasm	Other	−1.128
P49411	TUFM	Tu translation elongation factor, mitochondrial	Cytoplasm	Transcription regulator	−1.173
Q12792	TWF1	Twinfilin actin-binding protein 1	Cytoplasm	Kinase	−1.798
D6RG15	TWF2	Twinfilin actin-binding protein 2	Cytoplasm	Kinase	−1.347
P40222	TXLNA	Taxilin α	Extracellular Space	Cytokine	−1.162
Q86UY0	TXNDC5	Thioredoxin domain containing 5 (endoplasmic reticulum)	Cytoplasm	Enzyme	1.390
P26368	U2AF2	U2 small nuclear RNA auxiliary factor 2	Nucleus	Other	−1.209
Q16222	UAP1	UDP-N-acetylglucosamine pyrophosphorylase 1	Nucleus	Enzyme	1.145
P22314	UBA1	Ubiquitin-like modifier activating enzyme 1	Cytoplasm	Enzyme	1.182
P68036	UBE2L3	Ubiquitin-conjugating enzyme E2L 3	Nucleus	Enzyme	1.021	
F8VZ29	UBE2N	Ubiquitin-conjugating enzyme E2N	Cytoplasm	Enzyme	−1.650	
Q92575	UBXN4	UBX domain protein 4	Extracellular Space	Other	−1.082	
P09936	UCHL1	Ubiquitin carboxyl-terminal esterase L1 (ubiquitin thiolesterase)	Cytoplasm	Peptidase	−1.453	
P31930	UQCRC1	Ubiquinol-cytochrome c reductase core protein I	Cytoplasm	Enzyme	1.218	
Q9P0L0	VAPA	VAMP (vesicle-associated membrane protein)-associated protein A, 33 kDa	Plasma Membrane	Other	1.026	
P26640	VARS	Valyl-tRNA synthetase	Cytoplasm	Enzyme	−1.120	
K7ERT7	VAT1	Vesicle amine transport 1	Plasma Membrane	Transporter	−2.302	
P18206	VCL	Vinculin	Plasma Membrane	Enzyme	−1.175	
P55072	VCP	Valosin containing protein	Cytoplasm	Enzyme	−1.058	
P21796	VDAC1	Voltage-dependent anion channel 1	Cytoplasm	Ion channel	−1.335	
P45880	VDAC2	Voltage-dependent anion channel 2	Cytoplasm	Ion channel	−1.041	
P08670	VIM	Vimentin	Cytoplasm	Other	1.579	
Q96QK1	VPS35	Vacuolar protein sorting 35 homolog (*S. cerevisiae*)	Cytoplasm	Transporter	−1.641	
P23381	WARS	Tryptophanyl-tRNA synthetase	Cytoplasm	Enzyme	−1.477	
O75083	WDR1	WD repeat domain 1	Extracellular Space	Other	−1.206	
O14980	XPO1	exportin 1	Nucleus	Transporter	−1.091	
P13010	XRCC5	X-ray repair complementing defective repair in Chinese hamster cells 5 (double-strand-break rejoining)	Nucleus	Enzyme	−1.197	
P12956	XRCC6	X-ray repair complementing defective repair in Chinese hamster cells 6	Nucleus	Enzyme	−1.575	
P67809	YBX1	Y box binding protein 1	Nucleus	Transcription regulator	−1.196	
P16989	YBX3	Y box binding protein 3	Nucleus	Transcription regulator	−1.186	
P31946	YWHAB	Tyrosine 3-monooxygenase/tryptophan 5-monooxygenase activation protein, β	Cytoplasm	Transcription regulator	1.064	
P62258	YWHAE	Tyrosine 3-monooxygenase/tryptophan 5-monooxygenase activation protein, epsilon	Cytoplasm	Other	−1.216	
P61981	YWHAG	Tyrosine 3-monooxygenase/tryptophan 5-monooxygenase activation protein, γ	Cytoplasm	Other	−1.045	
Q04917	YWHAH	Tyrosine 3-monooxygenase/tryptophan 5-monooxygenase activation protein, η	Cytoplasm	Transcription regulator	−1.559	
P27348	YWHAQ	Tyrosine 3-monooxygenase/tryptophan 5-monooxygenase activation protein, θ	Cytoplasm	Other	1.180	
P63104	YWHAZ	Tyrosine 3-monooxygenase/tryptophan 5-monooxygenase activation protein, ζ	Cytoplasm	Enzyme	−1.123	
Q15942	ZYX	Zyxin	Plasma Membrane	Other	−1.100	

**Table 3:** 290 canonical signaling pathways regulated by vancomycin in HK-2 cells.

INGENUITY Canonical Pathways	−log(*p*-Value) ^a^	Ratio ^b^	*z*-Score ^c^	Molecules
eIF2 signaling	29.5	2.32 × 10^−1^	−0.229	RPL22, EIF3C, RPLP1, MAPK1, RPS3A, RPLP2, RPS8, EIF2S1, EIF4G1, RPL7, RPS11, RPS4X, RPS7, RPL6, RPL7A, EIF3B, PPP1CA, RPS3, RPS5, RPL18, RPS24, PABPC1, RPL4, RPL3, RPS2, RPL27, RPS10, RPL21, RPL23, RPL12, RPLP0, RPL10A, EIF3M, RPS12, EIF2S2, RPL8, EIF4A1, EIF3I, RPS15, RPL10, RPS27A, RPL5, RPSA
Remodeling of epithelial adherent junctions	16.2	2.94 × 10^−1^	0.000	TUBA1B, ACTR2, ARPC1B, TUBB4B, MAPRE1, RAB7A, CTNNA1, IQGAP1, TUBB, ACTR3, TUBB6, TUBB8, ARPC2, ZYX, VCL, ACTN4, DNM1L, ACTG1, ACTN1, ACTA1
Regulation of eIF4 and p70S6K signaling	16.0	1.85 × 10^−1^	−1.000	EIF3C, RPS3A, MAPK1, PPP2CA, RPS8, EIF4G1, EIF2S1, RPS4X, RPS11, RPS7, EIF3B, RPS3, RPS5, RPS24, ITGB1, PABPC1, RPS2, RPS10, RPS12, EIF3M, EIF2S2, PPP2R1A, EIF4A1, EIF3I, RPS15, RPS27A, RPSA
mTOR signaling	11.4	1.33 × 10^−1^	−0.447	EIF3C, RPS3A, MAPK1, PPP2CA, RPS8, FKBP1A, EIF4G1, RPS4X, RPS11, RPS7, EIF3B, RPS3, RPS5, RPS24, RPS2, RPS10, RPS12, EIF3M, PPP2R1A, RHOA, EIF4A1, RPS15, EIF3I, RPS27A, RPSA
Protein ubiquitination pathway	11.4	1.14 × 10^−1^		B2M, PSMA7, HLA-A, UBE2N, HLA-B, HSPA5, DNAJA1, UCHL1, HSPA4, HSP90B1, HSP90AB1, DNAJC8, PSMA2, HSPA9, PSME2, PSMC4, PSMA1, HSPD1, HSPA8, PSME1, PSMC1, UBE2L3, PSMD2, PSMA5, PSMA4, PSMB1, HSP90AA1, UBA1, HSPB1
Glycolysis I	11.4	4.40 × 10^−1^		PGK1, ENO1, GPI, TPI1, PGAM1, PKM, ENO2, ALDOA, GAPDH, PFKP, ALDOC
Caveolar-mediated endocytosis signaling	11.0	2.22 × 10^−1^		ITGB1, B2M, FLNB, HLA-A, HLA-B, COPA, COPE, COPB2, COPB1, COPG1, FLNA, FLNC, CAV1, PTRF, ACTG1, ACTA1
Ran signaling	10.3	5.29 × 10^−1^		KPNB1, KPNA3, CSE1L, KPNA2, TNPO1, RAN, XPO1, RANBP1, IPO5
Gluconeogenesis I	9.88	4.00 × 10^−1^		PGK1, ENO1, GPI, PGAM1, ENO2, ALDOA, GAPDH, MDH1, MDH2, ALDOC
Epithelial adherent junction signaling	9.51	1.37 × 10^−1^		TUBA1B, MYH10, ACTR2, MYH9, ARPC1B, TUBB4B, CTNNA1, IQGAP1, TUBB, ACTR3, TUBB6, TUBB8, ARPC2, RHOA, ZYX, VCL, ACTN4, ACTG1, ACTN1, ACTA1
tRNA charging	8.93	2.82 × 10^−1^		WARS, RARS, GARS, HARS, TARS, AARS, VARS, MARS, IARS, EPRS, QARS
Actin cytoskeleton signaling	8.59	1.06 × 10^−1^		ITGB1, ACTR2, MYH10, MYH9, FN1, PFN1, CFL1, MAPK1, ARPC1B, TLN1, IQGAP1, ACTR3, FLNA, EZR, ARPC2, RHOA, VCL, ACTN4, ACTG1, ACTA1, ACTN1, MSN, MYL12A
Regulation of cellular mechanics by calpain protease	8.10	2.11 × 10^−1^	0.378	ITGB1, CAPNS1, MAPK1, EZR, CAPN1, TLN1, CAPN2, VCL, CAST, ACTN4, CDK1, ACTN1
Integrin signaling	7.74	1.04 × 10^−1^	−0.894	ITGB1, ACTR2, ARPC1B, MAPK1, ARF1, TLN1, ACTR3, CAPNS1, CAPN1, RHOA, ARPC2, CAV1, ZYX, CAPN2, VCL, ACTN4, CTTN, ACTG1, ACTN1, ACTA1, MYL12A
Virus entry via endocytic pathways	7.62	1.57 × 10^−1^		B2M, ITGB1, FLNB, FLNC, FLNA, HLA-A, AP2B1, HLA-B, CLTC, CAV1, TFRC, AP1B1, ACTG1, ACTA1
Unfolded protein response	7.31	2.04 × 10^−1^		HSPA8, HSPA4, CALR, P4HB, HSP90B1, UBXN4, HSPA9, VCP, CANX, DNAJA2, HSPA5
ILK signaling	6.94	1.02 × 10^−1^	−1.414	ITGB1, FLNB, MYH10, MYH9, FN1, CFL1, MAPK1, PPP2CA, VIM, PPP2R1A, FLNA, FLNC, RHOA, KRT18, ACTN4, ACTG1, ACTN1, ACTA1, NACA
14-3-3-mediated signaling	6.89	1.28 × 10^−1^		TUBA1B, YWHAG, YWHAH, YWHAE, MAPK1, TUBB4B, PDIA3, YWHAB, YWHAZ, VIM, TUBB, YWHAQ, TUBB6, TUBB8, SFN
Rhogdi signaling	6.73	1.04 × 10^−1^	−1.000	ITGB1, GDI1, ACTR2, CFL1, ARPC1B, GNB2L1, GDI2, GNAI2, ACTR3, RHOA, ARPC2, EZR, CD44, ARHGDIA, ACTG1, ACTA1, MSN, MYL12A
RhoA signaling	6.65	1.23 × 10^−1^	−0.258	ACTR2, PFN1, SEPT9, ARPC1B, CFL1, SEPT7, ACTR3, ARPC2, EZR, RHOA, ACTG1, ACTA1, SEPT2, MSN, MYL12A
Signaling by Rho family GTPases	6.64	8.97 × 10^−2^	0.471	ITGB1, ACTR2, CFL1, MAPK1, SEPT9, ARPC1B, SEPT7, GNB2L1, VIM, IQGAP1, GNAI2, STMN1, ACTR3, EZR, ARPC2, RHOA, ACTG1, ACTA1, SEPT2, MSN, MYL12A
PI3K/Akt signaling	5.84	1.14 × 10^−1^	−0.535	YWHAQ, ITGB1, PPP2R1A, HSP90B1, YWHAG, HSP90AB1, YWHAH, MAPK1, YWHAE, YWHAB, PPP2CA, YWHAZ, HSP90AA1, SFN
Germ cell-sertoli cell junction signaling	5.82	1.00 × 10^−1^		ITGB1, TUBA1B, MAPK1, CFL1, TUBB4B, CTNNA1, TUBB, IQGAP1, TUBB6, TUBB8, RHOA, ZYX, ACTN4, ACTG1, ACTN1, ACTA1
Cell cycle: G_2_/M DNA damage checkpoint regulation	5.69	1.84 × 10^−1^	−0.447	YWHAQ, PRKDC, YWHAG, YWHAE, YWHAH, YWHAB, YWHAZ, SFN, CDK1
Clathrin-mediated endocytosis signaling	5.63	9.19 × 10^−2^		ITGB1, ACTR2, ARPC1B, PICALM, AP2B1, CLTC, RAB7A, AP1B1, HSPA8, ACTR3, ARPC2, RAB11A, TFRC, DNM1L, CTTN, ACTG1, ACTA1
Glutaryl-CoA degradation	5.51	4.55 × 10^−1^		HSD17B10, ACAT2, ACAT1, HADHA, HADH
p70S6K signaling	5.26	1.09 × 10^−1^	0.000	GNAI2, YWHAQ, PPP2R1A, YWHAG, YWHAH, MAPK1, YWHAE, EEF2, YWHAB, PDIA3, PPP2CA, YWHAZ, SFN
Hippo signaling	5.20	1.28 × 10^−1^	0.632	YWHAQ, PPP2R1A, YWHAG, YWHAE, YWHAH, PPP2CA, YWHAB, CD44, YWHAZ, SFN, PPP1CA
Regulation of actin-based motility by rho	4.96	1.21 × 10^−1^	−1.265	ITGB1, ACTR2, PFN1, ACTR3, ARPC1B, CFL1, ARPC2, RHOA, ARHGDIA, ACTA1, MYL12A
Isoleucine degradation I	4.89	3.57 × 10^−1^		HSD17B10, ECHS1, ACAT2, ACAT1, HADHA
Aldosterone signaling in epithelial cells	4.76	9.21 × 10^−2^		MAPK1, PDIA3, HSPA9, HSPD1, DNAJA1, HSPA5, HSPA8, HSPA4, HSP90B1, HSP90AB1, DNAJC8, HSP90AA1, AHCY, HSPB1
Protein kinase a signaling	4.67	6.22 × 10^−2^		FLNB, HIST1H1C, MYH10, YWHAG, YWHAH, MAPK1, YWHAE, YWHAB, PDIA3, GNB2L1, YWHAZ, PYGL, PYGB, PDE6H, GNAI2, YWHAQ, H3F3A/H3F3B, FLNA, FLNC, RHOA, SFN, PPP1CA, APEX1, MYL12A
Sertoli cell-sertoli cell junction signaling	4.59	8.43 × 10^−2^		ITGB1, SPTBN1, TUBA1B, MAPK1, TUBB4B, CTNNA1, YBX3, TUBB, TUBB6, TUBB8, ACTN4, SPTAN1, ACTG1, ACTN1, ACTA1
Lipid antigen presentation by CD1	4.56	2.31 × 10^−1^		B2M, CALR, PDIA3, AP2B1, CANX, AP1B1
Superpathway of geranylgeranyldiphosphate biosynthesis I (via mevalonate)	4.43	2.94 × 10^−1^		FDPS, ACAT2, IDI1, ACAT1, HADHA
VEGF signaling	4.20	1.10 × 10^−1^	0.000	EIF2S2, YWHAE, MAPK1, VCL, ACTN4, EIF2S1, SFN, ACTG1, ACTA1, ACTN1
Fcγ receptor-mediated phagocytosis in macrophages and monocytes	4.12	1.08 × 10^−1^	−1.265	ACTR2, ACTR3, ARPC1B, MAPK1, ARPC2, EZR, RAB11A, TLN1, ACTG1, ACTA1
Tryptophan degradation III (eukaryotic)	4.06	2.50 × 10^−1^		HSD17B10, ACAT2, ACAT1, HADHA, HADH
Pentose phosphate pathway	4.03	3.64 × 10^−1^		PGD, TKT, TALDO1, G6PD
Nrf2-mediated oxidative stress response	3.95	7.78 × 10^−2^	−1.890	SOD1, MAPK1, PRDX1, DNAJA1, ERP29, DNAJC8, STIP1, VCP, CCT7, DNAJA2, SQSTM1, ACTG1, ACTA1, GSTP1
Purine nucleotides *de novo* biosynthesis II	3.86	3.33 × 10^−1^		GMPS, IMPDH2, PAICS, ATIC
Huntington’s disease signaling	3.86	6.96 × 10^−2^		SDHA, MAPK1, GNB2L1, HSPA9, CLTC, PSME2, HSPA5, HSPA8, HSPA4, DYNC1I2, PSME1, CAPNS1, ATP5B, CAPN1, CAPN2, DNM1L
Erk/MAPK signaling	3.77	7.49 × 10^−2^	−1.000	ITGB1, YWHAG, YWHAH, MAPK1, PPP2CA, YWHAB, YWHAZ, TLN1, YWHAQ, PPP2R1A, H3F3A/H3F3B, PPP1CA, STAT1, HSPB1
Rac signaling	3.71	9.62 × 10^−2^	−0.333	ITGB1, ACTR2, ACTR3, ARPC1B, MAPK1, CFL1, ARPC2, RHOA, CD44, IQGAP1
Mevalonate pathway I	3.71	3.08 × 10^−1^		ACAT2, IDI1, ACAT1, HADHA
Breast cancer regulation by stathmin1	3.68	7.33 × 10^−2^		TUBA1B, MAPK1, PPP2CA, TUBB4B, GNB2L1, TUBB, CDK1, GNAI2, STMN1, PPP2R1A, TUBB6, TUBB8, RHOA, PPP1CA
Antigen presentation pathway	3.65	1.62 × 10^−1^		B2M, CALR, HLA-A, PDIA3, HLA-B, CANX
FAK signaling	3.64	1.03 × 10^−1^		ITGB1, CAPNS1, MAPK1, CAPN1, TLN1, CAPN2, VCL, ACTG1, ACTA1
Leukocyte extravasation signaling	3.52	7.07 × 10^−2^	0.000	ITGB1, MAPK1, CTNNA1, GNAI2, EZR, RHOA, CD44, ACTN4, VCL, CTTN, ACTG1, ACTN1, ACTA1, MSN
Telomere extension by telomerase	3.45	2.67 × 10^−1^		HNRNPA1, XRCC6, HNRNPA2B1, XRCC5
Gap junction signaling	3.45	7.74 × 10^−2^		GNAI2, DBN1, TUBA1B, TUBB6, MAPK1, TUBB8, TUBB4B, PDIA3, CAV1, TUBB, ACTG1, ACTA1
Myc mediated apoptosis signaling	3.35	1.21 × 10^−1^		YWHAQ, YWHAG, YWHAE, YWHAH, YWHAB, YWHAZ, SFN
Superpathway of cholesterol biosynthesis	3.32	1.79 × 10^−1^		FDPS, ACAT2, IDI1, ACAT1, HADHA
IGF-1 signaling	3.28	9.28 × 10^−2^		YWHAQ, YWHAG, YWHAE, MAPK1, YWHAH, YWHAB, YWHAZ, SFN, CYR61
Spliceosomal cycle	3.24	1.00		LOC102724594/U2AF1, U2AF2
Tight junction signaling	3.16	7.19 × 10^−2^		MYH10, PPP2R1A, MYH9, PPP2CA, RHOA, VAPA, CTNNA1, YBX3, SPTAN1, VCL, ACTG1, ACTA1
Cdc42 signaling	3.16	7.19 × 10^−2^	−0.707	ITGB1, B2M, ACTR2, ACTR3, ARPC1B, MAPK1, CFL1, HLA-A, ARPC2, HLA-B, IQGAP1, MYL12A
Erk5 signaling	3.13	1.11 × 10^−1^		YWHAQ, YWHAG, YWHAE, YWHAH, YWHAB, YWHAZ, SFN
Paxillin signaling	3.12	8.82 × 10^−2^	−1.667	ITGB1, MAPK1, ARF1, TLN1, VCL, ACTN4, ACTG1, ACTA1, ACTN1
Mitochondrial dysfunction	3.07	7.02 × 10^−2^		HSD17B10, SDHA, ATP5H, ATP5B, PARK7, PRDX5, ATP5A1, CYC1, VDAC1, UQCRC1, CYB5R3, VDAC2
Hypoxia signaling in the cardiovascular system	3.05	1.08 × 10^−1^		P4HB, HSP90B1, UBE2L3, HSP90AB1, UBE2N, HSP90AA1, LDHA
Mitotic roles of polo-like kinase	3.01	1.06 × 10^−1^		HSP90B1, PPP2R1A, HSP90AB1, PPP2CA, CAPN1, HSP90AA1, CDK1
Sucrose degradation V (mammalian)	2.99	3.33 × 10^−1^		TPI1, ALDOA, ALDOC
Ketolysis	2.99	3.33 × 10^−1^		ACAT2, ACAT1, HADHA
CTLA4 signaling in cytotoxic T lymphocytes	2.92	9.09 × 10^−2^		B2M, PPP2R1A, PPP2CA, HLA-A, AP2B1, CLTC, HLA-B, AP1B1
Endoplasmic reticulum stress pathway	2.86	1.90 × 10^−1^		CALR, HSP90B1, EIF2S1, HSPA5
Ketogenesis	2.84	3.00 × 10^−1^		ACAT2, ACAT1, HADHA
Glycogen degradation II	2.84	3.00 × 10^−1^		PGM1, PYGB, PYGL
Inosine-5′-phosphate biosynthesis II	2.77	6.67 × 10^−1^		PAICS, ATIC
Ephrin b signaling	2.75	9.59 × 10^−2^	−0.447	GNAI2, MAPK1, CFL1, RHOA, GNB2L1, CAP1, HNRNPK
TCA cycle II (eukaryotic)	2.70	1.74 × 10^−1^		SDHA, CS, MDH1, MDH2
Actin nucleation by ARP-WASP complex	2.67	1.07 × 10^−1^	−0.816	ITGB1, ACTR2, ACTR3, ARPC1B, ARPC2, RHOA
Glycogen degradation III	2.60	2.50 × 10^−1^		PGM1, PYGB, PYGL
Telomerase signaling	2.59	8.08 × 10^−2^	0.000	HSP90B1, PPP2R1A, MAPK1, HSP90AB1, PPP2CA, HSP90AA1, TPP1, PTGES3
Cell cycle control of chromosomal replication	2.44	1.48 × 10^−1^		MCM3, MCM6, MCM4, MCM7
Axonal guidance signaling	2.40	4.62 × 10^−2^		DPYSL2, ITGB1, TUBA1B, ACTR2, PFN1, CFL1, MAPK1, ARPC1B, TUBB4B, PDIA3, GNB2L1, TUBB, GNAI2, ACTR3, TUBB6, TUBB8, ARPC2, RHOA, RTN4, MYL12A
DNA double-strand break repair by non-homologous end joining	2.39	2.14 × 10^−1^		PRKDC, XRCC6, XRCC5
Glucocorticoid receptor signaling	2.37	5.36 × 10^−2^		MAPK1, YWHAH, HSPA9, HSPA5, PTGES3, HSPA8, HMGB1, HSPA4, HSP90B1, HSP90AB1, ANXA1, FKBP4, HSP90AA1, STAT1
Fatty acid β-oxidation I	2.27	1.33 × 10^−1^		HSD17B10, ECHS1, HADHA, HADH
Pentose phosphate pathway (oxidative branch)	2.27	4.00 × 10^−1^		PGD, G6PD
Trans, *trans*-farnesyl diphosphate biosynthesis	2.27	4.00 × 10^−1^		FDPS, IDI1
Apoptosis signaling	2.26	7.87 × 10^−2^	1.134	CAPNS1, MAPK1, CAPN1, LMNA, CAPN2, SPTAN1, CDK1
Granzyme B signaling	2.22	1.88 × 10^−1^		PRKDC, NUMA1, LMNB1
Parkinson’s signaling	2.22	1.88 × 10^−1^		UCHL1, MAPK1, PARK7
Agrin interactions at neuromuscular junction	2.22	8.70 × 10^−2^	−1.342	ITGB1, MAPK1, LAMB1, CTTN, ACTG1, ACTA1
Aryl hydrocarbon receptor signaling	2.18	6.43 × 10^−2^	−0.378	HSP90B1, MAPK1, HSP90AB1, HSP90AA1, GSTP1, PTGES3, HSPB1, MCM7, AIP
Pyruvate fermentation to lactate	2.10	3.33 × 10^−1^		LDHA, LDHB
Pentose phosphate pathway (non-oxidative branch)	2.10	3.33 × 10^−1^		TKT, TALDO1
Semaphorin signaling in neurons	2.07	9.43 × 10^−2^		ITGB1, DPYSL2, MAPK1, CFL1, RHOA
Ephrin receptor signaling	2.04	5.75 × 10^−2^		ITGB1, GNAI2, ACTR2, ACTR3, ARPC1B, MAPK1, CFL1, ARPC2, RHOA, GNB2L1
CDK5 signaling	2.02	7.07 × 10^−2^	0.000	ITGB1, PPP2R1A, MAPK1, PPP2CA, CAPN1, LAMB1, PPP1CA
Superpathway of serine and glycine biosynthesisI	1.96	2.86 × 10^−1^		PHGDH, SHMT2
Aspartate degradation II	1.96	2.86 × 10^−1^		MDH1, MDH2
Granzyme A signaling	1.94	1.50 × 10^−1^		HIST1H1C, SET, APEX1
Prostate cancer signaling	1.86	7.32 × 10^−2^		HSP90B1, MAPK1, PA2G4, HSP90AB1, HSP90AA1, GSTP1
fMLP signaling in neutrophils	1.82	6.48 × 10^−2^	0.000	GNAI2, ACTR2, ACTR3, ARPC1B, MAPK1, ARPC2, GNB2L1
Mechanisms of viral exit from host cells	1.79	9.76 × 10^−2^		XPO1, LMNB1, ACTG1, ACTA1
UDP-*N*-acetyl-d-galactosamine biosynthesis II	1.74	2.22 × 10^−1^		GPI, UAP1
eNOS signaling	1.70	5.67 × 10^−2^	0.000	HSPA8, HSPA4, HSP90B1, HSP90AB1, HSPA9, CAV1, HSP90AA1, HSPA5
Acetyl-CoA biosynthesis III (from citrate)	1.62	1.00		ACLY
Ber pathway	1.49	1.67 × 10^−1^		PCNA, APEX1
Amyloid processing	1.48	7.84 × 10^−2^	0.000	CAPNS1, MAPK1, CAPN1, CAPN2
Agranulocyte adhesion and diapedesis	1.42	4.76 × 10^−2^		ITGB1, GNAI2, MYH10, FN1, MYH9, EZR, ACTG1, ACTA1, MSN
Cytotoxic T lymphocyte-mediated apoptosis of target cells	1.40	9.38 × 10^−2^		B2M, HLA-A, HLA-B
Role of CHK proteins in cell cycle checkpoint control	1.37	7.27 × 10^−2^	0.000	PCNA, PPP2R1A, PPP2CA, CDK1
Oxidative phosphorylation	1.33	5.50 × 10^−2^		SDHA, ATP5H, ATP5B, ATP5A1, CYC1, UQCRC1
Palmitate biosynthesis I (animals)	1.33	5.00 × 10^−1^		FASN
Fatty acid biosynthesis initiation II	1.33	5.00 × 10^−1^		FASN
Formaldehyde oxidation II (glutathione-dependent)	1.33	5.00 × 10^−1^		ESD
Glycine biosynthesis I	1.33	5.00 × 10^−1^		SHMT2
Glutamate biosynthesis II	1.33	5.00 × 10^−1^		GLUD1
Glutamate degradation X	1.33	5.00 × 10^−1^		GLUD1
Chondroitin sulfate degradation (metazoa)	1.31	1.33 × 10^−1^		CD44, HEXB
Androgen signaling	1.30	5.41 × 10^−2^		GNAI2, HSPA4, CALR, MAPK1, GNB2L1, HSP90AA1
Dermatan sulfate degradation (metazoa)	1.26	1.25 × 10^−1^		CD44, HEXB
α-Adrenergic signaling	1.24	5.75 × 10^−2^	−1.000	GNAI2, MAPK1, GNB2L1, PYGB, PYGL
Neuregulin signaling	1.22	5.68 × 10^−2^		ITGB1, HSP90B1, MAPK1, HSP90AB1, HSP90AA1
Methionine degradation I (to homocysteine)	1.21	1.18 × 10^−1^		PRMT5, AHCY
Crosstalk between dendritic cells and natural killer cells	1.21	5.62 × 10^−2^		HLA-A, HLA-B, TLN1, ACTG1, ACTA1
Calcium signaling	1.19	4.49 × 10^−2^		MYH10, CALR, MYH9, MAPK1, TPM3, TPM4, TPM2, ACTA1
PPARα/RXRα activation	1.18	4.47 × 10^−2^	0.378	CAND1, HSP90B1, MAPK1, HSP90AB1, PDIA3, FASN, HSP90AA1, AIP
Valine degradation I	1.17	1.11 × 10^−1^		ECHS1, HADHA
Death receptor signaling	1.16	5.43 × 10^−2^	1.342	LMNA, SPTAN1, ACTG1, ACTA1, HSPB1
Neuroprotective role of THOP1 in Alzheimer’s disease	1.16	7.50 × 10^−2^		YWHAE, HLA-A, HLA-B
Diphthamide biosynthesis	1.16	3.33 × 10^−1^		EEF2
NADH repair	1.16	3.33 × 10^−1^		GAPDH
Methylglyoxal degradation I	1.16	3.33 × 10^−1^		GLO1
Hypusine biosynthesis	1.16	3.33 × 10^−1^		EIF5A
Oxidized GTP and DGTP detoxification	1.16	3.33 × 10^−1^		DDX6
Gadd45 signaling	1.13	1.05 × 10^−1^		PCNA, CDK1
DNA damage-induced 14-3-3σ signaling	1.13	1.05 × 10^−1^		SFN, CDK1
Cysteine biosynthesis III (mammalia)	1.13	1.05 × 10^−1^		PRMT5, AHCY
PPAR signaling	1.13	5.32 × 10^−2^	0.447	HSP90B1, MAPK1, HSP90AB1, HSP90AA1, AIP
Tec kinase signaling	1.07	4.43 × 10^−2^	−1.342	ITGB1, GNAI2, RHOA, GNB2L1, STAT1, ACTG1, ACTA1
Nitric oxide signaling in the cardiovascular system	1.05	5.05 × 10^−2^	0.447	HSP90B1, MAPK1, HSP90AB1, CAV1, HSP90AA1
Cellular effects of sildenafil (viagra)	1.05	4.65 × 10^−2^		MYH10, MYH9, PDIA3, ACTG1, ACTA1, MYL12A
Chemokine signaling	1.05	5.63 × 10^−2^	0.000	GNAI2, MAPK1, CFL1, RHOA
Uracil degradation II (reductive)	1.04	2.50 × 10^−1^		DPYSL2
Thymine degradation	1.04	2.50 × 10^−1^		DPYSL2
Geranylgeranyldiphosphate biosynthesis	1.04	2.50 × 10^−1^		FDPS
Rapoport-luebering glycolytic shunt	1.04	2.50 × 10^−1^		PGAM1
Polyamine regulation in colon cancer	1.02	9.09 × 10^−2^		PSME1, PSME2
HIF1α signaling	1.01	4.90 × 10^−2^		MAPK1, HSP90AA1, LDHA, APEX1, LDHB
Cardiac β-adrenergic signaling	1.00	4.51 × 10^−2^	−0.447	PPP2R1A, PPP2CA, GNB2L1, PPP1CA, APEX1, PDE6H
nNOS signaling in neurons	9.92 × 10^−1^	6.38 × 10^−2^		CAPNS1, CAPN1, CAPN2
AMPK signaling	9.90 × 10^−1^	4.48 × 10^−2^	−0.447	PPP2R1A, MAPK1, PPP2CA, FASN, PFKP, PPM1G
IL-22 signaling	9.53 × 10^−1^	8.33 × 10^−2^		MAPK1, STAT1
Serine biosynthesis	9.44 × 10^−1^	2.00 × 10^−1^		PHGDH
DTMP de novo biosynthesis	9.44 × 10^−1^	2.00 × 10^−1^		SHMT2
Folate polyglutamylation	9.44 × 10^−1^	2.00 × 10^−1^		SHMT2
Xenobiotic metabolism signaling	9.35 × 10^−1^	3.69 × 10^−2^		HSP90B1, PPP2R1A, MAPK1, HSP90AB1, PPP2CA, HSP90AA1, GSTP1, PTGES3, ESD, AIP
Cyclins and cell cycle regulation	9.34 × 10^−1^	5.13 × 10^−2^		PPP2R1A, PA2G4, PPP2CA, CDK1
Role of JAK family kinases in IL-6-type cytokine signaling	9.24 × 10^−1^	8.00 × 10^−2^		MAPK1, STAT1
Type I diabetes mellitus signaling	9.08 × 10^−1^	4.55 × 10^−2^		MAPK1, HLA-A, HLA-B, HSPD1, STAT1
Role of tissue factor in cancer	9.08 × 10^−1^	4.55 × 10^−2^		ITGB1, P4HB, MAPK1, CFL1, CYR61
Antiproliferative role of Tob in T cell signaling	8.95 × 10^−1^	7.69 × 10^−2^		PABPC1, MAPK1
Arginine biosynthesis IV	8.70 × 10^−1^	1.67 × 10^−1^		GLUD1
Superoxide radicals degradation	8.70 × 10^−1^	1.67 × 10^−1^		SOD1
UDP-*N*-acetyl-d-glucosamine biosynthesis II	8.70 × 10^−1^	1.67 × 10^−1^		UAP1
GDP-mannose biosynthesis	8.70 × 10^−1^	1.67 × 10^−1^		GPI
IL-15 production	8.69 × 10^−1^	7.41 × 10^−2^		TWF1, STAT1
CCR3 signaling in eosinophils	8.28 × 10^−1^	4.27 × 10^−2^		GNAI2, MAPK1, CFL1, RHOA, GNB2L1
IL-8 signaling	8.25 × 10^−1^	3.83 × 10^−2^	−0.816	GNAI2, MAPK1, RHOA, GNB2L1, IQGAP1, LASP1, CSTB
Glioma invasiveness signaling	8.09 × 10^−1^	5.26 × 10^−2^		MAPK1, RHOA, CD44
Acetyl-CoA biosynthesis I (pyruvate dehydrogenase complex)	8.08 × 10^−1^	1.43 × 10^−1^		DLAT
GDP-glucose biosynthesis	8.08 × 10^−1^	1.43 × 10^−1^		PGM1
G β γ signaling	7.98 × 10^−1^	4.55 × 10^−2^		GNAI2, MAPK1, GNB2L1, CAV1
Role of p14/p19arf in tumor suppression	7.95 × 10^−1^	6.67 × 10^−2^		NPM1, SF3A1
PAK signaling	7.86 × 10^−1^	4.49 × 10^−2^		ITGB1, MAPK1, CFL1, MYL12A
Induction of apoptosis by HIV1	7.63 × 10^−1^	5.00 × 10^−2^		SLC25A6, SLC25A3, SLC25A5
Glucose and glucose-1-phosphate degradation	7.55 × 10^−1^	1.25 × 10^−1^		PGM1
Superpathway of methionine degradation	7.51 × 10^−1^	6.25 × 10^−2^		PRMT5, AHCY
GM-CSFsignaling	7.34 × 10^−1^	4.84 × 10^−2^		MAPK1, GNB2L1, STAT1
Estrogen receptor signaling	7.26 × 10^−1^	3.94 × 10^−2^		PRKDC, DDX5, H3F3A/H3F3B, MAPK1, PHB2
PCP pathway	7.20 × 10^−1^	4.76 × 10^−2^		PFN1, RHOA, HSPB1
Oncostatin m signaling	7.11 × 10^−1^	5.88 × 10^−2^		MAPK1, STAT1
Interferon signaling	7.11 × 10^−1^	5.88 × 10^−2^		IFITM3, STAT1
Prostanoid biosynthesis	7.09 × 10^−1^	1.11 × 10^−1^		PTGES3
Folate transformations I	7.09 × 10^−1^	1.11 × 10^−1^		SHMT2
Pyridoxal 5′-phosphate salvage pathway	7.06 × 10^−1^	4.69 × 10^−2^		PDXK, MAPK1, CDK1
Cell cycle regulation by btg family proteins	6.92 × 10^−1^	5.71 × 10^−2^		PPP2R1A, PPP2CA
Trna splicing	6.92 × 10^−1^	5.71 × 10^−2^		APEX1, PDE6H
Amyotrophic lateral sclerosis signaling	6.84 × 10^−1^	4.08 × 10^−2^		SOD1, CAPNS1, CAPN1, CAPN2
p53 signaling	6.84 × 10^−1^	4.08 × 10^−2^		PRKDC, PCNA, SFN, GML
Role of PI3K/Akt signaling in the pathogenesis of influenza	6.80 × 10^−1^	4.55 × 10^−2^		GNAI2, KPNA3, MAPK1
Phospholipase c signaling	6.70 × 10^−1^	3.35 × 10^−2^	−1.633	ITGB1, PEBP1, MARCKS, AHNAK, MAPK1, RHOA, GNB2L1, MYL12A
Glycine βine degradation	6.68 × 10^−1^	1.00 × 10^−1^		SHMT2
Complement system	6.56 × 10^−1^	5.41 × 10^−2^		CD59, C1QBP
Macropinocytosis signaling	6.55 × 10^−1^	4.41 × 10^−2^		ITGB1, RHOA, ACTN4
Relaxin signaling	6.54 × 10^−1^	3.70 × 10^−2^		GNAI2, MAPK1, GNB2L1, APEX1, PDE6H
CCR5 signaling in macrophages	6.42 × 10^−1^	4.35 × 10^−2^		GNAI2, MAPK1, GNB2L1
Melatonin signaling	6.30 × 10^−1^	4.29 × 10^−2^		GNAI2, MAPK1, PDIA3
Role of PKR in interferon induction and antiviral response	6.06 × 10^−1^	5.00 × 10^−2^		STAT1, EIF2S1
Synaptic long term depression	6.06 × 10^−1^	3.55 × 10^−2^	0.447	GNAI2, PPP2R1A, MAPK1, PPP2CA, PDIA3
Dendritic cell maturation	5.90 × 10^−1^	3.35 × 10^−2^		B2M, MAPK1, HLA-A, PDIA3, HLA-B, STAT1
Production of nitric oxide and reactive oxygen species in macrophages	5.83 × 10^−1^	3.33 × 10^−2^	−0.816	PPP2R1A, MAPK1, PPP2CA, RHOA, STAT1, PPP1CA
Sphingosine-1-phosphate signaling	5.79 × 10^−1^	3.67 × 10^−2^	0.000	GNAI2, MAPK1, PDIA3, RHOA
Assembly of rna polymerase III complex	5.69 × 10^−1^	7.69 × 10^−2^		SF3A1
Systemic lupus erythematosus signaling	5.62 × 10^−1^	3.18 × 10^−2^		PRPF19, MAPK1, HLA-A, HNRNPA2B1, HLA-B, SNRPD1, SNRPD3
PDGF signaling	5.54 × 10^−1^	3.90 × 10^−2^		MAPK1, CAV1, STAT1
iNOS signaling	5.48 × 10^−1^	4.55 × 10^−2^		MAPK1, STAT1
Cardiac hypertrophy signaling	5.45 × 10^−1^	3.14 × 10^−2^	−0.816	GNAI2, MAPK1, PDIA3, RHOA, GNB2L1, HSPB1, MYL12A
Dopamine receptor signaling	5.44 × 10^−1^	3.85 × 10^−2^		PPP2R1A, PPP2CA, PPP1CA
Urate biosynthesis/inosine 5′-phosphate degradation	5.42 × 10^−1^	7.14 × 10^−2^		IMPDH2
Colanic acid building blocks biosynthesis	5.42 × 10^−1^	7.14 × 10^−2^		GPI
CXCR4 signaling	5.25 × 10^−1^	3.29 × 10^−2^	0.000	GNAI2, MAPK1, RHOA, GNB2L1, MYL12A
MSP-RON signaling pathway	5.21 × 10^−1^	4.35 × 10^−2^		ACTG1, ACTA1
Methylglyoxal degradation III	5.17 × 10^−1^	6.67 × 10^−2^		AKR1B1
Thrombin signaling	5.14 × 10^−1^	3.14 × 10^−2^	0.447	GNAI2, MAPK1, PDIA3, RHOA, GNB2L1, MYL12A
CD28 signaling in Thelper cells	5.06 × 10^−1^	3.39 × 10^−2^	0.000	ACTR2, ACTR3, ARPC1B, ARPC2
PTEN signaling	5.06 × 10^−1^	3.39 × 10^−2^		ITGB1, MAPK1, YWHAH, IGF2R
P2Y purigenic receptor signaling pathway	4.99 × 10^−1^	3.36 × 10^−2^		GNAI2, MAPK1, PDIA3, GNB2L1
Graft-versus-host disease signaling	4.97 × 10^−1^	4.17 × 10^−2^		HLA-A, HLA-B
Ephrin a signaling	4.97 × 10^−1^	4.17 × 10^−2^		CFL1, RHOA
Mismatch repair in eukaryotes	4.94 × 10^−1^	6.25 × 10^−2^		PCNA
G_αi_ signaling	4.92 × 10^−1^	3.33 × 10^−2^		GNAI2, MAPK1, GNB2L1, CAV1
Autoimmune thyroid disease signaling	4.85 × 10^−1^	4.08 × 10^−2^		HLA-A, HLA-B
TR/RXR activation	4.79 × 10^−1^	3.53 × 10^−2^		ENO1, FASN, PFKP
Γ-linolenate biosynthesis II (animals)	4.72 × 10^−1^	5.88 × 10^−2^		CYB5R3
Allograft rejection signaling	4.71 × 10^−1^	3.49 × 10^−2^		B2M, HLA-A, HLA-B
Dopamine-DARPP32 feedback in camp signaling	4.68 × 10^−1^	3.11 × 10^−2^		GNAI2, PPP2R1A, PPP2CA, PDIA3, PPP1CA
UVa-induced MAPK signaling	4.54 × 10^−1^	3.41 × 10^−2^		MAPK1, PDIA3, STAT1
CNTF signaling	4.51 × 10^−1^	3.85 × 10^−2^		MAPK1, STAT1
Endometrial cancer signaling	4.51 × 10^−1^	3.85 × 10^−2^		MAPK1, CTNNA1
OX40 signaling pathway	4.46 × 10^−1^	3.37 × 10^−2^		B2M, HLA-A, HLA-B
UVb-induced MAPK signaling	4.41 × 10^−1^	3.77 × 10^−2^		H3F3A/H3F3B, MAPK1
Communication between innate and adaptive immune cells	4.31 × 10^−1^	3.30 × 10^−2^		B2M, HLA-A, HLA-B
IL-1 signaling	4.31 × 10^−1^	3.30 × 10^−2^		GNAI2, MAPK1, GNB2L1
Thrombopoietin signaling	4.21 × 10^−1^	3.64 × 10^−2^		MAPK1, STAT1
Purine nucleotides degradation II (aerobic)	4.16 × 10^−1^	5.00 × 10^−2^		IMPDH2
Wnt/Ca^++^ pathway	4.11 × 10^−1^	3.57 × 10^−2^		PDIA3, PDE6H
EGF signaling	4.11 × 10^−1^	3.57 × 10^−2^		MAPK1, STAT1
Glioma signaling	4.01 × 10^−1^	3.16 × 10^−2^		MAPK1, PA2G4, IGF2R
Maturity onset diabetes of young (mody) signaling	3.84 × 10^−1^	4.55 × 10^−2^		GAPDH
ATM signaling	3.84 × 10^−1^	3.39 × 10^−2^		TRIM28, CDK1
Granulocyte adhesion and diapedesis	3.81 × 10^−1^	2.82 × 10^−2^		ITGB1, GNAI2, EZR, HSPB1, MSN
Estrogen-dependent breast cancer signaling	3.59 × 10^−1^	3.23 × 10^−2^		HSD17B10, MAPK1
Role of JAK1, JAK2 and TYK2 in interferon signaling	3.56 × 10^−1^	4.17 × 10^−2^		STAT1
Estrogen-mediated s-phase entry	3.56 × 10^−1^	4.17 × 10^−2^		CDK1
Antiproliferative role of somatostatin receptor 2	3.51 × 10^−1^	3.17 × 10^−2^		MAPK1, GNB2L1
Role of JAK1 and JAK3 in γc cytokine signaling	3.51 × 10^−1^	3.17 × 10^−2^		MAPK1, STAT1
Role of lipids/lipid rafts in the pathogenesis of influenza	3.43 × 10^−1^	4.00 × 10^−2^		FDPS
IL-17a signaling in gastric cells	3.43 × 10^−1^	4.00 × 10^−2^		MAPK1
Cell cycle: G_1_/S checkpoint regulation	3.43 × 10^−1^	3.12 × 10^−2^		PA2G4, RPL5
Non-small cell lung cancer signaling	3.35 × 10^−1^	3.08 × 10^−2^		MAPK1, PA2G4
Pancreatic adenocarcinoma signaling	3.31 × 10^−1^	2.83 × 10^−2^		MAPK1, PA2G4, STAT1
GABA receptor signaling	3.21 × 10^−1^	2.99 × 10^−2^		AP2B1, AP1B1
Pyrimidine ribonucleotides interconversion	3.19 × 10^−1^	3.70 × 10^−2^		CTPS1
Growth hormone signaling	3.07 × 10^−1^	2.90 × 10^−2^		MAPK1, STAT1
Corticotropin releasing hormone signaling	3.04 × 10^−1^	2.70 × 10^−2^		GNAI2, MAPK1, KRT1
Pyrimidine ribonucleotides *de novo* biosynthesis	2.97 × 10^−1^	3.45 × 10^−2^		CTPS1
IL-3 signaling	2.94 × 10^−1^	2.82 × 10^−2^		MAPK1, STAT1
PEDF signaling	2.94 × 10^−1^	2.82 × 10^−2^		MAPK1, RHOA
GPCR-mediated integration of enteroendocrine signaling exemplified by an L cell	2.94 × 10^−1^	2.82 × 10^−2^		GNAI2, PDIA3
JAK/STAT signaling	2.87 × 10^−1^	2.78 × 10^−2^		MAPK1, STAT1
Glutathione-mediated detoxification	2.87 × 10^−1^	3.33 × 10^−2^		GSTP1
Sonic hedgehog signaling	2.87 × 10^−1^	3.33 × 10^−2^		CDK1
Hereditary breast cancer signaling	2.84 × 10^−1^	2.61 × 10^−2^		NPM1, SFN, CDK1
NF-κB activation by viruses	2.81 × 10^−1^	2.74 × 10^−2^		ITGB1, MAPK1
Prolactin signaling	2.81 × 10^−1^	2.74 × 10^−2^		MAPK1, STAT1
STAT3 pathway	2.81 × 10^−1^	2.74 × 10^−2^		MAPK1, IGF2R
4-1BB signaling in T lymphocytes	2.78 × 10^−1^	3.23 × 10^−2^		MAPK1
Flt3 signaling in hematopoietic progenitor cells	2.75 × 10^−1^	2.70 × 10^−2^		MAPK1, STAT1
Leptin signaling in obesity	2.75 × 10^−1^	2.70 × 10^−2^		MAPK1, PDIA3
G_α12/13_ signaling	2.74 × 10^−1^	2.56 × 10^−2^		MAPK1, RHOA, MYL12A
p38 MAPK signaling	2.74 × 10^−1^	2.56 × 10^−2^		H3F3A/H3F3B, STAT1, HSPB1
TREM1 signaling	2.69 × 10^−1^	2.67 × 10^−2^		ITGB1, MAPK1
Synaptic long term potentiation	2.65 × 10^−1^	2.52 × 10^−2^		MAPK1, PDIA3, PPP1CA
HMGB1 signaling	2.60 × 10^−1^	2.50 × 10^−2^		HMGB1, MAPK1, RHOA
G protein signaling mediated by tubby	2.60 × 10^−1^	3.03 × 10^−2^		GNB2L1
MIF-mediated glucocorticoid regulation	2.60 × 10^−1^	3.03 × 10^−2^		MAPK1
Retinol biosynthesis	2.60 × 10^−1^	3.03 × 10^−2^		ESD
Ethanol degradation ii	2.60 × 10^−1^	3.03 × 10^−2^		HSD17B10
LXR/RXR activation	2.56 × 10^−1^	2.48 × 10^−2^		ECHS1, FASN, HADH
IL-9 signaling	2.51 × 10^−1^	2.94 × 10^−2^		STAT1
Inhibition of angiogenesis by TSP1	2.51 × 10^−1^	2.94 × 10^−2^		MAPK1
Reelin signaling in neurons	2.47 × 10^−1^	2.53 × 10^−2^		ITGB1, PAFAH1B2
IL-17a signaling in fibroblasts	2.43 × 10^−1^	2.86 × 10^−2^		MAPK1
Role of JAK2 in hormone-like cytokine signaling	2.43 × 10^−1^	2.86 × 10^−2^		STAT1
Stearate biosynthesis I (animals)	2.43 × 10^−1^	2.86 × 10^−2^		FASN
Noradrenaline and adrenaline degradation	2.43 × 10^−1^	2.86 × 10^−2^		HSD17B10
Nucleotide excision repair pathway	2.43 × 10^−1^	2.86 × 10^−2^		RAD23B
Ceramide signaling	2.42 × 10^−1^	2.50 × 10^−2^		PPP2R1A, PPP2CA
Estrogen biosynthesis	2.28 × 10^−1^	2.70 × 10^−2^		HSD17B10
April mediated signaling	2.21 × 10^−1^	2.63 × 10^−2^		MAPK1
Netrin signaling	2.14 × 10^−1^	2.56 × 10^−2^		ENAH
B cell activating factor signaling	2.07 × 10^−1^	2.50 × 10^−2^		MAPK1
Thyroid cancer signaling	2.07 × 10^−1^	2.50 × 10^−2^		MAPK1
Transcriptional regulatory network in embryonic stem cells	2.07 × 10^−1^	2.50 × 10^−2^		SET
MIF regulation of innate immunity	2.01 × 10^−1^	2.44 × 10^−2^		MAPK1

^a^ The *p* value of the protein is computed by the permutation test. ^b^ The average ratio is for all peptides associated with the protein (*i.e.*, log_2_ L/H). ^c^ *z*-Score is the measure of how many standard deviation units that protein’s log_2_ L/H ratio is away from its population mean.

**Table 4:** 24 networks of molecular signaling pathway regulated by vancomycin in HK-2 cells.

ID	Molecules in Network	Score	Molecules	Top Diseases and Functions
1	ALDO, ALDOA, ALDOC, ATP synthase, C14orf166, DDX1, ENO1, ENO2, enolase, FUS, HIST2H2AC, HNRNPU, NAP1L4, NCL, NONO, PA2G4, PGK1, Ras, RPL5, RPL6, RPL7, RPL8, RPL12, RPL18, RPL22, RPL23, RPL10A, RPL7A, RPLP1, RPLP2, SFPQ, SQSTM1, SYNCRIP, T3-TR-RXR, TARS	47	30	RNA post-transcriptional modification, carbohydrate metabolism, small molecule biochemistry
2	14-3-3, 14-3-3 (β, ε, ζ), 14-3-3(β, γ, θ, η, ζ), 14-3-3(η, θ, ζ), adenosine-tetraphosphatase, ATP5A1, ATP5B, C1QBP, CAPZB, CLTC, dishevelled, DYNC1H1, EPRS, ESYT1, GNAI2, GNB2L1, GSK3, HSP90AA1, HSPA9, KIF5B, MARS, PDCD6, RNH1, SFN, SLC25A3, SLC25A5, SLC25A6, TPI1, TUFM, YWHAB, YWHAE, YWHAG, YWHAH, YWHAQ, YWHAZ	42	28	Protein trafficking, cell death and survival, nucleic acid metabolism
3	60S ribosomal subunit, CAND1, CLIC1, EIF2, ERK, HNRNPH3, IPO9, Ku, LAMB1, laminin1, laminin2, NPM1, PDXK, PYGB, ribosomal 40s subunit, RNR, RPL3, RPL4, RPL10, RPL21, RPL27, RPLP0, RPS2, RPS3, RPS5, RPS7, RPS8, RPS10, RPS11, RPS12, RPS15, RPS24, RPS27A, RPS4X, RPSA	40	27	Hematological disease, organismal injury and abnormalities, RNA post-transcriptional modification
4	α-Tubulin, Ant, ATP5H, β-tubulin, CCT2, CCT3, CCT4, CCT5, CCT7, CCT8, CCT6A, DPYSL2, dynein, EHD1, ENAH, ERP29, GCN1L1, integrin α5 β1, MAP1B, MAPK1, NUMA1, PPP2CA, PPP2R1A, TCP1, TUBA1B, TUBB6, TUBB8, TUBB4B, tubulin (complex), tubulin (family), VAPA, VDAC, VDAC1, VDAC2, ZYX	40	27	Cellular assembly and organization, cell-to-cell signaling and interaction, reproductive system development and function
5	ACLY, aconitase, Akt, angiotensin II receptor type 1, Arf, ARF1, CNN3, COP I, COPA, COPB1, COPB2, COPE, COPG1, CS, EIF3, EIF6, EIF3C, EIF3M, HN1, LRRC59, malate dehydrogenase, MAP4, MDH1, MDH2, N-cadherin, PARK7, RAB1B, SEPT2, SEPT7, SEPT9, Septin, SHMT2, TALDO1, TXNDC5, WARS	38	26	Infectious disease, small molecule biochemistry, reproductive system disease
6	19S proteasome, 20S proteasome, 26S proteasome, BUB3, CPNE1, ECHS1, immunoproteasome Pa28/20s, KPNA3, MHC CLASS I (family), NF-kB (complex), NPLOC4, NSFL1C, OTUB1, PDLIM1, PRDX2, Proteasome PA700/20s, PSMA, PSMA1, PSMA2, PSMA4, PSMA5, PSMA7, PSMB1, PSMC1, PSMC4, PSMD2, PSME1, PSME2, RAD23B, RPN2, UBA1, UBE2, UBE2L3, UBE2N, ubiquitin	36	25	Infectious disease, organismal injury and abnormalities, immunological disease
7	AHCY, calpain, CYR61, DDX6, DDX3X, EIF2S2, EIF3B, EIF3I, EIF4A, EIF4A1, EIF4F, EIF4G, EIF4G1, EIF4H, fascin, filamin, FLNA, FLNB, FLNC, G3BP1, HNRNPA1, HNRNPL, ILF2, ILF3, LFA-1, MIRLET7, OLA1, PABPC1, PCBP1, PCBP2, PKC(s), PUF60, SAPK, SNX3, VPS35	36	25	Protein synthesis, cellular movement, gene expression
8	CALU, CBX3, CD59, CDK1, DBI, DDX5, DNA-methyltransferase, DPY30, GML, GSTP1, H3F3A/H3F3B, histone H3, histone H4, HNRNPA3, HNRNPK, HSP70, IFITM3, IgA, MATR3, NAP1L1, NASP, NOS, PKM, PPM1G, PRMT5, secretase γ, SH3BGRL3, Sod, SOD1, SRC (family), TFRC, thymidine kinase, TRIM28, UBXN4, YBX1	36	25	Cellular growth and proliferation, infectious disease, cellular development
9	APEX1, c-Src, CLIC4, CSE1L, H2AFY, HDGF, HIST1H1C, histone H1, HNRNP H, HNRNPH1, HNRNPM, importin α, importin β, integrin α3β1, IPO5, IPO7, karyopherin β, KPNA2, KPNB1, LOC102724594/U2AF1, PHGDH, PRPF19, PTMA, RAN, RANBP1, SAE1, SF3B1, snRNP, SNRPD1, SNRPD3, TNPO1, transportin, U2AF, U2AF2, VEGF	34	24	Molecular transport, protein trafficking, RNA post-transcriptional modification
10	Adaptor protein 1, AIP, AP1γ, AP1B1, CALR, CAP1, CD1, CD1D-CANX-CALR-ERp57, CFL1, CRIP2, cytoplasmic dynein, DNAJ, DNAJA1, DNAJA2, DNAJC8, FKBP4, G-actin, HSP, HSP90, HSP22/HSP40/HSP90, HSP90B1, HYOU1, JNK, P4HB, PCMT1, PDIA3, PDIA4, PDIA6, PRDX4, PTGES3, PYGL, STIP1, TMSB4, TPM2, TPM3	32	23	Post-translational modification, protein folding, drug metabolism
11	ACTG1, ADRB, AP2B1, ATPase, CAPZA1, casein, caspase, creatine kinase, DLAT, DYNC1I2, EEF2, EEF1A1, HSP90AB1, HSPA4, HSPA5, HSPA8, HSPB1, HSPD1, IDI1, IFNβ, LONP1, mediator, MHC Class II (complex), MMP, NACA, PHB2, PPA1, PPP2C, PRKDC, RPN1, RSK, STMN1, TLR, TXLNA, VCP	32	23	Post-translational modification, protein folding, cell morphology
12	Actin, ACTN1, ACTN4, α-actinin, α-catenin, ANXA6, ARP2/3, cadherin, CAPG, CAPN2, Cdc2, CSRP1, CTNNA1, cytokeratin, F actin, GPI, IMPDH2, integrinβ, KRT1, KRT8, KRT9, KRT18, lamin b, LMNB1, PI3K (complex), PLEC, spectrin, SPTAN1, SPTBN1, talin, TLN1, TPM4, TUBB, VCL, VIM	30	22	Cellular compromise, cellular assembly and organization, cellular function and maintenance
13	ACTR2, ACTR3, α-actin, ARHGDIA, ARPC2, ARPC1B, BCR (complex), CD44, CTTN, ERM, EZR, GDI1, GDI2, IQGAP1, MAPK, MIR124, MSN, PAFAH1B2, PAK, PLIN3, PLS3, RAB5, RAB11, RAB11A, RAB7A, Rac, Ras homolog, Rho GDI, RTN4, SEC13, Sos, TMOD3, TWF2, VAT1, VAV	30	22	Cellular assembly and organization, cellular function and maintenance, cell morphology
14	3-Hydroxyacyl-CoA dehydrogenase, ACAT1, ACAT2, acetyl-CoA C-acetyltransferase, AHNAK, AKR1B1, ANXA1, ANXA2, calmodulin, CAPN1, CAPNS1, CAST, CAV1, caveolin, CKAP4, CYC1, cytochrome bc1, cytochrome C, cytochrome-c oxidase, DNM1L, dynamin, fibrinogen, GAPDH, GLUD1, HADH, HADHA, HSD17B10, IMMT, integrin, MVP, PARP, Smad, tyrosine kinase, UQCRC1, VLDL-cholesterol	28	21	Endocrine system development and function, lipid metabolism, small molecule biochemistry
15	AARS, AHSA1, B2M, B2m-MHC1a, CACYBP, CANX, CAPRIN1, chymotrypsin, EEF1B2, EEF1D, EEF1G, ENaC, ERK1/2, ESD, GARS, GC-GCR dimer, GMPS, HARS, HLA Class I, HLA-A, HLA-abc, HLA-B, HLA-B27, KIR, MHC Class I (complex), MHC I-α, Na^+^/K^+^-ATPase, NPEPPS, PDE6H, PDGF (family), PKI, RPS3A, TAP, TBCA, VARS	26	20	Post-translational modification, protein folding, dermatological diseases and conditions
16	BTF3, Cbp/p300, CDK1/2, cyclin A, cyclin D, cyclin E, DHX9, E2f, Gap, HDLBP, Holo RNA polymerase II, MCM, MCM3, MCM4, MCM6, MCM7, PCM1, PCNA, PDE, Raf, Rb, RHOA, RPA, S100A6, SERBP1, SND1, SSRP1, SUPT16H, TAGLN, TIP60, TMPO, tropomyosin, XRCC5, XRCC6, YBX3	26	20	DNA replication, recombination, and repair, cell morphology, cellular function and maintenance
17	ACTA1, BASP1, CaMKII, cofilin, CORO1C, COTL1, CTPS1, DBN1, FKBP1A, GLO1, glycogen phosphorylase, ITPR, LDH (complex), LDHA, LDHB, MARCKS, MEF2, Mlc, MYH9, MYH10, MYL12A, myosin2, PFN1, PICALM, PKA, PP1 protein complex group, PP2A, profilin, PTRF, RAP1, RBM3, RNA polymerase I, Rock, voltage-gated calcium channel, WDR1	24	19	Cellular movement, connective tissue disorders, hematological disease
18	ALYREF, ATP1A1, BSG, calcineurin A, calcineurin protein(s), CK2, DDX39B, EIF2S1, EIF5A, ETFA, FCER1, focal adhesion kinase, HEXB, HINT1, IgE, IGF2R, Ikb, JINK1/2, MAP2K1/2, mitochondrial complex 1, NFAT (complex), NFAT (family), NF-kB (family), peptidylprolyl isomerase, phosphatase, PLCγ, PP1-C, PP1/PP2A, PPIA, PPP1CA, RCN1, SET, STOML2, VLA-4, XPO1	19	16	Molecular transport, RNA trafficking, cell death and survival
19	CARHSP1, CDV3, CECR2, CLDN12, CTSA, CUTA, CYB5R3, DMXL2, FADS1, FAM57A, FBXO11, HEBP2, HM13, KHSRP, LRPPRC, NEU1, NME1-NME2, NNMT, PARN, PFKP, PGM1, POLRMT, SDHA, SDHAF2, SDHC, SIRT5, SLC25A25, SURF4, TIPARP, TKT, TRMT1, UAP1, UBC, VPS37A, ZCCHC6	19	16	Developmental disorder, hereditary disorder, metabolic disease
20	ANXA5, collagen, collagen α1, collagen type I, collagen type II, collagen type III, collagen type IV, collagen(s), complement component 1, CPLA2, CSTB, CTSB, eotaxin, FDPS, fibrin, FUBP3, G6PD, GPIIB-IIIA, Hsp27, IARS, laminin, P38 MAPK, PDGF (complex), PDGF-AA, peroxidase (miscellaneous), PKG, PLOD2, PRDX1, PRDX5, QARS, RRBP1, SERPINH1, TNC, TPP1, trypsin	17	15	Cancer, endocrine system disorders, organismal injury and abnormalities
21	AMPK, caspase 3/7, CD3, CD8, cyclin B, FASN, HNRNPA2B1, HNRNPR, IFN, IFNγ, IFNAR, IL-2R, IL12 (complex), interferon-α, JAK, KHDRBS1, LGALS1, Mek, MHC, MTORC1, p70 S6K, p85 (PIK3R), PEBP1, PGAM1, PGD, PRKAA, PTBP1, PTK, RARS, SF3A1, SREBP, STAT1, STAT5a/b, TCR, TWF1	14	13	RNA post-transcriptional modification, dermatological diseases and conditions, cell death and survival
22	ARPIN/C15orf38-AP3S2, ATIC, ESD, FAM169A, γ-tubulin, GANAB, GBA3, GPATCH1, HEATR5A, KIAA1551, KIF18B, LMNA, MAPRE1, MUM1L1, NAPG, PAICS, PRKCSH, RCCD1, RNA polymerase II, SLC22A23, SLC33A1, SLC41A2, TAGLN2, TCF, THUMPD3, TMEM64, TMEM164, TMEM201, TMEM183A, TRAF6, UBC, UCHL1, WDR70, ZNF397, ZNF280C	8	9	Carbohydrate metabolism, small molecule biochemistry, cell morphology
23	ALP, Ap1, C1q, Creb, FGF, FGFR, FN1, HDL, hemoglobin, HIST1H2BL, histon, HMGB1, IgG, IgG1, IgG2a, IgM, IL1, IL12 (family), immunoglobulin, insulin, ITGB1, LASP1, LDL, LRP, NADPH oxidase, NR1H, PDGF BB, PEA15, PI3K (family), PLD, pro-inflammatory cytokine, Shc, SLC38A2, TGFβ, TSH	6	7	Cell-to-cell signaling and interaction, tissue development, cardiovascular system development and function
24	Adaptor protein 2, ADCY, βARK, CAP2, CG, chemokine, clathrin, CRH, estrogen receptor, FPR, FSH, G protein, G protein α, G protein αi, G protein β γ, Gi-coupled receptor, growth hormone, HINT1, IKK (complex), IL8r, LH, LPAR2, metalloprotease, MMP19, myosin2, NMDA receptor, PDGFR, PDXK, PLC, PPIH, proinsulin, S1PR5, SFK, TNF (family), TRHR	1	2	Connective tissue development and function, tissue morphology, behavior

**Table 5:** 500 categories of predicted diseases and functions regulated by vancomycin in HK-2 cells.

Categories	Diseases or Functions Annotation	*p*-Value	Activation *z*-Score	Molecules	Number of Molecules
Cell death and survival	Cell death	2.14 × 10^−35^	0.626	AARS, ACAT1, ACLY, ACTN4, AHSA1, AKR1B1, ALDOA, ALDOC, ANXA1, ANXA2, ANXA5, AP2B1, APEX1, ARHGDIA, ATP1A1, ATP5A1, B2M, BASP1, BSG, BTF3, C1QBP, CACYBP, CALR, CANX, CAPN1, CAPN2, CAPNS1, CAPRIN1, CAST, CAV1, CCT2, CCT3, CCT4, CCT5, CCT6A, CCT7, CCT8, CD44, CD59, CDK1, CFL1, CLIC4, CSE1L, CSTB, CTNNA1, CTSB, CTTN, CYR61, DDX3X, DDX5, DHX9, DNAJA1, DNM1L, DYNC1H1, EEF1A1, EEF1D, EHD1, EIF2S1, EIF3B, EIF3C, EIF3I, EIF4G1, EIF5A, EIF6, ENO1, EZR, FASN, FDPS, FKBP1A, FKBP4, FLNA, FLNB, FN1, FUS, G6PD, GAPDH, GLO1, GLUD1, GML, GNAI2, GNB2L1, GPI, GSTP1, HADHA, HDGF, HEXB, HINT1, HIST1H1C, HLA-A, HLA-B, HMGB1, HNRNPA1, HNRNPK, HSD17B10, HSP90AA1, HSP90AB1, HSP90B1, HSPA4, HSPA5, HSPA8, HSPA9, HSPB1, HSPD1, HYOU1, IGF2R, ILF2, ILF3, IMMT, IMPDH2, ITGB1, KHDRBS1, KPNA2, KPNB1, KRT18, KRT8, LDHA, LGALS1, LMNA, LMNB1, LONP1, MAP1B, MAP4, MAPK1, MCM7, MDH1, MSN, MVP, MYH9, NCL,	232
				NPM1, NUMA1, OTUB1, P4HB, PA2G4, PAFAH1B2, PARK7, PCBP2, PCMT1, PCNA, PDCD6, PDIA3, PDXK, PEA15, PEBP1, PHB2, PKM, PLEC, PLIN3, PPIA, PPP1CA, PPP2CA, PPP2R1A, PRDX1, PRDX2, PRDX4, PRDX5, PRKDC, PRMT5, PRPF19, PSMB1, PSMC1, PSMC4, PSMD2, PSME2, PTGES3, PTMA, QARS, RAD23B, RAN, RANBP1, RBM3, RHOA, RPL10, RPLP0, RPS24, RPS3, RPS3A, RPSA, RRBP1, RTN4, S100A6, SDHA, SERPINH1, SET, SF3A1, SFN, SFPQ, SH3BGRL3, SLC25A5, SLC25A6, SND1, SOD1, SPTBN1, SQSTM1, SSRP1, STAT1, STIP1, STMN1, STOML2, TAGLN2, TCP1, TFRC, TNC, TPM3, TPP1, TRIM28, TUBB, TUBB6, TUFM, TXNDC5, UBA1, UBE2L3, UBE2N, UCHL1, VAPA, VCL, VCP, VDAC1, VDAC2, VIM, XPO1, XRCC5, XRCC6, YBX1, YBX3, YWHAB, YWHAE, YWHAG, YWHAH, YWHAQ, YWHAZ, ZYX	
Cellular growth and proliferation	Proliferation of cells	5.39 × 10^−34^	−1.530	AARS, ACAT1, ACLY, ACTG1, ACTN1, ACTN4, AHCY, AHNAK, AHSA1, AKR1B1, ALDOA, ANXA1, ANXA2, ANXA6, APEX1, ARF1, ARHGDIA, ATIC, ATP5A1, ATP5B, B2M, BASP1, BSG, BUB3, C1QBP, CACYBP, CALR, CAPN1, CAPN2, CAPNS1, CAPRIN1, CAPZA1, CAST, CAV1, CCT2, CCT3, CCT5, CCT7, CD44, CD59, CDK1, CFL1, CLIC1, CLTC, COPE, CSE1L, CSRP1, CTNNA1, CTSB, CTTN, CYR61, DBI, DBN1, DDX3X, DDX5, DNAJA1, DNAJA2, DNM1L, DPY30, DPYSL2, DYNC1H1, EEF1A1, EEF1B2, EEF1D, EIF3B, EIF3C, EIF3I, EIF4A1, EIF4G1, EIF5A, EIF6, ENO1, EZR, FASN, FKBP1A, FKBP4, FLNA, FN1, FUS, G3BP1, G6PD, GAPDH, GML, GNAI2, GNB2L1, GPI, GSTP1, H2AFY, H3F3A/H3F3B, HADHA, HDGF, HEXB, HINT1, HMGB1, HNRNPA1, HNRNPA2B1, HNRNPK, HNRNPL, HNRNPM, HNRNPR, HNRNPU, HSP90AA1, HSP90AB1, HSP90B1, HSPA4, HSPA5, HSPA8, HSPB1, HSPD1, IFITM3, IGF2R, ILF2, ILF3, IMMT, IMPDH2, IPO7, IQGAP1, ITGB1, KHDRBS1, KPNA2, KRT8, LAMB1, LASP1, LDHA, LGALS1, LMNA, LMNB1, LONP1, MAP1B, MAPK1, MAPRE1, MARCKS, MCM3, MCM4, MCM7, MVP, MYH10, MYH9, NACA, NAP1L1, NASP, NCL, NNMT, NPM1, NUMA1, PA2G4, PARK7, PCNA, PDCD6, PDIA3, PDXK, PEA15, PEBP1, PFKP, PFN1, PGK1, PICALM, PKM, PLEC, PLIN3, PLS3, PPIA, PPM1G, PPP1CA, PPP2CA, PPP2R1A, PRDX1, PRDX2, PRDX4, PRKCSH, PRKDC, PRMT5, PRPF19, PSMA4, PSMC1, PSMC4, PSMD2, PSME2, PTBP1, PTGES3, PTMA, RAB11A, RAN, RANBP1, RBM3, RHOA, RNH1, RPS3A, RPS4X, RPSA, RTN4, S100A6, SAE1, SEPT9, SERPINH1, SET, SFN, SFPQ, SHMT2, SLC25A5, SLC25A6, SND1, SNX3, SOD1, SPTAN1, SPTBN1, SQSTM1, STAT1, STIP1, STMN1, SURF4, TAGLN, TAGLN2, TCP1, TFRC, TLN1, TMPO, TNC, TPM3, TRIM28, TUBB, TUBB4B, TXLNA, TXNDC5, UBA1, UBE2L3, UBE2N, UCHL1, VAPA, VCL, VCP, VDAC1, VIM, WARS, XRCC5, XRCC6, YBX1, YBX3, YWHAG, YWHAQ, YWHAZ, ZYX	242
Cell death and survival	Apoptosis	8.59 × 10^−31^	0.054	AARS, ACLY, ACTN4, AHSA1, AKR1B1, ALDOA, ALDOC, ANXA1, ANXA2, ANXA5, APEX1, ARHGDIA, ATP1A1, B2M, BASP1, BSG, BTF3, C1QBP, CALR, CANX, CAPN1, CAPN2, CAPNS1, CAPRIN1, CAST, CAV1, CCT2, CCT4, CD44, CD59, CDK1, CFL1, CLIC4, CSE1L, CSTB, CTNNA1, CTSB, CTTN, CYR61, DDX3X, DDX5, DHX9, DNAJA1, DNM1L, DYNC1H1, EEF1A1, EEF1D, EHD1, EIF2S1, EIF3B, EIF3C, EIF3I, EIF4G1, EIF5A, EIF6, ENO1, EZR, FASN, FKBP1A, FKBP4, FLNA, FLNB, FN1, FUS, G6PD, GAPDH, GLO1, GLUD1, GML, GNAI2, GNB2L1, GPI, GSTP1, HDGF, HEXB, HINT1, HIST1H1C, HLA-B,	192
				HMGB1, HNRNPA1, HNRNPK, HSD17B10, HSP90AA1, HSP90AB1, HSP90B1, HSPA4, HSPA5, HSPA8, HSPA9, HSPB1, HSPD1, HYOU1, IGF2R, ILF3, IMMT, ITGB1, KHDRBS1, KPNA2, KRT18, KRT8, LDHA, LGALS1, LMNA, LMNB1, MAP1B, MAP4, MAPK1, MDH1, MSN, MVP, NCL, NPM1, NUMA1, OTUB1, P4HB, PA2G4, PAFAH1B2, PARK7, PCBP2, PCMT1, PCNA, PDCD6, PDIA3, PDXK, PEA15, PEBP1, PHB2, PKM, PPIA, PPP1CA, PPP2CA, PPP2R1A, PRDX1, PRDX2, PRDX4, PRDX5, PRKDC, PRMT5, PRPF19, PSMB1, PTMA, QARS, RAD23B, RANBP1, RBM3, RHOA, RPL10, RPLP0, RPS24, RPS3, RPS3A, RRBP1, RTN4, S100A6, SERPINH1, SET, SFN, SFPQ, SH3BGRL3, SLC25A5, SLC25A6, SND1, SOD1, SPTBN1, SQSTM1, SSRP1, STAT1, STIP1, STMN1, STOML2, TAGLN2, TCP1, TFRC, TNC, TRIM28, TXNDC5, UBA1, UCHL1, VCL, VCP, VDAC1, VDAC2, VIM, XPO1, XRCC5, XRCC6, YBX1, YBX3, YWHAB, YWHAE, YWHAQ, YWHAZ	
Cell death and survival	Necrosis	1.65 × 10^−30^	1.232	AARS, ACAT1, ACLY, AHSA1, AKR1B1, ALDOA, ANXA1, ANXA2, AP2B1, APEX1, ARHGDIA, ATP1A1, ATP5A1, B2M, BSG, CALR, CAPN1, CAPN2, CAPNS1, CAPRIN1, CAST, CAV1, CCT2, CCT3, CCT4, CCT5, CCT6A, CCT7, CCT8, CD44, CD59, CDK1, CLIC4, CSE1L, CTNNA1, CTSB, CTTN, CYR61, DDX3X, DNM1L, DYNC1H1, EEF1A1, EEF1D, EIF2S1, EIF3B, EIF3C, EIF3I, EIF6, ENO1, EZR, FASN, FDPS, FKBP1A, FKBP4, FLNA, FLNB, FN1, FUS, G6PD, GAPDH, GLO1, GLUD1, GML, GNAI2, GNB2L1, GPI, GSTP1, HADHA, HDGF, HINT1, HIST1H1C, HLA-B, HMGB1, HNRNPA1, HNRNPK, HSD17B10, HSP90AA1, HSP90AB1, HSP90B1, HSPA4, HSPA5, HSPA8, HSPA9, HSPB1, HSPD1, HYOU1, IGF2R, ILF2, IMMT, ITGB1, KHDRBS1, KPNA2, KPNB1, KRT18, KRT8, LDHA, LGALS1, LMNA, LMNB1, LONP1, MAP1B, MAP4, MAPK1, MCM7, MDH1, MSN, MVP, NCL, NPM1, OTUB1, P4HB, PA2G4, PARK7, PCBP2, PCNA, PDIA3, PEA15, PEBP1, PKM, PLEC, PPIA, PPP1CA, PPP2CA, PPP2R1A, PRDX1, PRDX2, PRKDC, PRMT5, PRPF19, PSMB1, PSMD2, PTMA, RAD23B, RAN, RBM3, RHOA, RPL10, RPLP0, RPS24, RPS3, RPS3A, RPSA, RTN4, S100A6, SDHA, SERPINH1, SET, SF3A1, SFN, SFPQ, SLC25A5, SLC25A6, SND1, SOD1, SPTBN1, SQSTM1, SSRP1, STAT1, STIP1, STMN1, STOML2, TAGLN2, TCP1, TFRC, TPP1, TRIM28, TUBB, TUBB6, TUFM, UBA1, UBE2L3, UCHL1, VAPA, VCP, VDAC1, VDAC2, VIM, XPO1, XRCC5, XRCC6, YBX1, YWHAB, YWHAE, YWHAG, YWHAH, YWHAQ, YWHAZ, ZYX	188
Infectious disease	Viral infection	2.33 × 10^−30^	−1.968	ACTN1, ACTR2, ANXA1, ANXA2, ANXA5, ANXA6, AP1B1, AP2B1, ARF1, ARPC1B, ATP5B, B2M, BSG, BTF3, C14orf166, CAV1, CCT2, CD44, CLIC4, CLTC, COPA, COPB1, COPB2, COPG1, CSE1L, CTSB, DDX3X, DDX5, DDX6, DHX9, DNAJA1, DNAJA2, DNM1L, EEF1A1, EIF2S1, EIF3C, EIF3I, FASN, FDPS, FKBP1A, FLNA, FN1, G3BP1, GANAB, GAPDH, GML, GPI, H3F3A/H3F3B, HIST1H1C, HLA-A, HLA-B, HMGB1, HNRNPH1, HNRNPK, HNRNPM, HNRNPU, HSP90AA1, HSP90AB1, HSP90B1, HSPA5, HSPA9, HSPD1, IFITM3, IGF2R, ILF3, IMPDH2, ITGB1, KHDRBS1, KHSRP, KPNB1, KRT18, KRT8, LGALS1, LMNA, LONP1, MAP4, MAPK1, MVP, NCL, NPM1, PCBP2, PDIA3, PDIA6, PDXK, PGM1, PICALM, PLIN3, PLOD2, PPIA, PRDX1, PRDX2, PSMA1, PSMA2, PSMA5, PSMA7, PSMC4, PSMD2, PSME2, PTGES3, PYGL, RAB11A, RAB1B, RAB7A, RAN, RANBP1, RHOA, RNH1, RPL10A, RPL12, RPL18, RPL3, RPL5, RPS10, RPS27A, RPS5, RPSA, SAE1, SF3A1, SF3B1, SFN, SFPQ,	142
				SNRPD3, SPTAN1, SPTBN1, SSRP1, STAT1, STIP1, SUPT16H, TAGLN2, TALDO1, TFRC, TKT, TMPO, TUBB, TUBB4B, TWF1, UAP1, UBE2L3, UQCRC1, XPO1, YBX1, ZYX	
Cell death and survival	Cell death of tumor cell lines	1.13 × 10^−27^	0.949	AHSA1, AKR1B1, ANXA2, APEX1, ARHGDIA, ATP1A1, ATP5A1, B2M, CALR, CAPN1, CAPN2, CAPNS1, CAST, CAV1, CCT2, CCT3, CCT4, CCT5, CCT6A, CCT7, CCT8, CD44, CD59, CDK1, CSE1L, CTSB, CTTN, CYR61, DDX3X, DNM1L, DYNC1H1, EIF2S1, ENO1, EZR, FASN, FKBP1A, FLNB, FN1, G6PD, GAPDH, GLO1, GML, GNB2L1, GPI, GSTP1, HINT1, HNRNPA1, HNRNPK, HSP90AB1, HSPA4, HSPA5, HSPA8, HSPA9, HSPB1, HSPD1, IGF2R, ILF2, IMMT, ITGB1, KHDRBS1, KPNA2, KPNB1, KRT18, LGALS1, LMNA, LMNB1, LONP1, MAPK1, MCM7, MSN, MVP, NCL, NPM1, OTUB1, P4HB, PA2G4, PARK7, PCBP2, PCNA, PEA15, PEBP1, PKM, PPIA, PPP1CA, PPP2CA, PPP2R1A, PRDX1, PRDX2, PRKDC, PRMT5, PRPF19, PSMD2, PTMA, RAD23B, RHOA, RPLP0, RPS24, RPS3, RTN4, S100A6, SF3A1, SFN, SFPQ, SLC25A5, SLC25A6, SND1, SOD1, SQSTM1, SSRP1, STAT1, STMN1, STOML2, TAGLN2, TCP1, TFRC, TRIM28, TUBB6, TUFM, UBE2L3, UCHL1, VCP, VDAC1, VDAC2, XPO1, XRCC5, XRCC6, YBX1, YWHAE, YWHAG, YWHAH, YWHAZ	131
Protein synthesis	Translation	6.09 × 10^−25^	0.792	APEX1, CALR, CAPRIN1, DDX3X, DDX6, DHX9, EEF1A1, EEF1B2, EEF2, EIF2S1, EIF3B, EIF3C, EIF3I, EIF3M, EIF4A1, EIF4G1, EIF4H, EIF5A, EPRS, FN1, GAPDH, GNB2L1, HNRNPK, HSPA5, HSPB1, ILF3, ITGB1, MARS, NACA, NCL, PABPC1, PCBP1, PPP1CA, PTBP1, RBM3, RPL23, RPS24, RPS3, RPS3A, RPS4X, RPS5, RPS7, RRBP1, SYNCRIP, TUFM, WARS, YBX1	47
Protein synthesis	Translation of protein	2.00 × 10^−23^	0.860	APEX1, CALR, CAPRIN1, DDX3X, DDX6, DHX9, EEF1A1, EEF1B2, EIF2S1, EIF3B, EIF3C, EIF3I, EIF3M, EIF4A1, EIF4G1, EIF4H, EIF5A, EPRS, FN1, GAPDH, GNB2L1, HNRNPK, HSPA5, HSPB1, ILF3, ITGB1, MARS, NACA, NCL, PABPC1, PCBP1, PPP1CA, PTBP1, RBM3, RPL23, RPS24, RPS3, RPS3A, RPS4X, RPS5, RPS7, RRBP1, SYNCRIP, WARS, YBX1	45
Protein synthesis	Expression of protein	2.95 × 10^−22^	0.229	ANXA1, APEX1, CALR, CAPRIN1, CAV1, DDX3X, DDX6, DHX9, EEF1A1, EEF1B2, EIF2S1, EIF3B, EIF3C, EIF3I, EIF3M, EIF4A1, EIF4G1, EIF4H, EIF5A, EPRS, FN1, GAPDH, GNB2L1, HNRNPK, HSPA5, HSPB1, ILF3, ITGB1, KHDRBS1, MARS, NACA, NCL, PABPC1, PCBP1, PPM1G, PPP1CA, PTBP1, RBM3, RPL23, RPS24, RPS3, RPS3A, RPS4X, RPS5, RPS7, RRBP1, SOD1, SYNCRIP, WARS, YBX1	50
Protein synthesis	Synthesis of protein	3.25 × 10^−22^	−0.265	ANXA1, APEX1, CALR, CAPRIN1, CAV1, DDX3X, DDX6, DHX9, EEF1A1, EEF1B2, EEF2, EIF2S1, EIF3B, EIF3C, EIF3I, EIF3M, EIF4A1, EIF4G1, EIF4H, EIF5A, EIF6, EPRS, FN1, GAPDH, GNB2L1, HNRNPK, HSPA5, HSPB1, ILF3, ITGB1, KHDRBS1, MAPK1, MARS, NACA, NCL, NPM1, PABPC1, PARK7, PCBP1, PPM1G, PPP1CA, PTBP1, RBM3, RPL23, RPL5, RPS24, RPS3, RPS3A, RPS4X, RPS5, RPS7, RRBP1, SOD1, STIP1, SYNCRIP, TUFM, WARS, YBX1	58
Cell death and survival	Apoptosis of tumor cell lines	3.17 × 10^−21^	0.816	AHSA1, AKR1B1, ANXA2, ARHGDIA, B2M, CALR, CAPN1, CAPN2, CAPNS1, CAST, CAV1, CCT2, CCT4, CD44, CD59, CDK1, CSE1L, CTSB, CTTN, CYR61, DNM1L, DYNC1H1, ENO1, EZR, FASN, FLNB, FN1, G6PD, GAPDH, GLO1, GML, GNB2L1, GSTP1, HINT1, HNRNPA1, HNRNPK, HSP90AB1, HSPA4, HSPA5, HSPA8, HSPA9, HSPB1, HSPD1, IGF2R, IMMT, ITGB1, KHDRBS1, KPNA2, KRT18, LGALS1, LMNB1, MAPK1, MSN, MVP, NCL, NPM1, OTUB1, P4HB, PA2G4, PARK7, PCBP2, PCNA, PEA15, PEBP1, PPIA, PPP1CA, PPP2CA, PPP2R1A, PRDX1, PRKDC, PRMT5, PRPF19, PTMA, RAD23B, RHOA, RPLP0, RPS24, RPS3, RTN4, S100A6, SFN, SFPQ, SLC25A5, SND1, SOD1, SSRP1, STAT1,	102
				STMN1, STOML2, TAGLN2, TCP1, TFRC, TRIM28, UCHL1, VCP, VDAC1, VDAC2, XPO1, XRCC5, YBX1, YWHAE, YWHAZ	
Cellular assembly and organization, cellular function and maintenance	Organization of cytoplasm	4.80 × 10^−20^	−1.198	ACTG1, ACTN4, ACTR2, ACTR3, ALDOA, ANXA1, ARHGDIA, ARPC2, BASP1, BSG, C1QBP, CALR, CANX, CAP1, CAPG, CAPN2, CAPNS1, CAPRIN1, CAPZB, CAST, CAV1, CD44, CDK1, CFL1, CKAP4, CLTC, COPB2, CORO1C, CSRP1, CTTN, CYR61, DBN1, DNM1L, DPYSL2, EEF1A1, EHD1, ENAH, EZR, FASN, FKBP4, FLNA, FLNB, FLNC, FN1, GAPDH, GDI1, HDGF, HEXB, HMGB1, HNRNPK, HSP90AA1, HSPB1, IQGAP1, ITGB1, KIF5B, KPNB1, KRT18, KRT9, LAMB1, LASP1, LONP1, MAP1B, MAP4, MAPK1, MAPRE1, MARCKS, MSN, MYH10, MYH9, NNMT, NPLOC4, NSFL1C, NUMA1, PARK7, PCM1, PDIA3, PFN1, PHGDH, PICALM, PKM, PLEC, PLS3, PPP2CA, PRKCSH, PRKDC, RAB11A, RAN, RANBP1, RHOA, RRBP1, RTN4, SEPT2, SEPT7, SEPT9, SOD1, SPTAN1, SPTBN1, SSRP1, STIP1, STMN1, STOML2, SURF4, TLN1, TMOD3, TNC, TPM3, TPP1, TUBB, TWF2, UCHL1, VAPA, VCL, VIM, XPO1, YBX1, YWHAH	116
Protein synthesis	Metabolism of protein	8.33 × 10^−20^	0.064	ANXA1, APEX1, CALR, CANX, CAPN1, CAPN2, CAPNS1, CAPRIN1, CAST, CAV1, COPG1, CSTB, CTSB, DDX3X, DDX6, DHX9, EEF1A1, EEF1B2, EEF2, EIF2S1, EIF3B, EIF3C, EIF3I, EIF3M, EIF4A1, EIF4G1, EIF4H, EIF5A, EIF6, EPRS, FLNA, FN1, GAPDH, GNB2L1, HEXB, HNRNPK, HSP90B1, HSPA5, HSPB1, HSPD1, ILF3, IPO9, ITGB1, KHDRBS1, LONP1, MAPK1, MARS, MYH9, NACA, NCL, NPEPPS, NPM1, PABPC1, PARK7, PCBP1, PDIA3, PPM1G, PPP1CA, PPP2CA, PSMC4, PSMD2, PTBP1, RBM3, RPL23, RPL5, RPS24, RPS3, RPS3A, RPS4X, RPS5, RPS7, RRBP1, SNX3, SOD1, SQSTM1, STIP1, SYNCRIP, TPP1, TUFM, UBE2L3, UBE2N, UBXN4, UCHL1, VCP, WARS, XPO1, YBX1	87
Cancer, organismal injury and abnormalities, reproductive system disease	Mammary tumor	1.12 × 10^−19^	1.847	AKR1B1, ALDOA, ALDOC, ALYREF, ANXA1, ARHGDIA, ATP5A1, BSG, C1QBP, CAV1, CBX3, CCT3, CD44, CDK1, CSE1L, CTTN, CYR61, DDX5, DYNC1H1, EEF1A1, EIF3B, EIF3C, EIF4A1, ENAH, ENO1, ETFA, FASN, FDPS, FKBP1A, FLNA, FLNB, FN1, FUS, GCN1L1, GLO1, GSTP1, H2AFY, H3F3A/H3F3B, HDLBP, HLA-A, HLA-B, HNRNPA1, HSP90AA1, HSP90AB1, HSP90B1, HSPA5, HSPD1, ILF3, ITGB1, KHSRP, KPNA2, KRT18, KRT8, LAMB1, LASP1, LGALS1, LMNA, LONP1, MAPRE1, MARS, MCM4, MCM6, MYH10, MYH9, NAP1L4, NCL, NPEPPS, P4HB, PAFAH1B2, PCNA, PDCD6, PFN1, PGK1, PGM1, PKM, PLEC, PRDX1, PRDX5, PRKCSH, PTBP1, PTRF, RANBP1, RHOA, RPL4, RPL6, RPLP0, RPS24, RPS3, RPS4X, S100A6, SF3B1, SFPQ, SHMT2, SLC25A5, SLC25A6, SOD1, STAT1, STIP1, TAGLN, TAGLN2, TCP1, TLN1, TNC, TPI1, TUBA1B, TUBB, TUBB4B, TUBB6, UBA1, UCHL1, VCL, VDAC2, WDR1, XPO1, XRCC5, YWHAH, YWHAQ, YWHAZ, ZYX	119
Dermatological diseases and conditions	Psoriasis	2.52 × 10^−19^		ANXA1, ANXA2, ARHGDIA, ARPC1B, C1QBP, CALR, CAV1, CBX3, CCT5, CFL1, COPB2, CSTB, CTSB, CYB5R3, DBN1, EEF1A1, EIF2S1, EIF2S2, EIF5A, EIF6, FASN, FKBP1A, FN1, GAPDH, GARS, GSTP1, H2AFY, HSPA5, HSPA8, KPNA2, KPNB1, KRT1, KRT18, LGALS1, MARCKS, OTUB1, P4HB, PCBP2, PCMT1, PCNA, PGAM1, PGD, PKM, PPP2CA, PSME2, PTRF, RAN, RANBP1, SERBP1, SFN, SLC25A5, SPTAN1, STAT1, SYNCRIP, TAGLN, TNC, TUBB, TWF1, UBE2N, VDAC1, YWHAB, YWHAE, YWHAQ	63
Infectious disease	Infection of cells	2.88 × 10^−19^	−2.041	ACTR2, ANXA2, AP1B1, AP2B1, ARF1, ARPC1B, ATP5B, BSG, C1QBP, CAV1, CCT2, CD44, CLTC, COPA, COPB1, COPB2, COPG1, CTSB, DDX3X, DHX9, DNAJA2, EIF3I, FN1, G3BP1,	78
				GANAB, GML, GPI, H3F3A/H3F3B, HMGB1, HNRNPH1, HNRNPK, HNRNPU, HSPA5, HSPA9, IFITM3, IGF2R, ITGB1, KHDRBS1, KPNB1, KRT18, MAP4, PCBP2, PDIA3, PDIA6, PGM1, PICALM, PLOD2, PSMA1, PSMA2, PSMA5, PSMA7, PSMC4, PSME2, PTGES3, RAB1B, RANBP1, RNH1, RPL10A, RPL12, RPL18, RPL3, RPL5, SF3A1, SF3B1, SNRPD3, SPTAN1, SPTBN1, STAT1, STIP1, TAGLN2, TFRC, TWF1, UAP1, UBE2L3, UQCRC1, XPO1, YBX1, ZYX	
Infectious disease	Replication of virus	5.92 × 10^−19^	−0.358	ANXA5, ANXA6, ATP5B, B2M, BTF3, C14orf166, CAV1, CLIC4, COPA, COPB1, COPB2, COPG1, CSE1L, DDX3X, DDX5, DDX6, DHX9, DNAJA1, EEF1A1, EIF2S1, EIF3C, FASN, FDPS, FKBP1A, G3BP1, HMGB1, HNRNPM, HSP90AA1, HSP90AB1, HSP90B1, HSPD1, IFITM3, ILF3, KPNB1, KRT8, LONP1, MAPK1, MVP, NCL, NPM1, PCBP2, PLIN3, PPIA, PRDX1, PRDX2, PSMA1, PSMD2, RAB11A, RPS10, RPS27A, RPS5, RPSA, SAE1, SF3A1, SF3B1, SFPQ, SSRP1, STAT1, TMPO, TUBB, XPO1, YBX1	62
Neurological disease	Progressive motor neuropathy	9.90 × 10^−19^		ANXA1, ANXA2, ANXA5, CAPN2, CAPZB, CCT2, COPE, CSE1L, CYR61, DBI, EEF1A1, EIF4A1, EIF4G1, ENO2, EZR, FASN, FUS, GSTP1, H3F3A/H3F3B, HLA-B, HN1, HNRNPA1, HSPA5, HSPB1, IMPDH2, LDHA, LDHB, MAP1B, MAPK1, MDH1, NNMT, PARK7, PCNA, PDIA3, PEBP1, PFKP, PFN1, PGK1, PRDX2, PSMC1, PTGES3, RAN, RHOA, RPL3, RPL5, RPS3A, RPS4X, RTN4, S100A6, SLC25A6, SOD1, STMN1, TFRC, TUBA1B, UCHL1, VCP, VIM, VPS35	58
Cellular assembly and organization, cellular function and maintenance	Organization of cytoskeleton	1.01 × 10^−18^	−1.190	ACTG1, ACTN4, ACTR2, ACTR3, ALDOA, ANXA1, ARHGDIA, ARPC2, BASP1, BSG, C1QBP, CALR, CANX, CAP1, CAPG, CAPN2, CAPNS1, CAPRIN1, CAPZB, CAST, CAV1, CD44, CDK1, CFL1, CKAP4, COPB2, CORO1C, CSRP1, CTTN, CYR61, DBN1, DNM1L, DPYSL2, EEF1A1, EHD1, ENAH, EZR, FASN, FKBP4, FLNA, FLNB, FLNC, FN1, GAPDH, GDI1, HDGF, HMGB1, HNRNPK, HSP90AA1, HSPB1, IQGAP1, ITGB1, KIF5B, KPNB1, KRT18, KRT9, LAMB1, LASP1, MAP1B, MAP4, MAPK1, MAPRE1, MARCKS, MSN, MYH10, MYH9, NNMT, NUMA1, PCM1, PDIA3, PFN1, PHGDH, PICALM, PKM, PLEC, PLS3, PPP2CA, PRKCSH, PRKDC, RAB11A, RAN, RANBP1, RHOA, RRBP1, RTN4, SEPT2, SEPT7, SEPT9, SOD1, SPTAN1, SPTBN1, SSRP1, STIP1, STMN1, TLN1, TMOD3, TNC, TPM3, TUBB, TWF2, UCHL1, VAPA, VCL, VIM, XPO1, YBX1, YWHAH	107
Post-translational modification, protein folding	Folding of protein	1.21 × 10^−18^	0.988	AARS, AIP, B2M, CALR, CANX, CCT4, DNAJA1, DNAJA2, ERP29, FKBP1A, FKBP4, HSP90AA1, HSP90AB1, HSPA5, HSPA8, HSPB1, HSPD1, P4HB, PDIA6, PPIA, PRDX4, RAB7A, TBCA, TCP1	24
Neurological disease, psychological disorders	Disorder of basal ganglia	2.74 × 10^−18^		ACAT1, AHCY, ANXA2, B2M, BASP1, CAPNS1, CAPZB, CAV1, CD44, COPE, CSE1L, CYC1, DDX1, DNAJA1, EEF1A1, EIF4G1, ENO2, FKBP4, GAPDH, GPI, GSTP1, H3F3A/H3F3B, HADH, HINT1, HLA-B, HNRNPU, HSP90AA1, HSPA5, HSPA8, HSPB1, LAMB1, LDHA, LDHB, MAP1B, MDH1, MYL12A, NPM1, PARK7, PCMT1, PCNA, PDE6H, PDLIM1, PEBP1, PFN1, PGK1, PKM, PLOD2, PPIA, PRDX2, PSMC1, PSME1, PTGES3, RAB11A, RAN, RANBP1, RPL3, RPS3A, RPS4X, RPSA, RTN4, SDHA, SLC25A6, SOD1, STMN1, TPI1, TPM3, TUBA1B, TUBB4B, UCHL1, UQCRC1, VIM, VPS35, XRCC6, YWHAZ	74
Cellular assembly and organization, cellular function and maintenance	Microtubule dynamics	4.81 × 10^−18^	−0.705	ACTG1, ACTN4, ACTR2, ACTR3, ARPC2, BASP1, BSG, C1QBP, CANX, CAP1, CAPG, CAPN2, CAPNS1, CAPRIN1, CAPZB, CAST, CAV1, CD44, CDK1, CFL1, CKAP4, COPB2, CSRP1, CTTN, CYR61, DBN1, DNM1L, DPYSL2, EEF1A1, EHD1, ENAH, EZR, FASN, FKBP4, FLNA, FN1, GAPDH, GDI1, HDGF, HMGB1, HNRNPK, HSP90AA1, HSPB1, IQGAP1, ITGB1, KIF5B, KPNB1, KRT18, LAMB1, LASP1, MAP1B, MAP4, MAPK1, MAPRE1, MARCKS, MSN, MYH10, MYH9, NNMT, NUMA1, PCM1, PDIA3, PFN1, PHGDH, PICALM, PKM, PLEC, PPP2CA, PRKCSH, RAB11A, RAN, RANBP1, RHOA, RRBP1, RTN4, SEPT2, SEPT7, SEPT9, SOD1, SPTBN1, SSRP1, STIP1, STMN1, TLN1, TNC, TPM3, TUBB, TWF2, UCHL1, VAPA, VCL, VIM, XPO1, YBX1, YWHAH	95
Infectious disease	Replication of RNA virus	4.87 × 10^−18^	−0.056	ANXA5, ANXA6, B2M, BTF3, C14orf166, CAV1, CLIC4, COPA, COPB1, COPB2, COPG1, CSE1L, DDX3X, DDX5, DDX6, DHX9, DNAJA1, EEF1A1, EIF2S1, EIF3C, FASN, FDPS, G3BP1, HMGB1, HNRNPM, HSP90AA1, HSP90AB1, HSPD1, IFITM3, ILF3, KPNB1, LONP1, MAPK1, MVP, NCL, NPM1, PCBP2, PLIN3, PPIA, PRDX1, PRDX2, PSMA1, PSMD2, RAB11A, RPS10, RPS27A, RPS5, RPSA, SAE1, SF3A1, SF3B1, SFPQ, STAT1, TMPO, TUBB, XPO1, YBX1	57
Infectious disease	Infection by RNA virus	7.40 × 10^−18^	−2.209	ACTR2, ANXA1, ANXA2, AP1B1, AP2B1, ARF1, ARPC1B, ATP5B, B2M, BSG, CCT2, CD44, CLTC, COPA, COPB1, COPB2, COPG1, CTSB, DDX3X, DHX9, DNAJA2, EIF3I, FDPS, FN1, G3BP1, GANAB, GML, GPI, H3F3A/H3F3B, HMGB1, HNRNPH1, HNRNPK, HNRNPU, HSPA5, HSPA9, IFITM3, IMPDH2, ITGB1, KHDRBS1, KPNB1, MAP4, PCBP2, PDIA3, PDIA6, PGM1, PICALM, PLOD2, PSMA1, PSMA2, PSMA5, PSMA7, PSMC4, PSME2, PTGES3, RAB1B, RANBP1, RHOA, RNH1, RPL10A, RPL12, RPL18, RPL3, RPL5, SF3A1, SF3B1, SNRPD3, SPTAN1, SPTBN1, STAT1, STIP1, TAGLN2, TFRC, TWF1, UAP1, UBE2L3, UQCRC1, XPO1, YBX1, ZYX	79
Neurological disease, skeletal and muscular disorders	Neuromuscular disease	2.20 × 10^−17^		ACAT1, AHCY, ANXA1, ANXA2, B2M, BASP1, CAPNS1, CAPZB, CD44, COPE, CSE1L, CYC1, DDX1, DNAJA1, EEF1A1, EIF4G1, ENO2, FKBP1A, FKBP4, GAPDH, GPI, GSTP1, H3F3A/H3F3B, HADH, HINT1, HLA-B, HNRNPA1, HNRNPU, HSP90AA1, HSPA5, HSPA8, HSPB1, IMPDH2, LAMB1, LDHA, LDHB, MAP1B, MDH1, MYL12A, NPM1, PARK7, PCMT1, PCNA, PDE6H, PDIA3, PDLIM1, PEBP1, PFN1, PGK1, PKM, PLOD2, PPIA, PRDX2, PSMC1, PSME1, PTGES3, RAB11A, RAN, RANBP1, RPL3, RPL5, RPS3A, RPS4X, RPSA, RTN4, SDHA, SLC25A6, SOD1, STMN1, TPI1, TPM3, TUBA1B, UCHL1, UQCRC1, VIM, VPS35, XRCC6, YWHAZ	78
Cancer, organismal injury and abnormalities, reproductive system disease	Breast cancer	6.17 × 10^−17^		AKR1B1, ALDOA, ALDOC, ANXA1, ARHGDIA, ATP5A1, BSG, C1QBP, CAV1, CBX3, CCT3, CD44, CDK1, CSE1L, CYR61, DDX5, DYNC1H1, EEF1A1, EIF3B, EIF3C, EIF4A1, ENAH, ENO1, ETFA, FASN, FDPS, FKBP1A, FLNA, FLNB, FN1, FUS, GCN1L1, GLO1, GSTP1, H2AFY, H3F3A/H3F3B, HLA-A, HLA-B, HNRNPA1, HSP90AA1, HSP90AB1, HSP90B1, HSPD1, ILF3, ITGB1, KHSRP, KPNA2, KRT8, LAMB1, LASP1, LGALS1, LMNA, LONP1, MAPRE1, MARS, MCM6, MYH10, MYH9, NAP1L4, NCL, NPEPPS, P4HB, PAFAH1B2, PCNA, PDCD6, PFN1, PGK1, PGM1, PKM, PLEC, PRDX1, PRDX5, PRKCSH, PTBP1, PTRF, RANBP1, RPL4, RPL6, RPLP0, RPS24, RPS3, S100A6, SF3B1, SFPQ, SHMT2, SLC25A5, SLC25A6, SOD1, STAT1, TAGLN, TAGLN2, TCP1, TLN1, TNC, TPI1, TUBA1B, TUBB, TUBB4B, TUBB6, UBA1, VCL, VDAC2, WDR1, XPO1, YWHAH, YWHAQ, YWHAZ, ZYX	108
Neurological disease	Movement disorders	1.91 × 10^−16^	0.283	ACAT1, AHCY, ANXA2, B2M, BASP1, CANX, CAPNS1, CAPZB, CAV1, CD44, COPE, CSE1L, CSTB, CTSA, CTSB, CYC1, DDX1, DNAJA1, EEF1A1, EEF2, EIF4G1, ENO2, FDPS, FKBP1A, FKBP4, FUS, GAPDH, GPI, GSTP1, H3F3A/H3F3B, HADH, HEXB, HINT1, HLA-B, HNRNPU, HSP90AA1, HSPA5, HSPA8, HSPB1, IGF2R, LAMB1, LDHA, LDHB, LMNA, MAP1B, MDH1, MYL12A, NPM1, PARK7, PCMT1, PCNA, PDE6H, PDLIM1, PEBP1, PFN1, PGK1, PKM, PLOD2, PPIA, PRDX2, PSMC1, PSME1, PTGES3, RAB11A, RAN, RANBP1, RPL3, RPS3A, RPS4X, RPSA, RTN4, SDHA, SLC25A6, SOD1, SQSTM1, STMN1, TPI1, TPM3, TPP1, TUBA1B, TUBB4B, UCHL1, UQCRC1, VIM, VPS35, XRCC6, YWHAZ	87
Cancer	Breast or ovarian cancer	2.54 × 10^−16^		AKR1B1, ALDOA, ALDOC, ANXA1, ARHGDIA, ATIC, ATP5A1, B2M, BSG, C1QBP, CAV1, CBX3, CCT3, CCT5, CD44, CDK1, CSE1L, CYR61, DDX5, DYNC1H1, EEF1A1, EIF3B, EIF3C, EIF4A1, ENAH, ENO1, ETFA, FASN, FDPS, FKBP1A, FLNA, FLNB, FN1, FUS, GAPDH, GCN1L1, GLO1, GSTP1, H2AFY, H3F3A/H3F3B, HLA-A, HLA-B, HN1, HNRNPA1, HSP90AA1, HSP90AB1, HSP90B1, HSPD1, ILF3, ITGB1, KHSRP, KPNA2, KRT8, LAMB1, LASP1, LGALS1, LMNA, LONP1, MAPRE1, MARS, MCM6, MYH10, MYH9, NAP1L4, NCL, NPEPPS, P4HB, PAFAH1B2, PCNA, PDCD6, PDIA3, PEA15, PFN1, PGK1, PGM1, PKM, PLEC, PPP2R1A, PRDX1, PRDX5, PRKCSH, PTBP1, PTRF, RANBP1, RPL4, RPL6, RPLP0, RPS24, RPS3, S100A6, SF3B1, SFPQ, SHMT2, SLC25A5, SLC25A6, SOD1, SSRP1, STAT1, TAGLN, TAGLN2, TCP1, TLN1, TNC, TPI1, TUBA1B, TUBB, TUBB4B, TUBB6, UBA1, VCL, VDAC2, VIM, WDR1, XPO1, YWHAH, YWHAQ, YWHAZ, ZYX	118
Cellular movement	Cell movement	4.44 × 10^−15^	−1.559	ACTN4, ACTR2, ACTR3, AHCY, AKR1B1, ALDOA, ANXA1, ANXA2, ANXA5, ARF1, ARHGDIA, ARPC1B, ARPC2, BSG, C1QBP, CALR, CAP1, CAPG, CAPN1, CAPN2, CAPNS1, CAST, CAV1, CD44, CD59, CDK1, CFL1, CLIC4, CORO1C, CRIP2, CSE1L, CTNNA1, CTSB, CTTN, CYR61, DBN1, DDX3X, DNAJA1, DPYSL2, EHD1, ENAH, EZR, FASN, FKBP4, FLNA, FLNB, FLNC, FN1, G6PD, GDI1, GNAI2, GNB2L1, GPI, HARS, HDGF, HLA-A, HMGB1, HNRNPA2B1, HNRNPK, HNRNPL, HSP90AA1, HSP90AB1, HSP90B1, HSPA5, HSPB1, HSPD1, HYOU1, IGF2R, ILF3, IQGAP1, ITGB1, KHDRBS1, KPNA2, KRT8, LAMB1, LASP1, LGALS1, LMNA, LMNB1, MAP1B, MAPK1, MAPRE1, MARCKS, MCM3, MCM7, MSN, MYH10, MYH9, MYL12A, NACA, NCL, NPM1, PA2G4, PARK7, PDCD6, PEBP1, PFN1, PHB2, PKM, PLEC, PPIA, PRDX1, PRDX2, PRMT5, PTMA, RHOA, RNH1, RPSA, RTN4, S100A6, SEPT7, SEPT9, SERPINH1, SFN, SOD1, SQSTM1, STAT1, STMN1, TAGLN2, TARS, TLN1, TMOD3, TNC, TPM3, VCL, VCP, VIM, WARS, YBX1, YWHAE, YWHAZ, ZYX	132
Dermatological diseases and conditions	Chronic psoriasis	5.65 × 10^−15^		C1QBP, CCT5, COPB2, CSTB, CTSB, EIF2S1, EIF2S2, EIF5A, GARS, GSTP1, HSPA8, KPNA2, KPNB1, MARCKS, PCMT1, PGAM1, PGD, PKM, PPP2CA, PSME2, RANBP1, SLC25A5, STAT1, TAGLN, TWF1, UBE2N	26
Nucleic acid metabolism, small molecule biochemistry	Metabolism of nucleoside triphosphate	1.02 × 10^−14^	−2.160	ACLY, ALDOA, ANXA1, ATP1A1, ATP5A1, ATP5B, ATP5H, CAV1, CCT8, CDK1, CTPS1, DDX1, DDX39B, DDX3X, DDX5, DHX9, DNM1L, G3BP1, HMGB1, HSP90AA1, HSPA8, HSPD1, LONP1, MCM7, MYH10, MYH9, NNMT, OLA1, PKM, PSMC1, PSMC4, SLC25A5, SOD1, VCP, VDAC1	35
Neurological disease, psychological disorders, skeletal and muscular disorders	Parkinson’s disease	1.16 × 10^−14^		ANXA2, CAPZB, COPE, CSE1L, EEF1A1, EIF4G1, ENO2, GSTP1, H3F3A/H3F3B, HLA-B, HSPB1, LDHA, LDHB, MAP1B, MDH1, PARK7, PCNA, PEBP1, PGK1, PRDX2, PSMC1, PTGES3, RAN, RPL3, RPS3A, RPS4X, RTN4, SLC25A6, SOD1, STMN1, TUBA1B, UCHL1, VIM, VPS35	34
Cellular development, cellular growth and proliferation	Proliferation of tumor cell lines	3.28 × 10^−14^	−0.292	ACAT1, ACLY, ACTN4, AHSA1, ALDOA, ANXA1, ANXA2, ANXA6, APEX1, ARF1, BSG, C1QBP, CACYBP, CALR, CAV1, CD44, CD59, CDK1, CSE1L, CTSB, CTTN, CYR61, DDX5, DYNC1H1, EEF1A1, EEF1B2, EIF3C, EIF4A1, EIF5A, EZR, FASN, FLNA, FN1, FUS, G6PD, GAPDH, GNB2L1, H2AFY, HDGF, HMGB1, HNRNPA1, HNRNPA2B1, HNRNPK, HSP90AA1, HSPA5, IGF2R, ILF3, IMMT, IPO7, IQGAP1, ITGB1, KPNA2, KRT8, LGALS1, MAP1B, MAPK1, MAPRE1, MCM7, NASP, NCL, NPM1, NUMA1, PA2G4, PCNA, PDIA3, PEBP1, PFKP, PFN1, PKM, PPP2R1A, PRDX2, PRKCSH, PRMT5, PSMA4, PTBP1, PTMA, RAN, RHOA, S100A6, SEPT9, SET, SFN, SFPQ, SLC25A6, SND1, SOD1, SPTAN1, STAT1, STMN1, TAGLN2, TCP1, TFRC, TRIM28, TUBB, UBA1, UCHL1, XRCC5, XRCC6, YBX1, YWHAG, YWHAQ	101
Molecular transport, protein trafficking	Transport of protein	3.57 × 10^−14^	0.362	AP1B1, ARF1, CALR, CAV1, CFL1, CSE1L, CTSA, DNAJA1, DNAJA2, EHD1, EIF5A, ERP29, GDI2, HSPA9, IGF2R, IPO5, IPO7, IPO9, KPNA2, KPNB1, MAP1B, MYH9, NPM1, PDCD6, PDIA3, PICALM, RAB11A, RAB7A, RAN, SNX3, SOD1, SPTBN1, SQSTM1, TNPO1, VAPA, XPO1, YWHAH	37
Immunological disease	Allergy	7.01 × 10^−14^		AHCY, AHNAK, ALDOA, ANXA1, ANXA5, CAPG, CAPN1, CAPZB, CD44, CFL1, CNN3, CPNE1, DPYSL2, EEF1A1, ENO1, FKBP1A, FKBP4, FLNA, GAPDH, H3F3A/H3F3B, HN1, HNRNPR, HSPA5, IDI1, KRT1, LGALS1, MSN, MYH9, P4HB, PDIA3, PHGDH, PPIA, PRDX1, STAT1, TFRC, TKT, TPI1, TPM3, TUBB, VCL, VCP, VDAC2, XRCC6, ZYX	44
Cellular movement	Cell movement of tumor cell lines	7.21 × 10^−14^	0.345	ACTN4, ANXA1, ANXA2, ARF1, ARPC1B, ARPC2, BSG, C1QBP, CALR, CAP1, CAPN1, CAPN2, CAPNS1, CAV1, CD44, CSE1L, CTSB, CTTN, CYR61, DPYSL2, ENAH, EZR, FLNA, FLNB, FLNC, FN1, GDI1, GNB2L1, GPI, HDGF, HNRNPA2B1, HNRNPK, HSP90AA1, ILF3, IQGAP1, ITGB1, KHDRBS1, KPNA2, KRT8, LASP1, LGALS1, MAPK1, MSN, MYH10, MYH9, NCL, PA2G4, PEBP1, PHB2, PPIA, PRDX2, PRMT5, RHOA, SEPT9, SFN, SQSTM1, STAT1, STMN1, TAGLN2, TLN1, TNC, TPM3, VCL, VCP, VIM, YBX1, ZYX	67
Cell death and survival	Cell survival	1.06 × 10^−13^	−1.997	ACLY, ANXA5, AP2B1, APEX1, ATP5H, B2M, CALR, CAPN2, CAV1, CD44, CD59, CDK1, COPB2, CSE1L, CTSB, CYR61, DDX3X, DDX5, DHX9, DNM1L, EEF2, EIF2S1, EIF3C, EIF4A1, EIF4G1, EZR, FLNA, FN1, GLUD1, GNB2L1, GSTP1, HDGF, HINT1, HMGB1, HNRNPU, HSD17B10, HSP90AB1, HSP90B1, HSPA4, HSPA5, HSPB1, HSPD1, HYOU1, IGF2R, IQGAP1, ITGB1, LDHA, LMNA, MAPK1, MVP, P4HB, PARK7, PCNA, PDIA3, PEA15, PKM, PPIA, PPM1G, PPP1CA, PPP2CA, PPP2R1A, PRDX2, PRKDC, PRPF19, PSMA1, PSMA4, PSMA7, PSMC4, RAB11A, RHOA, RPL27, RPSA, S100A6, SET, SF3A1, SFN, SHMT2, SND1, SNRPD1, SOD1, SQSTM1, STAT1, STMN1, TCP1, TRIM28, TUBB, TXNDC5, UCHL1, VCL, VCP, VDAC1, VIM, XPO1, XRCC5, YBX1, YWHAZ	96
Cellular movement	Migration of cells	1.22 × 10^−13^	−2.069	ACTN4, ACTR3, AHCY, ALDOA, ANXA1, ANXA2, ANXA5, ARF1, ARHGDIA, ARPC2, BSG, C1QBP, CALR, CAP1, CAPG, CAPN2, CAPNS1, CAST, CAV1, CD44, CDK1, CFL1, CLIC4, CORO1C, CRIP2, CSE1L, CTNNA1, CTSB, CTTN, CYR61, DBN1, DDX3X, DPYSL2, EHD1, EZR, FASN, FKBP4, FLNA, FLNB, FLNC, FN1, G6PD, GNAI2, GNB2L1, GPI, HARS, HDGF, HLA-A, HMGB1, HNRNPA2B1, HNRNPK, HNRNPL, HSP90AA1, HSP90AB1, HSP90B1, HSPA5, HSPB1, HSPD1, HYOU1, IGF2R, ILF3, IQGAP1, ITGB1, KHDRBS1, KPNA2, KRT8, LAMB1, LASP1, LGALS1, LMNA, LMNB1, MAP1B, MAPK1, MAPRE1, MARCKS, MCM3, MCM7, MSN, MYH10, MYH9, MYL12A, NACA, NCL, NPM1, PA2G4, PARK7, PDCD6, PFN1, PHB2, PKM, PLEC, PPIA, PRDX1, PRDX2, PRMT5, PTMA, RHOA, RNH1, RPSA, RTN4, S100A6, SEPT7, SFN, SOD1, STAT1, STMN1, TARS, TLN1, TMOD3, TNC, TPM3, VCL, VCP, VIM, WARS, YBX1, YWHAE, YWHAZ, ZYX	119
Gene expression, protein synthesis	Translation of mRNA	2.07 × 10^−13^	1.632	CALR, CAPRIN1, DDX3X, EEF1B2, EIF2S1, EIF3B, EIF3C, EIF3I, EIF3M, EIF4G1, EIF4H, EPRS, GAPDH, GNB2L1, HSPB1, ILF3, NACA, PPP1CA, RBM3, RPL23, RPS24, RPS3A, RPS4X, RPS5, RRBP1, SYNCRIP, WARS	27
Cancer, respiratory disease	Respiratory system tumor	3.89 × 10^−13^	0.316	ACLY, AHNAK, AKR1B1, ALDOA, ALDOC, ALYREF, ANXA1, ANXA2, APEX1, ATIC, B2M, CAPRIN1, CAV1, CD44, CDK1, CYR61, DHX9, EEF1A1, EEF1B2, EIF4A1, ENAH, ENO1, ENO2, EZR, FASN, FDPS, FN1, G3BP1, GNB2L1, GPI, GSTP1, H2AFY, HIST2H2AC, HLA-A, HSP90AA1, HSP90AB1, HSP90B1, HSPD1, IMPDH2, ITGB1, KRT8, LDHA, LGALS1, LOC102724594/U2AF1, MAP4, MSN, MYH9, MYL12A, NACA, NCL, NSFL1C, PABPC1, PCNA, PDCD6, PFKP, PKM, PPIA, PRDX1, PRDX2, PRPF19, RHOA, RPL27, RPL7, RPS11, RPS27A, SERBP1, SFN, SHMT2, SND1, SQSTM1, STAT1, STMN1, TNC, TPI1, TUBB, TUBB4B, VIM, YWHAE, ZYX	79
Cancer	Malignant neoplasm of thorax	4.70 × 10^−13^	0.949	ACLY, AHNAK, ALDOA, ALDOC, ANXA2, APEX1, ATIC, B2M, CAPRIN1, CAV1, CD44, CDK1, CYR61, DHX9, EEF1A1, EEF1B2, EIF4A1, ENAH, ENO1, ENO2, EZR, FASN, FDPS, FN1, G3BP1, GNB2L1, GPI, GSTP1, H2AFY, HIST2H2AC, HLA-A, HSP90AA1, HSP90AB1, HSP90B1, HSPD1, IMPDH2, ITGB1, KRT8, LDHA, LGALS1, LOC102724594/U2AF1, MAP4, MSN, MYH9, MYL12A, NACA, NCL, NSFL1C, PABPC1, PCNA, PDCD6, PFKP, PKM, PPIA, PRDX1, PRDX2, PRKDC, PRPF19, RHOA, RPL27, RPL7, RPS11, RPS27A, SERBP1, SHMT2, SND1, SQSTM1, STAT1, STMN1, TPI1, TUBB, TUBB4B, VIM, XRCC5, XRCC6, YWHAE	76
Gene expression	Expression of mRNA	6.12 × 10^−13^	0.865	CALR, CAPRIN1, DDX3X, EEF1B2, EIF2S1, EIF3B, EIF3C, EIF3I, EIF3M, EIF4G1, EIF4H, EPRS, GAPDH, GNB2L1, HMGB1, HSPB1, ILF3, KHDRBS1, NACA, PFN1, PPP1CA, RBM3, RPL23, RPS24, RPS3A, RPS4X, RPS5, RRBP1, STAT1, SYNCRIP, WARS	31
Cell morphology, cellular assembly and organization, cellular function and maintenance	Formation of cellular protrusions	6.14 × 10^−13^	−0.837	ACTN4, ACTR2, ACTR3, ARPC2, BASP1, BSG, C1QBP, CAP1, CAPG, CAPN2, CAPNS1, CAPRIN1, CAPZB, CAST, CAV1, CD44, CFL1, CSRP1, CTTN, CYR61, DBN1, DNM1L, DPYSL2, EEF1A1, EHD1, ENAH, EZR, FASN, FLNA, FN1, GDI1, HMGB1, HNRNPK, HSP90AA1, HSPB1, IQGAP1, ITGB1, KIF5B, LAMB1, LASP1, MAP1B, MARCKS, MSN, MYH10, NNMT, PCM1, PDIA3, PFN1, PHGDH, PICALM, PLEC, PPP2CA, PRKCSH, RAB11A, RANBP1, RHOA, RTN4, SEPT2, SOD1, SPTBN1, STIP1, STMN1, TNC, TPM3, TWF2, UCHL1, VAPA, VCL, VIM, YWHAH	70
Cancer, respiratory disease	Lung tumor	7.06 × 10^−13^	0.316	ACLY, AHNAK, AKR1B1, ALDOA, ALDOC, ALYREF, ANXA2, APEX1, ATIC, B2M, CAPRIN1, CAV1, CD44, CDK1, CYR61, DHX9, EEF1A1, EEF1B2, EIF4A1, ENAH, ENO1, ENO2, EZR, FASN, FDPS, FN1, G3BP1, GNB2L1, GPI, GSTP1, H2AFY, HIST2H2AC, HLA-A, HSP90AA1, HSP90AB1, HSP90B1, HSPD1, IMPDH2, ITGB1, KRT8, LDHA, LGALS1, LOC102724594/U2AF1, MAP4, MSN, MYH9, MYL12A, NACA, NCL, NSFL1C, PABPC1, PCNA, PDCD6, PFKP, PKM, PPIA, PRDX1, PRDX2, PRPF19, RHOA, RPL27, RPL7, RPS11, RPS27A, SERBP1, SHMT2, SND1, SQSTM1, STAT1, STMN1, TNC, TPI1, TUBB, TUBB4B, VIM, YWHAE, ZYX	77
Organismal survival	Organismal death	9.82 × 10^−13^	2.742	ACLY, ACTA1, ACTG1, ACTN4, AIP, ANXA1, APEX1, ARHGDIA, ATP1A1, B2M, BSG, BUB3, C1QBP, CALR, CANX, CAP1, CAPN1, CAPN2, CAPNS1, CAPRIN1, CAPZB, CAST, CAV1, CD44, CD59, CDK1, CFL1, CLIC4, CSE1L, CTSA, CTSB, CTTN, CYR61, DBI, DDX1, DDX5, DHX9, DNM1L, DYNC1H1, EHD1, EIF2S1, EIF3M, EIF4A1, EIF6, ENAH, FASN, FKBP1A, FKBP4, FLNA, FLNB, FLNC, FN1, FUS, G6PD, GNAI2, GSTP1, H3F3A/H3F3B, HADHA, HEXB, HIST1H1C, HMGB1, HNRNPK, HSP90AA1, HSP90AB1, HSP90B1, HSPA5, HYOU1, IGF2R, ILF2, ILF3, IMPDH2, ITGB1, KHDRBS1, KIF5B, KRT1, KRT8, LMNA, LMNB1, LRPPRC, MAP1B, MAPK1, MSN, MYH10, MYH9, NASP, NPM1, NUMA1, PCNA, PDIA3, PEA15, PFN1, PHB2, PHGDH, PICALM, PLEC, PLS3, PPIA, PPP2CA, PRDX1, PRKDC, PRMT5, PRPF19, PSMC1, PSMC4, PTBP1, PTGES3, RAD23B, RHOA, RPL4, RPL6, RPSA, RTN4, SERPINH1, SF3B1, SHMT2, SOD1, SPTBN1, SQSTM1, SSRP1, STAT1, STIP1, TFRC, TKT, TLN1, TPM3, TPP1, TRIM28, UBE2L3, UBE2N, VCL, VCP, VDAC1, VIM, WDR1, XRCC5, XRCC6, YBX1, YBX3, YWHAE	139
Infectious disease, renal and urological disease	Infection of kidney cell lines	1.19 × 10^−12^	−1.281	BSG, CTSB, GANAB, HMGB1, HNRNPH1, HSPA5, IFITM3, KHDRBS1, KPNB1, KRT18, MAP4, PCBP2, PGM1, PLOD2, PSMA1, PSMA2, PSMA5, PSMA7, PSMC4, PTGES3, RNH1, RPL10A, RPL12, RPL18, RPL3, RPL5, SF3A1, SF3B1, SNRPD3, TAGLN2, TFRC, UBE2L3, YBX1, ZYX	34
Inflammatory disease, skeletal and muscular disorders	Inclusion body myopathy	2.03 × 10^−12^		CALR, CANX, HNRNPA1, HNRNPA2B1, HSP90B1, HSPA5, PSMA2, PSMA4, SQSTM1, VCP	10
Cancer, respiratory disease	Lung cancer	2.29 × 10^−12^	0.205	ACLY, AHNAK, ALDOA, ALDOC, ANXA2, APEX1, ATIC, B2M, CAPRIN1, CAV1, CD44, CDK1, CYR61, DHX9, EEF1A1, EEF1B2, EIF4A1, ENAH, ENO1, ENO2, EZR, FASN, FDPS, FN1, G3BP1, GNB2L1, GPI, GSTP1, H2AFY, HIST2H2AC, HLA-A, HSP90AA1, HSP90AB1, HSP90B1, HSPD1, IMPDH2, ITGB1, KRT8, LDHA, LGALS1, LOC102724594/U2AF1, MAP4, MSN, MYH9, MYL12A, NACA, NCL, NSFL1C, PABPC1, PCNA, PDCD6, PFKP, PKM, PPIA, PRDX1, PRDX2, PRPF19, RHOA, RPL27, RPL7, RPS11, RPS27A, SERBP1, SHMT2, SND1, SQSTM1, STAT1, STMN1, TPI1, TUBB, TUBB4B, VIM, YWHAE	73
Dermatological diseases and conditions, immunological disease, inflammatory disease, inflammatory response	Atopic dermatitis	2.95 × 10^−12^		AHCY, AHNAK, ANXA1, ANXA5, CAPG, CAPZB, CD44, CFL1, CNN3, CPNE1, DPYSL2, EEF1A1, ENO1, FKBP1A, FKBP4, FLNA, H3F3A/H3F3B, HN1, HNRNPR, IDI1, KRT1, LGALS1, MSN, PHGDH, STAT1, TPI1, TPM3, VCL, VCP, VDAC2, XRCC6, ZYX	32
Developmental disorder, hereditary disorder, skeletal and muscular disorders	Distal myopathy	4.20 × 10^−12^		CALR, CANX, FLNC, HSP90B1, HSPA5, MATR3, PSMA2, PSMA4, SQSTM1, VCP	10
RNA post-transcriptional modification	Processing of RNA	4.45 × 10^−12^	0.640	AARS, AHNAK, C1QBP, DDX39B, DDX5, FUS, HNRNPA1, HNRNPA2B1, HNRNPH1, HNRNPH3, HNRNPK, HNRNPL, HNRNPM, HNRNPU, KHDRBS1, KHSRP, LOC102724594/U2AF1, NPM1, PABPC1, PCBP1, PRPF19, PTBP1, RBM3, RPL5, RPL7, RPS15, RPS24, RPS7, SF3A1, SF3B1, SFPQ, SNRPD1, SYNCRIP, U2AF2	34
Nucleic acid metabolism	Metabolism of nucleic acid component or derivative	7.96 × 10^-12^	−1.213	ACLY, AHCY, ALDOA, ANXA1, ATIC, ATP1A1, ATP5A1, ATP5B, ATP5H, CAV1, CCT8, CDK1, CS, CTPS1, DBI, DDX1, DDX39B, DDX3X, DDX5, DHX9, DNM1L, DPYSL2, FASN, FDPS, G3BP1, G6PD, GMPS, GNAI2, HMGB1, HSP90AA1, HSPA8, HSPD1, IMPDH2, LONP1, MAPK1, MCM7, MDH1, MDH2, MYH10, MYH9, NNMT, OLA1, PGK1, PKM, PPA1, PSMC1, PSMC4, SET, SLC25A5, SOD1, TALDO1, UAP1, VCP, VDAC1	54
Infectious disease, organismal injury and abnormalities	Infection of embryonic cell lines	1.06 × 10^-11^	−1.045	BSG, GANAB, HMGB1, HNRNPH1, HSPA5, IFITM3, KHDRBS1, KPNB1, MAP4, PCBP2, PGM1, PLOD2, PSMA1, PSMA2, PSMA5, PSMA7, PSMC4, PTGES3, RNH1, RPL10A, RPL12, RPL18, RPL3, RPL5, SF3A1, SF3B1, SNRPD3, TAGLN2, TFRC, UBE2L3, YBX1, ZYX	32
Infectious disease	Infection of epithelial cell lines	1.06 × 10^-11^	−1.045	BSG, GANAB, HMGB1, HNRNPH1, HSPA5, IFITM3, KHDRBS1, KPNB1, MAP4, PCBP2, PGM1, PLOD2, PSMA1, PSMA2, PSMA5, PSMA7, PSMC4, PTGES3, RNH1, RPL10A, RPL12, RPL18, RPL3, RPL5, SF3A1, SF3B1, SNRPD3, TAGLN2, TFRC, UBE2L3, YBX1, ZYX	32
Cellular movement	Invasion of cells	1.64 × 10^-11^	−0.711	ACAT1, AHCY, ANXA1, ANXA2, APEX1, ARHGDIA, BSG, C1QBP, CALR, CAP1, CAPN2, CAPNS1, CAV1, CD44, CDK1, CSE1L, CTSB, CTTN, CYR61, ENAH, EZR, FKBP1A, FLNA, FN1, GNAI2, GNB2L1, GPI, HDGF, HDLBP, HMGB1, HSP90AA1, HSP90AB1, HSP90B1, HSPA5, ILF3, IQGAP1, ITGB1, KRT8, LASP1, LGALS1, MAPK1, MARCKS, MYH10, NPM1, OTUB1, PA2G4, PARK7, PEBP1, PICALM, PKM, PTGES3, RHOA, RNH1, RPSA, S100A6, SEPT9, SQSTM1, STMN1, TAGLN, TAGLN2, VCP, VIM, YWHAQ	63
Cell death and survival	Cell viability	3.09 × 10^-11^	−1.538	ACLY, ANXA5, AP2B1, APEX1, ATP5H, B2M, CALR, CAPN2, CAV1, CD44, CD59, CDK1, COPB2, CTSB, CYR61, DDX5, DHX9, DNM1L, EEF2, EIF3C, EIF4A1, EIF4G1, EZR, FLNA, FN1, GLUD1, GNB2L1, GSTP1, HDGF, HINT1, HMGB1, HNRNPU, HSD17B10, HSP90AB1, HSP90B1, HSPA5, HSPB1, HSPD1, HYOU1, IGF2R, IQGAP1, ITGB1, LDHA, LMNA, MAPK1, MVP, P4HB, PARK7, PCNA, PDIA3, PPIA, PPM1G, PPP1CA, PPP2CA, PPP2R1A, PRDX2, PRKDC, PRPF19, PSMA1, PSMA4, PSMC4, RAB11A, RHOA, RPL27, RPSA, S100A6, SF3A1, SFN, SHMT2, SND1, SNRPD1, SOD1, SQSTM1, STAT1, STMN1, TCP1, TRIM28, TXNDC5, UCHL1, VCP, VDAC1, XPO1, XRCC5, YBX1, YWHAZ	85
Cancer, organismal injury and abnormalities, reproductive system disease	Cervical tumor	3.68 × 10^-11^		ACTR3, ALYREF, ANXA1, ANXA2, ANXA5, ATIC, CTSB, ENO1, GSTP1, H2AFY, HIST1H2BL, HIST2H2AC, HLA-A, HLA-B, HSP90AA1, HSP90AB1, HSP90B1, HSPB1, KRT1, KRT9, MAP4, MCM7, RPS12, TAGLN, TFRC, TPM2, TPM3, TUBB, TUBB4B, XPO1, YWHAE	31
Developmental disorder, hereditary disorder, inflammatory disease, neurological disease, skeletal and muscular disorders	Nonaka myopathy	4.55 × 10^-11^		CALR, CANX, HSP90B1, HSPA5, PSMA2, PSMA4, SQSTM1, VCP	8
Dermatological diseases and conditions, inflammatory disease, inflammatory response	Dermatitis	5.49 × 10^-11^		AHCY, AHNAK, ANXA1, ANXA5, ARHGDIA, ATP1A1, CALR, CAPG, CAPZB, CD44, CFL1, CNN3, CPNE1, DPYSL2, EEF1A1, ENO1, FKBP1A, FKBP4, FLNA, GSTP1, H3F3A/H3F3B, HN1, HNRNPR, IDI1, KRT1, LGALS1, MSN, PHGDH, SFN, STAT1, TPI1, TPM3, TUBB, TUBB4B, TUBB6, TUBB8, VCL, VCP, VDAC2, XRCC6, ZYX	41
Infectious disease	Replication of influenza A virus	6.00 × 10^-11^	−0.322	ANXA6, B2M, C14orf166, CLIC4, COPA, COPB1, COPB2, COPG1, CSE1L, EEF1A1, EIF3C, FDPS, HSP90AA1, HSPD1, IFITM3, ILF3, KPNB1, LONP1, MAPK1, PSMA1, PSMD2, RPS10, RPS27A, RPS5, RPSA, SAE1, SF3A1, SF3B1, SFPQ, STAT1, TUBB, XPO1	32
Cell cycle	M phase	6.05 × 10^-11^	−0.578	ACTN4, BUB3, CAP1, CAPN2, CDK1, CFL1, FLNA, FN1, GNAI2, ITGB1, LMNA, MAPRE1, MCM4, MCM7, MSN, MYH10, MYH9, NPM1, NUMA1, PFN1, RAB11A, RHOA, SEPT2, SEPT7, SEPT9, SSRP1, XRCC5, YBX1	28
DNA replication, recombination, and repair, energy production, nucleic acid metabolism, small molecule biochemistry	Catabolism of ATP	7.68 × 10^-11^		ACLY, ANXA1, ATP1A1, ATP5A1, ATP5B, ATP5H, CCT8, DDX1, DDX39B, DDX3X, DDX5, DHX9, G3BP1, HSP90AA1, HSPA8, LONP1, MCM7, MYH10, MYH9, OLA1, PSMC1, PSMC4, VCP	23
Cell death and survival	Cell death of neuroblastoma cell lines	1.35 × 10^-10^	−1.206	ATP5A1, CAPN1, CAPN2, CAST, CCT2, CCT3, CCT4, CCT5, CCT6A, CCT7, CCT8, ENO1, FKBP1A, GAPDH, HSPA8, ITGB1, PARK7, PEBP1, PRDX2, S100A6, SOD1, SQSTM1, TCP1, YWHAE	24
Hematological disease, immunological disease, inflammatory disease, inflammatory response, respiratory disease	Allergic pulmonary eosinophilia	1.55 × 10^-10^		ALDOA, ENO1, GAPDH, HSPA5, MYH9, P4HB, PDIA3, PRDX1, TKT, TPI1, TUBB	11
Cell morphology	Cell spreading	1.72 × 10^-10^	−0.739	CAP1, CAPN2, CAST, CD44, CYR61, EHD1, FLNA, FLNB, FLNC, FN1, GNB2L1, IGF2R, IPO9, ITGB1, LGALS1, MAP4, MAPK1, MARCKS, PFN1, PTBP1, RHOA, RRBP1, RTN4, TLN1, TNC, UCHL1, VCL, VIM, YWHAZ, ZYX	30
Skeletal and muscular disorders	Myopathy	1.81 × 10^-10^	−2.236	AARS, ACTA1, AHNAK, ATP1A1, BUB3, CALR, CANX, CAST, DYNC1H1, ECHS1, FKBP1A, FLNC, FN1, GARS, HARS, HEXB, HINT1, HLA-A, HMGB1, HNRNPA1, HNRNPA2B1, HSP90B1, HSPA5, HSPB1, ITGB1, LMNA, LRPPRC, MATR3, PLEC, PPP1CA, PPP2CA, PSMA2, PSMA4, RAB7A, RHOA, SDHA, SOD1, SQSTM1, STMN1, TPM2, TPM3, TUBB, TUBB4B, UBA1, VCL, VCP	46
Molecular transport, protein trafficking	Internalization of protein	2.17 × 10^-10^	1.900	AP1B1, AP2B1, CAP1, CFL1, CLTC, DNAJA1, DNAJA2, IPO5, IPO7, IPO9, KPNA2, KPNB1, PDIA3, RAN, RHOA, SPTBN1, XPO1	17
Cell death and survival	Cell death of cervical cancer cell lines	2.24 × 10^-10^	−0.340	ATP1A1, CALR, CCT4, CDK1, CTSB, DNM1L, DYNC1H1, EZR, FLNB, HNRNPK, IMMT, KHDRBS1, KPNB1, KRT18, LMNA, MAPK1, MSN, PCBP2, PKM, PPIA, PPP2R1A, PRDX2, PRKDC, PRPF19, PTMA, RPS24, SOD1, SQSTM1, STAT1, TCP1, UCHL1, VCP, VDAC1, YWHAH	34
Cellular development, cellular growth and proliferation, connective tissue development and function	Proliferation of fibroblast cell lines	2.34 × 10^-10^	−0.983	ACAT1, AKR1B1, ALDOA, APEX1, ATIC, CAPRIN1, CAST, CAV1, CLTC, DDX3X, DPY30, EEF1D, EIF3B, EIF3C, EIF3I, EIF6, FN1, G6PD, GNAI2, GNB2L1, HDGF, HINT1, HMGB1, ITGB1, KHDRBS1, LMNA, MAPK1, NPM1, PRPF19, PSMA4, PTMA, RHOA, S100A6, STAT1, STIP1, STMN1, TFRC, TPM3, XRCC6	39
Cancer, respiratory disease	Stage 1-2 non-small cell lung cancer	2.36 × 10^-10^		ANXA2, B2M, EEF1A1, EIF4A1, FN1, MYL12A, PPIA, RPL27, RPL7, RPS11, RPS27A, SERBP1	12
Immunological disease	Immediate hypersensitivity	2.58 × 10^-10^		AHCY, AHNAK, ANXA1, ANXA5, CAPG, CAPZB, CD44, CFL1, CNN3, CPNE1, DPYSL2, EEF1A1, ENO1, FKBP1A, FKBP4, FLNA, H3F3A/H3F3B, HN1, HNRNPR, IDI1, KRT1, LGALS1, MSN, PHGDH, PPIA, STAT1, TPI1, TPM3, VCL, VCP, VDAC2, XRCC6, ZYX	33
DNA replication, recombination, and repair	Metabolism of DNA	2.73 × 10^-10^	−1.611	APEX1, BSG, CACYBP, CALR, CAPN2, CAST, CAV1, CDK1, DHX9, ENO1, HMGB1, HNRNPA1, HSD17B10, HSPB1, KPNA2, KRT8, LMNA, MAPK1, MCM7, NAP1L1, NASP, NCL, NPM1, PCM1, PCNA, PEA15, PFN1, PPIA, RAN, RHOA, SET, SOD1, SSRP1, STAT1, SUPT16H, TMPO, XRCC5, XRCC6	38
Cellular assembly and organization	Organization of organelle	3.33 × 10^-10^	−1.732	ALDOA, ANXA2, CAPZB, CAV1, CD44, CFL1, CLTC, CTNNA1, DBN1, DNM1L, DPYSL2, ENAH, FKBP1A, FLNA, FN1, GAPDH, H3F3A/H3F3B, HEXB, ITGB1, KRT18, KRT9, LMNA, LONP1, MAPRE1, MSN, MYH9, NPLOC4, NPM1, NSFL1C, NUMA1, PARK7, PCM1, PLS3, RHOA, RRBP1, SERPINH1, SOD1, SPTBN1, STOML2, SURF4, TPP1, TUBB, VCL, VIM	44
Cancer, respiratory disease	Carcinoma in lung	3.34 × 10^-10^	0.555	AHNAK, ALDOA, ALDOC, ANXA2, APEX1, ATIC, B2M, CAPRIN1, CAV1, CD44, CDK1, DHX9, EEF1A1, EEF1B2, EIF4A1, ENO1, ENO2, EZR, FASN, FDPS, FN1, G3BP1, GNB2L1, GPI, GSTP1, HIST2H2AC, HLA-A, HSP90AA1, HSP90AB1, HSP90B1, HSPD1, IMPDH2, ITGB1, LDHA, LOC102724594/U2AF1, MAP4, MSN, MYH9, MYL12A, NACA, NSFL1C, PABPC1, PCNA, PDCD6, PFKP, PKM, PPIA, PRDX1, PRPF19, RPL27, RPL7, RPS11, RPS27A, SERBP1, STAT1, STMN1, TPI1, TUBB, TUBB4B, VIM, YWHAE	61
Nucleic acid metabolism, small molecule biochemistry	Metabolism of nucleotide	4.07 × 10^-10^	−1.647	ACLY, ALDOA, ANXA1, ATP1A1, ATP5A1, ATP5B, ATP5H, CAV1, CCT8, CDK1, CTPS1, DDX1, DDX39B, DDX3X, DDX5, DHX9, DNM1L, FASN, FDPS, G3BP1, G6PD, GMPS, GNAI2, HMGB1, HSP90AA1, HSPA8, HSPD1, IMPDH2, LONP1, MCM7, MDH1, MDH2, MYH10, MYH9, NNMT, OLA1, PKM, PPA1, PSMC1, PSMC4, SET, SLC25A5, SOD1, TALDO1, VCP, VDAC1	46
DNA replication, recombination, and repair, nucleic acid metabolism, small molecule biochemistry	Hydrolysis of nucleotide	4.19 × 10^-10^	−1.306	ATP1A1, CALR, CCT4, CCT5, CDK1, DNM1L, GNAI2, HMGB1, HSPA5, HSPD1, IPO5, MAPK1, RAB7A, RAN, RANBP1, RHOA, STMN1, UBA1, XPO1	19
Infectious disease	HIV infection	4.22 × 10^-10^	−1.958	ANXA2, ARF1, ATP5B, B2M, CCT2, CD44, DDX3X, DHX9, DNAJA2, FDPS, FN1, GANAB, GML, GPI, H3F3A/H3F3B, HMGB1, HNRNPH1, HNRNPU, IMPDH2, ITGB1, KHDRBS1, KPNB1, MAP4, PDIA3, PDIA6, PGM1, PLOD2, PSMA1, PSMA2, PSMA5, PSMA7, PSMC4, PSME2, PTGES3, RAB1B, RANBP1, RNH1, RPL10A, RPL12, RPL18, SF3A1, SF3B1, SNRPD3, SPTAN1, SPTBN1, STIP1, TAGLN2, TFRC, TWF1, UAP1, UBE2L3, UQCRC1, XPO1, YBX1, ZYX	55
Nucleic acid metabolism, small molecule biochemistry	Metabolism of purine nucleotide	4.60 × 10^-10^		ACLY, ANXA1, ATP1A1, ATP5A1, ATP5B, ATP5H, CCT8, DDX1, DDX39B, DDX3X, DDX5, DHX9, G3BP1, G6PD, HSP90AA1, HSPA8, LONP1, MCM7, MDH1, MDH2, MYH10, MYH9, OLA1, PSMC1, PSMC4, VCP	26
Cancer, respiratory disease	Non-small cell lung cancer	4.91 × 10^-10^		AHNAK, ALDOA, ALDOC, ANXA2, APEX1, ATIC, B2M, CAPRIN1, CAV1, CD44, CDK1, DHX9, EEF1A1, EEF1B2, EIF4A1, ENO1, EZR, FASN, FDPS, FN1, G3BP1, GNB2L1, GPI, GSTP1, HIST2H2AC, HLA-A, HSP90AA1, HSP90AB1, HSP90B1, HSPD1, IMPDH2, ITGB1, LDHA, LOC102724594/U2AF1, MAP4, MSN, MYH9, MYL12A, NACA, NSFL1C, PABPC1, PCNA, PFKP, PKM, PPIA, PRDX1, PRPF19, RPL27, RPL7, RPS11, RPS27A, SERBP1, STAT1, STMN1, TPI1, TUBB, TUBB4B, VIM	58
Immunological disease	Hypersensitive reaction	5.39 × 10^-10^	0.404	AHCY, AHNAK, ANXA1, ANXA5, CALR, CAPG, CAPZB, CD44, CFL1, CNN3, CPNE1, DPYSL2, EEF1A1, ENO1, FKBP1A, FKBP4, FLNA, GARS, H3F3A/H3F3B, HLA-A, HLA-B, HN1, HNRNPR, IDI1, KRT1, LGALS1, MSN, PHGDH, PPIA, PSME2, SOD1, STAT1, TPI1, TPM3, VCL, VCP, VDAC2, XRCC6, ZYX	39
Cancer, endocrine system disorders, organismal injury and abnormalities	Benign cold thyroid nodule	1.09 × 10^-9^		ANXA5, CALR, CTSB, GSTP1, HSP90AB1, PARK7, PRDX2, PRDX5	8
Cancer, organismal injury and abnormalities, reproductive system disease	Cervical cancer	1.13 × 10^-9^		ACTR3, ANXA1, ANXA2, ANXA5, ATIC, CTSB, ENO1, GSTP1, H2AFY, HIST1H2BL, HIST2H2AC, HSP90AA1, HSP90AB1, HSP90B1, HSPB1, KRT1, KRT9, MAP4, MCM7, RPS12, TAGLN, TFRC, TPM2, TPM3, TUBB, TUBB4B, XPO1, YWHAE	28
Cellular assembly and organization, cellular function and maintenance	Organization of filaments	1.22 × 10^-9^	−1.929	ALDOA, ANXA2, CFL1, DBN1, DPYSL2, ENAH, FKBP1A, FLNA, FN1, GAPDH, ITGB1, KRT18, KRT9, MAPRE1, MSN, NUMA1, PCM1, PLS3, RHOA, RRBP1, SERPINH1, SOD1, TUBB, VIM	24
Cancer, respiratory disease	Pulmonary metastasis	1.30 × 10^-9^		ATIC, B2M, EEF1A1, EIF4A1, FN1, MYL12A, PPIA, RHOA, RPL27, RPL7, RPS11, RPS27A, YWHAE	13
Cellular assembly and organization	Stabilization of filaments	1.55 × 10^-9^	−0.454	CFL1, COPB2, CTTN, DNM1L, DPYSL2, FLNA, IQGAP1, KRT18, KRT8, MAP1B, MAP4, MAPRE1, NUMA1, PKM, RHOA, SEPT7, STMN1	17
Cancer, respiratory disease	Metastatic lung carcinoma	1.60 × 10^-9^		ATIC, B2M, EEF1A1, EIF4A1, FN1, MYL12A, PPIA, RPL27, RPL7, RPS11, RPS27A, YWHAE	12
Cancer	Metastasis	1.88 × 10^-9^	−1.645	ANXA1, ANXA5, ATIC, B2M, C1QBP, CAPG, CAPN2, CAV1, CD44, CSE1L, CTSB, CTTN, CYR61, DPYSL2, EEF1A1, EIF4A1, ENAH, EZR, FASN, FDPS, FKBP1A, FLNA, FN1, FUS, GNB2L1, HMGB1, HSP90AA1, HSP90AB1, HSP90B1, HYOU1, ITGB1, KHDRBS1, KRT18, KRT8, LGALS1, MAPK1, MYL12A, PLOD2, PPIA, PRDX2, RHOA, RNH1, RPL27, RPL7, RPS11, RPS27A, SND1, SQSTM1, STAT1, TUBB, TUBB4B, UBE2N, VIM, YWHAE, YWHAZ	55
Cellular movement	Migration of tumor cell lines	1.96 × 10^-9^	−0.054	ACTN4, ANXA1, ANXA2, ARF1, ARPC2, BSG, C1QBP, CAPN2, CAPNS1, CAV1, CD44, CSE1L, CTTN, CYR61, DPYSL2, EZR, FLNA, FLNB, FLNC, FN1, GNB2L1, HDGF, HNRNPA2B1, HNRNPK, HSP90AA1, ILF3, IQGAP1, ITGB1, KHDRBS1, KPNA2, KRT8, LASP1, LGALS1, MAPK1, MSN, MYH10, MYH9, NCL, PHB2, PRDX2, PRMT5, RHOA, SFN, STAT1, STMN1, TNC, TPM3, VCP, VIM, ZYX	50
Cell morphology, connective tissue development and function	Cell spreading of fibroblast cell lines	2.05 × 10^-9^	0.201	CAST, EHD1, FLNA, FLNC, FN1, GNB2L1, IPO9, ITGB1, PTBP1, RTN4, VCL, VIM	12
Cancer, respiratory disease	Metastatic lung cancer	2.05 × 10^-9^		ATIC, B2M, EEF1A1, EIF4A1, FN1, MYL12A, PPIA, RPL27, RPL7, RPS11, RPS27A, YWHAE	12
Gene expression	Expression of RNA	2.21 × 10^-9^	1.310	ACTR2, ACTR3, ALYREF, BASP1, BTF3, C14orf166, C1QBP, CALR, CAND1, CAPRIN1, CAV1, CBX3, CD44, CDK1, CSE1L, CYR61, DDX3X, DDX5, DHX9, EEF1B2, EEF1D, EEF2, EIF2S1, EIF3B, EIF3C, EIF3I, EIF3M, EIF4G1, EIF4H, ENO1, EPRS, EZR, FKBP1A, FLNA, FN1, FUBP3, GAPDH, GLO1, GNB2L1, GSTP1, H2AFY, HDGF, HEXB, HINT1, HIST1H1C, HMGB1, HNRNPA1, HNRNPA2B1, HNRNPK, HSPA4, HSPA8, HSPB1, ILF2, ILF3, IQGAP1, KHDRBS1, KPNA2, LGALS1, LMNA, LRPPRC, MAPK1, MATR3, MCM7, MSN, NACA, NONO, NPM1, OTUB1, PA2G4, PARK7, PCNA, PDLIM1, PEA15, PEBP1, PFN1, PHB2, PHGDH, PICALM, PPP1CA, PPP2CA, PRKDC, PRMT5, PTGES3, PTMA, PTRF, RBM3, RHOA, RPL23, RPL6, RPS24, RPS3A, RPS4X, RPS5, RRBP1, SET, SFPQ, SQSTM1, SSRP1, STAT1, SYNCRIP, TAGLN, TMPO, TNC, TRIM28, UBE2L3, VAPA, VIM, WARS, XPO1, XRCC5, XRCC6, YBX1, YBX3, YWHAB, YWHAH, YWHAQ, YWHAZ	117
Inflammatory response	Inflammation of organ	2.36 × 10^-9^	0.481	ACTN4, AHCY, AHNAK, ALDOA, ANXA1, ANXA5, ARHGDIA, ATP1A1, B2M, BSG, CALR, CAPG, CAPZB, CAV1, CD44, CFL1, CNN3, CPNE1, CTSB, CYR61, DDX5, DPYSL2, EEF1A1, ENO1, FKBP1A, FKBP4, FLNA, FUS, GAPDH, GNAI2, GSTP1, H3F3A/H3F3B, HARS, HLA-A, HMGB1, HN1, HNRNPR, HSP90B1, HSPA5, IDI1, IMPDH2, KRT1, KRT18, KRT8, LGALS1, MSN, MYH10, MYH9, P4HB, PDIA3, PDLIM1, PHGDH, PPIA, PPP2CA, PRDX1, PSMB1, PSMD2, SFN, SOD1, STAT1, TKT, TPI1, TPM3, TUBB, TUBB4B, TUBB6, TUBB8, VCL, VCP, VDAC2, VIM, XRCC6, YBX1, ZYX	74
Embryonic development, organismal survival	Death of embryo	2.51 × 10^-9^	0.942	BSG, CAPN2, CAPNS1, EIF6, FASN, IMPDH2, KIF5B, LRPPRC, MAP1B, NASP, PCNA, PFN1, PHB2, PRPF19, PSMC4, RPSA, SF3B1, TKT, TPM3, VCP	20
Cell cycle	Cell cycle progression	2.53 × 10^-9^	−1.218	BUB3, C1QBP, CALR, CAPN2, CAST, CAV1, CCT4, CD44, CD59, CDK1, CLTC, CSE1L, CYR61, DBI, DHX9, DYNC1H1, EIF6, EZR, FASN, FKBP1A, FLNA, GNB2L1, GPI, H3F3A/H3F3B, HMGB1, HSPA8, HSPB1, ITGB1, KHDRBS1, KPNB1, KRT18, KRT8, LMNA, MAP4, MAPK1, MARCKS, MCM7, MYH10, NASP, NPM1, NUMA1, PA2G4, PCM1, PCNA, PEBP1, PPM1G, PPP1CA, PPP2CA, PRDX1, PRMT5, PTMA, RAN, RBM3, RHOA, RPS24, SEPT9, SFN, SFPQ, SSRP1, STAT1, STMN1, TCP1, TMPO, TNC, TUBB, VCP, XRCC6, YWHAB, YWHAE	69
Cell morphology	Morphology of cells	2.61 × 10^-9^		ACTA1, ACTN4, ACTR2, ANXA1, ANXA2, ARF1, ARHGDIA, ARPC2, B2M, BUB3, CALR, CAP1, CAPN1, CAPN2, CAPRIN1, CAPZB, CAST, CAV1, CD44, CD59, CDK1, CFL1, CLIC4, CLTC, CSTB, CTSA, CTSB, CTTN, DNAJA1, DNM1L, DPY30, DPYSL2, EHD1, EIF6, EZR, FASN, FLNA, FLNC, FN1, GPI, H3F3A/H3F3B, HADH, HADHA, HEXB, HLA-A, HMGB1, HSP90AA1	110
				HSP90B1, HSPA4, IGF2R, ILF3, IMPDH2, IQGAP1, ITGB1, KIF5B, KRT1, KRT18, KRT8, LAMB1, LGALS1, LMNA, LMNB1, MAP1B, MAP4, MAPK1, MARCKS, MYH10, MYH9, NPEPPS, NPM1, PAFAH1B2, PEA15, PEBP1, PLEC, PLIN3, PLS3, PRDX1, PRDX2, PRKDC, PTBP1, RAB11A, RAD23B, RAN, RHOA, RPSA, RTN4, SEPT7, SEPT9, SERPINH1, SNX3, SOD1, SPTBN1, STAT1, STMN1, SYNCRIP, TALDO1, TFRC, TLN1, TMOD3, TNC, TPM3, TPP1, VCL, VIM, XRCC5, XRCC6, YBX1, YWHAG, YWHAZ, ZYX	
Cancer, respiratory disease	Stage 1 non-small-cell lung carcinoma	2.83 × 10^-9^		B2M, EEF1A1, EIF4A1, FN1, MYL12A, PPIA, RPL27, RPL7, RPS11, RPS27A, SERBP1	11
Cellular assembly and organization, tissue development	Formation of filaments	3.49 × 10^-9^	−0.462	ACTA1, ACTR3, ARF1, ARPC2, CAPN1, CAPZB, CAV1, CD44, CFL1, CTTN, DPYSL2, FKBP4, FN1, GNAI2, GPI, HSPA5, HSPB1, ITGB1, KRT18, MAP1B, MAPK1, MAPRE1, NPM1, PFN1, RHOA, SERPINH1, SOD1, STMN1, TCP1, TNC, TPM2, TUBB, TWF1, TWF2, VIM, ZYX	36
Infectious disease	Infection by HIV-1	4.09 × 10^-9^	−1.626	ARF1, ATP5B, CCT2, CD44, DDX3X, DHX9, DNAJA2, GANAB, GML, H3F3A/H3F3B, HMGB1, HNRNPH1, HNRNPU, ITGB1, KHDRBS1, KPNB1, MAP4, PDIA3, PDIA6, PGM1, PLOD2, PSMA1, PSMA2, PSMA5, PSMA7, PSMC4, PSME2, PTGES3, RAB1B, RANBP1, RNH1, RPL10A, RPL12, RPL18, SF3A1, SF3B1, SNRPD3, SPTAN1, SPTBN1, STIP1, TAGLN2, TWF1, UAP1, UBE2L3, UQCRC1, XPO1, YBX1, ZYX	48
Cancer	Malignant neoplasm of heart, mediastinum and pleura	4.65 × 10^-9^	1.982	ATIC, B2M, EEF1A1, EIF4A1, FN1, MYL12A, PPIA, PRDX1, PRKDC, RHOA, RPL27, RPL7, RPS11, RPS27A, TUBB, TUBB4B, XRCC5, XRCC6, YWHAE	19
Cancer	Growth of tumor	5.06 × 10^-9^	−0.277	ACLY, ACTN4, AHCY, AKR1B1, ANXA1, ANXA2, BSG, CACYBP, CALR, CAPN2, CAV1, CD44, CD59, CTSB, CTTN, CYR61, EEF1A1, EZR, FASN, FLNA, FN1, GAPDH, GNB2L1, HDGF, HMGB1, HSPA4, HSPA5, HSPA8, HSPD1, HYOU1, ILF3, ITGB1, LAMB1, LDHA, LGALS1, MAPK1, NPM1, PARK7, PKM, PLEC, PPP2CA, RHOA, RPL22, RPS4X, S100A6, SET, SQSTM1, STAT1, STMN1, TNC, TXLNA, UCHL1, XRCC5, YBX1	54
RNA post-transcriptional modification	Splicing of RNA	5.23 × 10^-9^	0.239	AHNAK, C1QBP, DDX39B, DDX5, FUS, HNRNPA2B1, HNRNPH1, HNRNPH3, HNRNPK, HNRNPM, KHSRP, NPM1, PRPF19, PTBP1, SF3A1, SF3B1, SFPQ, SNRPD1, SYNCRIP, U2AF2	20
Cell death and survival	Cell death of connective tissue cells	5.91 × 10^-9^	−0.258	CAPNS1, CD44, CDK1, CLIC4, CTSB, CYR61, DDX3X, DNM1L, EEF1A1, EIF2S1, EIF3B, EIF3C, EIF3I, EIF6, ENO1, FKBP4, FLNA, FN1, FUS, GLO1, GPI, GSTP1, HINT1, HSPA5, HSPD1, IGF2R, ITGB1, KHDRBS1, KRT18, KRT8, LMNA, MAP4, MAPK1, PARK7, PRKDC, RHOA, RPL10, SERPINH1, STAT1, STMN1, TPP1, UBA1, VCP, VIM, XRCC6, YWHAZ	46
Cancer, hematological disease	Blood tumor	6.43 × 10^-9^	1.121	ACTG1, ANXA1, ANXA2, ANXA6, ATIC, B2M, CALR, CAV1, CD44, CDK1, CFL1, CSE1L, CYR61, DNM1L, EEF1A1, FASN, FDPS, FKBP1A, FLNA, FN1, FUS, G3BP1, GMPS, GNB2L1, GSTP1, HIST1H1C, HLA-A, HMGB1, HSPD1, IMPDH2, IQGAP1, KPNA2, KRT1, LDHA, LGALS1, LOC102724594/U2AF1, LONP1, MARS, MYH10, NNMT, NONO, NPM1, PCNA, PDIA6, PICALM, PLOD2, PPP2CA, PRDX1, PRKDC, PSMA2, PSMB1, PSMD2, PSME1, RHOA, RPL3, RPL6, RPS12, RPS2, RPS24, RPS4X, SF3B1, SHMT2, SNRPD3, SPTBN1, STIP1, STMN1, TUBB, TUBB4B, TUBB6, TUBB8, TXLNA, UQCRC1, VIM, XPO1, XRCC5, XRCC6, YWHAE, YWHAZ	78
Cell-to-cell signaling and interaction	Binding of cells	6.68 × 10^-9^	−0.441	ANXA2, ANXA5, BSG, C1QBP, CALR, CAV1, CCT2, CCT3, CCT4, CCT5, CCT6A, CCT7, CCT8, CD44, CKAP4, CYR61, DDX3X, FN1, HLA-A, HMGB1, HSPA5, IGF2R, ITGB1, KRT1, MAP4, MSN, MYH9, NCL, PDIA3, PPP2CA, PRMT5, RHOA, RPSA, STIP1, STMN1, TCP1, TFRC, TLN1, TNC, VCL	40
Cancer, hematological disease	Hematologic cancer	7.44 × 10^-9^	1.121	ACTG1, ANXA1, ANXA2, ANXA6, ATIC, B2M, CALR, CAV1, CD44, CDK1, CFL1, CSE1L, CYR61, DNM1L, EEF1A1, FASN, FDPS, FKBP1A, FLNA, FN1, FUS, G3BP1, GMPS, GNB2L1, GSTP1, HIST1H1C, HLA-A, HMGB1, HSPD1, IMPDH2, IQGAP1, KPNA2, KRT1, LDHA, LGALS1, LOC102724594/U2AF1, LONP1, MARS, MYH10, NNMT, NONO, NPM1, PCNA, PDIA6, PICALM, PPP2CA, PRDX1, PRKDC, PSMA2, PSMB1, PSMD2, PSME1, RHOA, RPL3, RPL6, RPS12, RPS2, RPS24, RPS4X, SF3B1, SHMT2, SNRPD3, SPTBN1, STIP1, STMN1, TUBB, TUBB4B, TUBB6, TUBB8, TXLNA, UQCRC1, VIM, XPO1, XRCC5, XRCC6, YWHAE, YWHAZ	77
Cellular assembly and organization	Development of cytoplasm	8.43 × 10^-9^	−0.761	ACTR3, ANXA1, ARF1, ARPC2, CAPN1, CAPZB, CAV1, CD44, CFL1, CORO1C, CTTN, DNM1L, DPYSL2, ERP29, FKBP4, FLNA, FN1, GDI1, GNAI2, GPI, HSPA8, IPO9, ITGB1, MAP1B, MAPK1, MAPRE1, NPM1, PARK7, PFN1, RHOA, STMN1, STOML2, TNC, TPM2, TUBB, TWF1, TWF2, ZYX	38
Neurological disease	Amyotrophic lateral sclerosis	9.09 × 10^-9^		ANXA1, ANXA5, CAPN2, CCT2, CYR61, DBI, EEF1A1, EIF4A1, EZR, FASN, FUS, HN1, HNRNPA1, MAPK1, NNMT, PFKP, PFN1, RHOA, RTN4, S100A6, SOD1, TFRC, VCP, VIM	24
Cell death and survival	Cell viability of tumor Cell lines	9.74 × 10^-9^	−0.894	APEX1, ATP5H, B2M, CAPN2, CAV1, CD44, COPB2, DHX9, EEF2, EIF3C, EIF4A1, EIF4G1, FLNA, FN1, GLUD1, GNB2L1, GSTP1, HINT1, HMGB1, HSP90AB1, HSP90B1, HSPA5, HSPB1, IGF2R, ITGB1, P4HB, PARK7, PCNA, PPM1G, PPP1CA, PPP2CA, PPP2R1A, PRDX2, PRKDC, PRPF19, PSMA1, PSMA4, PSMC4, RAB11A, RHOA, RPL27, RPSA, S100A6, SF3A1, SFN, SNRPD1, SOD1, SQSTM1, TCP1, TRIM28, VCP, XPO1, YBX1	53
Hereditary disorder, neurological disease, psychological disorders, skeletal and muscular disorders	Huntington’s disease	1.18 × 10^-8^		ACAT1, AHCY, B2M, BASP1, CAPNS1, CD44, CYC1, DDX1, DNAJA1, ENO2, FKBP4, GAPDH, GPI, HADH, HINT1, HNRNPU, HSP90AA1, HSPA5, HSPA8, LAMB1, LDHA, LDHB, MYL12A, NPM1, PCMT1, PCNA, PDE6H, PDLIM1, PFN1, PGK1, PKM, PLOD2, PPIA, PRDX2, PSME1, RAB11A, RANBP1, RPSA, SDHA, TPI1, TPM3, UCHL1, UQCRC1, XRCC6, YWHAZ	45
Cellular development, cellular growth and proliferation	Proliferation of breast cancer cell lines	1.19 × 10^-8^	0.389	ANXA2, ARF1, C1QBP, CAV1, CD44, CD59, CDK1, CSE1L, CYR61, EEF1A1, EEF1B2, EIF3C, FN1, GNB2L1, HNRNPK, HSPA5, ILF3, ITGB1, KPNA2, KRT8, LGALS1, MAPK1, PDIA3, PFKP, PFN1, PPP2R1A, PRDX2, PTMA, SEPT9, SFN, SOD1, TRIM28, UBA1, YBX1, YWHAQ	35
Carbohydrate metabolism	Glycolysis of cells	1.30 × 10^-8^	−1.474	ALDOA, BSG, C1QBP, CAV1, ENO1, ENO2, GAPDH, GPI, LDHA, PFKP, PGAM1, PGK1, PGM1, PKM, TPI1	15
Cancer, respiratory disease	Metastatic non-small cell lung cancer	1.31 × 10^-8^		ATIC, B2M, EEF1A1, EIF4A1, FN1, MYL12A, PPIA, RPL27, RPL7, RPS11, RPS27A	11
Protein synthesis	Polymerization of protein	1.44 × 10^-8^		ACAT1, AHNAK, ALDOC, ANXA2, ANXA5, ANXA6, ARPC2, CAV1, CTNNA1, CUTA, DNM1L, EEF1A1, EHD1, ENAH, HSD17B10, IMPDH2, LONP1, NPM1, PFKP, PFN1, PRKCSH, RHOA, SEPT2, SEPT7, SEPT9, SHMT2, SQSTM1, STOML2, TRIM28, VCP, YWHAB	31
Cell death and survival	Cell death of fibroblast cell lines	1.61 × 10^-8^	−0.361	CAPNS1, CDK1, CLIC4, CTSB, CYR61, DDX3X, DNM1L, EEF1A1, EIF2S1, EIF3B, EIF3C, EIF3I, EIF6, ENO1, FKBP4, FN1, FUS, GLO1, GPI, GSTP1, HINT1, HSPA5, HSPD1, ITGB1, KRT18, KRT8, MAPK1, PARK7, RHOA, RPL10, STAT1, STMN1, UBA1, VCP, VIM, YWHAZ	36
Cellular assembly and organization, cellular function and maintenance	Organization of actin cytoskeleton	2.12 × 10^-8^	−1.937	ACTR2, ALDOA, ARHGDIA, CALR, CAP1, CFL1, CORO1C, CSRP1, DBN1, DPYSL2, ENAH, EZR, FLNA, FLNB, FLNC, FN1, ITGB1, MSN, MYH10, MYH9, PLS3, RAN, RHOA, SEPT2, SPTAN1, TLN1, TMOD3, TWF2	28
Cancer	Malignant neoplasm of mediastinum	2.27 × 10^-8^	1.982	ATIC, B2M, EEF1A1, EIF4A1, FN1, MYL12A, PPIA, PRDX1, PRKDC, RHOA, RPL27, RPL7, RPS11, RPS27A, TUBB4B, XRCC5, XRCC6, YWHAE	18
Metabolic disease	Amyloidosis	2.45 × 10^-8^		ACLY, ATP5A1, B2M, CANX, CAPN1, CAST, CAV1, CDK1, CTSB, DHX9, DNM1L, DPYSL2, EEF1G, EEF2, EIF2S1, EPRS, FDPS, G3BP1, GAPDH, GNB2L1, HNRNPA1, HNRNPA2B1, HNRNPU, HSPA5, HSPD1, LGALS1, PCNA, PICALM, PRDX1, PRKDC, PSMB1, PSMD2, RAN, SET, SOD1, STIP1, TAGLN, TUBB, UBXN4, UCHL1, VIM, VPS35, YWHAZ	43
Nucleic acid metabolism, small molecule biochemistry	Biosynthesis of purine ribonucleotide	2.57 × 10^-8^	−2.378	ALDOA, ATP5A1, ATP5B, CAV1, CDK1, DNM1L, HMGB1, HSPD1, NNMT, PKM, PPA1, SLC25A5, SOD1, VCP, VDAC1	15
Neurological disease	Neurological signs	2.58 × 10^-8^		ACAT1, AHCY, B2M, BASP1, CAPNS1, CAV1, CD44, CYC1, DDX1, DNAJA1, ENO2, FKBP4, GAPDH, GPI, HADH, HINT1, HNRNPA2B1, HNRNPU, HSP90AA1, HSPA5, HSPA8, LAMB1, LDHA, LDHB, MYL12A, NPM1, PCMT1, PCNA, PDE6H, PDLIM1, PFN1, PGK1, PKM, PLOD2, PPIA, PRDX2, PSME1, RAB11A, RANBP1, RPSA, SDHA, SOD1, TPI1, TPM3, UCHL1, UQCRC1, XRCC6, YWHAZ	48
Cancer	Epithelial cancer	3.20 × 10^-8^	−0.050	AARS, ACAT1, ACAT2, ACLY, ACTG1, ACTN1, ACTN4, ACTR2, ACTR3, AHNAK, AKR1B1, ALDOA, ALDOC, ANXA1, ANXA2, ANXA5, AP1B1, APEX1, ARF1, ARHGDIA, ARPC2, ATIC, ATP5A1, ATP5B, B2M, BASP1, BSG, C14orf166, C1QBP, CACYBP, CAND1, CANX, CAPG, CAPN2, CAPRIN1, CAST, CAV1, CBX3, CCT2, CCT4, CCT5, CCT6A, CCT7, CCT8, CD44, CD59, CDK1, CLIC1, CLTC, COPA, COPB1, COPB2, COPE, COPG1, CORO1C, COTL1, CRIP2, CSE1L, CTNNA1, CTPS1, CTSA, CTSB, CTTN, CUTA, CYC1, CYR61, DBI, DBN1, DDX1, DDX39B, DDX3X, DDX5, DHX9, DLAT, DNAJA1, DNAJA2, DNAJC8, DNM1L, DPYSL2, DYNC1H1, DYNC1I2, ECHS1, EEF1A1, EEF1B2, EEF1D, EEF2, EIF2S1, EIF2S2, EIF3I, EIF4A1, EIF4G1, ENAH, ENO1, ENO2, EPRS, ESYT1, ETFA, EZR, FASN, FDPS, FKBP1A, FLNA, FLNB, FLNC, FN1, FUBP3, FUS, G3BP1, G6PD, GANAB, GARS, GCN1L1, GDI2, GLUD1, GML, GMPS, GNAI2, GNB2L1, GPI, GSTP1, H2AFY, H3F3A/H3F3B, HADHA, HARS, HDGF, HDLBP, HEXB, HINT1, HIST1H1C, HIST1H2BL, HIST2H2AC, HLA-A, HLA-B, HMGB1, HN1, HNRNPA1, HNRNPA2B1, HNRNPA3, HNRNPH1, HNRNPH3, HNRNPK, HNRNPL, HNRNPM, HNRNPU, HSP90AA1, HSP90AB1, HSP90B1, HSPA5, HSPA8, HSPA9, HSPD1, HYOU1, IARS, IDI1, IFITM3, IGF2R, ILF2, ILF3, IMMT, IMPDH2, IPO5, IPO7, IPO9, IQGAP1, ITGB1, KHDRBS1, KHSRP, KIF5B, KPNA2, KPNA3, KPNB1, KRT1, KRT18, KRT8, KRT9, LAMB1, LASP1, LDHA, LGALS1, LMNA, LMNB1, LOC102724594/U2AF1, LONP1, LRPPRC, LRRC59, MAP1B, MAP4, MAPK1, MAPRE1, MARS, MATR3, MCM3, MCM4, MCM6, MCM7, MDH1, MDH2, MSN, MVP, MYH10, MYH9, MYL12A, NACA, NAP1L1, NAP1L4, NASP, NCL, NNMT, NONO, NPEPPS	350
				NPLOC4, NPM1, NSFL1C, NUMA1, OLA1, P4HB, PA2G4, PABPC1, PAFAH1B2, PAICS, PCBP1, PCBP2, PCM1, PCMT1, PCNA, PDCD6, PDIA3, PDIA4, PDLIM1, PDXK, PEA15, PFKP, PFN1, PGAM1, PGD, PGK1, PGM1, PHGDH, PICALM, PKM, PLEC, PLOD2, PPIA, PPM1G, PPP1CA, PPP2CA, PPP2R1A, PRDX1, PRDX2, PRKCSH, PRKDC, PRMT5, PRPF19, PSMA1, PSMC1, PSMC4, PSMD2, PTBP1, PTMA, PTRF, PUF60, PYGB, PYGL, QARS, RANBP1, RARS, RHOA, RPL10, RPL12, RPL22, RPL27, RPL4, RPL5, RPL7, RPL7A, RPL8, RPLP0, RPN1, RPN2, RPS11, RPS12, RPS2, RPS24, RPS27A, RPS7, RRBP1, RTN4, S100A6, SDHA, SEPT9, SERBP1, SERPINH1, SF3A1, SF3B1, SFN, SFPQ, SH3BGRL3, SLC25A3, SLC25A5, SLC38A2, SND1, SOD1, SPTAN1, SPTBN1, SSRP1, STAT1, STIP1, STMN1, SYNCRIP, TAGLN, TALDO1, TARS, TCP1, TFRC, TLN1, TMPO, TNC, TNPO1, TPI1, TPM4, TUBB, TUBB4B, TUBB6, TUBB8, TWF2, TXNDC5, UAP1, UBA1, UBE2N, UBXN4, UCHL1, VARS, VCL, VCP, VIM, VPS35, WARS, WDR1, XPO1, XRCC5, XRCC6, YBX1, YBX3, YWHAB, YWHAE, YWHAG, YWHAH, YWHAQ, YWHAZ, ZYX	
Cancer	Metastatic carcinoma	3.23 × 10^-8^		ATIC, B2M, CSE1L, EEF1A1, EIF4A1, FKBP1A, FN1, HSP90AA1, HSP90AB1, HSP90B1, MYL12A, PPIA, RPL27, RPL7, RPS11, RPS27A, TUBB, TUBB4B, YWHAE	19
Neurological disease	Dyskinesia	3.44 × 10^-8^		ACAT1, AHCY, B2M, BASP1, CAPNS1, CAV1, CD44, CYC1, DDX1, DNAJA1, ENO2, FKBP4, GAPDH, GPI, HADH, HINT1, HNRNPU, HSP90AA1, HSPA5, HSPA8, LAMB1, LDHA, LDHB, MYL12A, NPM1, PCMT1, PCNA, PDE6H, PDLIM1, PFN1, PGK1, PKM, PLOD2, PPIA, PRDX2, PSME1, RAB11A, RANBP1, RPSA, SDHA, TPI1, TPM3, UCHL1, UQCRC1, XRCC6, YWHAZ	46
Cellular assembly and organization	Formation of cytoskeleton	3.46 × 10^-8^	−0.867	ACTR3, ARF1, ARPC2, CAPN1, CAPZB, CAV1, CD44, CFL1, CORO1C, CTTN, DPYSL2, ERP29, FKBP4, FLNA, FN1, GDI1, GNAI2, GPI, ITGB1, MAP1B, MAPK1, MAPRE1, NPM1, PFN1, RHOA, STMN1, TNC, TPM2, TUBB, TWF1, TWF2, ZYX	32
Inflammatory disease	Chronic inflammatory disorder	3.62 × 10^-8^		ACLY, ACTA1, ACTN4, ALDOA, ANXA1, ARF1, ATIC, B2M, BSG, CALR, CAPG, CD59, COTL1, CTSB, DDX39B, DDX5, EEF1G, EEF2, ENO1, FDPS, FKBP1A, H3F3A/H3F3B, HLA-A, HMGB1, HNRNPA1, HNRNPA3, HSP90B1, HSPA8, HSPD1, IMPDH2, KHSRP, KRT18, KRT8, LDHB, LGALS1, MAPRE1, MYL12A, NONO, P4HB, PCM1, PDIA3, PDLIM1, PGK1, PKM, PRDX1, PRDX2, PRDX5, PSMB1, PSMD2, PTMA, RHOA, RPS24, RPS3, RPSA, SND1, STAT1, TFRC, TPM2, TRIM28, UCHL1, VARS, VIM	62
Cell morphology, connective tissue development and function	Shape change of fibroblast cell lines	3.74 × 10^-8^	−0.342	CAST, EHD1, FLNA, FLNC, FN1, GNB2L1, IPO9, ITGB1, MARCKS, PTBP1, RHOA, RTN4, VCL, VIM	14
Cell morphology, cellular assembly and organization, cellular function and maintenance	Formation of lamellipodia	3.90 × 10^-8^	0.308	ACTN4, ACTR3, ARPC2, BSG, C1QBP, CAP1, CAPZB, CD44, CFL1, CYR61, DNM1L, EZR, FN1, HSP90AA1, HSPB1, LASP1, RHOA, TWF2, VCL	19
Cell cycle, cell morphology, cellular assembly and organization, cellular movement	Elongation of filaments	4.03 × 10^-8^	−1.000	ACTN4, CAP1, FN1, MAPRE1, PFN1, SEPT7, SEPT9	7
Cell death and survival	Neuronal cell death	4.08 × 10^-8^	0.202	AARS, AP2B1, CAPN1, CAPNS1, CAPRIN1, CAST, CDK1, CTSB, DNM1L, FKBP1A, FN1, FUS, G6PD, GAPDH, GLUD1, GPI, HDGF, HMGB1, HSD17B10, HSP90AB1, HSPA5, HSPB1, HSPD1, HYOU1, ITGB1, LDHA, LGALS1, MAP1B, MAPK1, NPM1, P4HB, PARK7, PDIA3, PEA15, PRDX2, RHOA, RPS3, SDHA, SET, SOD1, STAT1, STIP1, TCP1, UCHL1, VAPA, XRCC5, XRCC6, YWHAB, YWHAZ	49
Cell-to-cell signaling and interaction, cellular assembly and organization, tissue development	Quantity of focal adhesions	4.08 × 10^-8^	1.463	CAPNS1, CAV1, FLNA, FLNB, FN1, GNB2L1, ITGB1, MYH9, RHOA, SPTAN1	10
Carbohydrate metabolism	Glycolysis	4.46 × 10^-8^	−1.946	ALDOA, BSG, C1QBP, CAV1, DNM1L, ENO1, ENO2, GAPDH, GPI, LDHA, PFKP, PGAM1, PGK1, PGM1, PKM, TPI1	16
RNA post-transcriptional modification	Processing of mRNA	4.53 × 10^-8^	−0.128	C1QBP, DDX39B, DDX5, HNRNPA1, HNRNPA2B1, HNRNPH3, HNRNPK, HNRNPM, KHDRBS1, LOC102724594/U2AF1, NPM1, PABPC1, PCBP1, PRPF19, PTBP1, SF3A1, SF3B1, SFPQ, SNRPD1, U2AF2	20
Molecular transport	Transport of molecule	4.60 × 10^-8^	−1.226	ACAT2, ALYREF, ANXA1, ANXA2, ANXA6, AP1B1, ARF1, ATP1A1, ATP5B, B2M, BSG, CALR, CANX, CAV1, CD44, CFL1, CLIC1, CLIC4, CPNE1, CSE1L, CTSA, DDX39B, DDX3X, DNAJA1, DNAJA2, DNM1L, DPYSL2, EHD1, EIF5A, ERP29, FKBP4, FN1, FUS, GDI2, GNAI2, HNRNPA2B1, HSPA8, HSPA9, IGF2R, IPO5, IPO7, IPO9, ITGB1, KHDRBS1, KIF5B, KPNA2, KPNB1, KRT8, LASP1, LGALS1, MAP1B, MYH9, NPM1, P4HB, PDCD6, PDIA3, PDIA4, PEA15, PICALM, PLIN3, PPIA, RAB11A, RAB7A, RAN, RHOA, RRBP1, RTN4, S100A6, SEC13, SEPT2, SLC25A3, SLC25A5, SLC38A2, SNX3, SOD1, SPTBN1, SQSTM1, STAT1, STOML2, TFRC, TNPO1, U2AF2, VAPA, VDAC1, XPO1, YWHAB, YWHAE, YWHAH, YWHAZ	89
Cellular development	Differentiation of cells	4.66 × 10^-8^	−1.742	ACLY, ACTR3, ALYREF, ANXA1, ANXA2, ANXA6, ATP5B, B2M, BASP1, BSG, C1QBP, CACYBP, CALR, CAND1, CAPNS1, CAPZB, CAST, CAV1, CBX3, CD44, CDK1, CFL1, CLIC1, CLIC4, CLTC, CSRP1, CTSB, CYR61, DBN1, DDX5, DHX9, DNM1L, DPYSL2, EIF5A, ENO1, EZR, FASN, FLNB, FLNC, FN1, GAPDH, GLO1, GNB2L1, H2AFY, H3F3A/H3F3B, HMGB1, HNRNPA2B1, HNRNPK, HNRNPL, HNRNPU, HSP90AA1, HSP90AB1, HSP90B1, HSPA5, HSPD1, IARS, IGF2R, ITGB1, KHDRBS1, KRT1, KRT8, LGALS1, LMNA, LMNB1, LONP1, MAP1B, MAPK1, NAP1L1, NNMT, NPEPPS, NPM1, PA2G4, PDIA3, PFN1, PHGDH, PICALM, PKM, PPIA, PPP1CA, PRDX2, PRKDC, PRMT5, PRPF19, RHOA, RNH1, RPL22, RPS11, RPS15, RPS3A, RPS7, RRBP1, RTN4, SFN, SFPQ, SND1, SOD1, SQSTM1, STAT1, STMN1, SYNCRIP, TAGLN, TAGLN2, TFRC, TLN1, TNC, TPM2, TPM4, VIM, XPO1, XRCC5, XRCC6, YBX1, YBX3, YWHAG, YWHAQ	115
Cancer	Neoplasia of epithelial tissue	4.68 × 10^-8^	0.453	AARS, ACAT1, ACAT2, ACLY, ACTG1, ACTN1, ACTN4, ACTR2, ACTR3, AHNAK, AIP, AKR1B1, ALDOA, ALDOC, ANXA1, ANXA2, ANXA5, AP1B1, APEX1, ARF1, ARHGDIA, ARPC2, ATIC, ATP5A1, ATP5B, B2M, BASP1, BSG, C14orf166, C1QBP, CACYBP, CALR, CAND1, CANX, CAPG, CAPN2, CAPRIN1, CAST, CAV1, CBX3, CCT2, CCT4, CCT5, CCT6A, CCT7, CCT8, CD44, CD59, CDK1, CLIC1, CLTC, COPA, COPB1, COPB2, COPE, COPG1, CORO1C, COTL1, CRIP2, CSE1L, CTNNA1, CTPS1, CTSA, CTSB, CTTN, CUTA, CYC1, CYR61	355
				DBI, DBN1, DDX1, DDX39B, DDX3X, DDX5, DHX9, DLAT, DNAJA1, DNAJA2, DNAJC8, DNM1L, DPYSL2, DYNC1H1, DYNC1I2, ECHS1, EEF1A1, EEF1B2, EEF1D, EEF2, EIF2S1, EIF2S2, EIF3I, EIF4A1, EIF4G1, ENAH, ENO1, ENO2, EPRS, ESYT1, ETFA, EZR, FASN, FDPS, FKBP1A, FLNA, FLNB, FLNC, FN1, FUBP3, FUS, G3BP1, G6PD, GANAB, GARS, GCN1L1, GDI2, GLUD1, GML, GMPS, GNAI2, GNB2L1, GPI, GSTP1, H2AFY, H3F3A/H3F3B, HADHA, HARS, HDGF, HDLBP, HEXB, HINT1, HIST1H1C, HIST1H2BL, HIST2H2AC, HLA-A, HLA-B, HMGB1, HN1, HNRNPA1, HNRNPA2B1, HNRNPA3, HNRNPH1, HNRNPH3, HNRNPK, HNRNPL, HNRNPM, HNRNPU, HSP90AA1, HSP90AB1, HSP90B1, HSPA5, HSPA8, HSPA9, HSPD1, HYOU1, IARS, IDI1, IFITM3, IGF2R, ILF2, ILF3, IMMT, IMPDH2, IPO5, IPO7, IPO9, IQGAP1, ITGB1, KHDRBS1, KHSRP, KIF5B, KPNA2, KPNA3, KPNB1, KRT1, KRT18, KRT8, KRT9, LAMB1, LASP1, LDHA, LGALS1, LMNA, LMNB1, LOC102724594/U2AF1, LONP1, LRPPRC, LRRC59, MAP1B, MAP4, MAPK1, MAPRE1, MARCKS, MARS, MATR3, MCM3, MCM4, MCM6, MCM7, MDH1, MDH2, MSN, MVP, MYH10, MYH9, MYL12A, NACA, NAP1L1, NAP1L4, NASP, NCL, NNMT, NONO, NPEPPS, NPLOC4, NPM1, NSFL1C, NUMA1, OLA1, P4HB, PA2G4, PABPC1, PAFAH1B2, PAICS, PARK7, PCBP1, PCBP2, PCM1, PCMT1, PCNA, PDCD6, PDIA3, PDIA4, PDLIM1, PDXK, PEA15, PFKP, PFN1, PGAM1, PGD, PGK1, PGM1, PHGDH, PICALM, PKM, PLEC, PLOD2, PPIA, PPM1G, PPP1CA, PPP2CA, PPP2R1A, PRDX1, PRDX2, PRKCSH, PRKDC, PRMT5, PRPF19, PSMA1, PSMC1, PSMC4, PSMD2, PTBP1, PTMA, PTRF, PUF60, PYGB, PYGL, QARS, RANBP1, RARS, RHOA, RPL10, RPL12, RPL21, RPL22, RPL27, RPL4, RPL5, RPL7, RPL7A, RPL8, RPLP0, RPN1, RPN2, RPS11, RPS12, RPS2, RPS24, RPS27A, RPS7, RRBP1, RTN4, S100A6, SDHA, SEPT9, SERBP1, SERPINH1, SF3A1, SF3B1, SFN, SFPQ, SH3BGRL3, SLC25A3, SLC25A5, SLC38A2, SND1, SOD1, SPTAN1, SPTBN1, SSRP1, STAT1, STIP1, STMN1, SYNCRIP, TAGLN, TALDO1, TARS, TCP1, TFRC, TLN1, TMPO, TNC, TNPO1, TPI1, TPM4, TUBB, TUBB4B, TUBB6, TUBB8, TWF2, TXNDC5, UAP1, UBA1, UBE2N, UBXN4, UCHL1, VARS, VCL, VCP, VIM, VPS35, WARS, WDR1, XPO1, XRCC5, XRCC6, YBX1, YBX3, YWHAB, YWHAE, YWHAG, YWHAH, YWHAQ, YWHAZ, ZYX	
Hereditary disorder, skeletal and muscular disorders	Autosomal recessive myopathy	4.96 × 10^-8^		AHNAK, CALR, CANX, FN1, HINT1, HSP90B1, HSPA5, ITGB1, LMNA, PLEC, PSMA2, PSMA4, SQSTM1, VCP	14
Infectious disease	Infection of tumor cell lines	5.02 × 10^-8^	−1.728	ACTR2, AP1B1, AP2B1, ARF1, ARPC1B, ATP5B, CCT2, CLTC, COPA, COPB1, COPB2, COPG1, DDX3X, DNAJA2, EIF3I, G3BP1, GML, H3F3A/H3F3B, HMGB1, HNRNPK, HNRNPU, HSPA9, IFITM3, IGF2R, MAP4, PDIA3, PDIA6, PICALM, PSME2, RAB1B, RANBP1, SPTAN1, SPTBN1, STAT1, STIP1, TFRC, TWF1, UAP1, UQCRC1, XPO1	40
Cancer	Abdominal neoplasm	5.10 × 10^-8^	−1.025	AARS, ACAT1, ACAT2, ACLY, ACTG1, ACTN1, ACTN4, ACTR2, ACTR3, AHCY, AHNAK, AKR1B1, ALDOA, ALYREF, ANXA1, ANXA2, ANXA5, AP1B1, APEX1, ARF1, ARHGDIA, ARPC2, ATIC, ATP5A1, ATP5B, B2M, BASP1, BSG, C14orf166, CACYBP, CALR, CAND1, CANX, CAP1, CAPG, CAPN2, CAST, CAV1, CBX3, CCT2, CCT4, CCT5, CCT6A, CCT8, CD44, CD59, CDK1, CLIC1, CLTC, COPA, COPB1, COPB2, COPE, COPG1, CORO1C, COTL1, CRIP2, CSE1L, CTNNA1, CTPS1, CTSA, CTSB, CTTN, CUTA, CYC1, CYR61, DBI, DBN1, DDX1, DDX39B, DDX3X, DDX5, DDX6, DHX9, DLAT, DNAJA1, DNAJA2, DNAJC8, DNM1L, DPYSL2	350
				DYNC1H1, DYNC1I2, ECHS1, EEF1A1, EEF1B2, EEF1D, EEF2, EIF2S1, EIF2S2, EIF3I, EIF4A1, EIF4G1, ENAH, ENO1, ENO2, EPRS, ESYT1, FASN, FDPS, FKBP1A, FLNA, FLNB, FLNC, FN1, FUBP3, FUS, G3BP1, G6PD, GANAB, GAPDH, GARS, GCN1L1, GDI2, GLUD1, GML, GMPS, GNAI2, GNB2L1, GPI, GSTP1, H2AFY, H3F3A/H3F3B, HADHA, HARS, HDGF, HDLBP, HEXB, HINT1, HIST1H1C, HIST1H2BL, HIST2H2AC, HLA-A, HLA-B, HMGB1, HN1, HNRNPA1, HNRNPA2B1, HNRNPA3, HNRNPH1, HNRNPH3, HNRNPK, HNRNPL, HNRNPM, HNRNPU, HSD17B10, HSP90AA1, HSP90AB1, HSP90B1, HSPA5, HSPA8, HSPA9, HSPB1, HSPD1, HYOU1, IARS, IDI1, IFITM3, IGF2R, ILF2, ILF3, IMMT, IMPDH2, IPO5, IPO7, IPO9, IQGAP1, ITGB1, KHDRBS1, KHSRP, KIF5B, KPNA2, KPNA3, KPNB1, KRT1, KRT18, KRT8, KRT9, LAMB1, LDHA, LGALS1, LMNA, LMNB1, LOC102724594/U2AF1, LONP1, LRPPRC, LRRC59, MAP1B, MAP4, MAPK1, MAPRE1, MARS, MATR3, MCM3, MCM4, MCM6, MCM7, MDH1, MDH2, MSN, MVP, MYH10, MYH9, NACA, NAP1L1, NAP1L4, NASP, NCL, NNMT, NONO, NPEPPS, NPLOC4, NPM1, NSFL1C, NUMA1, OLA1, P4HB, PA2G4, PABPC1, PAICS, PCBP1, PCBP2, PCM1, PCNA, PDIA3, PDIA4, PDLIM1, PDXK, PEA15, PFKP, PFN1, PGAM1, PGD, PGK1, PGM1, PHGDH, PICALM, PKM, PLEC, PLIN3, PLOD2, PLS3, PPM1G, PPP1CA, PPP2CA, PPP2R1A, PRDX1, PRDX2, PRKCSH, PRKDC, PSMA1, PSMA7, PSMC1, PSMC4, PSMD2, PTBP1, PTMA, PTRF, PUF60, PYGB, PYGL, QARS, RAN, RANBP1, RARS, RBM3, RHOA, RPL12, RPL22, RPL27, RPL4, RPL5, RPL6, RPL7A, RPL8, RPLP0, RPN1, RPN2, RPS12, RPS15, RPS2, RPS24, RPS4X, RPS7, RRBP1, RTN4, S100A6, SDHA, SEPT9, SERBP1, SERPINH1, SET, SF3A1, SF3B1, SFPQ, SH3BGRL3, SHMT2, SLC25A3, SLC25A5, SLC38A2, SND1, SOD1, SPTAN1, SPTBN1, SQSTM1, SSRP1, STAT1, STIP1, STMN1, SYNCRIP, TAGLN, TALDO1, TARS, TCP1, TFRC, TLN1, TMPO, TNC, TNPO1, TPI1, TPM2, TPM3, TPM4, TUBB, TUBB4B, TUBB8, TWF1, TWF2, TXNDC5, UAP1, UBA1, UBE2N, UBXN4, UCHL1, VARS, VCL, VCP, VIM, VPS35, WARS, XPO1, XRCC5, XRCC6, YBX1, YBX3, YWHAE, YWHAG, YWHAH, YWHAQ, YWHAZ, ZYX	
RNA post-transcriptional modification	Splicing of mRNA	5.56 × 10^-8^	0.239	C1QBP, DDX39B, DDX5, HNRNPA2B1, HNRNPH3, HNRNPK, HNRNPM, NPM1, PRPF19, PTBP1, SF3A1, SF3B1, SFPQ, SNRPD1, U2AF2	15
Cell morphology	Shape of cells	7.25 × 10^-8^		ACTN4, CAPN1, FLNA, GPI, IGF2R, ITGB1, MYH10, RHOA, TPM3	9
Cell morphology, cellular assembly and organization, cellular development, cellular function and maintenance	Formation of plasma membrane projections	7.91 × 10^-8^	−0.331	ACTR3, BASP1, CAPNS1, CAPRIN1, CAPZB, CAV1, CD44, CSRP1, CYR61, DBN1, DNM1L, DPYSL2, EHD1, EZR, GDI1, HMGB1, HNRNPK, ITGB1, KIF5B, LAMB1, MAP1B, MSN, MYH10, NNMT, PDIA3, PFN1, PHGDH, PICALM, PPP2CA, PRKCSH, RAB11A, RHOA, RTN4, SEPT2, SOD1, SPTBN1, STIP1, STMN1, TNC, UCHL1, VAPA, VIM, YWHAH	43
Cell cycle, cellular movement	Cytokinesis	8.89 × 10^-8^	−0.943	ACTN4, CAP1, CFL1, FN1, GNAI2, ITGB1, LMNA, MAPRE1, MSN, MYH10, MYH9, NPM1, PFN1, RAB11A, RHOA, SEPT2, SEPT7, SEPT9, SSRP1, YBX1	20
Cellular movement	Cell movement of breast cancer cell lines	1.01 × 10^-7^	0.407	ANXA1, ANXA2, ARF1, ARPC1B, CAPN2, CAV1, CSE1L, CTTN, ENAH, FLNA, FN1, GNB2L1, GPI, ILF3, ITGB1, KHDRBS1, KPNA2, KRT8, LASP1, MAPK1, MYH9, RHOA, SEPT9, SFN, VIM	25
Post-translational modification, protein folding	Refolding of protein	1.08 × 10^-7^		B2M, DNAJA1, DNAJA2, FKBP1A, HSP90AA1, HSPA8, HSPD1	7
Cell death and survival	Cell viability of myeloma cell lines	1.08 × 10^-7^	−0.376	COPB2, EIF3C, EIF4A1, EIF4G1, HSP90B1, PSMA1, PSMA4, PSMC4, RAB11A, RPL27, SF3A1, SOD1, XPO1	13
Cell morphology, cellular assembly and organization, cellular development, cellular function and maintenance, nervous system development and function, tissue development	Neuritogenesis	1.09 × 10^-7^	−0.401	ACTR3, BASP1, CAPNS1, CAPRIN1, CAPZB, CAV1, CD44, CSRP1, CYR61, DBN1, DNM1L, DPYSL2, EHD1, EZR, GDI1, HMGB1, HNRNPK, ITGB1, LAMB1, MAP1B, MSN, MYH10, NNMT, PDIA3, PFN1, PHGDH, PICALM, PPP2CA, PRKCSH, RAB11A, RHOA, RTN4, SEPT2, SOD1, SPTBN1, STIP1, STMN1, TNC, UCHL1, VAPA, VIM, YWHAH	42
Free radical scavenging	Synthesis of reactive oxygen species	1.15 × 10^-7^	−0.019	AKR1B1, ANXA1, ARHGDIA, CAV1, CD44, CLIC1, CTTN, CYR61, DNM1L, FN1, G6PD, GNB2L1, GSTP1, HMGB1, HNRNPK, HSD17B10, HSP90AB1, HSPA9, HSPB1, IMMT, IQGAP1, ITGB1, LDHA, LONP1, MAPK1, PARK7, PPIA, PRDX1, PRDX2, RHOA, S100A6, SOD1, SQSTM1, TAGLN, TFRC, VDAC1, YWHAZ	37
Free radical scavenging	Metabolism of reactive oxygen species	1.17 × 10^-7^	−0.644	AKR1B1, ANXA1, ARHGDIA, CAV1, CD44, CLIC1, CTTN, CYR61, DNM1L, FN1, G6PD, GNB2L1, GSTP1, HMGB1, HNRNPK, HSD17B10, HSP90AB1, HSPA9, HSPB1, IMMT, IQGAP1, ITGB1, LDHA, LONP1, MAPK1, PARK7, PPIA, PRDX1, PRDX2, PRDX5, RHOA, S100A6, SOD1, SQSTM1, TAGLN, TFRC, VDAC1, YWHAZ	38
Cancer	Breast or colorectal cancer	1.22 × 10^-7^	0.555	AARS, ACAT1, ACAT2, ACLY, ACTN1, AHCY, AHNAK, AKR1B1, ALDOA, ALDOC, ANXA1, ANXA2, ARHGDIA, ATIC, ATP5A1, B2M, BSG, C1QBP, CACYBP, CAPG, CAPN2, CAV1, CBX3, CCT3, CCT4, CCT5, CD44, CDK1, COPA, COPB2, CSE1L, CTSB, CYC1, CYR61, DBI, DBN1, DDX1, DDX3X, DDX5, DLAT, DNAJA1, DNAJC8, DPYSL2, DYNC1H1, DYNC1I2, ECHS1, EEF1A1, EEF1D, EEF2, EIF3B, EIF3C, EIF4A1, EIF4G1, ENAH, ENO1, ESYT1, ETFA, FASN, FDPS, FKBP1A, FLNA, FLNB, FLNC, FN1, FUBP3, FUS, G3BP1, G6PD, GARS, GCN1L1, GLO1, GML, GMPS, GNAI2, GSTP1, H2AFY, H3F3A/H3F3B, HDGF, HIST1H1C, HLA-A, HLA-B, HNRNPA1, HNRNPA3, HNRNPH3, HNRNPL, HNRNPM, HNRNPU, HSP90AA1, HSP90AB1, HSP90B1, HSPA8, HSPA9, HSPD1, IDI1, IFITM3, IGF2R, ILF2, ILF3, IMMT, IPO7, IQGAP1, ITGB1, KHSRP, KIF5B, KPNA2, KPNA3, KRT1, KRT18, KRT8, LAMB1, LASP1, LDHA, LGALS1, LMNA, LMNB1, LONP1, LRPPRC, MAP1B, MAPRE1, MARS, MATR3, MCM3, MCM4, MCM6, MDH1, MDH2, MVP, MYH10, MYH9, NACA, NAP1L1, NAP1L4, NCL, NNMT, NPEPPS, OLA1, P4HB, PAFAH1B2, PAICS, PCBP1, PCNA, PDCD6, PFKP, PFN1, PGAM1, PGD, PGK1, PGM1, PKM, PLEC, PLIN3, PLOD2, PLS3, PPP1CA, PPP2CA, PPP2R1A, PRDX1, PRDX5, PRKCSH, PRKDC, PSMA7, PTBP1, PTMA, PTRF, PYGB, RANBP1, RARS, RHOA, RPL4, RPL5, RPL6, RPLP0, RPN1, RPN2, RPS24, RPS3, RTN4, S100A6, SEPT9, SERBP1, SERPINH1, SET, SF3B1, SFPQ, SHMT2, SLC25A5, SLC25A6, SOD1, SPTAN1, SSRP1, STAT1, STIP1, SYNCRIP, TAGLN, TAGLN2, TARS, TCP1, TFRC, TLN1, TMPO, TNC, TNPO1, TPI1, TPM4, TUBA1B, TUBB, TUBB4B, TUBB6, TUBB8, UBA1, VCL, VDAC2, VIM, VPS35, WDR1, XPO1, XRCC6, YBX1, YWHAE, YWHAH, YWHAQ, YWHAZ, ZYX	223
Cellular function and maintenance	Endocytosis	1.22 × 10^-7^	−0.427	ACTN4, ANXA5, ANXA6, ATP5B, CANX, CAP1, CAV1, CD44, CLTC, CORO1C, CTTN, DPYSL2, EHD1, EZR, GNB2L1, HNRNPK, HSPA8, HSPA9, HYOU1, IGF2R, IQGAP1, ITGB1, MAP1B, NCL, PICALM, RAB7A, RHOA, SFPQ, TLN1	29
Neurological disease, psychological disorders	Dementia	1.30 × 10^-7^		ACLY, ATP5A1, CANX, CAPN1, CAV1, CDK1, CTSB, DHX9, DNM1L, DPYSL2, EEF1G, EEF2, EIF2S1, EPRS, FDPS, G3BP1, GAPDH, GNB2L1, HNRNPA1, HNRNPA2B1, HNRNPU, HSPA5, HSPD1, LGALS1, PARK7, PCNA, PICALM, PRDX1, PRKDC, RAN, SET, SOD1, STIP1, TAGLN, TUBB, UBXN4, UCHL1, VCP, VIM, VPS35, YWHAZ	41
Cancer, respiratory disease	Non-squamous non-small cell lung cancer	1.39 × 10^-7^		AHNAK, ALDOA, ALDOC, ATIC, CAPRIN1, CAV1, CD44, CDK1, DHX9, EEF1B2, EIF4A1, ENO1, EZR, G3BP1, GPI, HIST2H2AC, HLA-A, HSP90AA1, HSP90AB1, HSP90B1, IMPDH2, LDHA, LOC102724594/U2AF1, MAP4, MSN, MYH9, NACA, NSFL1C, PCNA, PFKP, PKM, PRDX1, PRPF19, SERBP1, TPI1, TUBB4B	36
Skeletal and muscular disorders	Caveolinopathy	1.42 × 10^-7^		ACTA1, AHNAK, CALR, CANX, FLNC, HSP90B1, HSPA5, LMNA, MATR3, PSMA2, PSMA4, SQSTM1, VCL, VCP	14
Cellular movement	Invasion of tumor cell lines	1.49 × 10^-7^	−0.733	ACAT1, ANXA1, APEX1, ARHGDIA, BSG, CALR, CAP1, CAPNS1, CAV1, CD44, CSE1L, CTSB, CTTN, ENAH, EZR, FN1, GNB2L1, GPI, HDGF, HMGB1, HSP90AA1, ILF3, IQGAP1, ITGB1, KRT8, LGALS1, MAPK1, MARCKS, PA2G4, PEBP1, PKM, PTGES3, RHOA, RPSA, S100A6, SEPT9, SQSTM1, STMN1, TAGLN, TAGLN2, VCP, VIM, YWHAQ	43
Cellular movement	Cell movement of connective tissue cells	1.54 × 10^-7^	−0.391	CAPN1, CAPN2, CAPNS1, CAV1, CD44, CYR61, EHD1, ENAH, EZR, FLNB, FN1, GNB2L1, HMGB1, IGF2R, ITGB1, LGALS1, MAPK1, MYH10, PLEC, STMN1, VCL, ZYX	22
Cancer	Follicular adenoma	1.67 × 10^-7^		ANXA5, CALR, CTSB, GSTP1, HSP90AB1, PARK7, PRDX2	7
Neurological disease, psychological disorders	Tauopathy	1.70 × 10^-7^		ACLY, ATP5A1, CANX, CAPN1, CAV1, CDK1, CTSB, DHX9, DNM1L, DPYSL2, EEF1G, EEF2, EIF2S1, EPRS, FDPS, G3BP1, GAPDH, GNB2L1, HNRNPA1, HNRNPA2B1, HNRNPU, HSPA5, HSPD1, LGALS1, PARK7, PCNA, PICALM, PRDX1, PRKDC, RAN, SET, SOD1, STIP1, TAGLN, TUBB, TUBB4B, UBXN4, UCHL1, VIM, VPS35, YWHAZ	41
Cancer	Metastatic malignant solid tumor	1.70 × 10^-7^		ATIC, B2M, CAV1, CSE1L, EEF1A1, EIF4A1, FKBP1A, FN1, HSP90AA1, HSP90AB1, HSP90B1, MYL12A, PPIA, RPL27, RPL7, RPS11, RPS27A, TUBB, TUBB4B, YWHAE, YWHAZ	21
Cell death and survival	Cell death of central nervous system cells	1.85 × 10^-7^	0.374	CAPN1, CAPNS1, CAST, CDK1, DNM1L, FUS, GAPDH, HMGB1, HSP90AA1, HSPA5, HSPB1, HSPD1, HYOU1, MAP1B, MAPK1, NPM1, P4HB, PARK7, PEA15, RHOA, RPS3, SOD1, STIP1, TCP1, VAPA, YWHAB	26
Molecular transport, protein trafficking	Import of protein	2.01 × 10^-7^	0.816	CFL1, DNAJA1, DNAJA2, IPO5, IPO7, IPO9, KPNA2, KPNB1, PDIA3, RAN, SPTBN1, XPO1	12
Nucleic acid metabolism, small molecule biochemistry	Synthesis of purine nucleotide	2.04 × 10^-7^	−2.561	ALDOA, ATP5A1, ATP5B, CAV1, CDK1, DNM1L, FASN, G6PD, GMPS, HMGB1, HSPD1, IMPDH2, NNMT, PKM, PPA1, SLC25A5, SOD1, VCP, VDAC1	19
Cancer, organismal injury and abnormalities	Lymphatic neoplasia	2.22 × 10^-7^	0.882	ACTG1, ANXA1, ATIC, B2M, CALR, CD44, CFL1, CSE1L, CYR61, DNM1L, EEF1A1, FKBP1A, FN1, FUS, GMPS, GNB2L1, GSTP1, HIST1H1C, HMGB1, HSP90AA1, HSP90AB1, HSP90B1, HSPD1, IMPDH2, IQGAP1, ITGB1, KHDRBS1, KRT1, LDHA, LGALS1, LOC102724594/U2AF1, LONP1, MARS, MYH10, NNMT, NPM1, PICALM, PLOD2, PRDX1, PRKDC, PSMB1, PSMD2, PSME1, RHOA, RPL10, RPL3, RPL6, RPS12, RPS2, RPS24, RPS4X, SF3B1, SHMT2, SNRPD3, SPTBN1, STMN1, TUBB, TUBB4B, TUBB6, TUBB8, TXLNA, UQCRC1, XPO1, XRCC5, XRCC6, YWHAZ	66
Energy production, nucleic acid metabolism, small molecule biochemistry	Synthesis of ATP	2.47 × 10^-7^	−2.160	ALDOA, ATP5B, CAV1, CDK1, DNM1L, HMGB1, HSPD1, NNMT, PKM, SLC25A5, SOD1, VCP, VDAC1	13
Cell morphology, cellular assembly and organization, cellular development, cellular function and maintenance, nervous system development and function, tissue development	Morphogenesis of neurites	2.71 × 10^-7^	−0.815	ACTR3, CAPNS1, CAPRIN1, CAPZB, CAV1, CD44, CSRP1, CYR61, DBN1, DNM1L, DPYSL2, EHD1, EZR, HMGB1, HNRNPK, ITGB1, LAMB1, MAP1B, MYH10, NNMT, PDIA3, PFN1, PHGDH, PICALM, RAB11A, RHOA, RTN4, SEPT2, SOD1, TNC, VAPA, YWHAH	32
Cell death and survival	Apoptosis of neurons	2.84 × 10^-7^	1.726	AARS, CAPN1, CAPRIN1, CAST, CDK1, CTSB, DNM1L, FN1, G6PD, GAPDH, GLUD1, GPI, HSD17B10, HSP90AB1, HSPA5, HSPB1, HSPD1, HYOU1, LGALS1, MAP1B, MAPK1, NPM1, P4HB, PARK7, PEA15, PRDX2, RHOA, RPS3, SET, SOD1, STAT1, XRCC5, XRCC6, YWHAB	34
Nucleic acid metabolism, small molecule biochemistry	Biosynthesis of nucleoside triphosphate	2.93 × 10^-7^	−2.160	ALDOA, ATP5B, CAV1, CDK1, CTPS1, DNM1L, HMGB1, HSPD1, NNMT, PKM, SLC25A5, SOD1, VCP, VDAC1	14
Cellular development, skeletal and muscular system development and function, tissue development	Differentiation of osteoblasts	3.01 × 10^-7^	−1.093	ALYREF, ATP5B, CAPNS1, CLIC1, CLTC, CYR61, DDX5, DHX9, FASN, FN1, GNB2L1, H3F3A/H3F3B, HNRNPU, IARS, MAPK1, RPS11, RPS15, RRBP1, SND1, STAT1, STMN1, SYNCRIP, TNC, TPM4, VIM	25
Cell-to-cell signaling and interaction, cellular movement, hematological system development and function, immune cell trafficking, tissue development	Detachment of granulocytes	3.22 × 10^-7^	0.254	ANXA1, ANXA5, ITGB1, RHOA	4
Cellular movement, connective tissue development and function	Cell movement of fibroblasts	3.30 × 10^-7^	−0.142	CAPN2, CAPNS1, CAV1, CD44, CYR61, EHD1, ENAH, EZR, FLNB, FN1, HMGB1, IGF2R, ITGB1, MAPK1, MYH10, PLEC, STMN1, VCL, ZYX	19
Cancer, organismal injury and abnormalities	Lymphoid cancer	3.34 × 10^-7^	0.818	ACTG1, ANXA1, ATIC, B2M, CALR, CD44, CFL1, CSE1L, CYR61, DNM1L, EEF1A1, FKBP1A, FN1, FUS, GMPS, GNB2L1, GSTP1, HIST1H1C, HMGB1, HSP90AA1, HSP90AB1, HSP90B1, HSPD1, IMPDH2, IQGAP1, ITGB1, KHDRBS1, KRT1, LDHA, LGALS1, LOC102724594/U2AF1, LONP1, MARS, MYH10, NNMT, NPM1, PICALM, PRDX1, PRKDC, PSMB1, PSMD2, PSME1, RHOA, RPL3, RPL6, RPS12, RPS2, RPS24, RPS4X, SF3B1, SHMT2, SNRPD3, SPTBN1, STMN1, TUBB, TUBB4B, TUBB6, TUBB8, TXLNA, UQCRC1, XPO1, XRCC5, XRCC6, YWHAZ	64
Metabolic disease, neurological disease, psychological disorders	Alzheimer’s disease	3.37 × 10^-7^		ACLY, ATP5A1, CANX, CAPN1, CAV1, CDK1, CTSB, DHX9, DNM1L, DPYSL2, EEF1G, EEF2, EIF2S1, EPRS, FDPS, G3BP1, GAPDH, GNB2L1, HNRNPA1, HNRNPA2B1, HNRNPU, HSPA5, HSPD1, LGALS1, PCNA, PICALM, PRDX1, PRKDC, RAN, SET, SOD1, STIP1, TAGLN, TUBB, UBXN4, UCHL1, VIM, VPS35, YWHAZ	39
Cancer, tumor morphology	Invasion of tumor	3.40 × 10^-7^	−1.402	AHCY, ANXA2, BSG, CAPN2, CAV1, CD44, CTSB, CTTN, EZR, FASN, FKBP1A, FLNA, FN1, HDLBP, HMGB1, HNRNPA1, HSPA5, ITGB1, LGALS1, MAPK1, PARK7, RHOA, VIM	23
Hereditary disorder, neurological disease	Autosomal dominant neuropathy	4.17 × 10^-7^		AARS, DYNC1H1, FUS, GARS, HSPB1, HSPD1, RAB7A, SOD1, UCHL1	9
Gene expression	Binding of DNA	4.32 × 10^-7^	−1.045	ALYREF, APEX1, CALR, CAV1, CDK1, CFL1, FN1, GAPDH, GNB2L1, GSTP1, H3F3A/H3F3B, HMGB1, LGALS1, LMNA, MAPK1, NCL, NPM1, PCNA, PPIA, PRDX1, PRDX4, PRKDC, RHOA, SET, SFPQ, SND1, SOD1, STAT1, TAGLN, TRIM28, UBE2N, XRCC5, YBX1, YBX3, YWHAB, YWHAE, YWHAG, YWHAZ	38
Cell-to-cell signaling and interaction, tissue development	Adhesion of connective tissue cells	5.10 × 10^-7^	−1.487	ANXA2, CALR, CD44, CYR61, FN1, IGF2R, IPO9, IQGAP1, ITGB1, MAPK1, PLEC, RHOA, RPL22, TLN1, TNC, VCL, ZYX	17
Cellular movement	Cell movement of fibrosarcoma cell lines	5.20 × 10^-7^	−1.621	ARPC2, CTTN, FLNA, FLNB, FLNC, FN1, GPI, HNRNPK, TPM3	9
Lipid metabolism, small molecule biochemistry	Binding of lipid	5.48 × 10^-7^	0.823	ANXA2, DNAJA1, FKBP4, FN1, HMGB1, HSP90AB1, MAP4, PTGES3, RPSA, SFN, STIP1, STMN1	12
DNA replication, recombination, and repair	Double-stranded DNA break repair	5.75 × 10^-7^	0.116	CDK1, DDX1, FUS, KPNA2, NPM1, OTUB1, PCNA, PRKDC, PRPF19, TRIM28, UBE2N, VCP, XRCC5, XRCC6	14
Cell signaling, DNA replication, recombination, and repair, nucleic acid metabolism, small molecule biochemistry	Hydrolysis of GTP	5.79 × 10^-7^	−1.580	CALR, CDK1, DNM1L, GNAI2, IPO5, RAB7A, RAN, RANBP1, RHOA, STMN1, XPO1	11
Cell death and survival	Cell death of breast cancer Cell lines	6.10 × 10^-7^	−0.856	ARHGDIA, B2M, CAV1, CD44, CDK1, CSE1L, CYR61, FASN, FN1, HINT1, HSPA5, HSPB1, HSPD1, KPNA2, KRT18, MAPK1, PEA15, PEBP1, PRKDC, RHOA, SLC25A6, SND1, SSRP1, STAT1, STMN1, TFRC, VCP	27
Connective tissue disorders, skeletal and muscular disorders	Arthropathy	7.25 × 10^-7^	−0.277	ACLY, ACTA1, ALDOA, ANXA1, ARF1, ATIC, B2M, CALR, CAPN2, CD44, CD59, CTSB, DDX39B, EEF1G, EEF2, ENO1, FASN, FDPS, FKBP1A, FN1, GNAI2, GPI, H3F3A/H3F3B, HLA-A, HLA-B, HMGB1, HNRNPA1, HNRNPA3, HSP90B1, HSPA5, HSPA8, HSPD1, KHSRP, LDHB, LGALS1, MAPRE1, MYL12A, NONO, PCM1, PDIA3, PGK1, PHB2, PRDX1, PRDX2, PRDX5, PTMA, RPS24, RPS3, RPSA, SND1, STAT1, TFRC, TPM2, TRIM28, TUBB, TUBB4B, UBA1, VARS, VIM	59
Cancer, gastrointestinal disease, hepatic system disease	Cholangiocarcinoma	7.33 × 10^-7^		ANXA1, ANXA2, GNB2L1, HSP90AA1, HSP90AB1, PGK1, PKM, RPL4, RPLP0, VIM	10
Endocrine system disorders, gastrointestinal disease, immunological disease, inflammatory disease	Autoimmune pancreatitis	7.47 × 10^-7^		CALR, CAPG, COTL1, P4HB, PKM, PRDX2, UCHL1	7
Cellular assembly and organization, cellular compromise	Formation of cytoplasmic inclusions	7.50 × 10^-7^		FUS, KRT18, KRT8, SOD1, SQSTM1, UCHL1	6
Cell death and survival	Cell death of colon cancer Cell lines	7.75 × 10^-7^	1.899	AHSA1, CAPN2, CD44, CDK1, CSE1L, FASN, GSTP1, HINT1, HSPD1, IGF2R, ITGB1, KRT18, LMNA, PARK7, PPIA, RHOA, SFN, SFPQ, SQSTM1, STAT1, VCP, XRCC5, YWHAE, YWHAH	24
Cancer	Abdominal cancer	8.21 × 10^-7^	−0.593	AARS, ACAT1, ACAT2, ACLY, ACTG1, ACTN1, ACTN4, ACTR2, ACTR3, AHCY, AHNAK, AKR1B1, ANXA1, ANXA2, ANXA5, AP1B1, APEX1, ARF1, ARHGDIA, ARPC2, ATIC, ATP5A1, ATP5B, B2M, BASP1, BSG, C14orf166, CACYBP, CALR, CAND1, CANX, CAP1, CAPG, CAPN2, CAST, CAV1, CBX3, CCT2, CCT4, CCT5, CCT6A, CCT8, CD44, CD59, CDK1, CLIC1, CLTC, COPA, COPB1, COPB2, COPE, COPG1, CORO1C, COTL1, CRIP2, CSE1L, CTNNA1, CTPS1, CTSA, CTSB, CTTN, CUTA, CYC1, CYR61, DBI, DBN1, DDX1, DDX39B, DDX3X, DDX5, DHX9, DLAT, DNAJA1, DNAJA2, DNAJC8, DNM1L, DPYSL2, DYNC1H1, DYNC1I2, ECHS1, EEF1A1, EEF1B2, EEF1D, EEF2, EIF2S1, EIF2S2, EIF3I, EIF4A1, EIF4G1, ENAH, ENO1, ENO2, EPRS, ESYT1, FASN, FDPS, FKBP1A, FLNA, FLNB, FLNC, FN1, FUBP3, FUS, G3BP1, G6PD, GANAB, GAPDH, GARS, GCN1L1, GDI2, GLUD1, GML, GMPS, GNAI2, GNB2L1, GPI, GSTP1, H2AFY, H3F3A/H3F3B, HADHA, HARS, HDGF, HDLBP, HEXB, HINT1, HIST1H1C, HIST1H2BL, HIST2H2AC, HLA-A, HLA-B, HMGB1, HN1, HNRNPA1, HNRNPA2B1, HNRNPA3, HNRNPH1, HNRNPH3, HNRNPK, HNRNPL, HNRNPM, HNRNPU, HSP90AA1, HSP90AB1, HSP90B1, HSPA5, HSPA8, HSPA9, HSPB1, HSPD1, HYOU1, IARS, IDI1, IFITM3, IGF2R, ILF2, ILF3, IMMT, IMPDH2, IPO5, IPO7, IPO9, IQGAP1, ITGB1, KHDRBS1, KHSRP, KIF5B, KPNA2, KPNA3, KPNB1, KRT1, KRT18, KRT8, KRT9, LAMB1, LDHA, LGALS1, LMNA, LMNB1, LOC102724594/U2AF1, LONP1, LRPPRC, LRRC59, MAP1B, MAP4, MAPK1, MAPRE1, MARS, MATR3, MCM3, MCM4, MCM6, MCM7, MDH1, MDH2, MSN, MVP, MYH10, MYH9, NACA, NAP1L1, NAP1L4, NASP, NCL, NNMT, NONO, NPEPPS, NPLOC4, NPM1, NSFL1C, NUMA1, OLA1, P4HB, PA2G4, PABPC1, PAICS, PCBP1, PCBP2, PCM1, PCNA, PDIA3, PDIA4, PDLIM1, PDXK, PEA15, PFKP, PFN1, PGAM1, PGD, PGK1, PGM1, PHGDH, PICALM, PKM, PLEC, PLIN3, PLOD2, PLS3, PPM1G, PPP1CA, PPP2CA, PPP2R1A, PRDX1, PRDX2, PRKCSH, PRKDC, PSMA1, PSMA7, PSMC1, PSMC4, PSMD2, PTBP1, PTMA, PTRF, PUF60, PYGB, PYGL, QARS, RAN, RANBP1, RARS, RBM3, RHOA, RPL12, RPL22, RPL4, RPL5, RPL6, RPL7A, RPL8, RPLP0, RPN1, RPN2, RPS12, RPS2, RPS24, RPS4X, RPS7, RRBP1, RTN4, S100A6, SDHA, SEPT9, SERBP1, SERPINH1, SET, SF3A1, SF3B1, SFPQ, SH3BGRL3, SHMT2, SLC25A3, SLC25A5, SLC38A2, SND1, SOD1, SPTAN1, SPTBN1, SQSTM1, SSRP1, STAT1, STIP1, SYNCRIP, TAGLN, TALDO1, TARS, TCP1, TFRC, TLN1, TMPO, TNC, TNPO1, TPI1, TPM2, TPM3, TPM4, TUBB, TUBB4B, TUBB8, TWF2, TXNDC5, UAP1, UBA1, UBE2N, UBXN4, UCHL1, VARS, VCL, VCP, VIM, VPS35, WARS, XPO1, XRCC5, XRCC6, YBX1, YBX3, YWHAE, YWHAG, YWHAH, YWHAQ, YWHAZ, ZYX	342
Drug metabolism, endocrine system development and function, lipid metabolism, small molecule biochemistry	Binding of progesterone	8.87 × 10^-7^	−0.277	DNAJA1, FKBP4, HSP90AB1, PTGES3, STIP1	5
Connective tissue disorders, inflammatory disease, skeletal and muscular disorders	Arthritis	8.98 × 10^-7^	−0.277	ACLY, ACTA1, ALDOA, ANXA1, ARF1, ATIC, B2M, CALR, CAPN2, CD44, CD59, CTSB, DDX39B, EEF1G, EEF2, ENO1, FASN, FDPS, FKBP1A, FN1, GNAI2, GPI, H3F3A/H3F3B, HLA-A, HLA-B, HMGB1, HNRNPA1, HNRNPA3, HSP90B1, HSPA5, HSPA8, HSPD1, KHSRP, LDHB, LGALS1, MAPRE1, MYL12A, NONO, PCM1, PDIA3, PGK1, PHB2, PRDX1, PRDX2, PRDX5, PTMA, RPS24, RPS3, RPSA, SND1, STAT1, TFRC, TPM2, TRIM28, TUBB, TUBB4B, VARS, VIM	58
Cellular compromise, cellular function and maintenance	Endoplasmic reticulum stress response	9.28 × 10^-7^	1.069	AARS, CALR, CTSB, DNAJA1, HSP90AA1, HSP90AB1, HSP90B1, HSPA5, HSPD1, HYOU1, SERPINH1, SLC38A2, SQSTM1, UCHL1, VCP	15
Cell death and survival	Cell death of cerebral cortex cells	9.41 × 10^-7^	1.181	CAPN1, CAPNS1, CAST, CDK1, FUS, GAPDH, HMGB1, HSPA5, HSPB1, HSPD1, HYOU1, MAP1B, P4HB, PARK7, PEA15, RPS3, SOD1, STIP1, TCP1, VAPA, YWHAB	21
Connective tissue disorders, immunological disease, inflammatory disease, skeletal and muscular disorders	Rheumatoid arthritis	9.82 × 10^-7^		ACLY, ACTA1, ALDOA, ANXA1, ARF1, ATIC, B2M, CALR, CTSB, DDX39B, EEF1G, EEF2, ENO1, FDPS, FKBP1A, H3F3A/H3F3B, HLA-A, HMGB1, HNRNPA1, HNRNPA3, HSP90B1, HSPA8, HSPD1, KHSRP, LDHB, LGALS1, MAPRE1, MYL12A, NONO, PCM1, PDIA3, PGK1, PRDX1, PRDX2, PRDX5, PTMA, RPS24, RPS3, RPSA, SND1, STAT1, TFRC, TPM2, TRIM28, VARS, VIM	46
Cellular assembly and organization, cellular function and maintenance	Bundling of microtubules	1.03 × 10^-6^	0.218	DNM1L, MAP1B, MAPRE1, NUMA1, RRBP1, SEPT9, SSRP1	7
Cellular movement	Migration of breast cancer Cell lines	1.21 × 10^-6^	0.280	ANXA1, ANXA2, ARF1, CAPN2, CAV1, CSE1L, CTTN, FLNA, FN1, GNB2L1, ILF3, ITGB1, KHDRBS1, KPNA2, KRT8, LASP1, MAPK1, MYH9, RHOA, SFN, VIM	21
Cellular compromise	Collapse of intermediate filaments	1.58 × 10^-6^		KRT18, KRT8, PLEC, RHOA	4
Connective tissue disorders, inflammatory disease, skeletal and muscular disorders	Rheumatic disease	1.66 × 10^-6^	−0.277	ACLY, ACTA1, ALDOA, ANXA1, ARF1, ATIC, B2M, CALR, CAPN2, CD44, CD59, CTSA, CTSB, DDX39B, EEF1G, EEF2, ENO1, FASN, FDPS, FKBP1A, FN1, GNAI2, GPI, H3F3A/H3F3B, HLA-A, HLA-B, HMGB1, HNRNPA1, HNRNPA3, HSP90B1, HSPA5, HSPA8, HSPD1, KHSRP, LDHB, LGALS1, MAPRE1, MYL12A, NONO, PCM1, PDIA3, PGK1, PHB2, PPP2CA, PRDX1, PRDX2, PRDX5, PTMA, RHOA, RPS24, RPS3, RPSA, SND1, STAT1, TFRC, TPM2, TRIM28, TUBB, TUBB4B, UBE2L3, VARS, VIM	62
Organismal injury and abnormalities, reproductive system disease	Adenomyosis	1.67 × 10^-6^		ALDOA, ANXA2, DDX6, GPI, IQGAP1, ITGB1, LDHA, MYH10, PRDX5, VDAC1	10
DNA replication, recombination, and repair, energy production, nucleic acid metabolism, small molecule biochemistry	Hydrolysis of ATP	1.72 × 10^-6^	−0.164	ATP1A1, CCT4, CCT5, CDK1, HMGB1, HSPA5, HSPD1, MAPK1, UBA1	9
Cell-to-cell signaling and interaction, tissue development	Detachment of blood cells	1.74 × 10^-6^	0.293	ANXA1, ANXA5, FN1, ITGB1, RHOA	5
Cell death and survival	Cell death of muscle cells	1.79 × 10^-6^	1.087	ALDOA, CALR, CAPN1, CAV1, CYR61, EEF1A1, EEF1D, GAPDH, GNAI2, HADHA, HLA-B, HMGB1, HSPB1, HSPD1, LMNA, MAPK1, MDH1, NCL, PARK7, PLEC, PSMB1, RBM3, RHOA, RPSA, S100A6, STAT1, ZYX	27
Cell-to-cell signaling and interaction, tissue development	Adhesion of tumor cell lines	1.86 × 10^-6^	−0.235	ANXA1, ANXA2, BSG, C1QBP, CAV1, CD44, CD59, CYR61, ERP29, FLNA, FN1, GNB2L1, HMGB1, ITGB1, MAPK1, MARCKS, MYH9, PTGES3, RHOA, RPSA, UCHL1, VCL, ZYX	23
Cardiovascular system development and function, cellular movement	Migration of endothelial cells	1.90 × 10^-6^	−2.222	ANXA2, CAV1, CYR61, FLNA, FLNB, FN1, G6PD, HMGB1, HSP90AB1, HSPA5, HSPB1, IGF2R, ITGB1, LGALS1, MAPK1, MARCKS, NCL, PDCD6, PKM, RHOA, RTN4, SEPT7, TARS, VIM, WARS, YWHAZ	26
Cancer, cellular development, cellular growth and proliferation, tumor morphology	Proliferation of tumor cells	2.04 × 10^-6^	−1.588	ACLY, ACTN4, AHCY, AKR1B1, ANXA1, ANXA2, BSG, CACYBP, CAV1, CD44, CTSB, CYR61, EEF1A1, EZR, FASN, FN1, HDGF, HMGB1, HSPA5, LDHA, LGALS1, MAPK1, NPM1, PARK7, PPP2CA, RHOA, RPS4X, S100A6, SQSTM1, STAT1, STMN1, TXLNA, UCHL1, XRCC5	34
Cellular assembly and organization	Binding of zona pellucida	2.10 × 10^-6^		CCT2, CCT3, CCT4, CCT5, CCT6A, CCT7, CCT8, TCP1	8
Cellular function and maintenance	Engulfment of cells	2.23 × 10^-6^	−0.600	ACTN4, ANXA1, ANXA5, CALR, CAPG, CAV1, CD44, CLIC4, CLTC, CORO1C, EHD1, EZR, FN1, GNB2L1, HMGB1, HYOU1, IQGAP1, ITGB1, MAP1B, MAPK1, MYH9, NCL, NPM1, PFN1, PICALM, RAB7A, RHOA, SFPQ, SNX3, VIM	30
Cancer, respiratory disease	Non small cell lung adenocarcinoma	2.24 × 10^-6^		AHNAK, ALDOA, ALDOC, ATIC, CAPRIN1, CAV1, CD44, CDK1, DHX9, EEF1B2, EIF4A1, ENO1, EZR, G3BP1, GPI, HIST2H2AC, HLA-A, IMPDH2, LDHA, LOC102724594/U2AF1, MAP4, MSN, MYH9, NACA, NSFL1C, PCNA, PFKP, PKM, PRDX1, PRPF19, SERBP1, TPI1, TUBB4B	33
Cancer, hematological disease, immunological disease, organismal injury and abnormalities	Peripheral t-cell lymphoma	2.47 × 10^-6^		CYR61, FN1, NNMT, PSMB1, PSMD2, TUBB, TUBB4B, TUBB6, TUBB8	9
Cell death and survival	Cell death of brain	2.52 × 10^-6^	0.904	CAPN1, CAPNS1, CAST, CDK1, DNM1L, FUS, GAPDH, HMGB1, HSPA5, HSPB1, HSPD1, HYOU1, MAP1B, P4HB, PARK7, PEA15, RPS3, SDHA, SOD1, STAT1, STIP1, TCP1, VAPA, YWHAB	24
Cancer	Metastatic cancer	2.58 × 10^-6^		ATIC, B2M, CAPG, CAV1, CSE1L, DPYSL2, EEF1A1, EIF4A1, FDPS, FKBP1A, FLNA, FN1, HSP90AA1, HSP90AB1, HSP90B1, KRT18, MYL12A, PLOD2, PPIA, RPL27, RPL7, RPS11, RPS27A, STAT1, TUBB, TUBB4B, YWHAE	27
Free radical scavenging	Production of reactive oxygen species	2.60 × 10^-6^	0.372	AKR1B1, ANXA1, ARHGDIA, CAV1, CD44, CTTN, DNM1L, FN1, G6PD, GNB2L1, HMGB1, HNRNPK, HSP90AB1, HSPA9, IMMT, LDHA, LONP1, MAPK1, PARK7, PPIA, PRDX1, PRDX2, RHOA, SOD1, SQSTM1, TAGLN, VDAC1, YWHAZ	28
Cellular movement	Migration of fibrosarcoma cell lines	2.60 × 10^-6^	−1.394	ARPC2, CTTN, FLNA, FLNB, FLNC, FN1, HNRNPK, TPM3	8
Cellular growth and proliferation	Outgrowth of cells	2.75 × 10^-6^	−0.580	APEX1, BASP1, CDK1, CYR61, DNM1L, DPYSL2, EZR, FKBP4, FN1, HMGB1, HSP90AA1, IQGAP1, ITGB1, LGALS1, MAP1B, MAPK1, MARCKS, MYH10, MYH9, NPM1, PDIA3, RAB11A, RHOA, RTN4, SLC25A5, SNX3, SOD1, SPTBN1, TNC, VAPA, VIM, YWHAZ	32
Cancer, hematological disease, immunological disease	Waldenstrom’s macroglobulinemia	2.79 × 10^-6^		ANXA2, ANXA6, CAV1, CDK1, FASN, FKBP1A, FN1, FUS, G3BP1, KPNA2, NONO, PCNA, PPP2CA, PSMB1, PSMD2, STIP1, XRCC6, YWHAE	18
Cancer, gastrointestinal disease	Upper gastrointestinal tract tumor	2.84 × 10^-6^		AKR1B1, ALYREF, ANXA1, ATIC, BSG, CANX, CAV1, CD44, COPB2, CSTB, CTNNA1, CTTN, DDX39B, EZR, FASN, FKBP1A, FN1, FUS, GSTP1, H3F3A/H3F3B, HDGF, HINT1, HLA-B, HNRNPH1, HSP90AA1, HSP90AB1, HSP90B1, IGF2R, IPO5, LGALS1, NPM1, P4HB, PA2G4, PCNA, PLOD2, PPP1CA, RAN, RHOA, RPL10, RPL22, SFN, TAGLN, TNC, TUBB, TUBB4B, XRCC5	46
Molecular transport, RNA trafficking	Transport of RNA	3.04 × 10^-6^	−1.969	ALYREF, DDX39B, DDX3X, EIF5A, FUS, HNRNPA2B1, KHDRBS1, RAN, U2AF2, XPO1	10
Cancer, hematological disease, immunological disease	Plasma cell dyscrasia	3.09 × 10^-6^		ANXA2, ANXA6, CAV1, CDK1, FASN, FDPS, FKBP1A, FLNA, FN1, FUS, G3BP1, HLA-A, KPNA2, NONO, PCNA, PDIA6, PPP2CA, PSMA2, PSMB1, PSMD2, STIP1, VIM, XRCC6, YWHAE, YWHAZ	25
Cell cycle	Arrest in interphase	3.10 × 10^-6^		ANXA2, CAV1, CD44, CDK1, CSE1L, CYR61, FASN, FKBP1A, FLNA, FN1, ITGB1, LGALS1, LMNA, MAPK1, MCM7, PDCD6, PPP1CA, PRKDC, PTGES3, RHOA, RPL23, RPL5, RPL7A, SFN, SPTAN1, STAT1, TCP1, TFRC, TMPO, YWHAG	30
Cell signaling, post-translational modification, protein synthesis	Actin capping of filament barbed ends	3.12 × 10^-6^		CAPG, CAPZA1, CAPZB, TWF1, TWF2	5
Cellular movement	Cell movement of bone cancer cell lines	3.21 × 10^-6^	−1.584	ACTN4, CAPN2, CAV1, CTSB, FN1, STMN1, TLN1, VCP	8
Hematological system development and function, inflammatory response, tissue development	Aggregation of blood platelets	3.42 × 10^-6^	−0.977	ACTG1, AKR1B1, CAPN1, CAST, CLIC1, CSRP1, FLNA, GNAI2, HSPB1, ITGB1, LGALS1, MAPK1, MYH9, MYL12A, P4HB, PDIA3, TLN1, VCL	18
Cancer	Cancer	3.45 × 10^-6^	−1.084	AARS, ACAT1, ACAT2, ACLY, ACTG1, ACTN1, ACTN4, ACTR2, ACTR3, AHCY, AHNAK, AKR1B1, ALDOA, ALDOC, ANXA1, ANXA2, ANXA5, ANXA6, AP1B1, APEX1, ARF1, ARHGDIA, ARPC2, ATIC, ATP5A1, ATP5B, B2M, BASP1, BSG, C14orf166, C1QBP, CACYBP, CALR, CAND1, CANX, CAP1, CAPG, CAPN1, CAPN2, CAPRIN1, CAPZA1, CAST, CAV1, CBX3, CCT2, CCT3, CCT4, CCT5, CCT6A, CCT7, CCT8, CD44, CD59, CDK1, CFL1, CLIC1, CLTC, CNN3, COPA, COPB1, COPB2, COPE, COPG1, CORO1C, COTL1, CRIP2, CSE1L, CSTB, CTNNA1, CTPS1, CTSA, CTSB	406
				CTTN, CUTA, CYB5R3, CYC1, CYR61, DBI, DBN1, DDX1, DDX39B, DDX3X, DDX5, DHX9, DLAT, DNAJA1, DNAJA2, DNAJC8, DNM1L, DPYSL2, DYNC1H1, DYNC1I2, ECHS1, EEF1A1, EEF1B2, EEF1D, EEF2, EHD1, EIF2S1, EIF2S2, EIF3B, EIF3C, EIF3I, EIF3M, EIF4A1, EIF4G1, EIF4H, ENAH, ENO1, ENO2, EPRS, ESYT1, ETFA, EZR, FASN, FDPS, FKBP1A, FLNA, FLNB, FLNC, FN1, FUBP3, FUS, G3BP1, G6PD, GANAB, GAPDH, GARS, GCN1L1, GDI1, GDI2, GLO1, GLUD1, GML, GMPS, GNAI2, GNB2L1, GPI, GSTP1, H2AFY, H3F3A/H3F3B, HADHA, HARS, HDGF, HDLBP, HEXB, HINT1, HIST1H1C, HIST1H2BL, HIST2H2AC, HLA-A, HLA-B, HMGB1, HN1, HNRNPA1, HNRNPA2B1, HNRNPA3, HNRNPH1, HNRNPH3, HNRNPK, HNRNPL, HNRNPM, HNRNPR, HNRNPU, HSP90AA1, HSP90AB1, HSP90B1, HSPA4, HSPA5, HSPA8, HSPA9, HSPB1, HSPD1, HYOU1, IARS, IDI1, IFITM3, IGF2R, ILF2, ILF3, IMMT, IMPDH2, IPO5, IPO7, IPO9, IQGAP1, ITGB1, KHDRBS1, KHSRP, KIF5B, KPNA2, KPNA3, KPNB1, KRT1, KRT18, KRT8, KRT9, LAMB1, LASP1, LDHA, LGALS1, LMNA, LMNB1, LOC102724594/U2AF1, LONP1, LRPPRC, LRRC59, MAP1B, MAP4, MAPK1, MAPRE1, MARCKS, MARS, MATR3, MCM3, MCM4, MCM6, MCM7, MDH1, MDH2, MSN, MVP, MYH10, MYH9, MYL12A, NACA, NAP1L1, NAP1L4, NASP, NCL, NNMT, NONO, NPEPPS, NPLOC4, NPM1, NSFL1C, NUMA1, OLA1, P4HB, PA2G4, PABPC1, PAFAH1B2, PAICS, PCBP1, PCBP2, PCM1, PCMT1, PCNA, PDCD6, PDE6H, PDIA3, PDIA4, PDIA6, PDLIM1, PDXK, PEA15, PFKP, PFN1, PGAM1, PGD, PGK1, PGM1, PHGDH, PICALM, PKM, PLEC, PLIN3, PLOD2, PLS3, PPIA, PPM1G, PPP1CA, PPP2CA, PPP2R1A, PRDX1, PRDX2, PRDX5, PRKCSH, PRKDC, PRMT5, PRPF19, PSMA1, PSMA2, PSMA7, PSMB1, PSMC1, PSMC4, PSMD2, PSME1, PTBP1, PTMA, PTRF, PUF60, PYGB, PYGL, QARS, RAN, RANBP1, RARS, RBM3, RCN1, RHOA, RNH1, RPL10, RPL12, RPL22, RPL27, RPL3, RPL4, RPL5, RPL6, RPL7, RPL7A, RPL8, RPLP0, RPN1, RPN2, RPS11, RPS12, RPS2, RPS24, RPS27A, RPS3, RPS3A, RPS4X, RPS5, RPS7, RRBP1, RTN4, S100A6, SDHA, SEPT9, SERBP1, SERPINH1, SET, SF3A1, SF3B1, SFN, SFPQ, SH3BGRL3, SHMT2, SLC25A3, SLC25A5, SLC25A6, SLC38A2, SND1, SNRPD3, SOD1, SPTAN1, SPTBN1, SQSTM1, SSRP1, STAT1, STIP1, STMN1, STOML2, SYNCRIP, TAGLN, TAGLN2, TALDO1, TARS, TCP1, TFRC, TLN1, TMPO, TNC, TNPO1, TPI1, TPM2, TPM3, TPM4, TUBA1B, TUBB, TUBB4B, TUBB6, TUBB8, TWF2, TXLNA, TXNDC5, UAP1, UBA1, UBE2N, UBXN4, UCHL1, UQCRC1, VARS, VAT1, VCL, VCP, VDAC2, VIM, VPS35, WARS, WDR1, XPO1, XRCC5, XRCC6, YBX1, YBX3, YWHAB, YWHAE, YWHAG, YWHAH, YWHAQ, YWHAZ, ZYX	
Cell morphology	Blebbing	3.47 × 10^-6^	0.053	CTTN, DPYSL2, EZR, HSPB1, LMNA, LMNB1, MARCKS, RHOA, SPTAN1	9
Hereditary disorder, neurological disease, organismal injury and abnormalities, skeletal and muscular disorders	Charcot-Marie-tooth disease type 2	3.75 × 10^-6^		AARS, DYNC1H1, GARS, HSPB1, LMNA, RAB7A	6
Endocrine system development and function, small molecule biochemistry	Binding of hormone	3.75 × 10^-6^	0.250	DNAJA1, FKBP4, HSP90AB1, PTGES3, SFN, STIP1	6
Cancer, hematological disease, immunological disease, organismal injury and abnormalities	Classic Hodgkin disease	3.75 × 10^-6^		FKBP1A, LGALS1, TUBB, TUBB4B, TUBB6, TUBB8	6
Cellular assembly and organization, cellular function and maintenance	Stabilization of microtubules	3.80 × 10^-6^	0.247	COPB2, DNM1L, DPYSL2, IQGAP1, MAP1B, MAP4, MAPRE1, NUMA1, PKM, RHOA, SEPT7, STMN1	12
Cell morphology, cellular function and maintenance	Transmembrane potential of mitochondria	4.05 × 10^-6^	−0.508	ANXA6, B2M, CLIC1, CLIC4, HSPA4, HSPB1, HSPD1, IMMT, LDHA, LGALS1, LONP1, PARK7, PHB2, SLC25A6, SOD1, STOML2, VCP, VDAC1, YWHAE	19
Cancer, gastrointestinal disease	Digestive organ tumor	4.26 × 10^-6^	−0.321	AARS, ACAT1, ACAT2, ACLY, ACTG1, ACTN1, ACTR2, ACTR3, AHCY, AHNAK, AKR1B1, ALYREF, ANXA1, ANXA2, AP1B1, APEX1, ARF1, ARHGDIA, ATIC, ATP5A1, ATP5B, B2M, BSG, CACYBP, CANX, CAPG, CAPN2, CAST, CAV1, CBX3, CCT4, CCT5, CCT6A, CCT8, CD44, CD59, CLIC1, CLTC, COPA, COPB2, COPG1, COTL1, CRIP2, CSE1L, CSTB, CTNNA1, CTPS1, CTSA, CTSB, CTTN, CUTA, CYC1, CYR61, DBI, DBN1, DDX1, DDX39B, DDX3X, DDX5, DHX9, DLAT, DNAJA1, DNAJC8, DNM1L, DPYSL2, DYNC1H1, DYNC1I2, ECHS1, EEF1A1, EEF1D, EEF2, EIF3I, EIF4A1, EIF4G1, ENAH, ENO1, ENO2, EPRS, ESYT1, EZR, FASN, FKBP1A, FLNA, FLNB, FLNC, FN1, FUBP3, FUS, G3BP1, G6PD, GANAB, GARS, GCN1L1, GDI2, GLUD1, GML, GMPS, GNAI2, GNB2L1, GPI, GSTP1, H2AFY, H3F3A/H3F3B, HADHA, HDGF, HDLBP, HEXB, HINT1, HIST1H1C, HLA-A, HLA-B, HMGB1, HNRNPA1, HNRNPA2B1, HNRNPA3, HNRNPH1, HNRNPH3, HNRNPK, HNRNPL, HNRNPM, HNRNPU, HSP90AA1, HSP90AB1, HSP90B1, HSPA5, HSPA8, HSPA9, HSPD1, HYOU1, IARS, IDI1, IFITM3, IGF2R, ILF2, ILF3, IMMT, IMPDH2, IPO5, IPO7, IQGAP1, ITGB1, KHDRBS1, KHSRP, KIF5B, KPNA2, KPNA3, KPNB1, KRT1, KRT18, KRT8, KRT9, LAMB1, LDHA, LGALS1, LMNA, LMNB1, LOC102724594/U2AF1, LONP1, LRPPRC, LRRC59, MAP1B, MAP4, MAPRE1, MARS, MATR3, MCM3, MCM4, MCM6, MCM7, MDH1, MDH2, MVP, MYH10, MYH9, NACA, NAP1L1, NAP1L4, NASP, NCL, NNMT, NPEPPS, NPLOC4, NPM1, NUMA1, OLA1, P4HB, PA2G4, PAICS, PCBP1, PCBP2, PCM1, PCNA, PDIA4, PFKP, PFN1, PGAM1, PGD, PGK1, PGM1, PHGDH, PKM, PLEC, PLIN3, PLOD2, PLS3, PPM1G, PPP1CA, PPP2CA, PPP2R1A, PRDX1, PRKCSH, PRKDC, PSMA7, PSMC1, PSMD2, PTBP1, PTMA, PTRF, PUF60, PYGB, PYGL, QARS, RAN, RANBP1, RARS, RHOA, RPL10, RPL12, RPL22, RPL4, RPL5, RPL7A, RPLP0, RPN1, RPN2, RPS2, RPS24, RPS4X, RRBP1, RTN4, S100A6, SDHA, SEPT9, SERBP1, SERPINH1, SET, SF3A1, SF3B1, SFN, SFPQ, SH3BGRL3, SLC38A2, SND1, SOD1, SPTAN1, SPTBN1, SQSTM1, SSRP1, STAT1, STIP1, STMN1, SYNCRIP, TAGLN, TALDO1, TARS, TCP1, TFRC, TLN1, TMPO, TNC, TNPO1, TPI1, TPM2, TPM4, TUBB, TUBB4B, TUBB8, TWF2, UAP1, UBA1, UBE2N, UBXN4, UCHL1, VARS, VCL, VCP, VIM, VPS35, WARS, XRCC5, XRCC6, YBX1, YBX3, YWHAE, YWHAG, YWHAH, YWHAZ	297
Cellular assembly and organization, tissue development	Polymerization of filaments	4.45 × 10^-6^	0.934	ACTR3, ARPC2, CAPZB, CAV1, CFL1, FKBP4, MAP1B, MAPRE1, PFN1, RHOA, STMN1, TUBB, TWF1, TWF2	14
Cellular assembly and organization, cellular development, cellular growth and proliferation, nervous system development and function, tissue development	Growth of neurites	4.55 × 10^-6^	−1.344	APEX1, BASP1, CAV1, CDK1, CSRP1, DNM1L, DPYSL2, EZR, FKBP4, FN1, HMGB1, IQGAP1, ITGB1, LGALS1, MAP1B, MAPK1, MARCKS, MYH10, MYH9, PDIA3, RAB11A, RHOA, RTN4, SEPT9, SLC25A5, SNX3, SOD1, SPTBN1, TNC, VAPA, VCL, VIM, YWHAZ	33
Hematological disease	Blood protein disorder	4.60 × 10^-6^		ANXA2, ANXA6, ARHGDIA, B2M, CAV1, CDK1, FASN, FDPS, FKBP1A, FLNA, FN1, FUS, G3BP1, HLA-A, KPNA2, NONO, PCNA, PDIA6, PPP2CA, PSMA2, PSMB1, PSMD2, STIP1, VIM, XRCC6, YWHAE, YWHAZ	27
Hereditary disorder	Autosomal dominant disease	4.66 × 10^-6^		AARS, ACTA1, ACTG1, ACTN4, AHNAK, CAV1, DNM1L, DYNC1H1, EEF2, EIF4G1, FKBP1A, FLNB, FLNC, FN1, FUS, GARS, HNRNPA1, HNRNPA2B1, HSPB1, HSPD1, KRT1, KRT9, LMNA, LMNB1, MYH9, PARK7, RAB7A, SOD1, STAT1, TNC, TPM2, TPM3, UCHL1, VCP, VPS35	35
Cancer, immunological disease, organismal injury and abnormalities	Lymphatic node tumor	4.71 × 10^-6^	0.000	ANXA1, ATIC, B2M, CSE1L, CYR61, FKBP1A, FN1, FUS, HIST1H1C, HMGB1, IQGAP1, ITGB1, KHDRBS1, LONP1, NNMT, PRDX1, PRKDC, PSMB1, PSMD2, RHOA, RPS2, SF3B1, SHMT2, STMN1, TUBB, TUBB4B, TUBB6, TUBB8, TXLNA, UQCRC1, XPO1, XRCC5, YWHAZ	33
Molecular transport	Nuclear export	4.97 × 10^-6^		ALYREF, CALR, CSE1L, DDX39B, EIF5A, HNRNPA1, HSPA9, KHDRBS1, NPM1, RAN, XPO1	11
Cell death and survival	Apoptosis of colon cancer cell lines	5.12 × 10^-6^	1.294	AHSA1, CD44, CDK1, CSE1L, FASN, GSTP1, HINT1, HSPD1, IGF2R, ITGB1, KRT18, PARK7, PPIA, RHOA, SFN, SFPQ, STAT1, VCP, XRCC5, YWHAE	20
Cellular movement	Cell movement of pericytes	5.37 × 10^-6^	−0.928	CD44, FN1, GNB2L1, HMGB1, LGALS1, MAPK1, MYH10	7
Molecular transport, protein synthesis, protein trafficking	Localization of protein	5.46 × 10^-6^	−0.324	APEX1, ARHGDIA, BTF3, C1QBP, CAV1, CUTA, FKBP1A, FKBP4, GNB2L1, HLA-A, HYOU1, ITGB1, NPM1, PICALM, SEPT2, SQSTM1, TFRC	17
Cellular movement	Migration of connective tissue cells	5.46 × 10^-6^	0.420	CAPNS1, CAV1, CD44, CYR61, EHD1, FLNB, FN1, GNB2L1, HMGB1, ITGB1, LGALS1, MAPK1, MYH10, PLEC, STMN1, VCL, ZYX	17
Hereditary disorder, skeletal and muscular disorders	Autosomal dominant myopathy	6.79 × 10^-6^		AARS, AHNAK, DYNC1H1, GARS, HSPB1, LMNA, RAB7A	7
Neurological disease	Lower motor neuron disease	6.79 × 10^-6^		DYNC1H1, GARS, HNRNPA1, HNRNPA2B1, HSPB1, UBA1, VCP	7
Cancer, endocrine system disorders	Thyroid adenoma	6.79 × 10^-6^		ANXA5, CALR, CTSB, GSTP1, HSP90AB1, PARK7, PRDX2	7
Cell death and survival	Apoptosis of cervical cancer cell lines	7.05 × 10^-6^	−0.772	CCT4, CDK1, CTSB, DNM1L, DYNC1H1, EZR, FLNB, HNRNPK, IMMT, KHDRBS1, MAPK1, MSN, PCBP2, PPIA, PRKDC, PRPF19, PTMA, RPS24, STAT1, TCP1, UCHL1, VDAC1	22
Cell morphology	Shape change of tumor cell lines	7.13 × 10^-6^	−1.937	ANXA5, CAP1, CAPN2, DPYSL2, FLNA, FLNB, FN1, ITGB1, MAPK1, MARCKS, MYH9, PFN1, RHOA, TNC, UCHL1	15
Energy production, molecular transport, nucleic acid metabolism, small molecule biochemistry	Concentration of ATP	7.22 × 10^-6^	−1.019	ALDOA, ATP5B, CAV1, DNM1L, ENO1, HSD17B10, KRT8, LDHA, LRPPRC, PKM, SQSTM1, STOML2, VDAC1	13
Cell death and survival	Cell death of brain cells	7.42 × 10^-6^	1.086	CAPN1, CAPNS1, CAST, CDK1, DNM1L, FUS, GAPDH, HMGB1, HSPA5, HSPB1, HSPD1, HYOU1, MAP1B, P4HB, PARK7, PEA15, RPS3, SOD1, STIP1, TCP1, VAPA, YWHAB	22
Cancer, gastrointestinal disease	Digestive tract cancer	7.58 × 10^-6^	0.345	AARS, ACAT1, ACAT2, ACLY, ACTG1, ACTN1, ACTR2, ACTR3, AHCY, AHNAK, AKR1B1, ANXA1, ANXA2, AP1B1, APEX1, ARF1, ARHGDIA, ATIC, ATP5A1, ATP5B, B2M, BSG, CACYBP, CANX, CAPG, CAPN2, CAST, CAV1, CBX3, CCT4, CCT5, CCT6A, CCT8, CD44, CD59, CLIC1, CLTC, COPA, COPB2, COPG1, COTL1, CRIP2, CSE1L, CSTB, CTNNA1, CTPS1, CTSA, CTSB, CTTN, CUTA, CYC1, CYR61, DBI, DBN1, DDX1, DDX39B, DDX3X, DDX5, DHX9, DLAT, DNAJA1, DNAJC8, DNM1L, DPYSL2, DYNC1H1, DYNC1I2, ECHS1, EEF1A1, EEF1D, EEF2, EIF3I, EIF4A1, EIF4G1, ENAH, ENO1, ENO2, EPRS, ESYT1, EZR, FASN, FKBP1A, FLNA, FLNB, FLNC, FN1, FUBP3, FUS, G3BP1, G6PD, GANAB, GARS, GCN1L1, GDI2, GLUD1, GML, GMPS, GNAI2, GNB2L1, GPI, GSTP1, H2AFY, H3F3A/H3F3B, HADHA, HDGF, HDLBP, HEXB, HINT1, HIST1H1C, HLA-A, HLA-B, HMGB1, HNRNPA1, HNRNPA2B1, HNRNPA3, HNRNPH1, HNRNPH3, HNRNPK, HNRNPL, HNRNPM, HNRNPU, HSP90AA1, HSP90AB1, HSP90B1, HSPA5, HSPA8, HSPA9, HSPD1, HYOU1, IARS, IDI1, IFITM3, IGF2R, ILF2, ILF3, IMMT, IMPDH2, IPO5, IPO7, IQGAP1, ITGB1, KHDRBS1, KHSRP, KIF5B, KPNA2, KPNA3, KPNB1, KRT1, KRT18, KRT8, KRT9, LAMB1, LDHA, LGALS1, LMNA, LMNB1, LOC102724594/U2AF1, LONP1, LRPPRC, LRRC59, MAP1B, MAP4, MAPRE1, MARS, MATR3, MCM3, MCM4, MCM6, MCM7, MDH1, MDH2, MVP, MYH10, MYH9, NACA, NAP1L1, NAP1L4, NASP, NCL, NNMT, NPEPPS, NPLOC4, NPM1, NUMA1, OLA1, P4HB, PA2G4, PAICS, PCBP1, PCBP2, PCM1, PCNA, PDIA4, PFKP, PFN1, PGAM1, PGD, PGK1, PGM1, PHGDH, PKM, PLEC, PLIN3, PLOD2, PLS3, PPM1G, PPP1CA, PPP2CA, PPP2R1A, PRDX1, PRKCSH, PRKDC, PSMA7, PSMC1, PSMD2, PTBP1, PTMA, PTRF, PUF60, PYGB, PYGL, QARS, RAN, RANBP1, RARS, RHOA, RPL10, RPL12, RPL22, RPL4, RPL5, RPL7A, RPLP0, RPN1, RPN2, RPS2, RPS24, RPS4X, RRBP1, RTN4, S100A6, SDHA, SEPT9, SERBP1, SERPINH1, SET, SF3A1, SF3B1, SFN, SFPQ, SH3BGRL3, SLC38A2, SND1, SOD1, SPTAN1, SPTBN1, SQSTM1, SSRP1, STAT1, STIP1, SYNCRIP, TAGLN, TALDO1, TARS, TCP1, TFRC, TLN1, TMPO, TNC, TNPO1, TPI1, TPM2, TPM4, TUBB, TUBB4B, TUBB8, TWF2, UAP1, UBA1, UBE2N, UBXN4, UCHL1, VARS, VCL, VCP, VIM, VPS35, WARS, XRCC6, YBX1, YBX3, YWHAE, YWHAG, YWHAH, YWHAZ	294
Cancer, gastrointestinal disease	Malignant neoplasm of digestive system	7.58 × 10^-6^	0.345	AARS, ACAT1, ACAT2, ACLY, ACTG1, ACTN1, ACTR2, ACTR3, AHCY, AHNAK, AKR1B1, ANXA1, ANXA2, AP1B1, APEX1, ARF1, ARHGDIA, ATIC, ATP5A1, ATP5B, B2M, BSG, CACYBP, CANX, CAPG, CAPN2, CAST, CAV1, CBX3, CCT4, CCT5, CCT6A, CCT8, CD44, CD59, CLIC1, CLTC, COPA, COPB2, COPG1, COTL1, CRIP2, CSE1L, CSTB, CTNNA1, CTPS1, CTSA, CTSB, CTTN, CUTA, CYC1, CYR61, DBI, DBN1, DDX1, DDX39B, DDX3X, DDX5, DHX9, DLAT, DNAJA1, DNAJC8, DNM1L, DPYSL2, DYNC1H1, DYNC1I2, ECHS1, EEF1A1, EEF1D, EEF2, EIF3I, EIF4A1, EIF4G1, ENAH, ENO1, ENO2, EPRS, ESYT1, EZR, FASN, FKBP1A, FLNA, FLNB, FLNC, FN1, FUBP3, FUS, G3BP1, G6PD, GANAB, GARS, GCN1L1, GDI2, GLUD1, GML, GMPS, GNAI2,	294
				GNB2L1, GPI, GSTP1, H2AFY, H3F3A/H3F3B, HADHA, HDGF, HDLBP, HEXB, HINT1, HIST1H1C, HLA-A, HLA-B, HMGB1, HNRNPA1, HNRNPA2B1, HNRNPA3, HNRNPH1, HNRNPH3, HNRNPK, HNRNPL, HNRNPM, HNRNPU, HSP90AA1, HSP90AB1, HSP90B1, HSPA5, HSPA8, HSPA9, HSPD1, HYOU1, IARS, IDI1, IFITM3, IGF2R, ILF2, ILF3, IMMT, IMPDH2, IPO5, IPO7, IQGAP1, ITGB1, KHDRBS1, KHSRP, KIF5B, KPNA2, KPNA3, KPNB1, KRT1, KRT18, KRT8, KRT9, LAMB1, LDHA, LGALS1, LMNA, LMNB1, LOC102724594/U2AF1, LONP1, LRPPRC, LRRC59, MAP1B, MAP4, MAPRE1, MARS, MATR3, MCM3, MCM4, MCM6, MCM7, MDH1, MDH2, MVP, MYH10, MYH9, NACA, NAP1L1, NAP1L4, NASP, NCL, NNMT, NPEPPS, NPLOC4, NPM1, NUMA1, OLA1, P4HB, PA2G4, PAICS, PCBP1, PCBP2, PCM1, PCNA, PDIA4, PFKP, PFN1, PGAM1, PGD, PGK1, PGM1, PHGDH, PKM, PLEC, PLIN3, PLOD2, PLS3, PPM1G, PPP1CA, PPP2CA, PPP2R1A, PRDX1, PRKCSH, PRKDC, PSMA7, PSMC1, PSMD2, PTBP1, PTMA, PTRF, PUF60, PYGB, PYGL, QARS, RAN, RANBP1, RARS, RHOA, RPL10, RPL12, RPL22, RPL4, RPL5, RPL7A, RPLP0, RPN1, RPN2, RPS2, RPS24, RPS4X, RRBP1, RTN4, S100A6, SDHA, SEPT9, SERBP1, SERPINH1, SET, SF3A1, SF3B1, SFN, SFPQ, SH3BGRL3, SLC38A2, SND1, SOD1, SPTAN1, SPTBN1, SQSTM1, SSRP1, STAT1, STIP1, SYNCRIP, TAGLN, TALDO1, TARS, TCP1, TFRC, TLN1, TMPO, TNC, TNPO1, TPI1, TPM2, TPM4, TUBB, TUBB4B, TUBB8, TWF2, UAP1, UBA1, UBE2N, UBXN4, UCHL1, VARS, VCL, VCP, VIM, VPS35, WARS, XRCC6, YBX1, YBX3, YWHAE, YWHAG, YWHAH, YWHAZ	
Molecular transport	Export of molecule	7.90 × 10^-6^	−1.118	ALYREF, ANXA6, CALR, CSE1L, DDX39B, EIF5A, HSPA9, KHDRBS1, RAN, SEC13, U2AF2, XPO1, YWHAE	13
Cellular assembly and organization	Quantity of filaments	8.03 × 10^-6^	−0.914	CFL1, FN1, GNB2L1, KRT18, MAP1B, MAP4, MARCKS, PLEC, RHOA, SERPINH1, SPTAN1, STMN1, TMOD3, VIM	14
Cell-to-cell signaling and interaction, reproductive system development and function	Binding of sperm	8.32 × 10^-6^		CCT2, CCT3, CCT4, CCT5, CCT6A, CCT7, CCT8, TCP1	8
Connective tissue disorders, developmental disorder, metabolic disease, skeletal and muscular disorders	Paget’s disease of bone	8.37 × 10^-6^		FDPS, HNRNPA1, HNRNPA2B1, SQSTM1, VCP	5
Cancer, hematological disease, immunological disease, organismal injury and abnormalities	Refractory classical hodgkin lymphoma	8.37 × 10^-6^		FKBP1A, TUBB, TUBB4B, TUBB6, TUBB8	5
Cancer, hematological disease, immunological disease, organismal injury and abnormalities	Relapsed classical hodgkin lymphoma	8.37 × 10^-6^		FKBP1A, TUBB, TUBB4B, TUBB6, TUBB8	5
Infectious disease	Infection of hepatoma cell lines	8.85 × 10^-6^	−0.425	ACTR2, AP1B1, AP2B1, ARPC1B, CLTC, G3BP1, HMGB1, IFITM3, TFRC	9
Cancer, gastrointestinal disease	Upper gastrointestinal tract cancer	8.86 × 10^-6^		ANXA1, ATIC, BSG, CANX, CAV1, CD44, COPB2, CSTB, CTNNA1, CTTN, DDX39B, EZR, FASN, FKBP1A, FN1, FUS, GSTP1, H3F3A/H3F3B, HDGF, HINT1, HLA-B, HNRNPH1, HSP90AA1, HSP90AB1, HSP90B1, IGF2R, IPO5, LGALS1, NPM1, P4HB, PA2G4, PLOD2, RAN, RHOA, RPL10, RPL22, SFN, TAGLN, TNC, TUBB, TUBB4B	41
Hematological disease, organismal injury and abnormalities	Myelodysplastic syndrome	9.44 × 10^-6^		CALR, LOC102724594/U2AF1, NPM1, PDIA3, RPL3, RPL4, RPL5, RPL6, RPL7, RPLP0, RPLP1, RPS27A, RPS4X, SF3B1	14
Cancer	Osteosarcoma	9.56 × 10^-6^		CD44, FASN, FDPS, HINT1, HSP90AA1, HSP90AB1, HSP90B1, IMPDH2, PRDX1, STOML2, TUBB, TUBB4B	12
Cellular function and maintenance	Uptake of cells	9.91 × 10^-6^	0.555	C1QBP, CAV1, ITGB1, NCL, OTUB1, RHOA, RPSA, SNX3	8
Cellular development, cellular growth and proliferation	Proliferation of carcinoma Cell lines	1.06 × 10^-5^	−0.759	ACLY, ANXA1, ANXA2, BSG, C1QBP, CAV1, CYR61, DYNC1H1, GAPDH, H2AFY, HMGB1, HNRNPA2B1, IGF2R, ITGB1, MAPK1, MAPRE1, PTBP1, S100A6, SET, SLC25A6, SOD1, STMN1, TRIM28, UCHL1	24
Cell death and survival	Apoptosis of breast cancer Cell lines	1.16 × 10^-5^	−1.394	ARHGDIA, B2M, CAV1, CD44, CDK1, CSE1L, CYR61, FASN, FN1, HINT1, HSPA5, HSPB1, HSPD1, KPNA2, KRT18, MAPK1, PEA15, PEBP1, SND1, STAT1, TFRC, VCP	22
Cell-to-cell signaling and interaction, connective tissue development and function, tissue development	Adhesion of fibroblast Cell lines	1.18 × 10^-5^	−1.591	CALR, CD44, CYR61, FN1, IQGAP1, ITGB1, MAPK1, RHOA, VCL	9
Cellular assembly and organization, cellular compromise	Formation of cellular inclusion bodies	1.21 × 10^-5^	0.246	CTSB, DNAJA1, FUS, HSPA4, KRT18, KRT8, PSMC4, SOD1, SQSTM1, TCP1, UCHL1	11
Cell-to-cell signaling and interaction	Binding of lymphoma cell lines	1.31 × 10^-5^	−0.881	ANXA2, CD44, FN1, ITGB1, NCL, RHOA, TFRC	7
Organismal injury and abnormalities, renal and urological disease	Nephrosis	1.35 × 10^-5^		ACTN4, ANXA1, ARHGDIA, CLTC, FKBP1A, IMPDH2, ITGB1, LAMB1, PDLIM1	9
Cancer, gastrointestinal disease, respiratory disease	CDKN2A positive oropharyngeal squamous cell carcinoma	1.35 × 10^-5^		HSP90AA1, HSP90AB1, HSP90B1	3
Cancer, respiratory disease	Advanced stage primary laryngeal cancer	1.35 × 10^-5^		HSP90AA1, HSP90AB1, HSP90B1	3
Gene expression	Binding of Y box	1.35 × 10^-5^		APEX1, YBX1, YBX3	3
Connective tissue disorders, developmental disorder, hereditary disorder, metabolic disease, skeletal and muscular disorders	Familial Paget’s disease of bone	1.35 × 10^-5^		HNRNPA1, HNRNPA2B1, VCP	3
Cellular movement	Initiation of migration of fibrosarcoma cell lines	1.35 × 10^-5^		FLNA, FLNB, FLNC	3
Cancer, organismal injury and abnormalities, reproductive system disease	Locally advanced cervical cancer	1.35 × 10^-5^		HSP90AA1, HSP90AB1, HSP90B1	3
Cancer, immunological disease, organismal injury and abnormalities, reproductive system disease	Node positive cervical cancer	1.35 × 10^-5^		HSP90AA1, HSP90AB1, HSP90B1	3
Cell morphology, cellular assembly and organization, cellular development, cellular growth and proliferation, nervous system development and function, tissue development	Outgrowth of neurites	1.36 × 10^-5^	−1.232	APEX1, BASP1, CDK1, DNM1L, DPYSL2, EZR, FKBP4, FN1, HMGB1, IQGAP1, ITGB1, LGALS1, MAP1B, MAPK1, MARCKS, MYH10, MYH9, PDIA3, RAB11A, RHOA, RTN4, SLC25A5, SNX3, SOD1, SPTBN1, TNC, VAPA, VIM, YWHAZ	29
Cancer, tumor morphology	Invasion of malignant tumor	1.38 × 10^-5^	−2.111	AHCY, BSG, CAPN2, CD44, CTSB, CTTN, EZR, FASN, HMGB1, HNRNPA1, HSPA5, ITGB1, LGALS1, MAPK1, VIM	15
Cellular assembly and organization, cellular function and maintenance	Quantity of cellular protrusions	1.38 × 10^-5^	0.905	ACTG1, ACTR2, ARPC2, BSG, CANX, CAPZB, CTTN, ENAH, EZR, FN1, MAP1B, MSN, SOD1, TLN1, TPM3	15
Cell cycle	Interphase	1.57 × 10^-5^	0.828	ANXA2, CAPNS1, CAV1, CD44, CDK1, CSE1L, CYR61, DDX3X, FASN, FKBP1A, FLNA, FN1, GNB2L1, GPI, ITGB1, LGALS1, LMNA, MAPK1, MCM7, NASP, NPM1, PCNA, PDCD6, PEA15, PPP1CA, PRKDC, PTGES3, RHOA, RPL23, RPL5, RPL7A, SFN, SPTAN1, STAT1, TCP1, TFRC, TMPO, XRCC5, YWHAG, YWHAQ	40
Cell morphology, cellular function and maintenance	Repair of cells	1.62 × 10^-5^	0.131	APEX1, KPNA2, NPM1, OTUB1, PCNA, PRKDC, UBE2N, XRCC5, XRCC6, YBX1	10
Protein synthesis	Oligomerization of protein	1.63 × 10^-5^		ACAT1, AHNAK, ANXA5, ANXA6, CAV1, CTNNA1, CUTA, EEF1A1, EHD1, LONP1, NPM1, PRKCSH, SEPT7, SEPT9, SQSTM1, STOML2, TRIM28, VCP, YWHAB	19
Cellular movement	Migration of pericytes	1.68 × 10^-5^	−0.600	CD44, FN1, GNB2L1, HMGB1, LGALS1, MYH10	6
Cellular development, nervous system development and function, tissue development	Development of neurons	1.85 × 10^-5^	−0.428	ACTR3, BASP1, CAPNS1, CAPRIN1, CAPZB, CAV1, CD44, CSRP1, CYR61, DBN1, DNM1L, DPYSL2, EHD1, ENAH, EZR, GDI1, HMGB1, HNRNPK, HSPB1, ITGB1, LAMB1, MAP1B, MSN, MYH10, NNMT, PDIA3, PFN1, PHGDH, PICALM, PPP2CA, PRKCSH, RAB11A, RHOA, RTN4, SEPT2, SOD1, SPTBN1, STIP1, STMN1, TNC, UCHL1, VAPA, VIM, YWHAG, YWHAH	45
Hereditary disorder, neurological disease, organismal injury and abnormalities, skeletal and muscular disorders	Autosomal dominant Charcot-Marie-Tooth disease type 2	1.88 × 10^-5^		AARS, DYNC1H1, GARS, HSPB1, RAB7A	5
Cell morphology, hair and skin development and function	Cell spreading of epithelial cell lines	1.88 × 10^-5^	−1.446	CYR61, FLNA, FN1, ITGB1, VIM	5
Cell morphology, cellular function and maintenance, DNA replication, recombination, and repair	Double-stranded DNA break repair of cells	1.91 × 10^-5^	0.131	KPNA2, NPM1, OTUB1, PCNA, PRKDC, UBE2N, XRCC5, XRCC6	8
Cell death and survival	Apoptosis of central nervous system cells	1.96 × 10^-5^	−0.963	CAPN1, CAST, CDK1, DNM1L, GAPDH, HSP90AA1, HSPA5, MAP1B, NPM1, P4HB, PEA15, RHOA, RPS3, SOD1, YWHAB	15
Cell cycle	Arrest in G1 phase	1.97 × 10^-5^		CYR61, FASN, FKBP1A, FN1, ITGB1, LGALS1, LMNA, MAPK1, MCM7, PPP1CA, PRKDC, PTGES3, RHOA, RPL23, RPL5, RPL7A, SPTAN1, TFRC, TMPO, YWHAG	20
Cellular function and maintenance	Cellular homeostasis	1.98 × 10^-5^	0.822	AKR1B1, ALDOA, ANXA1, ANXA2, ANXA6, ARF1, ATP1A1, B2M, BSG, CALR, CAPN1, CAPNS1, CAST, CAV1, CLIC1, CLIC4, CLTC, CTTN, CYR61, DNM1L, EEF1D, EIF2S1, EIF4G1, FKBP1A, FN1, GPI, H2AFY, HADH, HEXB, HMGB1, HNRNPL, HSP90AA1, HSP90B1, HSPA4, HSPA5, HSPB1, HSPD1, IGF2R, IMMT, ITGB1, KIF5B, KRT18, KRT8, LDHA, LGALS1, LONP1, MAPK1, PAFAH1B2, PARK7, PGM1, PHB2, PICALM, PPIA, PPP1CA, PPP2CA, PRDX2, PRKDC, PSMD2, PYGL, RAB11A, RAB7A, RPL22, SEPT9, SERPINH1, SLC25A6, SOD1, SQSTM1, STAT1, STOML2, TFRC, TUFM, UBE2N, VCP, VDAC1, XRCC5, XRCC6, YWHAE	77
Tissue development	Growth of connective tissue	2.07 × 10^-5^	0.381	ANXA2, ATIC, CAPNS1, CAV1, CD44, CLTC, CTSB, CYR61, DDX5, DNM1L, FN1, GNAI2, GPI, GSTP1, HDGF, HNRNPA2B1, HSPB1, ITGB1, KHDRBS1, LGALS1, LMNA, LMNB1, MAPK1, MYH10, MYH9, NPM1, PA2G4, PGK1, PRDX4, PRKDC, PTGES3, RBM3, RHOA, SERPINH1, SFN, STAT1, STMN1, TPM3, VCL, YBX1	40
Post-translational modification, protein folding	Folding maturation of protein	2.09 × 10^-5^		AIP, CALR, FKBP1A, PRDX4	4
Cellular movement	Invasion of breast cancer cell lines	2.24 × 10^-5^	0.051	BSG, CALR, CAV1, CD44, CSE1L, CTSB, CTTN, ENAH, EZR, FN1, HSP90AA1, ILF3, IQGAP1, KRT8, MAPK1, PTGES3, RHOA, SEPT9, STMN1	19
Cellular function and maintenance, inflammatory response	Phagocytosis	2.34 × 10^-5^	−0.334	ANXA1, ANXA5, CALR, CAPG, CAV1, CD44, CLIC4, CLTC, CORO1C, EHD1, GNB2L1, HMGB1, ITGB1, MAPK1, MSN, MYH9, NPM1, PFN1, RAB7A, RHOA, SNX3, VIM	22
Cellular development, cellular growth and proliferation, connective tissue development and function, tissue development	Proliferation of fibroblasts	2.34 × 10^-5^	0.428	ANXA2, CAV1, CD44, DDX5, DNM1L, FN1, GPI, GSTP1, HDGF, HSPB1, ITGB1, LMNA, LMNB1, MAPK1, NPM1, PA2G4, PGK1, PRDX4, PRKDC, PTGES3, RBM3, RHOA, STAT1, VCL, YBX1	25
Protein trafficking	Targeting of protein	2.35 × 10^-5^		AIP, CALR, CSE1L, EIF5A, HSPA9, RAB7A, RAN, XPO1, YWHAB, YWHAE, YWHAG, YWHAQ, YWHAZ	13
Hematological system development and function, tissue development	Aggregation of blood cells	2.36 × 10^-5^	−1.292	ACTG1, AKR1B1, CAPN1, CAST, CD44, CLIC1, CSRP1, FLNA, GNAI2, HSPB1, ITGB1, LGALS1, MAPK1, MYH9, MYL12A, P4HB, PDIA3, TLN1, VCL	19
Cellular movement, connective tissue development and function	Cell movement of fibroblast Cell lines	2.39 × 10^-5^	−0.697	ACTR2, ACTR3, ARPC2, CD44, CLIC4, CTTN, DDX3X, EHD1, FN1, GPI, HMGB1, ITGB1, MAPRE1, NPM1, RHOA	15
Cellular development, tissue development	Differentiation of bone cells	2.43 × 10^-5^	−1.093	ALYREF, ATP5B, CAPNS1, CLIC1, CLTC, CYR61, DDX5, DHX9, FASN, FN1, GLO1, GNB2L1, H3F3A/H3F3B, HNRNPU, IARS, MAPK1, RPS11, RPS15, RRBP1, SND1, SQSTM1, STAT1, STMN1, SYNCRIP, TFRC, TNC, TPM4, VIM	28
Cancer, hematological disease, immunological disease, organismal injury and abnormalities	Lymphocytic cancer	2.48 × 10^-5^	1.450	ANXA1, ATIC, B2M, CD44, CSE1L, CYR61, FKBP1A, FN1, HIST1H1C, HMGB1, IQGAP1, LGALS1, LONP1, NNMT, NPM1, PRDX1, PRKDC, PSMB1, PSMD2, RHOA, RPS2, SF3B1, SHMT2, SPTBN1, STMN1, TUBB, TUBB4B, TUBB6, TUBB8, TXLNA, UQCRC1, XPO1, XRCC5, XRCC6, YWHAZ	35
Cancer, cellular development, cellular growth and proliferation, tumor morphology	Proliferation of cancer cells	2.63 × 10^-5^	−1.887	AHCY, AKR1B1, BSG, CACYBP, CAV1, CD44, CTSB, CYR61, EEF1A1, EZR, FASN, HDGF, HMGB1, LDHA, LGALS1, MAPK1, NPM1, PPP2CA, RHOA, RPS4X, S100A6, SQSTM1, STAT1, STMN1, TXLNA, XRCC5	26
Cell-to-cell signaling and interaction	Fusion of cells	2.74 × 10^-5^	1.580	ANXA1, ANXA5, CAPN2, CAV1, CD44, CTSB, FLNC, LGALS1, MAPK1, MYH9, RHOA, SOD1, STAT1	13
Cell morphology, cellular assembly and organization, cellular development, cellular function and maintenance, nervous system development and function, tissue development	Branching of neurons	2.75 × 10^-5^	−0.900	ACTR3, BASP1, CAPNS1, CAPZB, CAV1, CSRP1, CYR61, DBN1, DNM1L, DPYSL2, EZR, HMGB1, HNRNPK, ITGB1, MAP1B, NNMT, PDIA3, PFN1, RHOA, RTN4, SOD1	21
Cell morphology	Cell spreading of tumor Cell lines	2.96 × 10^-5^	−1.711	CAP1, CAPN2, FLNA, FLNB, FN1, ITGB1, MARCKS, PFN1, RHOA, TNC, UCHL1	11
Dermatological diseases and conditions, immunological disease, inflammatory disease	Lichen planus	2.96 × 10^-5^		B2M, CFL1, EEF1A1, EIF5A, ENO1, FKBP1A, HLA-A, HLA-B, IFITM3, PFN1, PSME2	11
Cellular assembly and organization, cellular function and maintenance	Formation of vesicles	3.07 × 10^-5^	0.707	ANXA2, ANXA5, ARF1, CAST, CLTC, FLNA, IQGAP1, MARCKS, PICALM, RAB11A, RHOA, SNX3	12
Immunological disease	Systemic autoimmune syndrome	3.10 × 10^-5^		ACLY, ACTA1, ALDOA, ANXA1, ARF1, ATIC, B2M, CALR, CD44, CTSA, CTSB, DDX39B, EEF1G, EEF2, ENAH, ENO1, FDPS, FKBP1A, H3F3A/H3F3B, HLA-A, HLA-B, HMGB1, HNRNPA1, HNRNPA3, HSP90B1, HSPA8, HSPD1, KHSRP, LDHB, LGALS1, MAPRE1, MYH10, MYH9, MYL12A, NONO, PCM1, PDIA3, PGK1, PGM1, PPP2CA, PRDX1, PRDX2, PRDX5, PTMA, RPS24, RPS3, RPSA, S100A6, SND1, STAT1, TFRC, TPM2, TRIM28, TUBB, UBE2L3, VARS, VIM	57
Cell morphology, cellular assembly and organization, cellular function and maintenance	Formation of filopodia	3.11 × 10^-5^	−2.546	ACTN4, ACTR3, CAPNS1, CSRP1, CYR61, EEF1A1, ENAH, EZR, FN1, IQGAP1, ITGB1, MAP1B, MARCKS, RHOA, TNC	15
Cell-to-cell signaling and interaction, cellular assembly and organization, cellular function and maintenance, tissue development	Formation of focal adhesions	3.12 × 10^-5^	−2.245	ACTN1, CAPN1, CAV1, CTTN, EHD1, EZR, FN1, GNAI2, GNB2L1, ITGB1, RHOA, STMN1, VCL, VIM	14
Cellular growth and proliferation, tissue development	Proliferation of connective tissue cells	3.14 × 10^-5^	0.548	ANXA2, ATIC, CAPNS1, CAV1, CD44, CLTC, CTSB, CYR61, DDX5, DNM1L, FN1, GNAI2, GPI, GSTP1, HDGF, HSPB1, ITGB1, KHDRBS1, LGALS1, LMNA, LMNB1, MAPK1, NPM1, PA2G4, PGK1, PRDX4, PRKDC, PTGES3, RBM3, RHOA, SERPINH1, SFN, STAT1, STMN1, TPM3, VCL, YBX1	37
Hematological disease, immunological disease	Eosinophilia	3.45 × 10^-5^		ALDOA, ENO1, GAPDH, HLA-A, HLA-B, HSPA5, MYH9, P4HB, PDIA3, PRDX1, TKT, TPI1, TUBB	13
Cellular assembly and organization, cellular function and maintenance	Organization of actin filaments	3.56 × 10^-5^	−1.633	ALDOA, CFL1, DBN1, DPYSL2, ENAH, FLNA, FN1, ITGB1, MSN, PLS3, RHOA	11
Cancer	Benign neoplasia	3.63 × 10^-5^	1.591	ACAT1, AIP, ALDOA, ALDOC, ANXA1, ANXA2, ANXA5, CALR, CD44, CTSB, CYR61, DBN1, DDX5, DDX6, FKBP1A, GSTP1, HINT1, HSD17B10, HSP90AB1, IFITM3, IQGAP1, KPNA3, LGALS1, MAPK1, MDH1, MYH10, NPEPPS, PARK7, PCNA, PEA15, PRDX1, PRDX2, PRDX5, RPL27, RPS15, SF3A1, SPTBN1, TNC, TWF1, VCP, ZYX	41
Cancer, gastrointestinal disease	Oral cancer	3.66 × 10^-5^		ANXA1, ATIC, BSG, EZR, FASN, FN1, H3F3A/H3F3B, HSP90AA1, HSP90AB1, HSP90B1, LGALS1, NPM1, PLOD2, RPL10, TAGLN, TNC, TUBB, TUBB4B	18
Cell death and survival	Cell death of lymphoma Cell lines	3.67 × 10^-5^	1.961	ANXA2, ARHGDIA, CD59, EZR, GLO1, HNRNPA1, HSPB1, LGALS1, LMNB1, LONP1, MAPK1, MSN, NCL, RAD23B, RPLP0, YWHAG, YWHAZ	17
Cell cycle, cell morphology, cellular assembly and organization, cellular movement	Elongation of actin filaments	3.68 × 10^-5^	−1.969	ACTN4, CAP1, FN1, PFN1	4
Infectious disease	Infection by Marburg virus	3.68 × 10^-5^	−0.000	HSPA5, RPL18, RPL3, RPL5	4
Carbohydrate metabolism, nucleic acid metabolism	Pentose shunt of monosaccharide	3.68 × 10^-5^		G6PD, PGD, TALDO1, TKT	4
Cancer, hematological disease, immunological disease, organismal injury and abnormalities	Non-Hodgkin’s disease	3.72 × 10^-5^		ANXA1, ATIC, B2M, CSE1L, CYR61, FKBP1A, FN1, HIST1H1C, HMGB1, IQGAP1, LONP1, NNMT, PRDX1, PRKDC, PSMB1, PSMD2, RHOA, RPS2, SF3B1, SHMT2, STMN1, TUBB, TUBB4B, TUBB6, TUBB8, TXLNA, UQCRC1, XPO1, XRCC5, YWHAZ	30
Organismal injury and abnormalities	Nodule	3.73 × 10^-5^		ANXA5, CALR, CTSB, GSTP1, HMGB1, HSP90AB1, PARK7, PRDX2, PRDX5	9
Cell death and survival	Cell death of cortical neurons	3.77 × 10^-5^	0.671	CAPN1, CAPNS1, CAST, CDK1, FUS, GAPDH, HMGB1, HSPA5, HSPB1, HSPD1, MAP1B, PARK7, PEA15, TCP1, YWHAB	15
Cell morphology, connective tissue development and function	Morphology of fibroblast cell lines	3.85 × 10^-5^		CAV1, CTTN, DPY30, FN1, KRT18, KRT8, MARCKS, PTBP1, RHOA, RTN4	10
Infectious disease	Replication of flaviviridae	3.85 × 10^-5^	−0.905	DDX3X, DDX6, DNAJA1, FASN, G3BP1, HMGB1, IFITM3, MVP, PPIA, YBX1	10
Cell death and survival	Cell viability of neuroblastoma cell lines	3.96 × 10^-5^	0.537	APEX1, HSP90AB1, HSPB1, P4HB, S100A6, SOD1, SQSTM1, VCP	8
Cellular compromise	Stress response of cells	4.00 × 10^-5^	0.174	CALR, CTSB, DNAJA1, HNRNPA1, HSD17B10, HSP90B1, HSPA5, PRDX1, SLC38A2, SOD1, SQSTM1, VCP, YBX1	13
Cancer, respiratory disease	Laryngeal tumor	4.00 × 10^-5^		ANXA1, CD44, HSP90AA1, HSP90AB1, HSP90B1, TUBB, TUBB4B	7
DNA replication, recombination, and repair	DNA damage	4.01 × 10^-5^	1.228	CALR, FN1, G6PD, GNAI2, GSTP1, HSPB1, LONP1, NPM1, PARK7, PDIA3, PRDX1, PRKDC, SOD1, SQSTM1, XRCC5, YWHAE	16
Cellular development, cellular growth and proliferation, nervous system development and function, tissue development	Proliferation of neuronal cells	4.31 × 10^-5^	−1.654	APEX1, B2M, BASP1, CAV1, CDK1, CFL1, CSRP1, CTNNA1, DNM1L, DPYSL2, EZR, FKBP4, FN1, HMGB1, IQGAP1, ITGB1, LGALS1, MAP1B, MAPK1, MARCKS, MYH10, MYH9, PDIA3, RAB11A, RHOA, RTN4, SEPT9, SLC25A5, SNX3, SOD1, SPTBN1, TNC, VAPA, VCL, VIM, YWHAZ	36
Cellular movement	Cell movement of hepatic stellate cells	4.34 × 10^-5^	−0.600	CD44, GNB2L1, HMGB1, LGALS1, MAPK1, MYH10	6
Cancer, respiratory disease	Laryngeal squamous Cell carcinoma	4.34 × 10^-5^		CD44, HSP90AA1, HSP90AB1, HSP90B1, TUBB, TUBB4B	6
Cellular movement	Migration of bone cancer cell lines	4.34 × 10^-5^	−1.091	ACTN4, CAPN2, CAV1, FN1, STMN1, VCP	6
Cell morphology	Shape change of epithelial cell lines	4.34 × 10^-5^	−1.000	CYR61, FLNA, FN1, ITGB1, MAPK1, VIM	6
Molecular transport, protein trafficking	Nuclear transport of protein	4.54 × 10^-5^		CALR, CSE1L, EIF5A, HSPA9, KPNB1, RAN, TNPO1, XPO1	8
Cancer	Growth of malignant tumor	4.65 × 10^-5^	−1.175	AHCY, AKR1B1, BSG, CACYBP, CAV1, CD44, CTSB, CYR61, EEF1A1, EZR, FASN, GNB2L1, HDGF, HMGB1, LDHA, LGALS1, MAPK1, NPM1, PKM, PPP2CA, RHOA, RPS4X, S100A6, SQSTM1, STAT1, STMN1, TNC, TXLNA, XRCC5	29
Cell morphology, cellular assembly and organization, cellular development, cellular function and maintenance, embryonic development, nervous system development and function, tissue development	Branching of neurites	4.69 × 10^-5^	−0.668	ACTR3, CAPNS1, CAPZB, CAV1, CSRP1, CYR61, DBN1, DNM1L, DPYSL2, EZR, HMGB1, HNRNPK, ITGB1, MAP1B, NNMT, PDIA3, PFN1, RHOA, RTN4, SOD1	20
Cell-to-cell signaling and interaction, cellular function and maintenance, inflammatory response	Phagocytosis of cells	4.69 × 10^-5^	−0.618	ANXA1, ANXA5, CALR, CAPG, CAV1, CD44, CLIC4, CLTC, CORO1C, EHD1, GNB2L1, HMGB1, ITGB1, MAPK1, MYH9, NPM1, PFN1, RHOA, SNX3, VIM	20
Cell-to-cell signaling and interaction	Binding of stem cells	5.32 × 10^-5^		C1QBP, ITGB1, TLN1	3
Tissue morphology	Collapse of epithelial tissue	5.32 × 10^-5^		KRT18, KRT8, RHOA	3
Cell-to-cell signaling and interaction, tissue development	Growth of focal adhesions	5.32 × 10^-5^		FLNA, FLNB, VIM	3
Molecular transport, protein trafficking	Import of green fluorescent protein	5.32 × 10^-5^		KPNA2, KPNB1, RAN	3
Cell-to-cell signaling and interaction, hair and skin development and function	Binding of epithelial cell lines	5.36 × 10^-5^	−0.294	ANXA2, ANXA5, CAV1, ITGB1, PPP2CA, PRMT5	6
Cancer, gastrointestinal disease, respiratory disease	Oropharyngeal tumor	5.36 × 10^-5^		HSP90AA1, HSP90AB1, HSP90B1, PCNA, TUBB, TUBB4B	6
Protein degradation, protein synthesis	Catabolism of protein	5.52 × 10^-5^	0.237	CALR, CANX, CAPN1, CAPN2, CAPNS1, CAST, CAV1, COPG1, CSTB, CTSB, FLNA, GAPDH, HSP90B1, HSPA5, HSPD1, IPO9, ITGB1, LONP1, MYH9, NACA, NPEPPS, PARK7, PDIA3, PPP2CA, PSMC4, PSMD2, SNX3, SOD1, SQSTM1, TPP1, UBE2L3, UBE2N, UBXN4, UCHL1, VCP, XPO1	36
Cell cycle	Arrest in cell cycle progression	5.60 × 10^-5^		C1QBP, CALR, CAV1, CD44, FASN, FKBP1A, KHDRBS1, LMNA, MAP4, MAPK1, NPM1, PA2G4, PCM1, PPM1G, PRMT5, RHOA, SFN, SSRP1, STAT1, TCP1, TNC, XRCC6, YWHAE	23
Infectious disease	Infection by flaviviridae	5.71 × 10^-5^	0.460	ACTR2, AP1B1, AP2B1, ARPC1B, CLTC, G3BP1, HMGB1, IFITM3, STAT1, TFRC	10
Cell cycle	G1 phase	5.92 × 10^-5^	−0.221	CAPNS1, CDK1, CYR61, FASN, FKBP1A, FN1, GNB2L1, GPI, ITGB1, LGALS1, LMNA, MAPK1, MCM7, NASP, PPP1CA, PRKDC, PTGES3, RHOA, RPL23, RPL5, RPL7A, SPTAN1, TFRC, TMPO, YWHAG, YWHAQ	26
Cancer, hematological disease, immunological disease, organismal injury and abnormalities	CD30-positive peripheral t-Cell lymphoma	6.02 × 10^-5^		TUBB, TUBB4B, TUBB6, TUBB8	4
Cardiovascular system development and function, organ morphology	Contraction of left ventricle	6.02 × 10^-5^	0.849	CAPNS1, ITGB1, LMNA, RHOA	4
Cellular assembly and organization, cellular function and maintenance	Formation of caveolae	6.02 × 10^-5^	−1.009	ANXA6, CAV1, PICALM, PTRF	4
Cancer, hematological disease, immunological disease, organismal injury and abnormalities	Systemic large-cell KI-1 lymphoma	6.02 × 10^-5^		TUBB, TUBB4B, TUBB6, TUBB8	4
Cancer, gastrointestinal disease	Stomach tumor	6.30 × 10^-5^		AKR1B1, ALYREF, ANXA1, CANX, CAV1, CD44, COPB2, CTNNA1, DDX39B, FKBP1A, FUS, GSTP1, HDGF, HINT1, HNRNPH1, IGF2R, IPO5, P4HB, PA2G4, RHOA, RPL22, TUBB4B, XRCC5	23
Cell morphology, cellular function and maintenance, DNA replication, recombination, and repair	Double-stranded DNA break repair of tumor cell lines	6.57 × 10^-5^	0.878	KPNA2, NPM1, OTUB1, UBE2N, XRCC5, XRCC6	6
Cancer	Head and neck cancer	6.57 × 10^-5^	1.756	ACTN1, ANXA1, ATIC, BSG, CAV1, CD44, CTTN, DDX3X, EZR, FASN, FLNA, FN1, GSTP1, H3F3A/H3F3B, HNRNPK, HSP90AA1, HSP90AB1, HSP90B1, HSPA5, LDHA, LGALS1, MARCKS, MCM7, NPM1, PCM1, PKM, PLOD2, PRDX1, PRKDC, PRMT5, RPL10, SET, SF3B1, SFN, SPTBN1, STAT1, TAGLN, TNC, TUBB, TUBB4B, VIM, XRCC5, XRCC6	43
Cellular assembly and organization, cellular function and maintenance	Formation of actin cytoskeleton	6.71E-05		CORO1C, ERP29, FLNA, FN1, ITGB1	5
Gene expression	Transcription	7.48 × 10^-5^	0.968	ACTR2, ACTR3, ALYREF, BASP1, BTF3, C14orf166, C1QBP, CALR, CAND1, CAV1, CBX3, CD44, CDK1, CSE1L, CYR61, DDX3X, DDX5, DHX9, EEF1D, EIF2S1, ENO1, FKBP1A, FLNA, FN1, FUBP3, GLO1, GSTP1, H2AFY, HDGF, HEXB, HINT1, HIST1H1C, HMGB1, HNRNPA1, HNRNPA2B1, HNRNPK, HSPA8, ILF2, ILF3, IQGAP1, KHDRBS1, KPNA2, LGALS1, LMNA, LRPPRC, MAPK1, MATR3, MCM7, NONO, NPM1, OTUB1, PA2G4, PARK7, PCNA, PDLIM1, PEA15, PEBP1, PFN1, PHB2, PHGDH, PICALM, PPP2CA, PRKDC, PRMT5, PTGES3, PTMA, PTRF, RHOA, RPL12, RPL6, SET, SFPQ, SQSTM1, SSRP1, STAT1, SUPT16H, TAGLN2, TMPO, TRIM28, UBE2L3, VAPA, XPO1, XRCC5, XRCC6, YBX1, YBX3, YWHAB, YWHAH, YWHAQ, YWHAZ	90
Cell-to-cell signaling and interaction, connective tissue development and function, tissue development	Adhesion of fibroblasts	7.57 × 10^-5^	−0.896	CD44, FN1, IGF2R, PLEC, TNC, VCL, ZYX	7
Organismal injury and abnormalities, skeletal and muscular disorders	Neurogenic muscular atrophy	7.57 × 10^-5^		AARS, DYNC1H1, GARS, HINT1, HSPB1, LMNA, RAB7A	7
DNA replication, recombination, and repair	DNA replication	7.63 × 10^-5^	−1.248	CACYBP, CALR, CAV1, CDK1, HMGB1, MAPK1, MCM7, NAP1L1, NASP, NCL, NPM1, PCM1, PCNA, PEA15, SET, SSRP1, SUPT16H, TMPO, XRCC5	19
Molecular transport	Nuclear export of molecule	8.10 × 10^-5^		ALYREF, CALR, CSE1L, DDX39B, EIF5A, HSPA9, KHDRBS1, RAN, XPO1	9
Organismal injury and abnormalities, renal and urological disease	Chronic kidney disease	8.13 × 10^-5^		CDV3, DBI, EEF1B2, FDPS, FKBP1A, H3F3A/H3F3B, HMGB1, HSP90AA1, IMPDH2, MYH9, NPM1, PSMB1, PSMD2, RPL23, RPL7, TUBB, TUBB4B	17
Cellular movement	Cell movement of melanoma cell lines	8.37 × 10^-5^	−0.364	ARF1, BSG, CAPN1, FLNA, ITGB1, KHDRBS1, KRT8, MSN, PEBP1, PRDX2, RHOA	11
Cell death and survival	Cytolysis	8.37 × 10^-5^	0.280	ALDOA, ANXA1, B2M, CALR, CAV1, CD44, CD59, G6PD, GPI, HLA-A, IMPDH2, KRT18, LGALS1, PRDX1, PRDX2, PSME2, PTMA, SH3BGRL3, STAT1, STMN1	20
Cell morphology, cellular function and maintenance	Transmembrane potential	8.37 × 10^-5^	−0.508	ANXA6, B2M, CLIC1, CLIC4, HSPA4, HSPB1, HSPD1, IMMT, KIF5B, LDHA, LGALS1, LONP1, PARK7, PHB2, SLC25A6, SOD1, STOML2, VCP, VDAC1, YWHAE	20
Protein synthesis	Initiation of translation of protein	8.60E-05		EIF2S1, EIF3B, EIF3C, EIF3I, EIF4G1, EIF4H, HSPB1, RPS3A	8
Hematological disease, infectious disease	Infection by Zaire ebolavirus	8.77 × 10^-5^	−2.216	CTSB, HSPA5, RPL18, RPL3, RPL5	5
Cellular assembly and organization	Formation of nucleus	8.78 × 10^-5^	−2.364	CDK1, FLNA, FN1, GNAI2, LMNA, LMNB1, RAN	7
Cellular assembly and organization, cellular function and maintenance, tissue development	Polymerization of microtubules	8.78 × 10^-5^	1.141	CAPZB, CAV1, FKBP4, MAP1B, MAPRE1, STMN1, TUBB	7
Inflammatory response	Immune response of cells	9.25 × 10^-5^	−1.018	ANXA1, ANXA5, CALR, CAPG, CAV1, CD44, CD59, CLIC4, CLTC, CORO1C, CTSB, DNM1L, EHD1, FN1, GNB2L1, HLA-A, HMGB1, HSP90AA1, HSPB1, ITGB1, LGALS1, MAPK1, MYH9, NPM1, OTUB1, PFN1, PSME2, RHOA, SF3A1, SNX3, STAT1, TNPO1, UBE2L3, VIM	34
Cell-to-cell signaling and interaction, tissue development	Adhesion of myeloma cell lines	9.28 × 10^-5^		ANXA2, CD44, FN1, ITGB1	4
Cell morphology, embryonic development	Cell spreading of embryonic cell lines	9.28 × 10^-5^	−1.067	FLNA, FN1, ITGB1, VIM	4
Gene expression	Replication of RNA	9.28 × 10^-5^	0.152	G3BP1, NPM1, VAPA, YBX1	4
Cardiovascular system development and function	Angiogenesis	9.48 × 10^-5^	−1.134	AKR1B1, ANXA2, ARHGDIA, ATP5B, CALR, CAPZB, CAV1, CD44, CLIC4, CTSB, CYR61, FKBP1A, FLNA, FLNB, FN1, G6PD, HADHA, HMGB1, HSP90B1, HSPB1, HSPD1, IGF2R, LDHA, LGALS1, MAPK1, MYH10, MYH9, NCL, PDCD6, PGK1, PKM, PLEC, RHOA, RNH1, RPSA, RTN4, SPTBN1, STAT1, TNC, VCL, VIM, WARS, YWHAE, YWHAZ	44
Cancer, organismal injury and abnormalities, reproductive system disease	Metastatic cervical cancer	9.62 × 10^-5^		ATIC, HSP90AA1, HSP90AB1, HSP90B1, TUBB, TUBB4B	6
Molecular transport, protein trafficking	Nuclear export of protein	9.62 × 10^-5^		CALR, CSE1L, EIF5A, HSPA9, RAN, XPO1	6
Cancer, organismal injury and abnormalities, reproductive system disease	Recurrent cervical cancer	9.62 × 10^-5^		ATIC, HSP90AA1, HSP90AB1, HSP90B1, TUBB, TUBB4B	6
Cell morphology, renal and urological system development and function	Shape change of kidney Cell lines	9.70 × 10^-5^	0.050	ANXA2, FLNA, FLNC, FN1, ITGB1, MAPK1, RHOA, VIM	8
Cellular development, cellular growth and proliferation	Proliferation of lung cancer cell lines	1.00 × 10^-4^	−1.422	ACLY, ANXA1, ANXA2, C1QBP, CAV1, CYR61, GAPDH, H2AFY, HNRNPA2B1, ITGB1, MAPK1, NASP, PKM, PTBP1, SET, SLC25A6, SOD1, STMN1, TRIM28	19
Cancer	Mucoepidermoid carcinoma	1.01 × 10^-4^		ATIC, FN1, HNRNPK, KRT18, SFN, SLC25A5, TNC	7
DNA replication, recombination, and repair	Degradation of DNA	1.03 × 10^-4^	−0.941	APEX1, BSG, CAPN2, CAST, DHX9, ENO1, HNRNPA1, HSD17B10, HSPB1, KRT8, LMNA, NPM1, PPIA, RHOA, SOD1, STAT1	16
Cellular development	Branching of cells	1.12 × 10^-4^	−1.379	ACTR3, ANXA2, BASP1, CAPNS1, CAPZB, CAV1, CSRP1, CYR61, DBN1, DNM1L, DPYSL2, EZR, FN1, HMGB1, HNRNPK, ITGB1, MAP1B, NNMT, PDIA3, PFN1, RHOA, RTN4, SOD1, TNC	24
Cell morphology, embryonic development	Shape change of embryonic cell lines	1.13 × 10^-4^	−0.555	FLNA, FN1, ITGB1, MAPK1, VIM	5
Cardiovascular system development and function, organismal development	Vasculogenesis	1.15 × 10^-4^	−1.808	AKR1B1, ANXA2, ARHGDIA, ATP5A1, CALR, CAPNS1, CAPZB, CAV1, CD44, CLIC4, CTSB, CYR61, FKBP1A, FLNA, FN1, G6PD, HADHA, HMGB1, HSP90B1, IGF2R, ITGB1, KRT1, LDHA, LGALS1, MAPK1, MYH10, PDCD6, PKM, PLEC, PPIA, PRKDC, RAD23B, RHOA, SEPT9, SERPINH1, SOD1, SPTBN1, STAT1, TNC, VCL, VIM, WARS, YWHAE, YWHAG, YWHAZ	45
Cancer, cellular movement, organismal injury and abnormalities, reproductive system disease, tumor morphology	Invasion of mammary tumor cells	1.17 × 10^-4^	0.156	CD44, CTTN, FLNA, FN1, HDLBP, ITGB1, RHOA	7
Cancer	Solid tumor	1.21 × 10^-4^	0.779	AARS, ACAT1, ACAT2, ACLY, ACTG1, ACTN1, ACTN4, ACTR2, ACTR3, AHCY, AHNAK, AIP, AKR1B1, ALDOA, ALDOC, ANXA1, ANXA2, ANXA5, AP1B1, APEX1, ARF1, ARHGDIA, ARPC2, ATIC, ATP5A1, ATP5B, B2M, BASP1, BSG, C14orf166, C1QBP, CACYBP, CALR, CAND1, CANX, CAP1, CAPG, CAPN1, CAPN2, CAPRIN1, CAPZA1, CAST, CAV1, CBX3, CCT2, CCT3, CCT4, CCT5, CCT6A, CCT7, CCT8, CD44, CD59, CDK1, CFL1, CLIC1, CLTC, CNN3, COPA, COPB1, COPB2, COPE, COPG1, CORO1C, COTL1, CRIP2, CSE1L, CTNNA1, CTPS1, CTSA, CTSB, CTTN, CUTA, CYB5R3, CYC1, CYR61, DBI, DBN1, DDX1, DDX39B, DDX3X, DDX5, DHX9, DLAT, DNAJA1, DNAJA2, DNAJC8, DNM1L, DPYSL2, DYNC1H1, DYNC1I2, ECHS1, EEF1A1, EEF1B2, EEF1D, EEF2, EHD1, EIF2S1, EIF2S2, EIF3B, EIF3C, EIF3I, EIF3M, EIF4A1, EIF4G1, EIF4H, ENAH, ENO1, ENO2, EPRS, ESYT1, ETFA, EZR, FASN, FDPS, FKBP1A, FLNA, FLNB, FLNC, FN1, FUBP3, FUS, G3BP1, G6PD, GANAB, GARS, GCN1L1, GDI1, GDI2, GLUD1, GML, GMPS, GNAI2, GNB2L1, GPI, GSTP1, H2AFY, H3F3A/H3F3B, HADHA, HARS, HDGF, HDLBP, HEXB, HINT1, HIST1H1C, HIST1H2BL, HIST2H2AC, HLA-A, HLA-B, HMGB1, HN1, HNRNPA1, HNRNPA2B1, HNRNPA3, HNRNPH1, HNRNPH3, HNRNPK, HNRNPL, HNRNPM, HNRNPR, HNRNPU, HSP90AA1, HSP90AB1, HSP90B1, HSPA4, HSPA5, HSPA8, HSPA9, HSPD1, HYOU1, IARS, IDI1, IFITM3, IGF2R, ILF2, ILF3, IMMT, IMPDH2, IPO5, IPO7, IPO9, IQGAP1, ITGB1, KHDRBS1, KHSRP, KIF5B, KPNA2, KPNA3, KPNB1, KRT1, KRT18, KRT8, KRT9, LAMB1, LASP1, LDHA, LGALS1, LMNA, LMNB1, LOC102724594/U2AF1, LONP1, LRPPRC, LRRC59, MAP1B, MAP4, MAPK1, MAPRE1, MARCKS, MARS, MATR3, MCM3, MCM4, MCM6, MCM7, MDH1, MDH2, MSN, MVP, MYH10, MYH9, MYL12A, NACA, NAP1L1, NAP1L4, NASP, NCL, NNMT, NONO, NPEPPS, NPLOC4, NPM1, NSFL1C, NUMA1, OLA1, P4HB, PA2G4, PABPC1, PAFAH1B2, PAICS, PARK7, PCBP1, PCBP2, PCM1, PCMT1, PCNA, PDCD6, PDE6H, PDIA3, PDIA4, PDLIM1, PDXK, PEA15, PFKP, PFN1, PGAM1, PGD, PGK1, PGM1, PHGDH, PICALM, PKM, PLEC, PLOD2, PLS3, PPIA, PPM1G, PPP1CA, PPP2CA, PPP2R1A, PRDX1, PRDX2, PRDX5, PRKCSH, PRKDC, PRMT5, PRPF19, PSMA1, PSMB1, PSMC1, PSMC4, PSMD2, PSME1, PTBP1, PTMA, PTRF, PUF60, PYGB, PYGL, QARS, RANBP1, RARS, RCN1, RHOA, RNH1, RPL10, RPL12, RPL21, RPL22, RPL27, RPL4, RPL5, RPL7, RPL7A, RPL8, RPLP0, RPN1, RPN2, RPS11, RPS12, RPS2, RPS24, RPS27A, RPS3A, RPS5, RPS7, RRBP1, RTN4, S100A6, SDHA, SEPT9, SERBP1, SERPINH1, SF3A1, SF3B1, SFN, SFPQ, SH3BGRL3	387
				SHMT2, SLC25A3, SLC25A5, SLC38A2, SND1, SOD1, SPTAN1, SPTBN1, SSRP1, STAT1, STIP1, STMN1, STOML2, SYNCRIP, TAGLN, TALDO1, TARS, TCP1, TFRC, TLN1, TMPO, TNC, TNPO1, TPI1, TPM2, TPM3, TPM4, TUBB, TUBB4B, TUBB6, TUBB8, TWF2, TXLNA, TXNDC5, UAP1, UBA1, UBE2N, UBXN4, UCHL1, UQCRC1, VARS, VAT1, VCL, VCP, VIM, VPS35, WARS, WDR1, XPO1, XRCC5, XRCC6, YBX1, YBX3, YWHAB, YWHAE, YWHAG, YWHAH, YWHAQ, YWHAZ, ZYX	
Cancer, cellular movement, tumor morphology	Invasion of tumor cells	1.29 × 10^-4^	−1.586	AHCY, CAPN2, CD44, CTSB, CTTN, EZR, FKBP1A, FLNA, FN1, HDLBP, ITGB1, LGALS1, MAPK1, PARK7, RHOA	15
Embryonic development, organ development, organismal development, skeletal and muscular system development and function, tissue development	Formation of muscle	1.29 × 10^-4^	−0.543	ACTA1, ACTG1, ACTN4, ANXA1, ANXA5, CALR, CAPN2, CAPZB, CAV1, FKBP1A, FLNB, FLNC, FN1, HMGB1, HSP90B1, ITGB1, KRT8, MAPK1, MYH10, MYH9, PLEC, RHOA, STIP1, UCHL1, VCL, VIM	26
Cell-to-cell signaling and interaction, embryonic development	Binding of embryonic cells	1.31 × 10^-4^		C1QBP, ITGB1, TLN1	3
Cell death and survival, connective tissue disorders, developmental disorder, hematological disease, hereditary disorder	Hereditary nonspherocytic hemolytic anemia	1.31 × 10^-4^		ALDOA, G6PD, GPI	3
Cell morphology, endocrine system development and function, organ morphology, organismal development	Morphology of thyroid cells	1.31 × 10^-4^		CAV1, CTSB, FN1	3
Cellular assembly and organization, cellular function and maintenance	Quantity of filopodia-like projection	1.31 × 10^-4^		ACTR2, ARPC2, CAPZB	3
Amino acid metabolism, small molecule biochemistry	Synthesis of l-serine	1.31 × 10^-4^		PHGDH, PKM, SHMT2	3
Cellular assembly and organization, cellular function and maintenance, tissue development	Formation of actin filaments	1.34 × 10^-4^	−1.689	ACTR3, ARF1, ARPC2, CAPN1, CAV1, CD44, CFL1, CTTN, FN1, GNAI2, GPI, ITGB1, MAPK1, NPM1, PFN1, RHOA, TNC, TPM2, TWF1, TWF2, ZYX	21
Cell cycle, cellular movement	Cytokinesis of cervical cancer cell lines	1.34 × 10^-4^	0.218	GNAI2, LMNA, NPM1, RHOA, SEPT7, SEPT9, SSRP1	7
Cancer, respiratory disease	Metastatic non-small-cell lung cancer	1.34 × 10^-4^		ATIC, EIF4A1, HSP90AA1, HSP90AB1, HSP90B1, TUBB, TUBB4B	7
Cell-to-cell signaling and interaction, tissue development	Adhesion of hepatoma cell lines	1.37 × 10^-4^		BSG, C1QBP, ITGB1, RHOA	4
Cardiovascular system development and function, cell morphology, cell-to-cell signaling and interaction, organ morphology, organismal development, skeletal and muscular system development and function, tissue morphology	Morphology of intercalated disks	1.37 × 10^-4^		CALR, CAPZB, PLEC, VCL	4
Cellular compromise	Stress response of cervical cancer cell lines	1.37 × 10^-4^		CALR, HNRNPA1, HSP90B1, HSPA5	4
Cancer	Metastatic occult primary cancer of head and neck	1.43 × 10^-4^		HSP90AA1, HSP90AB1, HSP90B1, TUBB, TUBB4B	5
Cancer, connective tissue disorders, respiratory disease, skeletal and muscular disorders	Metastatic squamous cell cancer of the ethmoid sinus	1.43 × 10^-4^		HSP90AA1, HSP90AB1, HSP90B1, TUBB, TUBB4B	5
Cancer, respiratory disease	Metastatic squamous cell cancer of the glottis	1.43 × 10^-4^		HSP90AA1, HSP90AB1, HSP90B1, TUBB, TUBB4B	5
Cancer, gastrointestinal disease	Metastatic squamous cell cancer of the lip	1.43 × 10^-4^		HSP90AA1, HSP90AB1, HSP90B1, TUBB, TUBB4B	5
Cancer, respiratory disease	Metastatic squamous cell cancer of the maxillary sinus	1.43 × 10^-4^		HSP90AA1, HSP90AB1, HSP90B1, TUBB, TUBB4B	5
Cancer, gastrointestinal disease, respiratory disease	Metastatic squamous cell cancer of the oropharynx	1.43 × 10^-4^		HSP90AA1, HSP90AB1, HSP90B1, TUBB, TUBB4B	5
Cancer, respiratory disease	Metastatic squamous cell cancer of the supraglottis	1.43 × 10^-4^		HSP90AA1, HSP90AB1, HSP90B1, TUBB, TUBB4B	5
Cellular movement, connective tissue development and function, hepatic system development and function	Migration of hepatic stellate cells	1.43 × 10^-4^	−0.218	CD44, GNB2L1, HMGB1, LGALS1, MYH10	5
Cancer	Recurrent occult primary cancer of head and neck	1.43 × 10^-4^		HSP90AA1, HSP90AB1, HSP90B1, TUBB, TUBB4B	5
Cancer, connective tissue disorders, respiratory disease, skeletal and muscular disorders	Recurrent squamous cell cancer of the ethmoid sinus	1.43 × 10^-4^		HSP90AA1, HSP90AB1, HSP90B1, TUBB, TUBB4B	5
Cancer, respiratory disease	Recurrent squamous cell cancer of the glottis	1.43 × 10^-4^		HSP90AA1, HSP90AB1, HSP90B1, TUBB, TUBB4B	5
Cancer, gastrointestinal disease	Recurrent squamous cell cancer of the lip	1.43 × 10^-4^		HSP90AA1, HSP90AB1, HSP90B1, TUBB, TUBB4B	5
Cancer, respiratory disease	Recurrent squamous cell cancer of the maxillary sinus	1.43 × 10^-4^		HSP90AA1, HSP90AB1, HSP90B1, TUBB, TUBB4B	5
Cancer, respiratory disease	Recurrent squamous cell cancer of the supraglottis	1.43 × 10^-4^		HSP90AA1, HSP90AB1, HSP90B1, TUBB, TUBB4B	5
Cancer	Unresectable occult primary cancer of head and neck	1.43 × 10^-4^		HSP90AA1, HSP90AB1, HSP90B1, TUBB, TUBB4B	5
Cancer, connective tissue disorders, respiratory disease, skeletal and muscular disorders	Unresectable squamous Cell cancer of the ethmoid sinus	1.43 × 10^-4^		HSP90AA1, HSP90AB1, HSP90B1, TUBB, TUBB4B	5
Cancer, respiratory disease	Unresectable squamous Cell cancer of the glottis	1.43 × 10^-4^		HSP90AA1, HSP90AB1, HSP90B1, TUBB, TUBB4B	5
Cancer, gastrointestinal disease, respiratory disease	Unresectable squamous Cell cancer of the hypopharynx	1.43 × 10^-4^		HSP90AA1, HSP90AB1, HSP90B1, TUBB, TUBB4B	5
Cancer, gastrointestinal disease	Unresectable squamous Cell cancer of the lip	1.43 × 10^-4^		HSP90AA1, HSP90AB1, HSP90B1, TUBB, TUBB4B	5
Cancer, respiratory disease	Unresectable squamous Cell cancer of the maxillary sinus	1.43 × 10^-4^		HSP90AA1, HSP90AB1, HSP90B1, TUBB, TUBB4B	5
Cancer, gastrointestinal disease, respiratory disease	Unresectable squamous Cell cancer of the oropharynx	1.43 × 10^-4^		HSP90AA1, HSP90AB1, HSP90B1, TUBB, TUBB4B	5
Cancer, respiratory disease	Unresectable squamous Cell cancer of the supraglottis	1.43 × 10^-4^		HSP90AA1, HSP90AB1, HSP90B1, TUBB, TUBB4B	5
Cellular movement	Cell movement of carcinoma Cell lines	1.48 × 10^-4^	0.447	C1QBP, CAV1, CD44, CTTN, CYR61, HNRNPA2B1, ITGB1, LGALS1, STMN1, TAGLN2, VIM, ZYX	12
Cellular development, cellular growth and proliferation	Proliferation of colon cancer cell lines	1.48 × 10^-4^	1.556	AHSA1, CACYBP, CALR, CDK1, CSE1L, DDX5, EIF3C, FN1, HSP90AA1, IGF2R, IPO7, NCL, PKM, RHOA, SFN, SFPQ, UBA1, XRCC5	18
Cardiovascular disease, organismal injury and abnormalities	Dilated cardiomyopathy	1.50 × 10^-4^	−0.192	CAST, DNM1L, FKBP1A, LMNA, MYH9, PGK1, PPP1CA, SDHA, TLN1, TMPO, TPI1, VCL, VDAC1, VDAC2	14
Cardiovascular system development and function, organ morphology, organismal development, skeletal and muscular system development and function, tissue morphology	Morphology of cardiac muscle	1.50 × 10^-4^		CALR, CAPZB, CAV1, FKBP1A, FN1, HADHA, HSP90B1, IGF2R, MAPK1, MYH10, PLEC, SPTBN1, VCL, YWHAE	14
Infectious disease	Infection by dengue virus 2	1.53 × 10^-4^	0.896	ACTR2, AP1B1, AP2B1, ARPC1B, CLTC, IFITM3, STAT1	7
Cell morphology, cellular assembly and organization, cellular function and maintenance	Reorganization of cytoskeleton	1.59 × 10^-4^	−1.953	ARHGDIA, CD44, CFL1, DPYSL2, EZR, FLNA, FLNC, FN1, HMGB1, MARCKS, MSN, MYH9, PLS3, RHOA, SPTAN1	15
Cell-to-cell signaling and interaction, tissue development	Cell-cell adhesion of tumor Cell lines	1.62 × 10^-4^	0.128	C1QBP, ERP29, GNB2L1, ITGB1, MAPK1, ZYX	6
Cellular assembly and organization	Formation of cytoplasmic aggregates	1.62 × 10^-4^	−1.131	CAV1, DDX6, LMNA, RHOA, SOD1, YWHAZ	6
Tissue development	Aggregation of cells	1.65 × 10^-4^	−0.939	ACTG1, AKR1B1, BSG, CAPN1, CAST, CD44, CLIC1, CSRP1, FLNA, FN1, GNAI2, HNRNPA2B1, HSPB1, ITGB1, LGALS1, MAPK1, MYH9, MYL12A, P4HB, PDIA3, TLN1, VCL	22
Cell death and survival	Apoptosis of fibroblast Cell lines	1.65 × 10^-4^	−0.651	CAPNS1, CDK1, CLIC4, CYR61, DDX3X, DNM1L, EEF1A1, EIF2S1, EIF3B, EIF3C, EIF3I, EIF6, FN1, FUS, GPI, HSPA5, HSPD1, ITGB1, RHOA, RPL10, STAT1, VIM	22
Infectious disease, reproductive system disease	Infection of cervical cancer cell lines	1.69 × 10^-4^	−2.295	ARF1, ATP5B, CCT2, COPA, COPB1, COPB2, COPG1, DDX3X, DNAJA2, EIF3I, GML, H3F3A/H3F3B, HNRNPK, HNRNPU, HSPA9, MAP4, PDIA3, PDIA6, PSME2, RAB1B, RANBP1, SPTAN1, SPTBN1, STIP1, TWF1, UAP1, UQCRC1, XPO1	28
Cell cycle	Mitosis	1.76 × 10^-4^	−1.833	BUB3, CAPN2, CCT4, CDK1, CLTC, CSE1L, CYR61, DBI, DHX9, DYNC1H1, EIF6, KPNB1, KRT18, MAPK1, MARCKS, MYH10, NUMA1, PEBP1, PPP1CA, PRMT5, PTMA, RAN, RPS24, SEPT9, STAT1, STMN1, TCP1, TNC, TUBB, YWHAE	30
Cellular movement	Migration of tumor cells	1.76 × 10^-4^	−0.843	ACTN4, AHCY, CAV1, CD44, CDK1, CSE1L, CTSB, CTTN, EZR, FN1, HMGB1, IQGAP1, ITGB1, RHOA, S100A6, TNC, VIM	17
Cell morphology, cellular function and maintenance	Autophagy of cells	1.77 × 10^-4^	0.609	CAPN1, CAPNS1, CAST, DNM1L, EIF2S1, EIF4G1, FKBP1A, HMGB1, HSPA5, KRT18, PAFAH1B2, PRKDC, RAB7A, SERPINH1, SOD1, SQSTM1, STAT1, TUFM	18
Cardiovascular system development and function, organismal development	Development of blood vessel	1.78 × 10^-4^	−1.788	AKR1B1, ANXA2, ARHGDIA, ATP5A1, CALR, CAPNS1, CAPZB, CAV1, CD44, CLIC4, CTSB, CYR61, FKBP1A, FLNA, FN1, G6PD, HADHA, HMGB1, HSP90B1, HSPB1, IGF2R, ITGB1, KRT1, LDHA, LGALS1, MAPK1, MYH10, PDCD6, PGK1, PKM, PLEC, PPIA, PRKDC, RAD23B, RHOA, RNH1, SEPT9, SERPINH1, SOD1, SPTBN1, STAT1, TNC, VCL, VIM, WARS, YWHAE, YWHAG, YWHAZ	48
Cardiovascular system development and function, cell morphology	Cell spreading of endothelial cells	1.79 × 10^-4^	−1.000	FLNA, FN1, ITGB1, VIM, YWHAZ	5
Cell morphology, renal and urological system development and function	Cell spreading of kidney Cell lines	1.79 × 10^-4^	−0.678	FLNA, FLNC, FN1, ITGB1, VIM	5
Cell death and survival, DNA replication, recombination, and repair	Fragmentation of DNA	1.88 × 10^-4^	−0.910	APEX1, BSG, CAPN2, CAST, ENO1, HSD17B10, HSPB1, KRT8, LMNA, NPM1, PPIA, RHOA, SOD1, STAT1	14
Cancer, gastrointestinal disease, respiratory disease	Nasopharyngeal cancer	1.91 × 10^-4^		HSP90AA1, HSP90AB1, HSP90B1, SFN, TUBB, TUBB4B	6
Energy production, nucleic acid metabolism, small molecule biochemistry	Binding of ATP	1.94 × 10^-4^	−0.068	MAPK1, PCNA, PTGES3, STIP1	4
Free radical scavenging	Modification of hydrogen peroxide	1.94 × 10^-4^	−1.710	PRDX1, PRDX2, PRDX5, SOD1	4
Cell-to-cell signaling and interaction, cellular assembly and organization, tissue development	Turnover of focal adhesions	1.94 × 10^-4^	−0.254	CAV1, ITGB1, PLEC, VCL	4
Cancer, cell death and survival, tumor morphology	Cell death of tumor cells	1.95 × 10^-4^	0.142	ACLY, ANXA1, ANXA2, B2M, BSG, CAV1, CD44, CD59, CDK1, CTSB, CYR61, EIF3C, ENO1, FASN, GNB2L1, HMGB1, HSP90AB1, LGALS1, MAPK1, PPP2CA, RHOA, RPS3A, RTN4, SOD1	24
Cellular function and maintenance, tissue development	Organization of muscle cells	1.98 × 10^-4^		ACTG1, CALR, CAV1, CTNNA1, FN1, ITGB1, KRT8	7
RNA post-transcriptional modification	Processing of rRNA	1.98 × 10^-4^		DDX5, NPM1, RPL5, RPL7, RPS15, RPS24, RPS7	7
Cancer, gastrointestinal disease	Oral cavity carcinoma	2.06 × 10^-4^		ATIC, EZR, FASN, FN1, HSP90AA1, HSP90AB1, HSP90B1, LGALS1, NPM1, PLOD2, RPL10, TAGLN, TNC, TUBB, TUBB4B	15
Inflammatory response	Inflammation of body cavity	2.07 × 10^-4^	0.920	ACTN4, ALDOA, ANXA1, ANXA5, ARHGDIA, B2M, BSG, CAV1, CD44, CYR61, DDX5, ENO1, FKBP1A, GAPDH, GSTP1, HLA-A, HMGB1, HSPA5, IMPDH2, KRT18, KRT8, LGALS1, MYH10, MYH9, P4HB, PDIA3, PDLIM1, PPIA, PPP2CA, PRDX1, PSMB1, PSMD2, SOD1, STAT1, TKT, TPI1, TUBB, YBX1	38
Cancer	Malignant solid tumor	2.08 × 10^-4^	0.397	AARS, ACAT1, ACAT2, ACLY, ACTG1, ACTN1, ACTN4, ACTR2, ACTR3, AHCY, AHNAK, AKR1B1, ALDOA, ALDOC, ANXA1, ANXA2, ANXA5, AP1B1, APEX1, ARF1, ARHGDIA, ARPC2, ATIC, ATP5A1, ATP5B, B2M, BASP1, BSG, C14orf166, C1QBP, CACYBP, CAND1, CANX, CAP1, CAPG, CAPN1, CAPN2, CAPRIN1, CAPZA1, CAST, CAV1, CBX3, CCT2, CCT3, CCT4, CCT5, CCT6A, CCT7, CCT8, CD44, CD59, CDK1, CFL1, CLIC1, CLTC, CNN3, COPA, COPB1, COPB2, COPE, COPG1, CORO1C, COTL1, CRIP2, CSE1L, CTNNA1, CTPS1, CTSA, CTSB, CTTN, CUTA, CYB5R3, CYC1, CYR61, DBI, DBN1, DDX1, DDX39B, DDX3X, DDX5, DHX9, DLAT, DNAJA1, DNAJA2, DNAJC8, DNM1L, DPYSL2, DYNC1H1, DYNC1I2, ECHS1, EEF1A1, EEF1B2, EEF1D, EEF2, EHD1, EIF2S1, EIF2S2, EIF3B, EIF3C, EIF3I, EIF3M, EIF4A1, EIF4G1, EIF4H, ENAH, ENO1, ENO2, EPRS, ESYT1, ETFA, EZR, FASN, FDPS, FKBP1A, FLNA, FLNB, FLNC, FN1, FUBP3, FUS, G3BP1, G6PD, GANAB, GARS, GCN1L1, GDI1, GDI2, GLUD1, GML, GMPS, GNAI2, GNB2L1, GPI, GSTP1, H2AFY, H3F3A/H3F3B, HADHA, HARS, HDGF, HDLBP, HEXB, HINT1, HIST1H1C	383
				HIST1H2BL, HIST2H2AC, HLA-A, HLA-B, HMGB1, HN1, HNRNPA1, HNRNPA2B1, HNRNPA3, HNRNPH1, HNRNPH3, HNRNPK, HNRNPL, HNRNPM, HNRNPR, HNRNPU, HSP90AA1, HSP90AB1, HSP90B1, HSPA4, HSPA5, HSPA8, HSPA9, HSPD1, HYOU1, IARS, IDI1, IFITM3, IGF2R, ILF2, ILF3, IMMT, IMPDH2, IPO5, IPO7, IPO9, IQGAP1, ITGB1, KHDRBS1, KHSRP, KIF5B, KPNA2, KPNA3, KPNB1, KRT1, KRT18, KRT8, KRT9, LAMB1, LASP1, LDHA, LGALS1, LMNA, LMNB1, LOC102724594/U2AF1, LONP1, LRPPRC, LRRC59, MAP1B, MAP4, MAPK1, MAPRE1, MARCKS, MARS, MATR3, MCM3, MCM4, MCM6, MCM7, MDH1, MDH2, MSN, MVP, MYH10, MYH9, MYL12A, NACA, NAP1L1, NAP1L4, NASP, NCL, NNMT, NONO, NPEPPS, NPLOC4, NPM1, NSFL1C, NUMA1, OLA1, P4HB, PA2G4, PABPC1, PAFAH1B2, PAICS, PCBP1, PCBP2, PCM1, PCMT1, PCNA, PDCD6, PDE6H, PDIA3, PDIA4, PDLIM1, PDXK, PEA15, PFKP, PFN1, PGAM1, PGD, PGK1, PGM1, PHGDH, PICALM, PKM, PLEC, PLOD2, PLS3, PPIA, PPM1G, PPP1CA, PPP2CA, PPP2R1A, PRDX1, PRDX2, PRDX5, PRKCSH, PRKDC, PRMT5, PRPF19, PSMA1, PSMB1, PSMC1, PSMC4, PSMD2, PSME1, PTBP1, PTMA, PTRF, PUF60, PYGB, PYGL, QARS, RANBP1, RARS, RCN1, RHOA, RNH1, RPL10, RPL12, RPL22, RPL27, RPL4, RPL5, RPL7, RPL7A, RPL8, RPLP0, RPN1, RPN2, RPS11, RPS12, RPS2, RPS24, RPS27A, RPS3A, RPS5, RPS7, RRBP1, RTN4, S100A6, SDHA, SEPT9, SERBP1, SERPINH1, SF3A1, SF3B1, SFN, SFPQ, SH3BGRL3, SHMT2, SLC25A3, SLC25A5, SLC38A2, SND1, SOD1, SPTAN1, SPTBN1, SSRP1, STAT1, STIP1, STMN1, STOML2, SYNCRIP, TAGLN, TALDO1, TARS, TCP1, TFRC, TLN1, TMPO, TNC, TNPO1, TPI1, TPM2, TPM3, TPM4, TUBB, TUBB4B, TUBB6, TUBB8, TWF2, TXLNA, TXNDC5, UAP1, UBA1, UBE2N, UBXN4, UCHL1, UQCRC1, VARS, VAT1, VCL, VCP, VIM, VPS35, WARS, WDR1, XPO1, XRCC5, XRCC6, YBX1, YBX3, YWHAB, YWHAE, YWHAG, YWHAH, YWHAQ, YWHAZ, ZYX	
Cell cycle	M phase of cervical cancer Cell lines	2.10 × 10^-4^	0.218	GNAI2, LMNA, MCM7, NPM1, RHOA, SEPT7, SEPT9, SSRP1	8
Cardiovascular system development and function, organismal development, tissue morphology	Morphology of cardiovascular tissue	2.11 × 10^-4^		ARHGDIA, CALR, CAPZB, CAV1, CD44, CLIC4, FKBP1A, FN1, HADHA, HSP90B1, IGF2R, MAPK1, MYH10, PLEC, SPTBN1, VCL, YWHAE	17
Cell cycle	Interphase of tumor cell lines	2.11 × 10^-4^	1.067	ANXA2, CDK1, CSE1L, CYR61, FASN, ITGB1, LGALS1, MCM7, NASP, NPM1, PDCD6, PTGES3, RHOA, RPL23, RPL5, RPL7A, SFN, SPTAN1, STAT1, TCP1, TFRC, YWHAG, YWHAQ	23
Infectious disease	Replication of retroviridae	2.19 × 10^-4^	0.989	ANXA5, BTF3, CAV1, DDX5, DHX9, IFITM3, PLIN3, PPIA, PRDX1, PRDX2, RAB11A, TMPO	12
Cell morphology	Sprouting	2.22 × 10^-4^	−1.379	ACTR3, ANXA2, BASP1, CAPNS1, CAPZB, CAV1, CSRP1, CYR61, DBN1, DNM1L, DPYSL2, EZR, FN1, HMGB1, HNRNPK, ITGB1, MAP1B, NNMT, PDIA3, PFN1, RHOA, RTN4, SOD1, TNC	24
Connective tissue disorders, developmental disorder, hereditary disorder	Marfan’s syndrome	2.22 × 10^-4^		ALDOC, FLNA, MYH10, TAGLN, VCL	5
Cancer, gastrointestinal disease, respiratory disease	Metastatic squamous cell cancer of the hypopharynx	2.22 × 10^-4^		HSP90AA1, HSP90AB1, HSP90B1, TUBB, TUBB4B	5
Cellular function and maintenance	Pinocytosis	2.22 × 10^-4^	−1.000	ACTN4, CAV1, EZR, NCL, RHOA	5
Cancer, gastrointestinal disease, respiratory disease	Recurrent squamous cell cancer of the hypopharynx	2.22 × 10^-4^		HSP90AA1, HSP90AB1, HSP90B1, TUBB, TUBB4B	5
Cancer, gastrointestinal disease, respiratory disease	Recurrent squamous cell cancer of the oropharynx	2.22 × 10^-4^		HSP90AA1, HSP90AB1, HSP90B1, TUBB, TUBB4B	5
Embryonic development, tissue morphology	Morphology of visceral endoderm	2.23 × 10^-4^		DHX9, ITGB1, MAPK1, MYH9, RAB7A, SERPINH1	6
Cellular movement	Cell movement of lung cancer cell lines	2.26 × 10^-4^	0.276	C1QBP, CAV1, CD44, FN1, HNRNPA2B1, ITGB1, STMN1, VIM, YBX1, ZYX	10
Cellular movement	Migration of cancer cells	2.28 × 10^-4^	−0.743	AHCY, CAV1, CD44, CDK1, CSE1L, CTSB, EZR, FN1, HMGB1, IQGAP1, ITGB1, RHOA, S100A6, TNC, VIM	15
Cell cycle	Arrest in interphase of tumor cell lines	2.29 × 10^-4^		ANXA2, CDK1, CSE1L, CYR61, FASN, ITGB1, LGALS1, PDCD6, PTGES3, RHOA, RPL23, RPL5, RPL7A, SFN, SPTAN1, TCP1, TFRC, YWHAG	18
Cellular function and maintenance	Engulfment of tumor cell lines	2.48 × 10^-4^	0.222	ANXA1, CAV1, CD44, CLTC, EZR, GNB2L1, ITGB1, NCL, PFN1, RHOA, SFPQ, VIM	12
Cell-to-cell signaling and interaction, tissue development	Detachment of cells	2.52 × 10^-4^	0.851	ANXA1, ANXA5, CAPN2, FLNA, FN1, ITGB1, MAPK1, RHOA, TLN1	9
Cell morphology, cell-to-Cell signaling and interaction	Morphology of intercellular junctions	2.52 × 10^-4^		B2M, CALR, CAPRIN1, CAPZB, CAV1, LGALS1, PLEC, VCL, YWHAG	9
Gene expression, protein synthesis	Initiation of translation of mRNA	2.53 × 10^-4^		EIF3B, EIF3C, EIF3I, EIF4G1, EIF4H, HSPB1, RPS3A	7
Cell-to-cell signaling and interaction, connective tissue development and function, tissue development	Attachment of fibroblast Cell lines	2.57 × 10^-4^		FN1, IPO9, ITGB1	3
Cell morphology	Cell spreading of fibrosarcoma cell lines	2.57 × 10^-4^		FLNA, FLNB, ITGB1	3
Organ morphology, skeletal and muscular system development and function	Contraction of airway smooth muscle	2.57 × 10^-4^		CAV1, GNAI2, RHOA	3
Cellular assembly and organization, DNA replication, recombination, and repair	Formation of nuclear envelope	2.57 × 10^-4^		CDK1, LMNA, RAN	3
Carbohydrate metabolism	Metabolism of fructose-1, 6-diphosphate	2.57 × 10^-4^		ALDOA, ALDOC, PFKP	3
Cancer, endocrine system disorders, organismal injury and abnormalities, reproductive system disease	Metastatic ovarian tumor	2.57 × 10^-4^		HSP90AA1, HSP90AB1, HSP90B1	3
Cellular development, cellular growth and proliferation	Outgrowth of breast cancer Cell lines	2.57 × 10^-4^		CYR61, FN1, ITGB1	3
Cellular assembly and organization, cellular function and maintenance, protein trafficking	Sequestration of g-actin	2.57 × 10^-4^		PFN1, TWF1, TWF2	3
Cell cycle	Abnormal cell cycle	2.58 × 10^-4^		CAV1, H3F3A/H3F3B, HSPB1, PRDX1, RBM3, STAT1, TMPO, YWHAE	8
Cellular movement, connective tissue development and function	Migration of fibroblasts	2.64 × 10^-4^	0.975	CAPNS1, CAV1, CYR61, EHD1, FLNB, FN1, MAPK1, MYH10, PLEC, STMN1, VCL, ZYX	12
Developmental disorder, hematological disease, hereditary disorder, organismal injury and abnormalities	Diamond-blackfan anemia	2.66 × 10^-4^		RPL5, RPS10, RPS24, RPS7	4
Organismal injury and abnormalities, renal and urological disease	Minimal change nephrotic syndrome	2.66 × 10^-4^		ACTN4, FKBP1A, IMPDH2, PDLIM1	4
Cancer, organismal injury and abnormalities, renal and urological disease	Transitional cell bladder cancer	2.66 × 10^-4^		GSTP1, HSP90AA1, HSP90AB1, HSP90B1	4
Cellular assembly and organization, cellular function and maintenance	Growth of microtubules	2.72 × 10^-4^	−1.710	MAP4, MAPRE1, MYH9, SSRP1, STMN1	5
Cell death and survival	Apoptosis of cerebral cortex cells	2.84 × 10^-4^	0.050	CAPN1, CAST, CDK1, HSPA5, MAP1B, P4HB, PEA15, RPS3, SOD1, YWHAB	10
Cell death and survival, renal and urological system development and function	Cell viability of kidney cell lines	2.85 × 10^-4^	−0.378	ANXA5, CAV1, EZR, HNRNPU, HSP90B1, HYOU1, PPIA, VDAC1	8
Cell-to-cell signaling and interaction, renal and urological system development and function	Binding of kidney cell lines	2.85 × 10^-4^	0.055	ANXA2, BSG, CAV1, ITGB1, PPP2CA, PRMT5, RPSA	7
Molecular transport, RNA trafficking	Transport of mRNA	2.85 × 10^-4^		ALYREF, DDX39B, EIF5A, FUS, KHDRBS1, U2AF2, XPO1	7
Gene expression	Transcription of RNA	2.90 × 10^-4^	1.084	ACTR2, ACTR3, ALYREF, BASP1, BTF3, C14orf166, C1QBP, CALR, CAND1, CAV1, CBX3, CD44, CDK1, CSE1L, CYR61, DDX3X, DDX5, EEF1D, EIF2S1, ENO1, FKBP1A, FLNA, FN1, FUBP3, GLO1, GSTP1, H2AFY, HDGF, HEXB, HINT1, HIST1H1C, HMGB1, HNRNPA1, HNRNPA2B1, HNRNPK, HSPA8, ILF2, ILF3, IQGAP1, KHDRBS1, KPNA2, LGALS1, LMNA, LRPPRC, MAPK1, MATR3, MCM7, NONO, NPM1, OTUB1, PA2G4, PARK7, PCNA, PDLIM1, PEA15, PEBP1, PFN1, PHB2, PHGDH, PICALM, PPP2CA, PRKDC, PRMT5, PTGES3, PTMA, PTRF, RHOA, RPL6, SET, SFPQ, SQSTM1, SSRP1, STAT1, TMPO, TRIM28, UBE2L3, VAPA, XPO1, XRCC5, XRCC6, YBX1, YBX3, YWHAB, YWHAH, YWHAQ, YWHAZ	86
Cancer, endocrine system disorders, respiratory disease	Small cell lung cancer	2.99 × 10^-4^		ENO2, HSP90AA1, HSP90AB1, HSP90B1, HSPD1, PDCD6, TUBB, TUBB4B, YWHAE	9
Cellular development, cellular growth and proliferation, embryonic development, organ development, organismal development, skeletal and muscular system development and function, tissue development	Formation of muscle cells	3.04 × 10^-4^	−1.408	ACTA1, ACTG1, ACTN4, CALR, CAPZB, FLNC, FN1, HMGB1, HSP90B1, ITGB1, KRT8, MYH10, PLEC, UCHL1	14
Cellular assembly and organization	Quantity of actin filaments	3.14 × 10^-4^	−0.570	CFL1, FN1, GNB2L1, MARCKS, PLEC, RHOA, SPTAN1, TMOD3	8
DNA replication, recombination, and repair	Repair of DNA	3.18 × 10^-4^	−0.335	APEX1, CDK1, DDX1, FUS, HMGB1, HNRNPU, KPNA2, NPM1, OTUB1, PCNA, PRKDC, PRPF19, RAD23B, TRIM28, UBE2N, VCP, XRCC5, XRCC6, YBX1	19
Cell-to-cell signaling and interaction	Binding of leukemia cell lines	3.20 × 10^-4^	0.415	ANXA2, ANXA5, CD44, HLA-A, ITGB1, NCL, TNC	7
Molecular transport, RNA trafficking	Export of RNA	3.20 × 10^-4^	−1.974	ALYREF, DDX39B, EIF5A, KHDRBS1, RAN, U2AF2, XPO1	7
Cancer, hematological disease, immunological disease, organismal injury and abnormalities	Large-cell KI-1 lymphoma	3.20 × 10^-4^		HMGB1, PSMB1, PSMD2, TUBB, TUBB4B, TUBB6, TUBB8	7
Cellular assembly and organization, cellular function and maintenance	Organization of microtubules	3.20 × 10^-4^	−1.000	DPYSL2, GAPDH, MAPRE1, NUMA1, PCM1, RRBP1, TUBB	7
Cancer, organismal injury and abnormalities, reproductive system disease	Metastatic breast cancer	3.25 × 10^-4^		FDPS, FKBP1A, FLNA, HSP90AA1, HSP90AB1, HSP90B1, STAT1, TUBB, TUBB4B	9
Immunological disease, inflammatory disease, inflammatory response, organismal injury and abnormalities, renal and urological disease	IGA nephropathy	3.31 × 10^-4^		ACTN4, IMPDH2, PDLIM1, PSMB1, PSMD2	5
Cellular movement, hematological system development and function, immune cell trafficking	Cell rolling of granulocytes	3.47 × 10^-4^	0.000	ANXA1, CD44, CTTN, GNAI2, ITGB1, TLN1	6
DNA replication, recombination, and repair	Conformational modification of DNA	3.47 × 10^-4^		HMGB1, MCM4, MCM6, MCM7, NCL, SSRP1	6
Cancer	Locally advanced malignant tumor	3.47 × 10^-4^		ATIC, FKBP1A, HSP90AA1, HSP90AB1, HSP90B1, TUBB4B	6
Organismal survival	Viability	3.53 × 10^-4^	−1.671	B2M, GNAI2, MAP1B, PFN1, PRKDC, RAN, TKT, VCL, XRCC5	9
Cell-to-cell signaling and interaction, skeletal and muscular system development and function, tissue development	Adhesion of smooth muscle cells	3.56 × 10^-4^		CYR61, FN1, ITGB1, RHOA	4
Cellular assembly and organization, cellular function and maintenance	Quantity of lamellipodia	3.56 × 10^-4^	0.000	ACTR2, ARPC2, ENAH, TPM3	4
Cell morphology	Shape change of leukemia Cell lines	3.56 × 10^-4^		FN1, ITGB1, MAPK1, TNC	4
Cardiovascular disease, organismal injury and abnormalities	Primary cardiomyopathy	3.58 × 10^-4^		LMNA, PGK1, SDHA, TPI1, VCL, VDAC1, VDAC2	7
Cellular growth and proliferation	Formation of cells	3.63 × 10^-4^	−2.209	ACTA1, ACTG1, ACTN4, ANXA2, ARHGDIA, B2M, BSG, CALR, CAPZB, CD44, CTTN, DNAJA1, EEF1D, EHD1, ENAH, EZR, FLNA, FLNB, FLNC, FN1, HEXB, HMGB1, HSP90B1, HSPA4, ITGB1, KRT18, KRT8, LGALS1, MAPK1, MYH10, MYH9, NPEPPS, PAFAH1B2, PEBP1, PLEC, PLS3, PRDX4, RAD23B, RANBP1, RHOA, RPL22, SFN, SOD1, SQSTM1, STAT1, STIP1, STMN1, TCP1, UCHL1, XRCC6, YBX3	51
Cancer, organismal injury and abnormalities, reproductive system disease	Uterine leiomyoma	3.74 × 10^-4^		ALDOA, CYR61, DDX6, HSD17B10, HSP90AB1, IQGAP1, KPNA3, MDH1, MYH10, NPEPPS, PCNA, RPL27, RPS15, SF3A1, TNC, TWF1, VCP, ZYX	18
Cell morphology, cellular assembly and organization, cellular function and maintenance	Reorganization of actin cytoskeleton	3.78 × 10^-4^	−2.219	ARHGDIA, CFL1, DPYSL2, EZR, FLNA, FLNC, FN1, MYH9, PLS3, RHOA, SPTAN1	11
Cancer	Stage 3 cancer	3.78 × 10^-4^		ATIC, CAV1, CBX3, CCT3, FKBP1A, FN1, KRT8, MAPK1, PCNA, TUBB4B, TUBB6	11
Cancer, tumor morphology	Progression of tumor	3.83 × 10^-4^	−0.342	ANXA1, ANXA2, BSG, CAV1, CD44, EZR, FASN, FUS, GSTP1, HMGB1, STAT1, TNC, YWHAZ	13

**Table 6:** 187 categories of vancomycin-associated toxicity analyzed using IPA.

Categories	Disease or Function Annotation	*p*-Value	Activation *z*-Score	Molecules	Number of Molecules
Liver hyperplasia/hyperproliferation	Cholangiocarcinoma	7.33 × 10^−7^		ANXA1, ANXA2, GNB2L1, HSP90AA1, HSP90AB1, PGK1, PKM, RPL4, RPLP0, VIM	10
Renal damage, renal tubule injury	Proximal tubular toxicity	2.17 × 10^−6^		ACAT1, FASN, FN1, G6PD, GSTP1, HADH, HADHA, HSP90AA1, HSPB1, LGALS1, YBX1, YWHAH	12
Nephrosis	Nephrosis	1.35 × 10^−5^		ACTN4, ANXA1, ARHGDIA, CLTC, FKBP1A, IMPDH2, ITGB1, LAMB1, PDLIM1	9
Liver fibrosis	Migration of hepatic stellate cells	1.43 × 10^−4^	−0.218	CD44, GNB2L1, HMGB1, LGALS1, MYH10	5
Nephrosis	Minimal change nephrotic syndrome	2.66 × 10^−4^		ACTN4, FKBP1A, IMPDH2, PDLIM1	4
Renal necrosis/cell death	Cell viability of kidney cell lines	2.85 × 10^−4^	−0.378	ANXA5, CAV1, EZR, HNRNPU, HSP90B1, HYOU1, PPIA, VDAC1	8
Renal inflammation, renal nephritis	IgA nephropathy	3.31 × 10^−4^		ACTN4, IMPDH2, PDLIM1, PSMB1, PSMD2	5
Renal inflammation, renal nephritis	Fechtner syndrome	5.69 × 10^−4^		MYH10, MYH9	2
Liver cirrhosis	Cryptogenic cirrhosis	5.69 × 10^−4^		KRT18, KRT8	2
Renal inflammation, renal nephritis	Diffuse proliferative lupus nephritis	5.69 × 10^−4^		ACTN4, PDLIM1	2
Renal inflammation, renal nephritis	Epstein syndrome	5.69 × 10^−4^		MYH10, MYH9	2
Liver cirrhosis	Susceptibility to noncryptogenic cirrhosis	5.69 × 10^−4^		KRT18, KRT8	2
Liver hyperplasia/hyperproliferation	Liver cancer	1.13 × 10^−3^	1.133	ACLY, ACTG1, ACTR2, ACTR3, AHNAK, ANXA1, ANXA2, AP1B1, APEX1, ARF1, ARHGDIA, ATIC, ATP5A1, ATP5B, B2M, BSG, CACYBP, CAPG, CAST, CBX3, CCT6A, CCT8, CD44, CLIC1, CLTC, COPA, COPG1, COTL1, CRIP2, CSE1L, CTNNA1, CTPS1, CTSA, CTTN, CUTA, CYR61, DBI, DDX3X, DLAT, DNAJA1, DNM1L, DYNC1H1, EEF1A1, EEF2, EIF3I, EIF4A1, ENAH, ENO2, EPRS, FASN, FKBP1A, FLNA, FLNB, FLNC, FN1, GARS, GCN1L1, GDI2, GLUD1, GMPS, GNB2L1, GPI, GSTP1, H2AFY, H3F3A/H3F3B, HADHA, HDLBP, HEXB, HMGB1, HNRNPA1, HNRNPA2B1, HNRNPH1, HNRNPK, HNRNPM, HSP90AA1, HSP90AB1, HSPA5, HSPA8, HSPD1, IARS, IFITM3, IGF2R, ILF2, ILF3, IMPDH2, IPO5, IPO7, IQGAP1, ITGB1, KHSRP, KIF5B, KPNB1, KRT9, LAMB1, LDHA, LMNA, LOC102724594/U2AF1, LONP1, LRPPRC, LRRC59, MAP1B, MAPRE1, MARS, MCM3, MCM4, MCM6, MCM7, MDH1, MVP, MYH10, NACA, NAP1L4, NASP, NNMT, NPEPPS, NPLOC4, NPM1, NUMA1, P4HB, PA2G4, PAICS, PCBP2, PDIA4, PFKP, PFN1, PGK1, PGM1, PHGDH, PKM, PLEC, PPM1G, PPP2CA, PPP2R1A, PRDX1, PRKCSH, PRKDC, PSMC1, PSMD2, PTRF, PUF60, PYGL, QARS, RAN, RPL12, RPL4, RPL7A, RPLP0, RPN1, RPS2, RPS24, RPS4X, RRBP1, RTN4, SDHA, SF3A1, SF3B1, SFPQ, SH3BGRL3, SLC38A2, SND1, SOD1, SPTBN1, SQSTM1, STAT1, TPI1, TUBB4B, TWF2, UAP1, UBE2N, UBXN4, UCHL1, VARS, VCL, VCP, VIM, WARS, XRCC6, YBX3, YWHAG, YWHAH	180
Liver hyperplasia/hyperproliferation	Liver tumor	1.16 × 10^−3^	0.590	ACLY, ACTG1, ACTR2, ACTR3, AHNAK, ANXA1, ANXA2, AP1B1, APEX1, ARF1, ARHGDIA, ATIC, ATP5A1, ATP5B, B2M, BSG, CACYBP, CAPG, CAST, CBX3, CCT6A, CCT8, CD44, CLIC1, CLTC, COPA, COPG1, COTL1, CRIP2, CSE1L, CTNNA1, CTPS1, CTSA, CTTN, CUTA, CYR61, DBI, DDX3X, DLAT, DNAJA1, DNM1L, DYNC1H1, EEF1A1, EEF2, EIF3I, EIF4A1, ENAH, ENO2, EPRS, FASN, FKBP1A, FLNA, FLNB, FLNC, FN1, GARS, GCN1L1, GDI2, GLUD1, GMPS, GNB2L1, GPI, GSTP1, H2AFY, H3F3A/H3F3B, HADHA, HDLBP, HEXB, HMGB1, HNRNPA1, HNRNPA2B1, HNRNPH1, HNRNPK, HNRNPM, HSP90AA1, HSP90AB1, HSPA5, HSPA8, HSPD1, IARS, IFITM3, IGF2R, ILF2, ILF3, IMPDH2, IPO5, IPO7, IQGAP1, ITGB1, KHSRP, KIF5B, KPNB1, KRT9	181
				LAMB1, LDHA, LMNA, LOC102724594/U2AF1, LONP1, LRPPRC, LRRC59, MAP1B, MAPRE1, MARS, MCM3, MCM4, MCM6, MCM7, MDH1, MVP, MYH10, NACA, NAP1L4, NASP, NNMT, NPEPPS, NPLOC4, NPM1, NUMA1, P4HB, PA2G4, PAICS, PCBP2, PDIA4, PFKP, PFN1, PGK1, PGM1, PHGDH, PKM, PLEC, PPM1G, PPP2CA, PPP2R1A, PRDX1, PRKCSH, PRKDC, PSMC1, PSMD2, PTRF, PUF60, PYGL, QARS, RAN, RPL12, RPL4, RPL7A, RPLP0, RPN1, RPS2, RPS24, RPS4X, RRBP1, RTN4, S100A6, SDHA, SF3A1, SF3B1, SFPQ, SH3BGRL3, SLC38A2, SND1, SOD1, SPTBN1, SQSTM1, STAT1, TPI1, TUBB4B, TWF2, UAP1, UBE2N, UBXN4, UCHL1, VARS, VCL, VCP, VIM, WARS, XRCC6, YBX3, YWHAG, YWHAH	
Liver damage, liver inflammation/hepatitis	Susceptibility to hepatitis c virus	1.68 × 10^−3^		KRT18, KRT8	2
Renal necrosis/cell death	Cell death of kidney cell lines	2.01 × 10^−3^	0.222	CD44, CDK1, EZR, GAPDH, GSTP1, HSPA5, HSPB1, HYOU1, ITGB1, LDHA, PEA15, PRKDC, SOD1, SSRP1, TFRC, VCP, VDAC1, YWHAQ	18
Cardiac necrosis/cell death	Cell death of cardiomyocytes	2.22 × 10^−3^	1.920	CALR, CYR61, GAPDH, GNAI2, HADHA, HSPB1, HSPD1, MAPK1, NCL, PARK7, RHOA, RPSA, STAT1, ZYX	14
Renal necrosis/cell death	Cell death of kidney cells	2.29 × 10^−3^	0.515	ATP1A1, CD44, CDK1, EZR, GAPDH, GSTP1, HSPA5, HSPB1, HYOU1, ITGB1, LDHA, PEA15, PRKDC, SOD1, SSRP1, STMN1, TFRC, VCP, VDAC1, YWHAQ	20
Renal proliferation	Proliferation of kidney cell lines	4.64 × 10^−3^	−2.423	HSP90AA1, HSP90AB1, HSPA4, HSPD1, ITGB1, LMNA, NCL, PPP2R1A, RHOA, VDAC1, YBX3	11
Cardiac damage, cardiac degeneration	Degeneration of cardiomyocytes	4.98 × 10^−3^		CAV1, HADHA, MAPK1	3
Nephrosis	Autosomal recessive steroid-resistant nephrotic syndrome	5.43 × 10^−3^		ARHGDIA, IMPDH2	2
Decreased levels of albumin	Increased uptake of albumin	5.43 × 10^−3^		CAV1, FLNA	2
Cardiac hypertrophy	Hypertrophy of heart cells	5.90 × 10^−3^	−0.243	CAV1, CTSB, EEF1D, FN1, HMGB1, IGF2R, MAPK1, PPIA, RHOA, S100A6	10
Hepatocellular carcinoma, liver hyperplasia/hyperproliferation	Hepatocellular carcinoma	5.96 × 10^−3^	1.133	ACTG1, ARF1, BSG, COPG1, CSE1L, CTTN, FKBP1A, GLUD1, GNB2L1, H2AFY, HMGB1, HNRNPA1, HSPA5, IFITM3, IGF2R, IPO7, IQGAP1, MAPRE1, NNMT, PFN1, PPM1G, PRDX1, SOD1, SPTBN1, TPI1, TUBB4B, YWHAG	27
Cardiac necrosis/cell death	Cell viability of cardiomyocytes	6.07 × 10^−3^		CALR, HSPD1, IQGAP1, RHOA	4
Liver fibrosis, liver proliferation	Proliferation of hepatic stellate cells	6.67 × 10^−3^	−0.847	CTSB, ITGB1, LGALS1, SERPINH1, STAT1	5
Cardiac arrythmia	Chronic atrial fibrillation	8.01 × 10^−3^		PPP1CA, PPP2CA	2
Renal dilation	Dilation of collecting tubule	8.01 × 10^−3^		ARHGDIA, PRKCSH	2
Kidney failure	Acute renal failure	9.94 × 10^−3^		ATP1A1, CYR61, GSTP1, HEXB, STMN1	5
Liver damage, liver inflammation/hepatitis	Hepatitis c	1.51 × 10^−2^		DDX5, FKBP1A, IMPDH2, KRT18, KRT8	5
Liver damage	Damage of liver	1.67 × 10^−2^	0.513	BSG, CD44, CTSB, DDX5, FKBP1A, GSTP1, HLA-B, HMGB1, IMPDH2, KRT18, KRT8, SOD1, STAT1	13
Cardiac inflammation	Inflammation of heart	1.76 × 10^−2^	0.686	B2M, CAV1, HMGB1, PPIA	4
Glomerular injury	Focal segmental glomerulosclerosis	1.89 × 10^−2^		ACTN4, MYH9, PDLIM1	3
Cardiac dilation	Dilation of left ventricle	1.90 × 10^−2^	−1.000	CAPNS1, CAV1, LMNA, RHOA	4
Glomerular injury	Autosomal dominant focal segmental glomerulosclerosis	2.39 × 10^−2^		ACTN4	1
Liver inflammation/hepatitis	Chronic phase hepatitis	2.39 × 10^−2^		HLA-A	1
Decreased levels of albumin	Decreased accumulation of albumin	2.39 × 10^−2^		CAV1	1
Renal degeneration	Degeneration of renal tubular epithelial cells	2.39 × 10^−2^		ARHGDIA	1
Cardiac dysplasia	Dysplasia of right ventricle	2.39 × 10^−2^		RPSA	1
Cardiac arrythmia, tachycardia	Familial ventricular tachycardia	2.39 × 10^−2^		GNAI2	1
Cardiac fibrosis	Fibrosis of right ventricle	2.39 × 10^−2^		CTNNA1	1
Kidney failure	Gentamicin-induced renal injury	2.39 × 10^−2^		HEXB	1
Glomerular injury	Glomerulopathy with fibronectin deposits type 2	2.39 × 10^−2^		FN1	1
Pulmonary hypertension	Hypertension of pulmonary artery	2.39 × 10^−2^		CAV1	1
Increased levels of albumin	Increased export of albumin	2.39 × 10^−2^		RAN	1
Liver failure	Infantile liver failure syndrome type 2	2.39 × 10^−2^		MARS	1
Liver hyperplasia/hyperproliferation	Nodular hyperplasia of liver	2.39 × 10^−2^		SOD1	1
Pulmonary hypertension	Primary pulmonary hypertension 3	2.39 × 10^−2^		CAV1	1
Cardiac damage	Rupture of ventricular septum	2.39 × 10^−2^		CTNNA1	1
Bradycardia, cardiac arrythmia	Severe bradycardia	2.39 × 10^−2^		RHOA	1
Kidney failure	Failure of kidney	2.48 × 10^−2^		ACTN4, ARHGDIA, ATP1A1, CYR61, FDPS, FKBP1A, GSTP1, HEXB, IMPDH2, MYH9, STMN1	11
Renal inflammation, renal nephritis	Autoimmune glomerulonephritis	2.85 × 10^−2^		ACTN4, PDLIM1, PPP2CA	3
Liver cirrhosis	Cirrhosis	3.11 × 10^−2^		B2M, BSG, FN1, GSTP1, HNRNPK, KRT18, KRT8, PCNA, TXLNA	9
Liver necrosis/cell death	Necrosis of liver	3.18 × 10^−2^	0.791	CTSB, FKBP1A, FLNA, HMGB1, HSPD1, KRT8, NCL, SERPINH1, SLC25A5, SOD1, SPTBN1, STAT1	12
Cardiac damage	Damage of heart	3.20 × 10^−2^	0.156	ANXA1, CAV1, CTNNA1, HADHA, MAPK1	5
Liver inflammation/hepatitis	Inflammation of liver	3.24 × 10^−2^	1.231	CD44, CYR61, DDX5, FKBP1A, GSTP1, HLA-A, HMGB1, IMPDH2, KRT18, KRT8, PPIA, SOD1, STAT1	13
Renal inflammation, renal nephritis	Lupus nephritis	3.28 × 10^−2^		ACTN4, FKBP1A, IMPDH2, PDLIM1, PSMB1, PSMD2	6
Cardiac arrythmia	Arrhythmia	3.34 × 10^−2^	−1.103	ATP1A1, CALR, GNAI2, HMGB1, PPP1CA, PPP2CA, RHOA, TUBB, TUBB4B, VCL	10
Liver damage	Injury of liver	3.41 × 10^−2^	−0.447	BSG, CTSB, HLA-B, HMGB1, KRT8, SOD1, STAT1	7
Hepatocellular carcinoma, liver hyperplasia/hyperproliferation	Incidence of hepatocellular carcinoma	3.54 × 10^−2^	1.718	IQGAP1, PRDX1, SOD1, SPTBN1	4
Glomerular injury, renal hypertrophy	Hypertrophy of mesangial cells	3.73 × 10^−2^		MAPK1, PARK7	2
Liver hepatomegaly	Hepatomegaly	4.07 × 10^−2^		CTSA, HADHA, IGF2R, KRT18, STAT1	5
Cardiac hypertrophy	Hypertrophy of cardiomyocytes	4.35 × 10^−2^	−0.243	CAV1, FN1, HMGB1, IGF2R, MAPK1, PPIA, RHOA	7
Cardiac arrythmia	Supraventricular arrhythmia	4.35 × 10^−2^		ATP1A1, CALR, PPP1CA, PPP2CA, RHOA, TUBB, TUBB4B	7
Congenital heart anomaly	Ventricular septal defect	4.67 × 10^−2^	0.128	AIP, FKBP1A, FLNA, MYH10, MYH9, YWHAE	6
Liver degeneration	Degeneration of hepatocytes	4.72 × 10^−2^		XRCC5	1
Increased levels of potassium	Increased efflux of k+	4.72 × 10^−2^		MAPK1	1
Liver fibrosis, liver proliferation	Proliferation of hepatic stellate cells/myofibroblasts	4.72 × 10^−2^		CTSB	1
Cardiac regeneration	Regeneration of cardiomyocytes	4.72 × 10^−2^		HMGB1	1
Cardiac regeneration	Regeneration of myocardium	4.72 × 10^−2^		HMGB1	1
Liver hyperplasia/hyperproliferation	Hyperplasia of liver	4.87 × 10^−2^		IGF2R, SOD1	2
Renal inflammation, renal nephritis	Membranous glomerulonephritis	4.87 × 10^−2^		ACTN4, PDLIM1	2
Renal necrosis/cell death	Apoptosis of kidney cell lines	5.43 × 10^−2^	−0.777	CD44, CDK1, EZR, HSPA5, HSPB1, HYOU1, ITGB1, PEA15, TFRC, VDAC1, YWHAQ	11
Cardiac necrosis/cell death	Apoptosis of cardiomyocytes	5.55 × 10^−2^	1.348	CALR, CYR61, GAPDH, GNAI2, HSPD1, MAPK1, PARK7, RHOA, STAT1	9
Cardiac hypertrophy	Hypertrophy of heart	5.62 × 10^−2^	−0.859	CAV1, CTSB, EEF1D, FN1, GNAI2, HMGB1, IGF2R, MAPK1, MYH10, PARK7, PFN1, PPIA, RHOA, S100A6, TLN1	15
Liver cirrhosis	Cirrhosis of liver	6.13 × 10^−2^		B2M, BSG, FN1, HNRNPK, KRT18, KRT8, TXLNA	7
Cardiac arrythmia	Atrial fibrillation	6.18 × 10^−2^		ATP1A1, PPP1CA, PPP2CA, RHOA, TUBB, TUBB4B	6
Cardiac inflammation	Pericarditis	6.77 × 10^−2^		TUBB, TUBB4B	2
Liver steatosis	Advanced stage hepatic steatosis	7.00 × 10^−2^		CD44	1
Cardiac arteriopathy, cardiac hypertrophy	Hypertrophy of coronary artery	7.00 × 10^−2^		RHOA	1
Glomerular injury, renal hypoplasia	Hypoplasia of mesangial cells	7.00 × 10^−2^		ARHGDIA	1
Cardiac inflammation	Rheumatic carditis	7.00 × 10^−2^		ANXA1	1
Cardiac arteriopathy	Vasospasm of coronary artery	7.00 × 10^−2^		SOD1	1
Liver fibrosis	Fibrosis of liver	8.42 × 10^−2^	0.391	CTSB, GNB2L1, GSTP1, HDGF, HSPB1, STAT1	6
Cardiac fibrosis	Fibrosis of heart	9.03 × 10^−2^	−0.956	CAPNS1, CAV1, CTNNA1, FN1, ITGB1, LMNA, RHOA, TLN1	8
Liver hypoplasia	Hypoplasia of liver	9.21 × 10^−2^		EIF6, SPTBN1, YBX1	3
Renal damage, renal tubule injury	Damage of tubular cells	9.22 × 10^−2^		YBX1	1
Glomerular injury, renal fibrosis	Fibrosis of renal tubule	9.22 × 10^−2^		STMN1	1
Renal inflammation, renal nephritis	Progressive crescentic glomerulonephritis	9.22 × 10^−2^		ACTN4	1
Renal inflammation, renal nephritis	Nephritis	1.12 × 10^−1^		ACTN4, ARHGDIA, FKBP1A, IMPDH2, MYH10, MYH9, PDLIM1, PPP2CA, PSMB1, PSMD2, YBX1	11
Renal damage	Damage of kidney	1.13 × 10^−1^	−0.447	ARHGDIA, B2M, CD44, FN1, ITGB1, YBX1	6
Renal proliferation	Arrest in growth of kidney cell lines	1.14 × 10^−1^		LMNA	1
Cardiac enlargement	Enlargement of atrium	1.14 × 10^−1^		RHOA	1
Cardiac arteriopathy, cardiac fibrosis	Fibrosis of coronary artery	1.14 × 10^−1^		RHOA	1
Cardiac damage, cardiac degeneration	Injury of cardiomyocytes	1.14 × 10^−1^		MAPK1	1
Liver steatosis	Nonalcoholic fatty liver disease	1.22 × 10^−1^		FASN, HADHA, SOD1	3
Liver damage	Hepatotoxicity	1.27 × 10^−1^		AIP, HLA-B	2
Heart failure	Failure of heart	1.31 × 10^−1^	−0.798	ATP1A1, CAPNS1, CAV1, FKBP1A, HMGB1, IGF2R, MAPK1, MYH10, PARK7, PPP2R1A	10
Liver proliferation	Proliferation of liver cells	1.34 × 10^−1^	−1.461	CAV1, CTSB, ITGB1, LGALS1, MAPK1, SERPINH1, SPTBN1, STAT1	8
Kidney failure	Acute tubular necrosis	1.35 × 10^−1^		STMN1	1
Liver adhesion	Adhesion of hepatocytes	1.35 × 10^−1^		KRT8	1
Heart failure	End stage heart failure	1.35 × 10^−1^		PARK7	1
Liver enlargement	Enlargement of liver	1.35 × 10^−1^		LMNA	1
Liver hyperplasia/hyperproliferation	Polycystic liver disease	1.35 × 10^−1^		PRKCSH	1
Cardiac necrosis/cell death	Survival of ventricular myocytes	1.35 × 10^−1^		CALR	1
Renal transformation	Transformation of kidney cells	1.35 × 10^−1^		EIF2S1	1
Cardiac dysfunction	Systolic dysfunction	1.52 × 10^−1^		ANXA5, FKBP1A	2
Liver necrosis/cell death	Cell death of liver cells	1.52 × 10^−1^	0.256	CTSB, HMGB1, HSPD1, KRT8, NCL, SERPINH1, SLC25A5, SPTBN1	8
Renal proliferation	Proliferation of renal tubular epithelial cells	1.56 × 10^−1^		STMN1	1
Cardiac arrythmia, tachycardia	Ventricular tachycardia	1.61 × 10^−1^		ATP1A1, GNAI2, VCL	3
Liver hemorrhaging	Bleeding of liver	1.69 × 10^−1^		FKBP1A, KRT8	2
Renal inflammation, renal nephritis	Acute phase crescentic glomerulonephritis	1.76 × 10^−1^		ACTN4	1
Liver fibrosis	Chemotaxis of hepatic stellate cells	1.76 × 10^−1^		MAPK1	1
Increased levels of albumin	Increased localization of albumin	1.76 × 10^−1^		B2M	1
Hepatocellular peroxisome proliferation	Quantity of peroxisomes	1.76 × 10^−1^		DNM1L	1
Bradycardia, cardiac arrythmia	Sinus bradycardia	1.76 × 10^−1^		CALR	1
Cardiac inflammation	Carditis	1.85 × 10^−1^		ANXA1, TUBB, TUBB4B	3
Bradycardia, cardiac arrythmia	Bradycardia	1.86 × 10^−1^		CALR, RHOA	2
Renal dilation	Dilation of kidney	1.96 × 10^−1^		PRKCSH	1
Hepatocellular carcinoma, liver hyperplasia/hyperproliferation	Size of hepatocellular carcinoma	1.96 × 10^−1^		IQGAP1	1
Liver cirrhosis	Primary biliary cirrhosis	1.97 × 10^−1^		B2M, BSG, HNRNPK, TXLNA	4
Liver necrosis/cell death	Cell death of hepatocytes	2.11 × 10^−1^	−0.537	CTSB, HMGB1, KRT8, NCL, SLC25A5, SPTBN1	6
Glomerular injury	Glomerulosclerosis	2.13 × 10^−1^		ACTN4, ARHGDIA, MYH9, PDLIM1, STMN1	5
Renal necrosis/cell death	Apoptosis of proximal tubule cells	2.15 × 10^−1^		ATP1A1	1
Cardiac infarction	Infarction of heart	2.15 × 10^−1^		CAV1	1
Kidney failure	Ischemic acute renal failure	2.15 × 10^−1^		ATP1A1	1
Renal necrosis/cell death	Apoptosis of tubular cells	2.21 × 10^−1^		ATP1A1, STMN1	2
Liver necrosis/cell death	Apoptosis of liver cells	2.22 × 10^−1^	0.000	CTSB, HMGB1, KRT8, NCL, SERPINH1, SPTBN1	6
Increased levels of creatinine	Increased quantity of creatinine	2.30 × 10^−1^		AKR1B1, CD44	2
Glomerular injury	Cortical renal glomerulopathies	2.34 × 10^−1^		ACTN4	1
Cardiac hyperplasia/hyperproliferation	Hyperplasia of heart	2.34 × 10^−1^		IGF2R	1
Liver necrosis/cell death	Apoptosis of hepatocytes	2.48 × 10^−1^	−0.492	CTSB, HMGB1, KRT8, NCL, SPTBN1	5
Decreased levels of albumin	Decreased excretion of albumin	2.52 × 10^−1^		LAMB1	1
Cardiac proliferation	Proliferation of cardiac fibroblasts	2.52 × 10^−1^		STAT1	1
Cardiac congestive cardiac failure, heart failure	Congestive heart failure	2.58 × 10^−1^	−1.103	CAV1, FKBP1A, IGF2R, MYH10	4
Cardiac damage	Injury of heart	2.65 × 10^−1^		ANXA1, MAPK1	2
Renal damage, renal tubule injury	Damage of tubulointerstitium	2.70 × 10^−1^		FN1	1
Increased levels of creatinine	Increased clearance of creatinine	2.70 × 10^−1^		ARHGDIA	1
Renal damage, renal tubule injury	Damage of renal tubule	2.74 × 10^−1^		FN1, YBX1	2
Liver steatosis	Hepatic steatosis	2.75 × 10^−1^	1.715	CAV1, CD44, FASN, GSTP1, H2AFY, HADHA, KRT8, LMNA, SOD1	9
Congenital heart anomaly	Congenital heart disease	2.85 × 10^−1^	0.294	AIP, ENO2, FKBP1A, FLNA, MYH10, MYH9, YWHAE	7
Liver inflammation/hepatitis	Acute hepatitis	3.04 × 10^−1^		CD44	1
Increased levels of blood urea nitrogen	Increased quantity of blood urea nitrogen	3.04 × 10^−1^		CD44	1
Liver fibrosis	Activation of hepatic stellate cells	3.10 × 10^−1^		FN1, STAT1	2
Liver damage, liver inflammation/hepatitis	Chronic hepatitis c	3.10 × 10^−1^		DDX5, IMPDH2	2
Kidney failure	End stage renal disease	3.12 × 10^−1^		FDPS, FKBP1A, IMPDH2, MYH9	4
Liver necrosis/cell death	Focal necrosis of liver	3.21 × 10^−1^		STAT1	1
Increased levels of potassium	Increased excretion of k+	3.21 × 10^−1^		AKR1B1	1
Renal inflammation, renal nephritis	Glomerulonephritis	3.23 × 10^−1^		ACTN4, FKBP1A, IMPDH2, PDLIM1, PPP2CA, PSMB1, PSMD2	7
Cardiac arteriopathy	Coronary artery disease	3.23 × 10^−1^		ACAT2, EIF4H, FKBP1A, NNMT, RHOA, SOD1, TKT, TUBB, TUBB4B	9
Cardiac infarction	Myocardial infarction	3.27 × 10^−1^	2.000	ACTA1, CCT7, CD44, MAPK1, SOD1, STAT1	6
Renal damage	Injury of kidney	3.32 × 10^−1^		ARHGDIA, B2M, CD44	3
Renal inflammation, renal nephritis	Interstitial nephritis	3.37 × 10^−1^		ARHGDIA	1
Renal necrosis/cell death	Apoptosis of kidney cells	3.51 × 10^−1^		ATP1A1, ITGB1, STMN1	3
Cardiac hypoplasia	Hypoplasia of heart ventricle	3.62 × 10^−1^		CALR, MYH10	2
Liver necrosis/cell death	Apoptosis of hepatic stellate cells	3.68 × 10^−1^		SERPINH1	1
Liver hyperbilirubinemia	Hyperbilirubinemia	3.68 × 10^−1^		GSTP1	1
Renal damage	Injury of renal glomerulus	3.68 × 10^−1^		B2M	1
Glutathione depletion in liver	Conjugation of glutathione	3.84 × 10^−1^		GSTP1	1
Cardiac fibrosis	Fibrosis of myocardium	3.84 × 10^−1^		ITGB1	1
Increased levels of hematocrit	Increased hematocrit of organism	4.03 × 10^−1^		CD59, PRDX1, PRDX2	3
Cardiac hypertrophy	Hypertrophy of ventricular myocytes	4.13 × 10^−1^		RHOA	1
Liver cholestasis	Progressive familial intrahepatic cholestasis type 1	4.13 × 10^−1^		HDLBP, SLC25A6	2
Increased levels of red blood cells	Increased quantity of red blood cells	4.22 × 10^−1^		CD59, PICALM, SOD1	3
Renal necrosis/cell death	Apoptosis of mesangial cells	4.27 × 10^−1^		ITGB1	1
Renal dysfunction	Dysfunction of kidney	4.40 × 10^−1^		BSG	1
Congenital heart anomaly	Perimembranous ventricular septal defect	4.40 × 10^−1^		MYH10	1
Cardiac hypertrophy	Hypertrophy of right ventricle	4.54 × 10^−1^		CAV1	1
Cardiac necrosis/cell death	Apoptosis of ventricular myocytes	4.67 × 10^−1^		RHOA	1
Cardiac dilation	Dilation of heart	4.67 × 10^−1^		PPP2CA	1
Cardiac fibrosis	Interstitial fibrosis of heart	4.67 × 10^−1^		CAPNS1	1
Cardiac arrythmia	Ventricular fibrillation	4.67 × 10^−1^		ATP1A1	1
Cardiac arrythmia	Arrhythmia of heart ventricle	4.78 × 10^−1^		ATP1A1, GNAI2	2
Liver hyperplasia/hyperproliferation	Proliferation of liver cancer cells	4.80 × 10^−1^		S100A6	1
Increased levels of alkaline phosphatase	Increased activation of alkaline phosphatase	4.93 × 10^−1^		ANXA6, ITGB1	2
Renal damage	Reperfusion injury of kidney	5.04 × 10^−1^		CD44	1
Cardiac arrythmia, tachycardia	Supraventricular tachycardia	5.28 × 10^−1^		ATP1A1	1
Liver inflammation/hepatitis	Alcoholic hepatitis	5.39 × 10^−1^		GSTP1	1
Renal proliferation	Proliferation of kidney cells	5.45 × 10^−1^		AKR1B1, STMN1	2
Congenital heart anomaly	Conotruncal heart malformations	5.59 × 10^−1^		AIP, FLNA	2
Congenital heart anomaly	Persistent truncus arteriosus	5.61 × 10^−1^		FLNA	1
Cardiac hypoplasia	Hypoplasia of trabeculae carne	5.92 × 10^−1^		CALR	1
Cardiac output	Cardiac output	6.01 × 10^−1^		MAPK1	1
Liver inflammation/hepatitis, liver steatosis	Steatohepatitis	6.29 × 10^−1^		SOD1	1
Cardiac hypertrophy	Hypertrophy of left ventricle	1.00E00		CAV1	1
Liver proliferation	Proliferation of hepatocytes	1.00E00		CAV1, MAPK1, SPTBN1	3
Cardiac hypertrophy	Ventricular hypertrophy	1.00E00		CAV1, RHOA	2

### 2.3. Vancomycin Induces Toxicity in HK-2 Cells

After we observed the effect of vancomycin on cellular functions and disease development, we also analysed the toxic effect of vancomycin in HK-2 cells. As shown in Table 6, there were 187 different toxic effects of vancomycin which were predicted in HK-2 cells. The proteomic results showed that treatment of vancomycin induced renal, heart, and liver toxicity, with cell necrosis and cell death. Moreover, toxic function analysis showed that there were 500 different diseases and functions which were regulated by vancomycin in HK-2 cells (Table 5). Notably, the analysis showed that vancomycin primarily induced renal toxicity, to a lesser extent, liver and heart toxicity. Vancomycin-induced renal toxicity included renal degradation, renal inflammation, renal nephritis, renal atrophy, renal hydronephrosis, kidney failure, renal hypoplasia, glomerular injury, renal fibrosis, renal necrosis/cell death, renal damage, and renal tubule injury. These are all observed in clinical studies.

Following the analysis of the effect of vancomycin on the cellular function and toxicity in HK-2 cells, we analyzed the effect of vancomycin on cellular signaling pathways, including the G_2_/M DNA damage check point signaling pathway, apoptosis signaling pathway, autophagy signaling pathway, endoplasmic reticulum (ER) stress signaling pathway, unfolded protein response (URP) signaling pathway, ERK-MAPK signaling pathway, and tight junction signaling pathway.

### 2.4. Vancomycin Regulates G_2_/M DNA Damage Check Point Signaling Pathway in HK-2 Cells

**Figure 27:**
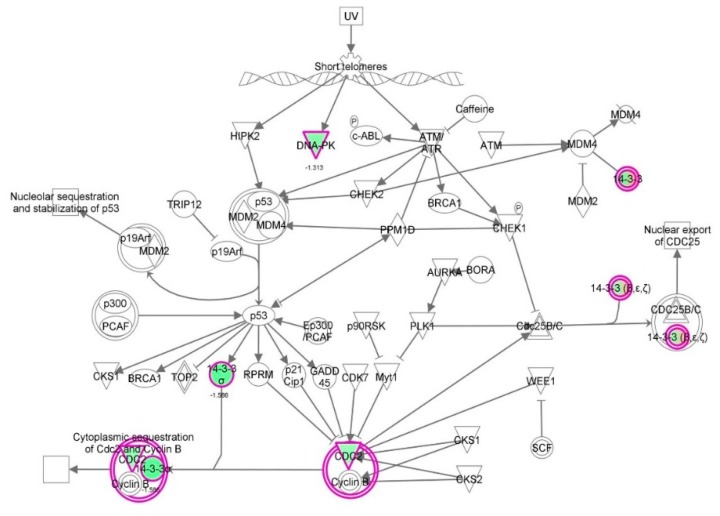
G_2_/M DNA damage check point signaling pathway was regulated by vancomycin in HK-2 cells. HK-2 cells were treated with 50 μg/mL vancomycin for 24 h and the protein samples were subject to quantitative proteomic analysis. Green indicates a down-regulation. The intensity of greencolor indicates the degree of down-regulation. Solid arrow indicates direct interaction.

The proteomic results showed that treatment of HK-2 cells with 50 µg/mL vancomycin regulated G_2_/M DNA damage check point signaling pathway (Table 3). Vancomycin reduced the expression level of CDK1, PRKDC, SFN, YWHAE, YWHAG, YWHAH, and YWHAZ by 1.1-, 1.3-, 1.6-, 1.2-, 1.1-, 1.6-, and 1.1-fold, respectively (Figure 27). However, treatment of vancomycin induced the expression level of YWHAB and YWHAQ by 1.1- and 1.2-fold, respectively. Taken together, the results suggest that vancomycin affects the G_2_/M DNA damage check point signaling pathway in HK-2 cells.

### 2.5. Vancomycin Regulates Apoptosis and Autophagy in HK-2 Cells

**Figure 28:**
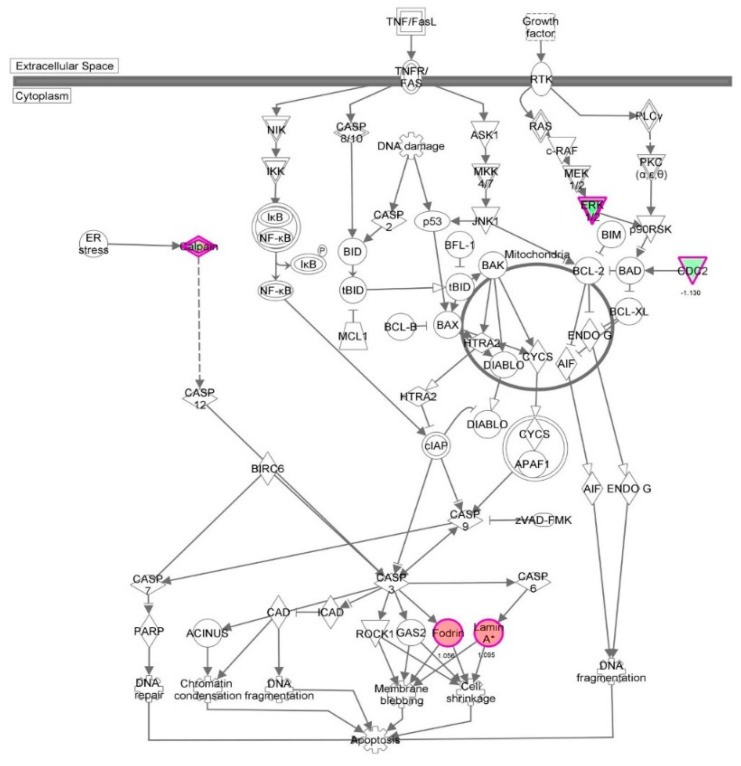
Apoptosis signaling pathway was regulated by vancomycin in HK-2 cells. HK-2 cells were treated with 50 μg/mL vancomycin for 24 h and the protein samples were subject to quantitative proteomic analysis. Red indicates an up-regulation; green indicates a down-regulation. The intensity of green and redcolors indicates the degree of down- or up-regulation. Solid arrow indicates direct interaction and dashed arrow indicates indirect interaction.

**Figure 29:**
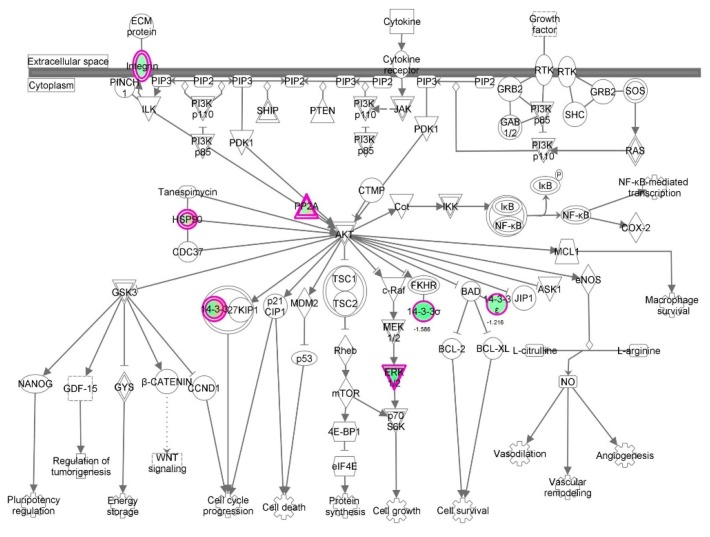
PI3K/Akt signaling pathway was regulated by vancomycin in HK-2 cells. HK-2 cells were treated with 50 μg/mL vancomycin for 24 h and the protein samples were subject to quantitative proteomic analysis. Red indicates an up-regulation; green indicates a down-regulation. The intensity of green and red colors indicates the degree of down- or up-regulation. Solid arrow indicates direct interaction and dashed arrow indicates indirect interaction.

**Figure 30:**
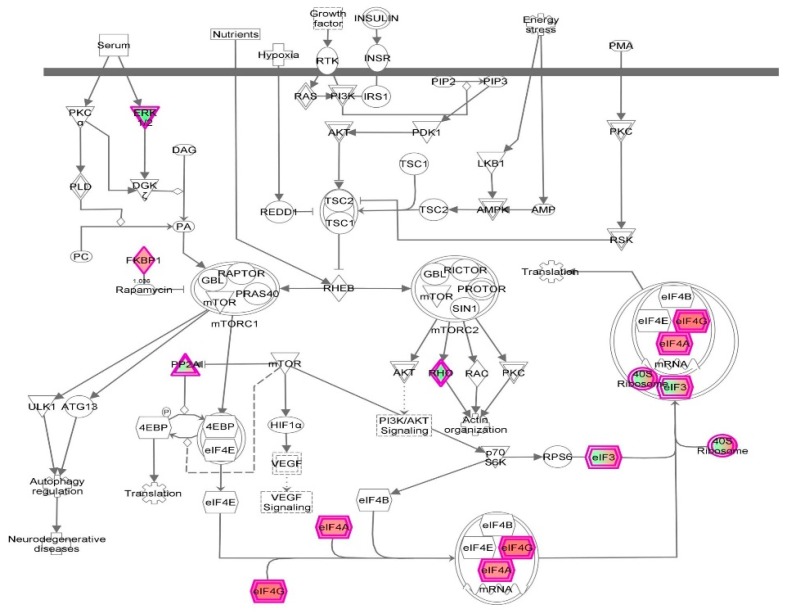
mTOR signaling pathway was regulated by vancomycin in HK-2 cells. HK-2 cells were treated with 50 μg/mL vancomycin for 24 h and the protein samples were subject to quantitative proteomic analysis. Red indicates an up-regulation; green indicates a down-regulation. The intensity of green and red colors indicates the degree of down- or up-regulation. Solid arrow indicates direct interaction and dashed arrow indicates indirect interaction.

**Figure 31:**
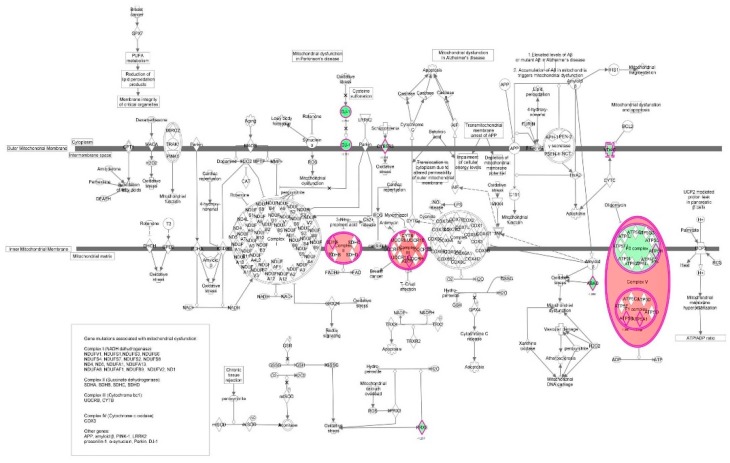
Mitochondrial dysfunction was induced by vancomycin in HK-2 cells. HK-2 cells were treated with 50 μg/ml vancomycin for 24 h and the protein samples were subject to quantitative proteomic analysis. Red indicates an up-regulation; green indicates a down-regulation. The intensity of green and red colors indicates the degree of down- or up-regulation. Solid arrow indicates direct interaction and dashed arrow indicates indirect interaction.

Apoptosis and autophagy are two predominant programmed cell death pathways, being promising targets for the treatment of cancer via regulating mitochondria-dependent or -independent or PI3K/Akt/mTOR-mediated signaling pathways. Furthermore, apoptosis and autophagy have been implicated in the pathogenesis of various diseases, including cancer [12,13,14,15,16]. In order to examine the effect of vancomycin on apoptosis and autophagy in HK-2 cells, the identified protein targets were subject to IPA pathway analysis. The results showed that vancomycin regulated apoptosis signaling pathway via the down-regulation of CAPN1, CDK1, and MAPK1 by 1.3-, 1.1-, and 1.7-fold, respectively (Table 1 and Table 2, and Figure 28). In addition, vancomycin regulated apoptotic signaling pathway via increasing the expression level of CAPN2, CAPNS1, LMNA, and SPTAN1 by 1.4-, 1.2-, 1.1-, and 1.21-fold, respectively. Moreover, Akt/mTOR signaling pathway plays a central role in the regulation of cell metabolism, growth, proliferation and survival through the integration of both intracellular and extracellular signals [17]. mTOR complexs 1 and 2 (mTORC1 and mTORC2) are two distinct complexes in mTOR signaling pathway transducting a variety of signals to downstream targets, including Akt, p70S6K, Atgs, eIF4G, PPAR-α, and PPAR-γ, to modulate cell growth, cell proliferation, energy metabolism, and autophagy [17]. Aberrant mTOR signaling pathway has been implicated in the pathogenesis of many diseases including cancer and targeting mTOR signaling pathway may be a promising strategy for cancer therapy [18]. As showed in Table 3, vancomycin exhibited a capability of modulating PI3K/Akt/mTOR signaling pathway in HK-2 cells (Figure 29 and Figure 30). The results showed that vancomycin decreased the expression level of a number of key proteins, such as HSP90B1, ITGB1, MAPK1, PPP2R1A, SFN, YWHAE, YWHAG, YWHAH, and YWHAZ by 1.1-, 1.2-, 1.7-, 1.2-, 1.6-, 1.2-, 1.1-, 1.6-, and 1.1-fod, respectively; but increased the expression level of EIF3C, EIF3M, EIF4A1, EIF4G1, FKBP1A, PPP2CA, RPS2, RPS3, RPS8, RPS11, RPS15, RPS24, RPS27A, RPS3A, RPS4X, and RPSA. Moreover, treatment of HK-2 cells with vancomycin induced mitochondrial dysfunction with alterations in the expression level of a number of proteins, including ATP5A1, ATP5B, ATP5H, CYB5R3, CYC1, HSD17B10, PARK7, PRDX5, SDHA, UQCRC1, VDAC1, and VDAC2 (Figure 31). Taken together, the results suggest that the regulatory effects of vancomycin on apoptosis signaling pathway, PI3K/Akt/mTOR signaling pathway, and mitochondrial dysfunction contribute to the toxicity of vancomycin in HK-2 cells.

### 2.6. Vancomycin Regulates EMT Pathways in HK-2 cells

**Figure 32:**
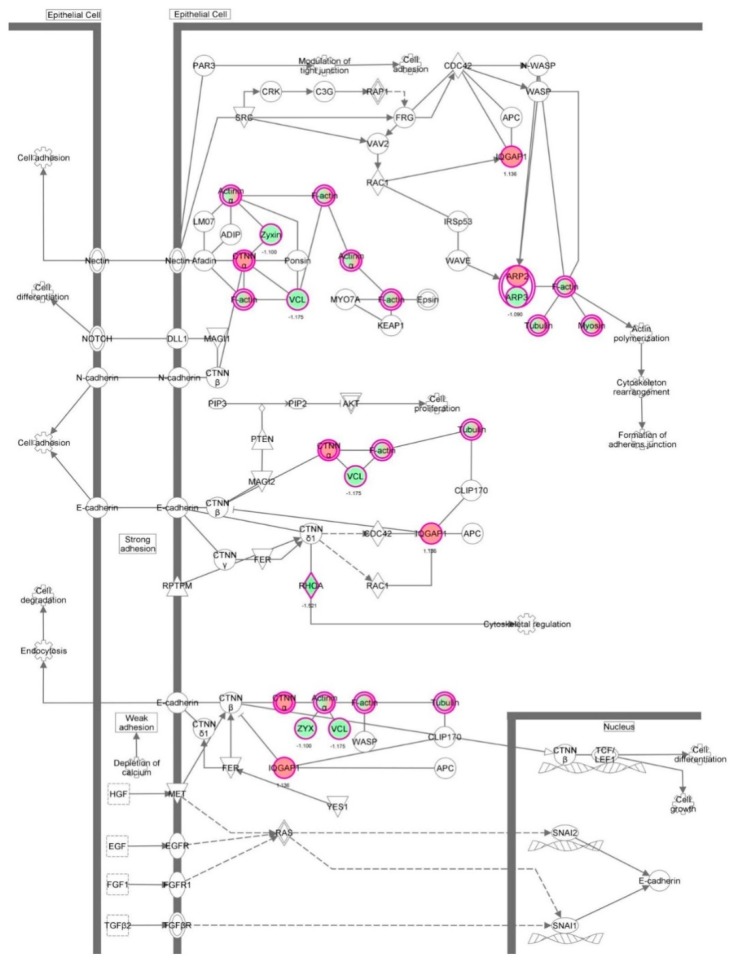
Epithelial adherent junction signaling pathway was regulated by vancomycin in HK-2 cells. HK-2 cells were treated with 50 μg/mL vancomycin for 24 h and the protein samples were subject to quantitative proteomic analysis. Red indicates an up-regulation; green indicates a down-regulation. The intensity of green and red colors indicates the degree of down- or up-regulation. Solid arrow indicates direct interaction and dashed arrow indicates indirect interaction.

**Figure 33:**
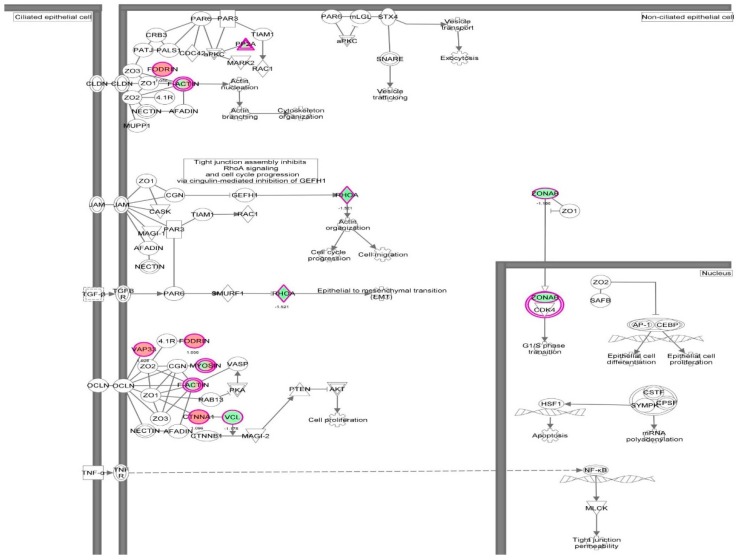
Tight junction signaling pathway was regulated by vancomycin in HK-2 cells. HK-2 cells were treated with 50 μg/mL vancomycin for 24 h and the protein samples were subject to quantitative proteomic analysis. Red indicates an up-regulation; green indicates a down-regulation. The intensity of green and red colors indicates the degree of down- or up-regulation. Solid arrow indicates direct interaction and dashed arrow indicates indirect interaction.

**Figure 34:**
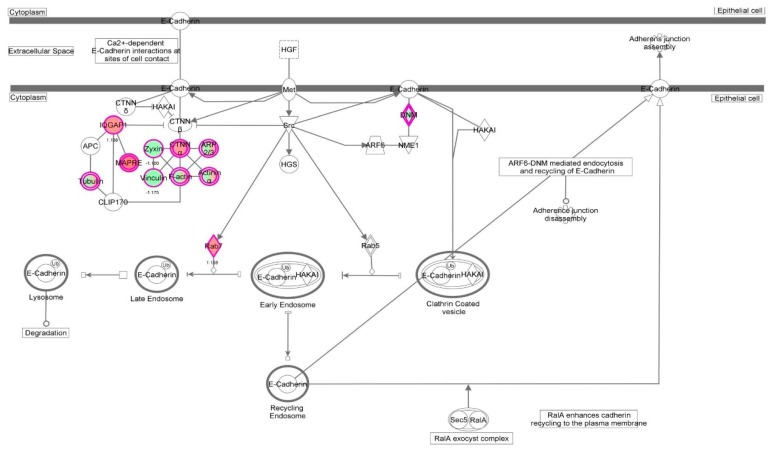
Remodeling of epithelial adherent junction signaling pathway was regulated by vancomycin in HK-2 cells. HK-2 cells were treated with 50 μg/mL vancomycin for 24 h and the protein samples were subject to quantitative proteomic analysis. Red indicates an up-regulation; green indicates a down-regulation. The intensity of green and red colors indicates the degree of down- or up-regulation. Solid arrow indicates direct interaction.

EMT has a close association with cell migration and invasion and it plays an important role in fibrosis and cancer development [19]. Suppressing the progress of EMT may represent a useful approach for the treratment of fibrosis and cancer. We analyzed the effect of vancomycin on EMT-related proteins and signaling pathways using SILAC-based proteomic approach. The proteomic data showed that vancomycin regulated the epithelial adherent junction signaling pathway (Figure 32), tight junction signaling pathway (Figure 33), and remodeling of epithelial adherent junction signaling pathway (Figure 34) in HK-2 cells involving a number of functional proteins, including ACTA1, ACTG1, ACTN1, ACTN4, ACTR2, ACTR3, ARPC2, ARPC1B, CTNNA1, DNM1L, IQGAP1, MYH9, MAPRE1, MYH10, PPP2CA, PPP2R1A, RAB7A, RHOA, TUBA1B, TUBB, TUBB6, TUBB8, TUBB4B, VCL, ZYX, SPTAN1, VAPA, VCL, and YBX3. Taken together, the proteomic data show that vancomycin markedly induces EMT in HK-2.

### 2.7. Vancomycin Regulates ER Stress Pathways in HK-2 cells

**Figure 35:**
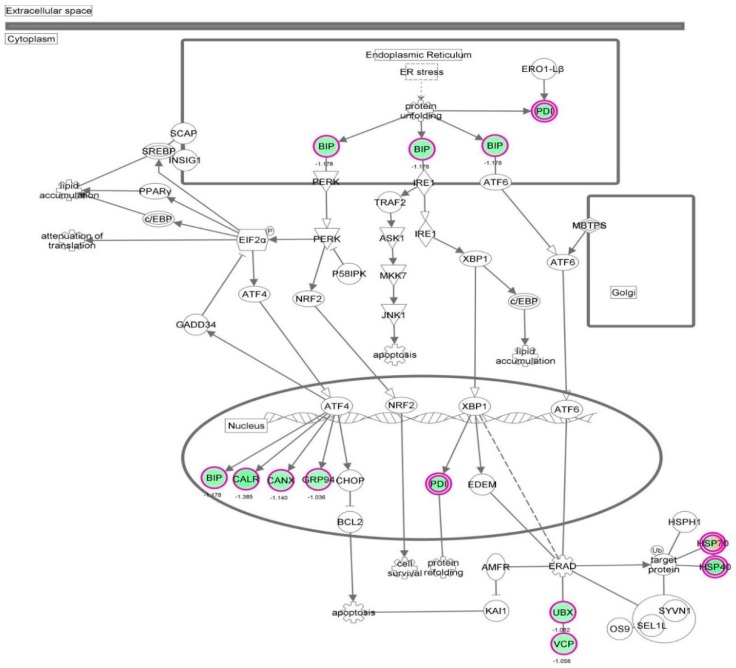
UPR signaling pathway was regulated by vancomycin in HK-2 cells. HK-2 cells were treated with 50 μg/mL vancomycin for 24 h and the protein samples were subject to quantitative proteomic analysis. Green indicates a down-regulation. The intensity of green color indicates the degree of down-regulation. Solid arrow indicates direct interaction.

**Figure 36:**
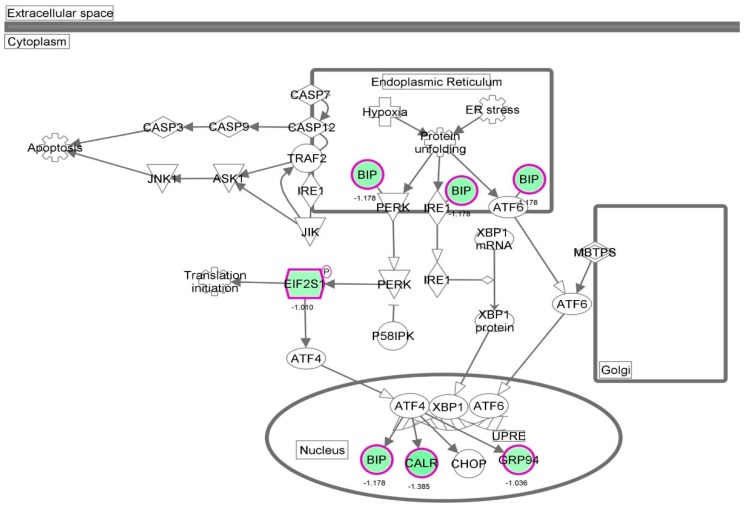
ER stress signaling pathway was regulated by vancomycin in HK-2 cells. HK-2 cells were treated with 50 μg/mL vancomycin for 24 h and the protein samples were subject to quantitative proteomic analysis. Green indicates a down-regulation. The intensity of green color indicates the degree of down-regulation. Solid arrow indicates direct interaction.

The unfolded protein response (UPR) is a cellular stress response related to the ER [20]. It is a stress response that has been found to be conserved between all mammalian species, as well as yeast and worm organisms [21]. The UPR is activated in response to an accumulation of unfolded or misfolded proteins in the lumen of the endoplasmic reticulum [20,21]. The proteomic data showed that vancomycin regulated UPR signaling pathway (Figure 35) and ER stress (Figure 36) in HK-2 cells. Incubation of HK-2 cells with vancomycin altered the expression level of several key proteins involved in UPR signaling pathway, including CALR, CANX, DNAJA2, HSP90B1, HSPA4, HSPA5, HSPA8, HSPA9, P4HB, UBXN4, and VCP. Furthermore, vancomycin altered the expression level of CALR, EIF2S1, HSP90B1, and HSPA5. Collectively, the regulatory effect of vancomycin on UPR and ER stress may contribute to vancomycin-associated nephropathy.

### 2.8. Vancomycin Regulates ERK-MAPK Signaling Pathway in HK-2 Cells

**Figure 37:**
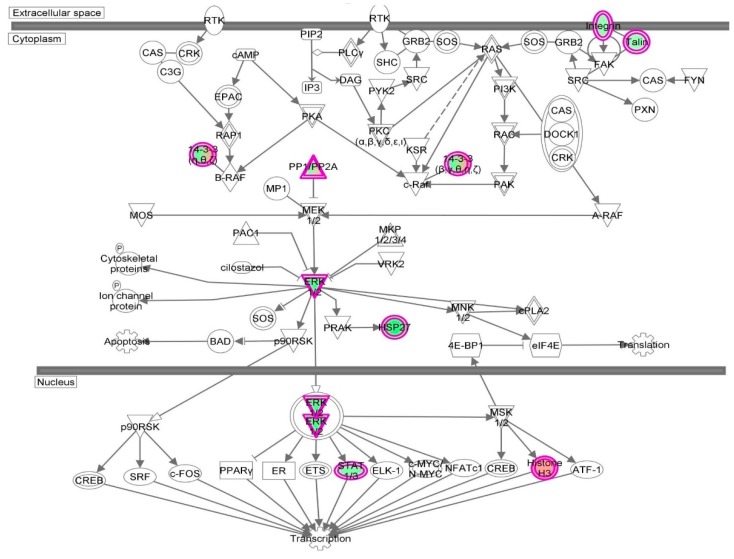
ERK-MAPK signaling pathway was regulated by vancomycin in HK-2 cells. HK-2 cells were treated with 50 μg/mL vancomycin for 24 h and the protein samples were subject to quantitative proteomic analysis. Red indicates an up-regulation; green indicates a down-regulation. The intensity of green and red colors indicates the degree of down- or up-regulation. Solid arrow indicates direct interaction and dashed arrow indicates indirect interaction.

Additionally, treatment of HK-2 cells with vancomycin induced the ERK-MAPK signaling response (Figure 37). There were a number of important proteins which were regulated by vancomycin in HK-2 cells. There was an increase in the expression level of H3F3A/H3F3B, PPP2CA, YWHAB, and YWHAQ, whereas there was a reduction in the expression level of HSPB1, ITGB1, MAPK1, PPP1CA, PPP2R1A, STAT1, TLN1, YWHAG, YWHAH, and YWHAZ. Taken together, the modulating effect of vancomycin on ERK-MAPK signaling pathway may contribute, at least in part, to vancomycin-induced nephrotoxicity.

## 3. Discussion

In the present study, we evaluated the global proteomic responses to vancomycin treatment with regard to cell cycle, programmed cell death, EMT and related molecular targets and signaling pathways in HK-2 cellsusing SILAC-based quantitative proteomic approach. The quantitative proteomic study showed that a large number of important proteins regulating cell proliferation, growth, cell death, and migration in HK-2 cells, with the involvement of a number of function proteins, such as CDK1, CDK2, E-cadherin, PI3K, Akt, mTOR, cytochrome c, caspase 9, caspase 3, Bcl-2, Bax, p53, PPAR, HSP, Erk1/2, Ras, and Rho.

This proteomic study also showed that vancomycin regulated mitochondrial function and cell death. Mitochondrial disruption and the subsequent release of cytochrome c initiate the process of apoptosis, with the latter being initiated by pro-apoptotic members of the Bcl-2 family but antagonized by anti-apoptotic members of this family [22,23]. Anti-apoptotic members of Bcl-2 can be inhibited by post-translational modification and/or by increased expression of PUMA, which is an essential regulator of p53-mediated cell apoptosis [24]. In addition, cytochrome c released from mitochondria can activate caspase 9, which then activates caspase 3 and caspase 7 [25]. In our study, we observed that vancomycin regulated expression level of a number of proteins, such as CAPN1, CDK1, and MAPK1. Furthermore, vancomycin regulated apoptotic signaling pathway via increasing the expression level of CAPN2, CAPNS1, LMNA, and SPTAN1 in HK-2 cells. 

Furthermore, the proteomic results show that vancomycin exhibits a modulating effect on PI3K/Akt/mTOR signaling pathway. Under optimal growth conditions, activated mTORC1 inhibits autophagy by direct phosphorylation of Atg13 and ULK1 at Ser757 [26,27,28]. This phosphorylation inhibits ULK1 kinase activity and subsequent autophagosome formation. When the kinase activity of mTORC1 is suppressed, the autophagic machinery is initiated. In the present study, vancomycin regulated autophagy in HK-2 cells as indicated by the alteration in the expression of HSP90B1, ITGB1, MAPK1, PPP2R1A, SFN, YWHAE, YWHAG, YWHAH, and YWHAZ. Taken together, the autophagy-modulating effect of vancomycin may contribute to its nephrotoxic effect via the regulation of PI3k/Akt/mTOR signaling pathway.

EMT is characterized as epithelial cells to lose their polarization and specialized junction structures, undergoing cytoskeleton reorganization and acquiring morphological and functional features of mesenchymal-like cells [19,29]. In our proteomic study, we observed marked regulatory effects of vancomycin on the expression of a number of functional proteins that modulate epithelial adherent junction signaling pathway in HK-2 cells. Again, the SILAC-based quantitative proteomic analysis can discriminate the role of EMT modulation in vancomycin-associated nephrotoxicity.

Our data have provided new insights into the molecular mechanisms of vancomycin-induced nephrotoxicity that is often observed in clinical practice. Our data are consistent with previously observed biochemical changes at cellular levels induced by vancomycin in renal epithelial cells. Our data may help identify new targets that are useful for discovery of new therapeutic approaches for vancomycin-induced nephrotoxicity. Further functional studies are warranted to validate our proteomic data.

## 4. Materials and Methods

### 4.1. Chemicals and Reagents

Vancomycin, ^13^C_6_-l-lysine, l-lysine, ^13^C_6_^15^N_4_-l-arginine, l-arginine, Dulbecco’s phosphate buffered saline (PBS), heat inactivate fetal bovine serum (FBS), and dialyzed FBS were purchased from Sigma-Aldrich (St. Louis, MO, USA). DEME/F12 medium was bought from Invitrogen Inc. (Carlsbad, CA, USA). FASP™ protein digestion kit was purchased from Protein Discovery Inc. (Knoxville, TN, USA). The proteomic quantitation kits for acidification, desalting, and digestion, Ionic Detergent Compatibility Reagent (IDCR), DMEM/F12 medium for SILAC, Pierce bicinchoninic acid protein assay kit, and Western blotting substrate were obtained from Thermo Fisher Scientific Inc. (Hudson, NH, USA).

### 4.2. Cell Culture and Treatment

The human proximal tubule epithelial cell line HK-2 was purchased from the American Type Culture Collection (Manassas, VA, USA) and maintained in regular DMEM/F12 (1:1) medium supplemented with 10% heat-inactivated FBS at 37 °C in a 5% CO_2_/95% air humidified incubator. Cells were seeded into the plates for 24 h to achieve a confluence of ~80% prior to vancomycin treatment. Vancomycin was dissolved in DMSO with a stock concentration of 50 mM, and was freshly diluted to the pre-determined concentrations with culture medium with 0.05% (*v*/*v*) final concentration of DMSO.

For proteomic analysis, HK-2 cells were cultured in DMEM/F12 medium (for SILAC) with (heavy) or without (light) stable isotope labeled amino acids (^13^C_6_-l-lysine and ^13^C_6_^15^N_4_-l-arginine) and 10% dialyzed FBS. HK-2 cells cultured in SILAC medium for six cell doubling times to achieve a high level (>98%) of labeled amino acid incorporation. Then the cells grown in “light” media were treated with 0.5% DMSO for 24 h to serve as the negative control; cells grown in “heavy” media were treated with pre-determined vancomycin for 24 h. All the experiments were performed three times independently. After vancomycin treatment, HK-2 cell samples were harvested, lysated with hot lysis buffer (100 mM Tris base, 4% SDS, and 100 mM dithiothreitol), and denatured for 5 min at 95 °C. Then, the samples were stored at −80 °C till further analysis.

### 4.3. Digestion and Desalting SILAC Protein Samples

Prior to the quantitative proteomic analysis, the protein samples were subject to digestion and desalting which were performed using SILAC-based approach as described previously [8,10,30]. Briefly, the thermal denatured cell lysate was subject to a brief sonication at 20% AMPL for 3 s with 6 pulses. The sonicated samples were centrifuged at 15,000× *g* for 20 min at room temperature and the supernatant was collected. The protein concentration was determined using IDCR. Subsequently, equal amount of heavy and light protein sample was combined to reach a total volume of 30–60 μL containing 300–600 μg protein. The combined protein sample was digested using FASP™ protein digestion kit. A quota of 40 μL of combined lysate was added to a spin column prewetted with 8 M urea. The lysate buffer was exchanged to 8 M urea using centrifugation prior to iodoacetamide alkylation and then exchanged to 50 mM ammonium bicarbonate for trypsin digestion at a ratio of 1:100 (*w*/*w*). Digestion was carried out overnight in a humidified incubator at 37 °C and terminated with the addition of 5% formic acid. After the digestion, the resultant sample was acidified to pH of 3 and desalted using a C_18_ solid-phase extraction column. Samples were loaded and washed in 0.1% formic acid and eluted using 90% acetonitrile/10% formic acid. Samples were concentrated in a vacuum concentrator at 45°C for 120 min and resuspended in 0.1% formic acid prior to LC-MS/MS analysis.

### 4.4. Liquid Chromatography-Tandem Mass Spectrometry (LC-MS/MS) and Statistical Analysis

The concentrated samples (5 μL) were subject to the hybrid linear ion trap-Orbitrap (LTQ Orbitrap XL, Thermo Scientific Inc.). LC-MS/MS was performed using a 10 cm-long 75 μm (inner diameter) reversed-phase column packed with 5 μm-diameter C_18_ material with 300 Å pore size (New Objective, Woburn, MA, USA) with a gradient mobile phase of 2%–40% acetonitrile in 0.1% formic acid at 200 μL/min for 125 min. The Orbitrap full MS scanning was performed at a mass (*m*/*z*) resolving power of 60,000, with positive polarity in profile mode [M + H]^+^. Raw data files were submitted for simultaneous search using standard SILAC settings for LTQ Orbitrap XL instruments. Proteins for which at least two fully trypsin-digested L and H peptides were detected at >99% confidence were used for subsequent comparative quantitative analysis. Peptide SILAC ratio was calculated using MaxQuant version 1.2.0.13. The SILAC ratio was determined by averaging all peptide SILAC ratios from peptides identified of the same protein. Differential regulation within each experimental data set was determined by normalization of each data set. Briefly, the L/H ratio was converted into log_2_ space to determine geometric means and facilitate normalization. The average log_2_ L/H ratios and standard deviation of the log_2_ L/H ratios were determined for each data set, both before and after computational removal of the few significant outliers found in a few data sets. Every protein’s log_2_ L/H ratio was then converted into a z-score that is the measure of how many standard deviation units (expressed as “σ”) that protein’s log_2_ L/H ratio is away from its population mean. Therefore, a protein with a z-score of >1.645σ indicates that that protein’s differential expression lies outside the 90% confidence level, >1.960σ indicates that it is outside the 95% confidence level, 2.576σ indicates 99% confidence, and 3.291σ indicates 99.9% confidence. *z*-Scores of >1.960 were considered significant.

### 4.5. Pathway Analysis and Bioinformatics

The protein IDs were identified using Scaffold 4.3.2 from Proteome Software Inc. (Portland, OR, USA) and the pathway was analyzed using Ingenuity Pathway Analysis (IPA) from QIAGEN (Redwood City, CA, USA). Geometric means for total protein expression ratios across biological samples were calculated respective to intensity. Geometric means and Uniprot Protein identification numbers were uploaded to Ingenuity Pathway Analysis (IPA) to determine localization, molecular function, and protein interactions. Upstream regulator analysis was also performed within IPA where activity of potential upstream regulators is predicted based on the expression profile of known down-stream targets in relation to known upstream regulatormediated expression changes reported in the literature. This analysis determines the significance of overlap of the detected targets with the upstream regulator through a Fisher’s exact test in addition to implementation of a *z*-score algorithm to make prediction of the direction of upstream regulator activity change. Description of the *z*-score algorithm is available on the IPA Web site (www.ingenuity.com).

### 4.6. Statistical Analysis

Data are expressed as the mean ± standard deviation (SD). Multiple comparisons were evaluated by one-way analysis of variance (ANOVA) followed by Tukey’s multiple comparison. A value of *p*<0.05 was considered statistically significant.

## 5. Conclusions

In summary, the quantitative SILAC-based proteomic approach showed that vancomycin regulated cell proliferation, mitochondria-dependent apoptotic pathway and autophagy, and EMT in HK-2 cells, involving a number of key functional proteins and related molecular signaling pathways. This study may provide a clue to fully identify the molecular targets and elucidate the underlying mechanism of vancomycin-associated nephrotoxicity, resulting in an improved therapeutic effect and reduced side effect in clinical settings.
